# Changes to the Fossil Record of Insects through Fifteen Years of Discovery

**DOI:** 10.1371/journal.pone.0128554

**Published:** 2015-07-15

**Authors:** David B. Nicholson, Peter J. Mayhew, Andrew J. Ross

**Affiliations:** 1 Department of Biology, University of York, York, United Kingdom; 2 Department of Natural Sciences, National Museum of Scotland, Edinburgh, United Kingdom; Onderstepoort Veterinary Institute, SOUTH AFRICA

## Abstract

The first and last occurrences of hexapod families in the fossil record are compiled from publications up to end-2009. The major features of these data are compared with those of previous datasets (1993 and 1994). About a third of families (>400) are new to the fossil record since 1994, over half of the earlier, existing families have experienced changes in their known stratigraphic range and only about ten percent have unchanged ranges. Despite these significant additions to knowledge, the broad pattern of described richness through time remains similar, with described richness increasing steadily through geological history and a shift in dominant taxa, from Palaeoptera and Polyneoptera to Paraneoptera and Holometabola, after the Palaeozoic. However, after detrending, described richness is not well correlated with the earlier datasets, indicating significant changes in shorter-term patterns. There is reduced Palaeozoic richness, peaking at a different time, and a less pronounced Permian decline. A pronounced Triassic peak and decline is shown, and the plateau from the mid Early Cretaceous to the end of the period remains, albeit at substantially higher richness compared to earlier datasets. Origination and extinction rates are broadly similar to before, with a broad decline in both through time but episodic peaks, including end-Permian turnover. Origination more consistently exceeds extinction compared to previous datasets and exceptions are mainly in the Palaeozoic. These changes suggest that some inferences about causal mechanisms in insect macroevolution are likely to differ as well.

## Introduction

A key contribution of palaeontology to the study of the diversity of life has been the elucidation of macroevolutionary patterns and processes through deep time, with fossils providing the only direct temporal evidence of how life has responded to a variety of biotic and abiotic forces [1–4]. If there are general rules underlying macroevolutionary responses to these forces, studying the past may also inform the future. Palaeontology can therefore, potentially, provide important information on the future progression of the extinction crisis facing the biosphere today, and its likely consequences [1,5].

In addition to such strategic questions, palaeontological data can help solve many basic questions of perennial interest. Comprising over 50% of described species [6], hexapods (insects and their close relatives such as springtails) form a major component of almost all continental ecosystems. An explanation of how and why this group has come to so dominate terrestrial biodiversity is a major challenge in macroevolutionary biology.

Palaeodiversity data are typically compiled in the form of taxonomic databases of fossils that provide either temporal ranges or discrete occurrence data. Commonly, criticisms of such databases focus around the integrity of the data and its resilience to the addition of further information [7]. Substantial additional knowledge, both taxonomic and stratigraphic, of the fossil records of tetrapods [8] and all marine animal families [9], has nonetheless yielded very similar variation in originations and extinctions though time. This supports the notion that broad biological signals can be seen through the statistical noise of an imperfect fossil record, providing the error is randomly distributed [10]. However, the effect of additional data on macroevolutionary patterns has not been tested for the majority of continental groups. This is important because many terrestrial taxa, such as insects, preserved primarily in exceptional conditions (*Lagerstätten* taxa) are likely to have substantially incomplete fossil records where the potential for change is much greater.

Using data on the temporal ranges of families, Labandeira [11] and Labandeira and Sepkoski [12] considered that, apart from the Late–end-Permian extinction, no other mass extinction event known from other groups appears to have had any major impact on insects at the family level. In addition, a steady increase of insect family-level richness began in the Triassic and was attributable not to particularly high levels of origination, but to consistently low extinction–noticeably lower than that in the Palaeozoic. The rise of angiosperms during the Cretaceous apparently did not cause any increase in family diversity in insects and may even have caused some decline in richness into the Late Cretaceous. However, Labandeira and Sepkoski [12] noted that much of the variation around this long term trend of increasing richness could be linked to specific rich fossil deposits (*Lagerstätten*) or stages where insect-bearing fossil deposits are poorly known and so were cautious with any such interpretations. Jarzembowski and Ross [13], using data based on but slightly updated from Ross and Jarzembowksi [14], highlighted four major insect origination events; during the Permo-Carboniferous, Early Jurassic, Early Cretaceous and the Eocene. They concurred with Labandeira and Sepkoski [12] that today’s exceptionally high insect diversity is the result of low extinction levels and sustained origination but disagreed that insects were essentially immune to mass extinction after the end-Permian event. Highlighting in particular an apparent decline in family richness seen in the Upper Cretaceous record, they suggest a causal link to the radiation of angiosperms. Additionally, Ross *et al*. [15] noted the increase in counts of origination and extinction in the Cretaceous as evidence of ecological turnover associated with angiosperms.

The field of palaeoentomology has expanded rapidly in the last two decades, with large increases in the number of active researchers and consequent publication output [16], as well important changes in taxonomy (e.g. the resurrection of the order Cnemidolestodea [17]), the dating of fossil deposits (e.g. the recognition of the mid-Cretaceous age of Burmese amber; see [18,19]) and the exploration of newly discovered insect-bearing formations globally (e.g. the Eocene amber deposits of India [20]).

To take account of these developments, in the first instance, a new dataset of the temporal ranges of hexapod families, compiled from literature (about 2,500 papers) published up to the end of 2009, is compared with that of Ross and Jarzembowski [14] (data from literature published up to the end of 1991) and Labandeira [11] by documenting changes and additions to the data. Then richness time series derived from these datasets are compared to assess any change in the signal provided by the fossil record in light of additional data. Although many recent studies of fossil richness through time have been derived from sample-based occurrence data (e.g. [1]), which facilitates the elimination of biases, studies of the face-value record are still valuable from a comparative perspective: they will, for example, help in the understanding of sampling-based artefacts by comparison with the geological record (e.g. [21,22]). In addition, taxonomic range data still retain considerable utility for the dating of phylogenies. There have to-date been no sample-standardized studies of fossil insect richness, and, although constraints on the completeness and comparability of data may limit what can currently be achieved, we expect that future studies will attempt them. A breakdown of the new data show which main groups of hexapods make a dominant contribution to the signal through time. From the first and last occurrence data, rates of origination and extinction can be calculated per stage indicating the timing of major radiation and extinction events as well as long-term trends and the relative importance of these to hexapod family richness.

## Methods

### Changes and additions to the hexapod fossil record

We quantified the amount of change in the new dataset (NEW) relative to the fossil insect family datasets presented by Ross and Jarzembowski [14] (downloaded from www.fossilrecord.net 2012-10-05) and Labandeira [11] (referred to herein as FR2 and LAB, respectively). The NEW dataset is dated according to the International Commission on Stratigraphy stages [23]. First, each family in NEW was categorised in the following ways with respect to FR2 and LAB: ‘no change’, ‘new in list’ and ‘range change’. The first of these is self-explanatory with respect to LAB, which, like NEW, presents data at stage resolution. However, FR2 presents data at both epoch and stage level, and no change for a family where data in FR2 were given at epoch or period level represents a case where the data in NEW confirm it was indeed present throughout that epoch or period. ‘New in list’ can refer to newly described families, those brought out of synonymy or Recent families which now have a fossil record. ‘Range change’, used only for comparison with FR2, involves a change in the recorded stratigraphic range of a family, whether an extension or contraction from the finding of new specimens but also includes improved resolution or revised dating of deposits from which previously known specimens occur (i.e. the deposit is now dated to a different stage). Since most of the LAB data is resolved to stage level and so is more directly comparable with the new data, range change was subdivided into three categories: contraction, extension and shift. A contraction is any situation where the NEW range has fewer stages than recorded in LAB, while an extension is any family where the new range covers a greater number of stages. This does not distinguish between whether the first and/or last occurrence has changed to create the contraction or extension and can also include instances where the NEW range has no overlap with that in LAB, e.g. the palaeodictyopteran family Hanidae, P1(Artinskian) in LAB but C2(Gzhelian)–P1(Sakmarian) in the new dataset. Shifts represent when the NEW range for a family covers a different set but the same total number of stages.

### Derivation of richness time series from origination and extinction data

**Fig 1:**
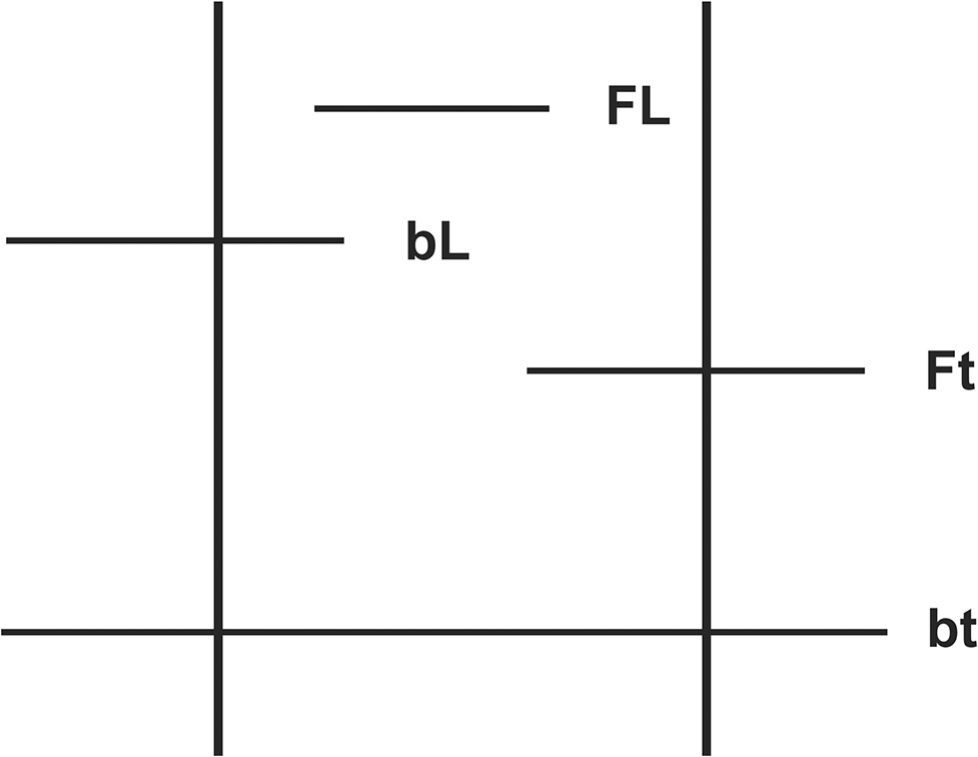
Four classes of taxa recorded in an interval using first and last occurrence data. After Foote [24]. The horizontal axis represents time progressing from left to right. The vertical lines represent the start (left) and end (right) of a specified time interval of interest. Horizontal lines represent the temporal ranges of four types of taxa of interest: **FL** originates and goes extinct within the interval, **bL** originates before and becomes extinct within the interval, **Ft** originates within and continues beyond the interval and **bt** originates before and continues after the interval.

Before describing how various time series can be derived from first and last occurrence data, it is worth defining the four classes of taxa which can be counted in a time interval [24] (Fig 1).

Some taxa (bt: bottom, top) originate before the time interval in question and have their last occurrence sometime after it, thus crossing the bottom and top boundaries. Some taxa (bL: bottom, Last) originate before the interval and have their last occurrence within it. Others (Ft: First, top) first appear in the interval and range beyond it. Finally, still others (FL: First, Last–also known as single-interval taxa) appear to originate and go extinct entirely within the interval, never crossing either the bottom or top boundaries. The term ‘single-interval taxon’ is preferable to the commonly used term ‘singleton’ when describing such taxa (as unfortunately done in e.g. [24–26]) as the word is already in common usage in ecology for taxa represented by one specimen [27,28].

Two commonly-used counting methods exist for deriving diversity time series from first and last occurrence data–range through (RT) and boundary crossers (BC), and a third employed here, minimum assumption (MIN) [22,28]. These are applied to NEW and LAB data, while with the FR2 data only RT is used but under two assumptions–FR2^+^ and FR2^–^, explained below.

RT is the classic method of counting a taxon as present in every stage between and including its first and last occurrences in the fossil record (or up to the present day if still extant), as well as those which originate and go extinct within the same time interval (known as single-interval taxa or FL in the notation given above), used, for example, by Sepkoski [9], Labandeira and Sepkoski [12] and Jarzembowski and Ross [13]. This is the sum total of taxa observed and inferred to exist within a time interval and can be written as RT = bt+Ft+bL+FL. For FR2, inconsistent stratigraphic resolution makes it necessary to use maximum and minimum assumptions of the ranges given when comparing with datasets at stage level. FR2^+^, then, is based on the assumption that the family originates in the first stage of the interval in which lies its first appearance and goes extinct in the last stage of the interval containing its last appearance, while FR2^–^ assumes the origination in the last stage of the interval of first appearance and extinction in the first stage of the interval of last appearance [5]. Consequently, any family which is recorded at epoch or period level but in only one interval is removed from the FR2^–^ series.

The BC series are made up of only those taxa which range between two or more time intervals, i.e. excluding single-interval taxa (FL). However, they are not simply RT minus FL. Rather, BC series represent the number of taxa crossing the bottom boundary into the interval, thereby tying diversity to a single point in time (the boundary) and not adding that diversity to events which occur cumulatively within the interval. It can be written as BC = bt+bL. By restricting the richness count to taxa which cross a single point in time, the data record an actual faunal cohort rather than the accumulation of taxa which exist throughout an interval. The specific advantage of this is that it is immune to changes in interval length, while it might be expected that longer intervals will accumulate more taxa than shorter ones, thereby inflating the richness measurement for that observation point. BC series have found use in some more recent palaeodiversity studies [29–31] and have been advocated within the palaeoentomological community more recently by Ponomarenko and Dmitriev [32]. As these are values for interval boundaries, in order to make possible the comparison with data within intervals (placed at stage-midpoint) the geometric mean of the bottom and top boundaries of each interval are used for analyses, i.e.

$$\sqrt{{BC}_{1}\times {BC}_{2}}$$

where *BC*_1_ and *BC*_2_ are the number of bottom and top boundary crossers of a given interval, respectively. Possible drawbacks of excluding single-interval taxa are that it excludes some true biological variation; may increase taxonomic bias by virtue of eliminating particular types of organism from the data; and the data then cease to represent all described variation, which is one of their chief merits.

The MIN series is derived from only the first, last and single-interval taxa, without filling in ranges. Like RT, this is a summation of events within a stage and can be written as MIN = Ft+FL+bL. This is the most conservative of the three as it makes the minimum assumption of what has actually been recorded in each stage and is more directly related to sampling proxies such as formation or collection counts [22]. It can be viewed as a subset of sampled-in-bin counts (counting only taxa which have actually been recorded in a time bin, rather than merely inferred to have existed at that time). Of course, it is a highly truncated version of true sampled-in-bin counts as the original purpose of the dataset was to record only first and last occurrences.

To quantify the similarity between the new and previous datasets, untransformed RT data from FR2, LAB and NEW are assessed using Spearman’s rank correlation because, even when logged, the data were skewed, breaking parametric assumptions. The normal associated probabilities are not reported because autocorrelations in the data invalidate them. Bootstrap estimates for significance of correlations, which reduce the necessary set of assumptions about the data, are instead calculated using the boot.ci function from the boot library in R to re-sample the original data 9999 times, each time recalculating the correlation coefficient, to generate a bootstrapped distribution of the test statistic which indicates the extent of uncertainty in it. Confidence intervals at the 95% and 99% level are calculated using the bca (bias corrected and accelerated or BC_*a*_) method due to Efron [33], which corrects for the bias (the difference between the mean of the bootstrap replicates and the true correlation) and asymmetry of the bootstrap distribution [33]. Where the confidence intervals do not bracket zero, the correlation can be said to be significantly different from zero. Note that in the case of “dependent” data such as time-series, bootstrapping may still not accurately estimate confidence intervals around statistical parameters, so, whilst likely more valid than the standard p-values, should still be treated with caution. Correlations were also explored for two detrended versions of each time series: first differencing explores the changes between successive time steps (stages), whilst generalized differencing (first differencing of the residuals from linear regression) quantifies the successive changes after removing the overall long term trend. Differences were calculated using the statistical programming language R [34]. All correlations are on data from the Serpukhovian (top of Early Carboniferous, stage midpoint 323.2Ma) to Piacenzian (top of the Pliocene, stage midpoint ~3.1Ma), as this is the range for which there is a reasonable fossil record of hexapods (i.e. including the long period of almost no record before the Carboniferous would increase all of the coefficients simply from a lack of data).

### Calculating origination and extinction rates

The rates of origination and extinction employed here are Foote’s [24] estimated per-capita rates, $\hat{p}$ and $\hat{q}$ respectively. They are derived as follows:

$$\hat{p}=-\text{ln}(\frac{N_{bt}}{N_{t}})/\Delta t$$

$$\hat{q}=-\text{ln}(\frac{N_{bt}}{N_{b}})/\Delta t$$

where *N*_*t*_ is the total number of taxa crossing the top boundary out of the interval (i.e. bt+Ft), *N*_*b*_ is the total number crossing the bottom boundary into it (i.e. bt+bL) and *N*_*bt*_ is the number of taxa crossing both the bottom and top boundary. The advantage of using these over counts of events within an interval is that they are robust to variation in interval duration, disregard single-interval taxa (which are prone to disproportionately distort the signal) and are independent of each other as they are derived from numbers of taxa passing into and out of intervals rather than the addition of events taking place within them. Due to inconsistent stratigraphic resolution, this is not attempted for the FR2 data.

## Results

### Changes in the data

**Fig 2:**
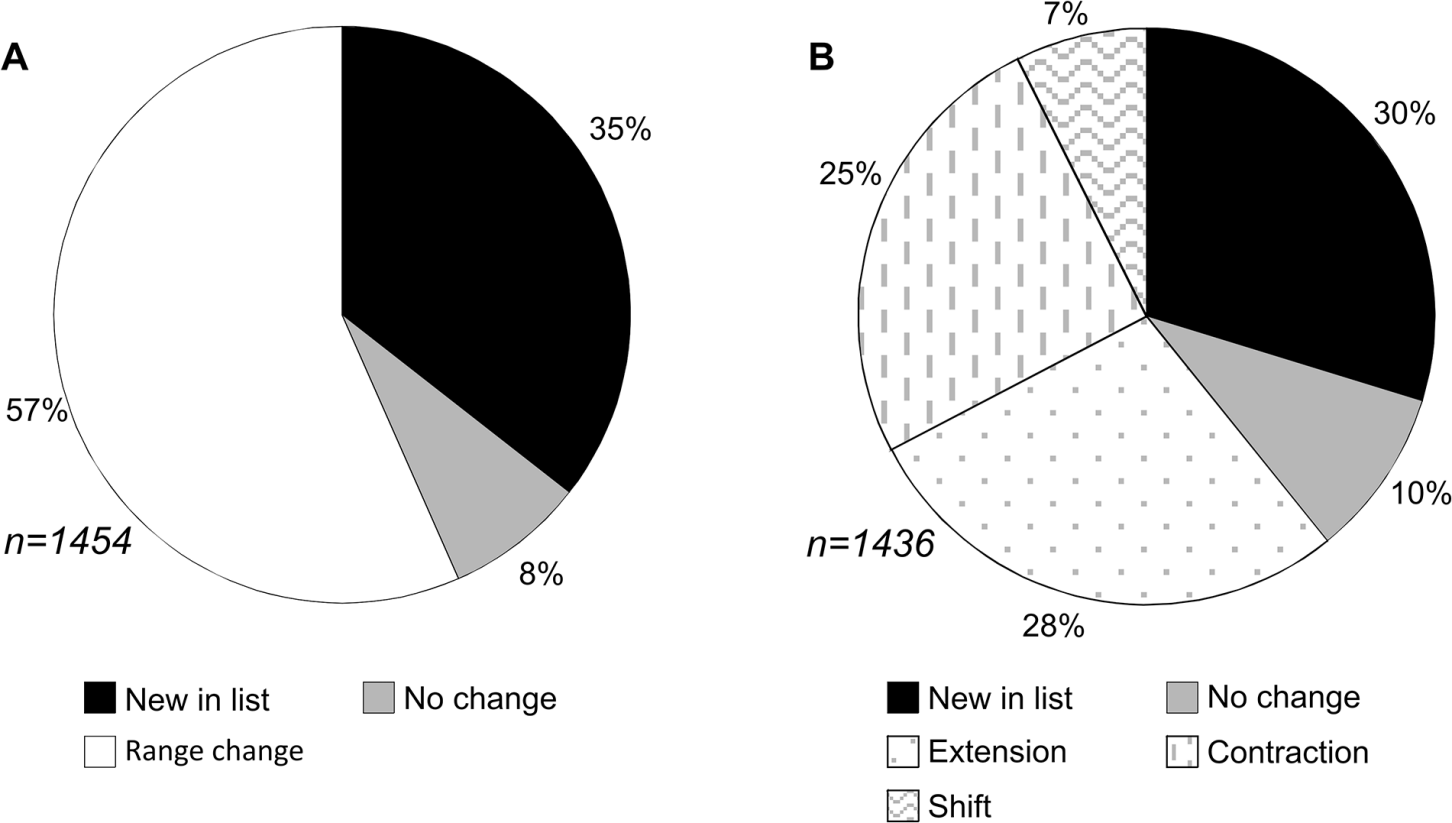
Proportions of changes in new data for family stratigraphic range compared with previous datasets. (A) FR2 [14] all hexapods and (B) LAB [11] all insects.

The NEW dataset (S1 Appendix and S1 Dataset) contains a total of 1454 families of Hexapoda, of which 1436 are Insecta. In comparison to FR2, a substantial amount of change has left only 8% of families with the same ranges as recorded in 1993; 35% are new to the record, and well over half have a change in the recorded range (Fig 2A). The picture is broadly similar when compared to LAB (Fig 2B), with 10% remaining unchanged and 30% new. The majority of the range changes are made up of roughly equal amounts of extensions and contractions, and only 7% of the total representing a shift in range. Although the NEW dataset has a higher total number of families (1454) than either FR2 (1087 in [14]) or LAB (1272; 1276 if including ‘uncertain’ families), 230 and 263 families listed in FR2 and LAB, respectively, are included in NEW within other families (as synonyms or subfamilies), or no longer have a fossil record, due mostly to taxonomic revisions.

### Richness series from new and previous datasets

**Fig 3:**
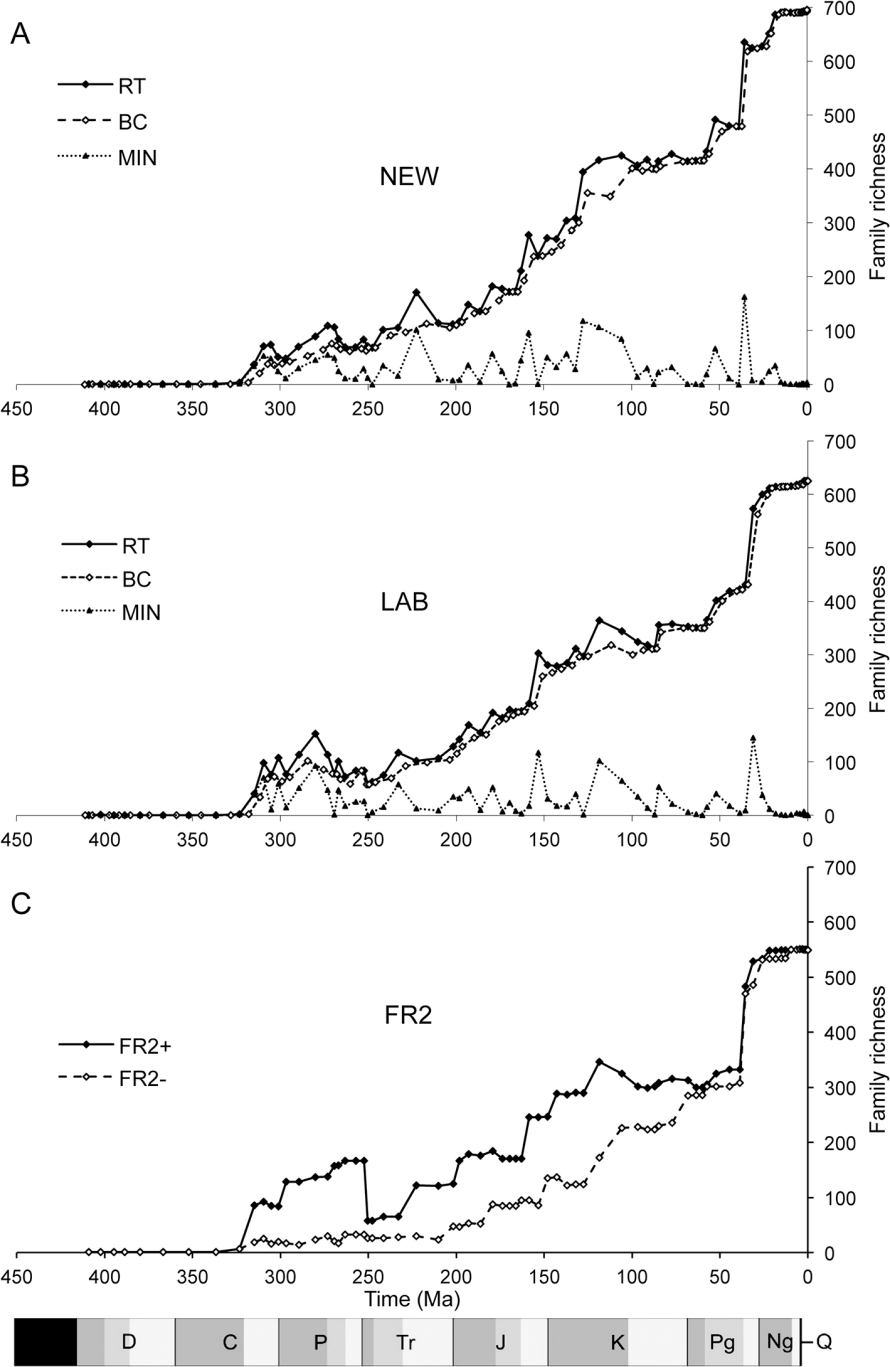
Family richness of insects through time. Richness time series derived from (A) NEW data, presented here, (B) LAB data from Labandeira [11] and (C) FR2 data from Ross and Jarzembowski [14]. RT = range through, i.e. all taxa ranging anywhere into an interval, with maximum (+) and minimum (–) assumptions for FR2, plotted at stage-midpoints. BC = boundary crossers, i.e. taxa crossing interval boundaries, plotted at stage boundaries. MIN = minimum richness, representing firm occurrences within stages (i.e. first, last and single-interval taxa records).

The richness time series of all three datasets show broad similarities in long-term trends of increasing richness and the synchronicity (or nearly so) of several pulses (Fig 3) but some differences are worth noting.

For the Palaeozoic, the RT series from NEW and LAB are more similar to each other than to FR2^+^. However, the NEW series shows consistently lower richness than LAB and the two main peaks are offset by one stage, reaching a maximum of 109 families by NEW RT (Kungurian: 273 Ma) and 153 by LAB RT (Artinskian: 280 Ma) (Fig 3). FR2^+^ shows a gradual and steady increase in richness through the Palaeozoic with a dramatic drop at the end-Permian (~250 Ma), after reaching a maximum of 168 families (Fig 3). FR2^–^ shows no such increase and decline but rather remains conspicuously flat through until the Late Triassic at around 210 Ma (Norian). This is also not mirrored by LAB RT and NEW RT, which show slightly less sharp declines from the Early–Middle Permian towards the end-Permian, when a small increase is seen in the final stage (Changhsingian, data point at 252 Ma). The BC series in NEW and LAB mirror the peaks and troughs of the RT curves but they are less pronounced (Fig 3).

In the Triassic (251–200 Ma) all three datasets show a marked increase in richness, with the largest increase in the Carnian (223 Ma) for FR2^+^ (up to 123 families) and NEW (171 families) and in the Ladinian (233 Ma) for LAB (117 families) (Fig 3). The NEW RT series show the most pronounced Triassic peak followed by an apparent crash in richness, mirrored in the NEW MIN series but NEW BC shows a smooth increase with only a slight decrease after the Carnian, reflecting that many of the records are single-interval taxa.

**Fig 4:**
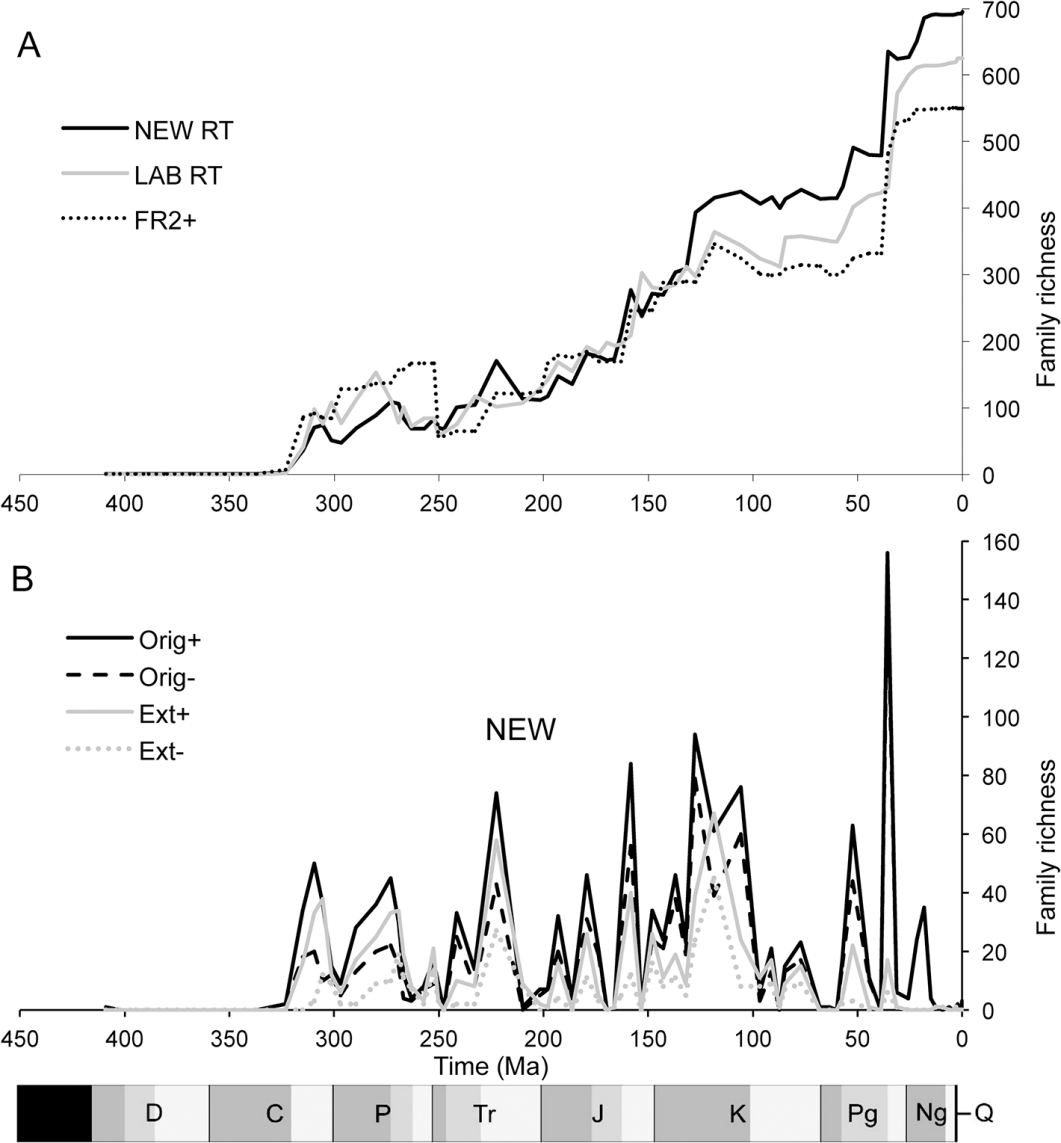
Richness and rates of origination and extinction in fossil hexapods. (A) Range through time series for NEW, LAB and FR2. (B) Origination (Orig) and extinction (Ext) counts, both including (+) and excluding (–) single interval taxa, from NEW.

The Jurassic (200–146 Ma) continues the long-term increase in described richness (Fig 4). The NEW RT series shows a distinct, four-pulsed increase at 193 (Sinemurian), 179 (Toarcian), 158 (Oxfordian), and 148 Ma (Tithonian); the first three are followed by drops in richness, although this is not reflected in the BC series which shows an uninterrupted, fairly smooth increase. An almost identical pattern is seen in LAB RT while FR2^+^ shows two distinct increases followed by plateaus.

During the Early Cretaceous (146–100 Ma) a more rapid rise is seen, most steeply in NEW RT. LAB and FR2 are similar in then showing a pronounced and sustained drop in richness after their synchronous peaks in the Aptian (point at 119 Ma) in both RT and BC series while the NEW RT series continues to increase, albeit at a decelerated rate until it plateaus across a similar range of stages as LAB and FR2. This plateau is accompanied by very low values in the NEW MIN series. No marked drop in richness is apparent at or near the Cretaceous/Palaeogene boundary (65.5 Ma).

The NEW RT series averages 15% and 26% higher across the Cretaceous and Tertiary compared with LAB and FR2, respectively ending with maxima of 695 (NEW), 549 (FR2) and 625 (LAB) families. All three show the most rapid increase in richness in the entire fossil record through the Tertiary with very little deviation between RT (or ^+^) and BC (or^−^) series.

**Fig 5:**
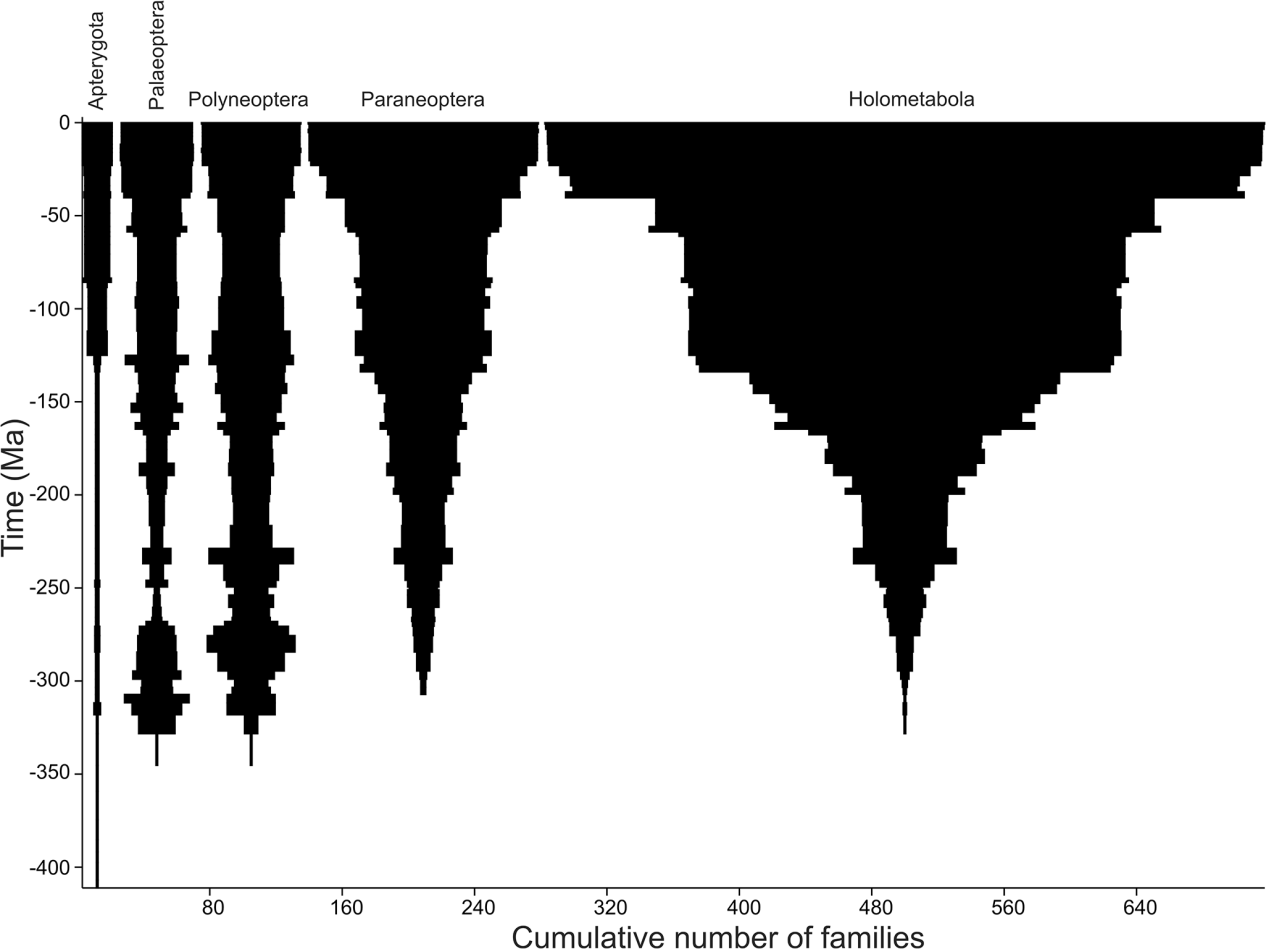
Spindle diagram showing NEW range-through family richness in major constituent hexapod groups through time. Generated using PAST [69].

The major taxa dominating richness in NEW (RT) varied at different times (Fig 5). The earliest known hexapod families are in the ‘Apterygota’. These contribute very little to hexapod fossil richness in the long term. The Carboniferous and Permian peaks and subsequent declines are seen only in the Palaeoptera and Polyneoptera. Paraneoptera and Holometabola had originated before the Permian peak but show no sign of any decline towards the end-Permian, rather a slow but steady increase in richness (Fig 5). The Late Triassic peak seen in the RT (but not BC) series is apparent in all groups except Apterygota. Except for occasional pulses of increased richness, which are synchronous with the other three major contributing groups, Palaeoptera show very slow and steady growth in richness, only attaining their previous Palaeozoic richness in the Palaeogene/Neogene from ~60 Ma onwards. A broadly similar pattern is seen in Polyneoptera. Paraneoptera, however, continue their steady increase from the Palaeozoic and show a pronounced increase during the Early Cretaceous (Berriasian–Albian), between ~150 and 100 Ma (Fig 5). This then levels out until they enter a phase of rapid expansion starting in the Palaeogene, from ~65 Ma onwards. The Holometabola enter a more rapid phase of expansion earlier than the Paraneoptera, starting in the Early Jurassic (Sinemurian, from ~193 Ma onwards). They show a pronounced jump in richness at 128 Ma (Barremian), being the largest contributing group to the rapid rise in richness during the Early Cretaceous seen in the NEW RT series. This is followed by a long plateau and then the most rapid expansion phase seen in the entire hexapod fossil record from the lower Eocene (52.2 Ma) onwards (Fig 5).

Both the FR2^+^ and LAB RT series are highly correlated (i.e. strongly co-vary) with NEW RT (Table 1), with all values of Spearman’s rho greater than 0.95 and significant at the 99% confidence limit. This decreases substantially with both first and generalised differencing (Table 1), and correlations between NEW RT and LAB RT lose significance, whilst those between NEW RT and FR2+ retain their significance.

**Table 1:** Spearman rank correlations between richness time series using raw values and after first differencing and generalised differencing.

	LAB RT	LAB BC	FR2^+^	FR2^–^	LAB $\hat{p}$	LAB $\hat{q}$
**Raw values**						
NEW RT	.976**		.956**			
NEW BC		.982**		.979**		
NEW $\hat{p}$					.277	
NEW $\hat{q}$						.559**
**First difference**						
NEW RT	.183		.367*			
NEW BC		.331*		.135		
NEW $\hat{p}$					-.070	
NEW $\hat{q}$						-.028
**Generalized difference**						
NEW RT	.241		.442**			
NEW BC		.273		.111		
NEW $\hat{p}$					.191	
NEW $\hat{q}$						.375*

NEW = new fossil hexapod family richness data presented here, LAB = insect family richness data from Labandeira [11]

FR2 = hexapod family richness data from Ross and Jarzembowski [14], RT = range through, BC = boundary crossers

^+^ = maximum assumption of richness and

^−^ = minimum assumption of richness for FR2

$\hat{p}$ = *per capita* origination rate and $\hat{q}$ = *per capita* extinction rate. Significance assessed using bootstrapping.

* = significant at 95% confidence limit

** = significant at 99% confidence limit.

### Calculated origination and extinction rates

**Fig 6:**
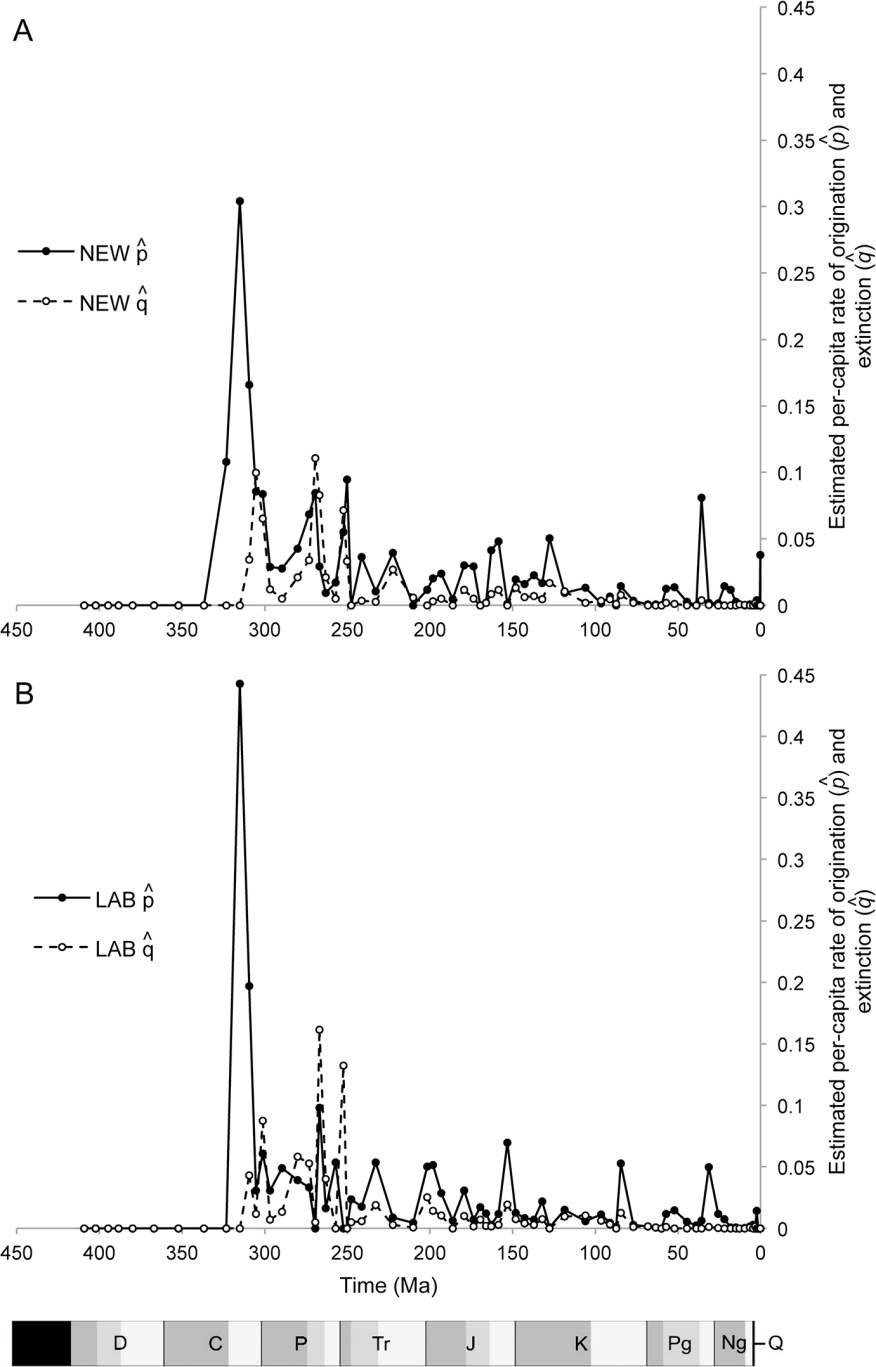
Estimated per-capita rates of origination $\hat{p}$ and extinction $\hat{q}$. Rates are from (A) NEW insect family data and (B) Labandeira [11].

First and last occurrences occur episodically throughout the fossil record of insects (Fig 4B), with an apparent synchrony between origination and extinction through time with origination outstripping extinction. The modal origination occurs in the Palaeogene with large peaks in the Triassic, Late Jurassic and Early Cretaceous. Modal extinction occurs in the Early Cretaceous with large peaks in the late Carboniferous, Permian, Triassic, later Jurassic and Early Cretaceous. Per capita rates of origination and extinction ($\hat{p}$ and $\hat{q}$, respectively; Fig 6), however, show distinctly different profiles in the Palaeozoic and post-Palaeozoic (boundary at 251 Ma) in both NEW and LAB data. Greater variance is seen in the Palaeozoic for both rates in both datasets as well as the highest values reached in each. As for raw counts, per capita origination rates stay robustly higher than extinction from the Triassic onwards and both show long term declines towards the present. There are some notable differences between NEW and LAB: the timing and size of Permian origination peaks differs; there is no Late Cretaceous origination peak; the Carboniferous extinction peak is more pronounced, and those in the Permian less pronounced, not exceeding originations by much. As a result, Spearman rank correlations of these rates between NEW and LAB show no significant relationship in origination rates, while the extinction rates are positively correlated in the raw and generalised differenced time series but retain no relationship after first differencing (Table 1). In general, origination rates seem to more consistently exceed extinction rates.

## Discussion

### Changes in the data

The robustness of described richness through time in the insects, to new discoveries over fifteen years (eighteen years from FR2 data up to end 1991), was tested by compiling a new dataset of fossil hexapod family-richness from literature published up to the end of 2009. Only ten percent of families in the new data remain unchanged over that time, with about 60% of families having different stratigraphic ranges, and 30% of families being completely new to the fossil record. For scientists interested in the details of individual fossil families, for example for dating phylogenies above family level (e.g. [35,36]), the current dataset represents a substantial improvement over previous datasets available. The implication is that the previous fossil insect datasets now have largely historical interest only and should not be used for future macroevolutionary research. Studies based on them ideally require re-assessment. For example, in an analysis based on Labandeira’s [11] data, Yang [37] suggested that differences in origination rates account for the higher diversity of Holometabola compared to Paraneoptera. However, Nicholson et al. [38], using the present data set, found the converse: origination rates between these groups is not significantly different, but extinction rates are significantly reduced in Holometabola compared to Paraneoptera.

While the change in ranges from FR2 in the NEW data (Fig 2A) can be attributed largely to improvement in the stratigraphic resolution of family ranges to stages, the differences from LAB (Fig 2B) require more subtle explanation. Extensions of known ranges in fossil families are to be expected, with continued exploration of fossil sites and descriptions of new finds likely to turn up new first or last occurrences, such as the high rate of discovery in Mesozoic deposits of China (e.g. see [39]). The high proportion of range contractions (25%) seems at first unexpected but can be ascribed to differences in the dates for fossil deposits used (e.g. the Karabastau Formation, Kazakhstan: Kimmeridgian in LAB but Oxfordian in NEW) and extensive changes in taxonomy reducing the number of fossils included in some families, such as in a recent review of termites by Engel *et al*. [40] wherein several fossil taxa, previously attributed to extant families, were reassigned, thus contracting the known range of some families and removing the Hodotermitidae from the fossil record altogether.

The rate of discovery of new fossil hexapods seems disproportionately concentrated in the Cretaceous, with high numbers of publications on the extensive Jiulongshan and Yixian formations in China [39], continued interest in the Crato Formation in Brazil [41], a new supply of Burmese amber [18,42] and abundant new amber deposits in France [43] and Spain [44], although new material continues to be found across almost the entire temporal range of hexapods [6,16,45]. There are an estimated 1067 extant hexapod families (data compiled from the relevant sections of [46]), implying that ~370 extant families (35%) are not yet known from the fossil record and could in principle be found in future. This sets a broad potential upper limit to the height of the richness curve, indicating substantial, but not excessive, potential for future discovery at the family level. The majority of these (196 families) are from the Holometabola. However, in terms of proportion of extant families represented in the fossil record, Holometabola have the most coverage with ~69% represented, followed closely by Polyneoptera (65%), Paraneoptera (64%) and Palaeoptera (58%). Only 33% of extant Apterygota families have a fossil record, perhaps a result of their small size, habitat requirements, and lack of wings [6].

Other informative ways of assessing the potential for future discovery, beyond the scope of the present study, would be to construct taxon vs. specimen accumulation curves to observe if the number of taxa described through time has asymptoted (e.g. [47–49]), or by quantifying the gaps in the record implied by phylogenies (e.g. [47,50,51]). Although some data pertinent to the former (dates of description of extinct families) are present in the current data, one would additionally need to compile the date at which extant families were first described from the fossil record, which is not normally the date of the taxon’s first description.

### Changes in the richness series

Despite major changes to the ranges of insect families over fifteen years of discovery, changes to the pattern of described richness through time derived from those data seem less extensive. Correlations between the time series of the new and previous datasets show that the broad pattern of rise in discovered taxa through time is very similar to that previously described. The generally steady rise in richness through time suggests support for the previous conclusion [12] that no strong logistic limits to family richness have yet been met. However, some of the Cenozoic rise may be attributable to the Pull-of-the-Recent [52] whereby the ranges of extant taxa are pulled forward, accentuating the richness rise nearer the present. Sampling may also have been strongly affected by the abundance of suitable deposits, such as Baltic amber and compression deposits such as the Green River and Florissant formations, which coincide with the Eocene rise [53]. These issues will be examined in future papers.

Other important features preserved in the NEW richness series include evidence for a mass extinction at the end-Permian. The Permian drop in richness is however less abrupt than in FR2. This effect is probably due to the improved temporal resolution from epoch to stage, which pulls the ranges of taxa in FR2 forward to the end of the Permian. At stage level resolution, many of these families are instead seen to have last occurrences before the end Permian. In turn, the asynchronicity in extinction may be genuine, but probably is partly an artefact of an incomplete record (the Signor-Lipps effect [54]) which tends to drag extinctions backwards in time. The major turnover in dominant taxa (Fig 5) accompanying the Permian to Triassic interval is strongly reminiscent of the end-Permian extinction in many other taxa (e.g. [55]). In the hexapod case there was a replacement of the Palaeozoic fauna of mainly Palaeoptera and Polyneoptera by a fauna dominated by Paraneoptera and Holometabola, which appear to have suffered little reduction in their richness [13,53]. Studies on the coherence of these different faunas would be useful (see [56]).

Despite the evidence for an end-Permian extinction, the NEW richness data leave no evidence of an end-Cretaceous extinction, in common with previous data [15,53]. Given the known widespread ecosystem impacts of this event, it is difficult to imagine that insects were completely unaffected but extinction may have occurred below the family level. Some genus-level data provide some support for this [13], as do some studies of trophic interactions [57], but others suggest a weaker extinction in insects than in other taxa [58].

Although all datasets show an increase in richness in the Triassic, a subsequent drop is suggested by the NEW RT series (Fig 4A). Many non-insect taxa apparently experienced a mass extinction at the end-Triassic [59,60] but there has never been good evidence for this in insects. However, the drop is lost in the NEW BC series (Fig 3A), indicating that it is due primarily to abundance of single interval taxa and hence may be an artefact of sampling bias. Indeed the total number of extinctions detected at the end-Triassic boundary is close to zero, indicating that it would be premature to suggest an insect extinction then (Fig 4B).

Surprisingly, the overall level of richness in the NEW data is not always higher than the older data. This is mostly the case in the Palaeozoic, where there was an historical tendency by early workers such as Handlirsch and Tillyard to oversplit taxa, while revisions have decreased the number of valid families. Additionally, and perhaps more importantly, of the 324 families in the new data with ranges in the Palaeozoic, 28% of them represent contractions with respect to LAB. This suggests a specific effect of taxonomy on apparent richness that may be important for other researchers.

The correlations between the differenced time series for the new and old data, although positive, are much less strong than for the raw time series, suggesting moderate differences in the shorter term variation in richness from stage to stage. This is potentially important when assessing the drivers behind diversity change, as time series are generally detrended to remove spurious correlations, and it is the short term variation around the long term trends that are analysed (e.g. [5,61]). The Palaeozoic contains much of the discordance between the series (Fig 4A), with FR2 and NEW having very different shapes while the richness peaks of LAB and NEW are offset from each other. Declines seen in both LAB and FR2 during the Early–mid-Cretaceous (~120–85 Ma) are not shared by NEW, which shows more of a plateau. This plateau could simply be a result of the relative paucity in insect-preserving localities in the Upper Cretaceous, however in an analysis of plant-associated insect families Labandeira [62] found that this plateau coincides with a period of transition from gymnosperm-associated families to angiosperm-associated families, with extinctions in the former approximately matching the level of originations in the latter. Future analyses of occurrence-based data, subsampled to remove the effects sampling bias, will help to elucidate the relative importance of these alternative (but not mutually exclusive) explanations.

### Patterns of origination and extinction

Labandeira [53] picks out five major periods of originations in the insects and four major extinctions. Of the originations, all are still found in the NEW origination series (Fig 4), namely in order, the Late Carboniferous (Bashkirian–Moscovian–first appearance of winged insects and colonization of forested ecosystems); Early Permian (peaking in the Kungurian–colonization of wider environments and the rise of Paraneoptera and Holometabola); Late Jurassic (Oxfordian–radiation of communities on advanced seed plants); Early Cretaceous (peaking in the Barremian–radiations in decomposer and freshwater systems); and the Eocene (Priabonian–suspected to be a sampling artefact that may represent earlier radiations that are poorly sampled). The main addition to this description in the NEW data is the higher peak in the Triassic, which Labandeira [53] attributes to a rebound from the Permian extinction.

In terms of extinctions, the Late Carboniferous peak is attributed by Labandeira [53] to changes in plant communities and trophic structure. The Permian extinction is high in absolute numbers of extinctions but lower in *per capita* rates (cf. Fig 4 and Fig 6) and is generally attributed to high continentality and hot dry climates on land [63]. In addition, there were substantial extinctions in the Late Jurassic (attributed to competitive turnover during the simultaneous radiation; [53]) and the Early Cretaceous (attributed to competitive turnover of taxa adapting to new environments, including angiosperms; see [13,15]). The NEW series add to this a large peak in extinctions in the Triassic, as seen for originations. As discussed above, this may represent the detection of the more general end-Triassic mass extinction, although it may also be an artefact of sampling bias.

In general the high agreement between the timing of originations and extinctions in NEW and FR2 is consistent with the findings of similar studies on other taxa [8,9], suggesting that the great potential for change in the insect fossil record has not translated into major changes in pattern. Some previous authors [9] have interpreted this as encouragement that incomplete and partially erroneous data can preserve broad generalizations about the history of life [10]. However, recent experiences with alternative ways of compiling the data suggest that other issues with the data can remain important in correctly describing and interpreting them [29,64,65].

In general there is high synchronicity between the origination and extinction series (Fig 4B), which is the pattern expected if one biologically depends on the other. This pattern is also expected if they are both simply artefacts of sampling, hence determined by the availability of insect-bearing deposits. The pattern is not simply due to the abundance of single interval taxa (Fig 4B), suggesting perhaps some biological signal in the data.

Originations mostly exceed extinctions across intervals, explaining the consistent rise in family level diversity through time, as well as high extant richness [4,15,66]. In terms of rates, the decline from the Palaeozoic to Mesozoic and Cenozoic is the most obvious feature, in common with other family and genus level analyses [60,64]. Explanations for this include lineage sorting, density-dependent processes and the fact that higher taxa are disproportionately described for older groups [64]. Some of the peaks are different in height in the NEW data compared to LAB (Fig 6); a result of taxonomic changes and shifts in the dating of deposits. The Late Cretaceous (Santonian: 85 Ma) LAB origination peak is not seen in NEW, probably from range extensions pulling more first occurrences back to Lower Cretaceous deposits.

An important question now is to what extent the updated richness, origination and extinction series reflect geological and sampling biases, and thus what features may remain once such biases are removed. Previous work on the marine invertebrate record suggests that many features of the face-value fossil record can remain preserved after sample-standardization (e.g. [67]), although diversity curves may become flatter due to the elimination of the Pull-of-the-Recent [65]. In addition, short term patterns after detrending may be altered, potentially altering macroevolutionary inference [64,68]. Because hexapods generally require exceptional preservation conditions, it is possible that sampling and other biases are a more prominent influence on the face-value record, although considerable biological signal may be retained. Occurrence data of community samples will likely help solve this. However, the range data here are likely to retain value in the dating of phylogenetic trees, since one can be more confident about the completeness of the family ranges, and they are easy to update.

In summary, a new compilation of the fossil ranges of insect families shows changes in the ranges of a high proportion of families, and significant changes in described short term richness and some origination and extinction patterns, but little change in broad temporal patterns. Representing an additional 15 years of data in a rapidly expanding field compared with previously available compendia [11,14], it is hoped that this new dataset will form the basis for future work on elucidating the evolutionary history of the hexapods.

## Supporting Information

S1 Appendix Fully referenced family-range dataset, including additional references [70–1097]. (PDF)

S1 Dataset Family-range spreadsheet dataset, with counts and rates of originations and extinctions. (XLSX)
