# Supplementary material for: Changes to the Fossil Record of Insects through Fifteen Years of Discovery
Source: PLoS One. 2015 Jul 15;10(7):e0128554. doi: 10.1371/journal.pone.0128554 (PMC4503423; doi:10.1371/journal.pone.0128554)
Supplement: S1 Appendix — (PDF) [file pone.0128554.s001.pdf]

# Changes to the fossil record of insects through fifteen years of discovery: Supplementary Material

The analyses are based on the following range data, compiled from a survey of the literature published up to the end of 2009. This was such a huge task that the deadline had to be imposed otherwise the database would never be finished and the data would not be analysed. At the end of 2011 it was felt that the database was complete enough for the analysis to go ahead, even though there were some obscure papers that had not yet been seen, and there were some question marks over some of the records, such as conflicting opinions, which needed checking. Since the analyses were carried out, obscure papers have been seen, queries have been checked and additional errors have been spotted, however these only affect the ranges of 22 families, while nine additional families have been added and one removed. Given the size of the database these corrections would make a negligible difference to the results of the analyses. These families are listed on the following page with the incorrect ranges as used in the analyses.

Kaddumi (2005) describes new families, genera and species in Jordanian amber, however this work is not peer-reviewed so it is uncertain whether they satisfy the ICZN code, so they are not included. Some may have been named in an earlier edition. Certainly those families with new names that do not use the stem of the type genus are not valid (article 11.7). However, the oldest records of pre-existing families are included where they appear to be reliable.

The following family range data are arranged hierarchically by supraordinal clade. Orders and families are arranged alphabetically within these.

Families with incorrect ranges as used in the analyses, or were not included:

| | |
|---|---|
| Arnoldidae | was not included |
| Cleridae | K1(Albian)-Holocene |
| Coleophoridae | Eoc.(Ypresian)-Holocene |
| Coptoclavidae | T3(Rhaetian)-K1(Aptian) |
| Elachistidae | Eoc.(Priabonian)-Holocene |
| Eriocraniidae | Eoc.(Priabonian)-Holocene |
| Eukulojidae | P2(Roadian) |
| Gallorommatidae | K1(Albian)-Eoc.(Priabonian) |
| Gelechiidae | Eoc.(Ypresian)-Holocene |
| Gracillariidae | K2(Cenomanian)-Holocene |
| Grohnidae | was not included |
| Hemeroscopidae | K1(Barremian)-K1(Aptian) |
| Hesperiidae | Mio.(Aquitanian)-Holocene |
| Kaltanidae | C2(Gzhelian)-K1(Valanginian) |
| Kulojidae | was a synonym of Eukulojidae |
| Kuwaniidae | was not included |
| Laemophloeidae | K1(Albian)-Holocene (no longer included) |
| Lithuanicoccidae | was not included |
| Mecynopteridae | C2(Moscovian) |
| Necrotauliidae | T3(Carnian)-K1(Valanginian) |
| Panfiloviidae | J3(Oxfordian) |
| Panorpodidae | Eoc.(Priabonian)-Holocene |
| Philopotamidae | J1(Toarcian)-Holocene |
| Prohemerobiidae | J1(Toarcian) |
| Pyralidae | Eoc.(Priabonian)-Holocene |
| Saurophthiridae | was a synonym of Chresmodidae |
| Serafinidae | was not included |
| Tetracampidae | K1(Barremian)-Holocene |
| Trisegmentatidae | was not included |
| Weitschatidae | was not included |
| ‘Xenopteridae’ | C2(Bashkirian) |
| Zorotypidae | K1(Albian)-Holocene |

## Epiclass Hexapoda

## Class Entognatha

**O. Collembola** Lubbock, 1871 Devonian(Pragian)-Quaternary(Holocene)

F. Arrhopalitidae K1(Albian)-Holocene

First: *Arrhopalites* sp. in Delclòs et al. (2007), Álava amber, Escucha Formation, Basco-Cantabrian Basin, Álava Province, Spain.

F. Bourletiellidae K1(Albian)-Holocene

First: *Fasciosminthurus* sp. in Delclòs et al. (2007), Álava amber, Escucha Formation, Basco-Cantabrian Basin, Álava Province, Spain.

F. Brachystomellidae K2(Campanian)-Holocene

First: *Bellingeria cornua* Christiansen and Pike, 2002, Canadian amber, Grassy Lake, Alberta, Canada.

F. Entomobryidae P1(Kungurian)-Holocene

First: *Permobrya mirabilis* Riek, 1976, carbonaceous shales, middle Ecca Group, Haakdoornfontein, near Pretoria, South Africa. (This species could belong to the Praentomobryidae Christiansen and Nascimbene, 2006.)

F. Hypogastruridae K2(Campanian)-Holocene

First: Mentioned in Christiansen and Pike (2002), Canadian amber, Grassy Lake, Alberta, Canada.

F. Isotomidae D1(Pragian)-Holocene

First: *Rhyniella praecursor* in Ross and York (2004), Rhynie chert, Aberdeenshire, Scotland, United Kingdom.

F. Neanuridae K1(Albian)-Holocene

First: e.g. *Protodontella minicornis* Christiansen and Nascimbene, 2006, Burmese amber (Burmite), Hukawng Valley, Kachin State, Myanmar.

F. Oncobryidae Christiansen and Pike, 2002 K2(Campanian)

First and Last: *Oncobrya decepta* Christiansen and Pike, 2002, Canadian amber, Medicine Hat, Alberta, Canada.

F. Onychiuridae K1(Albian)-Holocene

First: *Onychiurus* sp. in Delclòs et al. (2007), Álava amber, Escucha Formation, Basco-Cantabrian Basin, Álava Province, Spain.

F. Poduridae K2(Campanian)-Holocene

First: Mentioned in McKellar et al. (2008), Canadian amber, Grassy Lake, Alberta, Canada.

F. Praentomobryidae Christiansen and Nascimbene, 2006(Praentombryidae) K1(Albian)

e.g. *Praentomobrya avita* Christiansen and Nascimbene, 2006, Burmese amber (Burmite), Hukawng Valley, Kachin State, Myanmar.

F. Protentomobryidae K2(Campanian)

e.g. *Protentomobrya walkeri* in McKellar et al. (2008), Canadian amber, Cedar Lake, Manitoba, Canada.

F. Sminthuridae K1(Albian)-Holocene

First: e.g. *Grinnellia ventis* Christiansen and Nascimbene, 2006, Burmese amber (Burmite), Hukawng Valley, Kachin State, Myanmar.

F. Tomoceridae K1(Albian)-Holocene

First: Mentioned in Christiansen and Nascimbene (2006), Burmese amber (Burmite), Hukawng Valley, Kachin State, Myanmar.

**O. Diplura** Börner, 1904 Carboniferous(Moscovian)-Quaternary(Holocene)

F. Campodeidae Eoc.(Priabonian)-Holocene

First: *Campodea darwinii* in Weitschat and Wichard (2002), Baltic amber.

F. Japygidae Mio.(Aquitanian)-Holocene

First: Figured in Poinar (1992), Mexican amber, Simojovel, Chiapas, Mexico. (Wilson and Martill 2001 believe this specimen is a beetle larva.)

F. Procampodeidae Mio.(Burdigalian)-Holocene

First: Figured in Poinar (1992), Dominican amber, Cordillera Septentrional, near Santiago, Dominican Republic.

F. Testajapygidae Kukalová-Peck, 1987 C2(Moscovian)

First and Last: *Testajapyx thomasi* in Wilson and Martill (2001), Carbondale Formation, Mazon Creek, Illinois, United States.

## Class Insecta (= Ectognatha)

**O. Archaeognatha** Börner, 1904 (Machilida, Microcoryphia, Monura)
Carboniferous(Moscovian)-Quaternary(Holocene)

F. Dasyleptidae C2(Moscovian)-P2(Roadian)

First: "*Dasyleptus*" sp. in Engel (2009a), Carbondale Formation, Mazon Creek, Illinois, United States. (Assignment to Dasyleptidae is questionable; see Rasnitsyn 2000a.)

Last: *Dasyleptus brongniarti* in Engel (2009a), Kuznetsk Formation (Mitino Horizon), Kaltan, Kemerovo Region, Russian Federation.

F. Machilidae K1(Albian)-Holocene

First: Mentioned in Rasnitsyn and Ross (2000), Burmese amber (Burmite), Hukawng Valley, Kachin State, Myanmar.

F. Meinertellidae (Meunertellidae) K1(Barremian)-Holocene

First: *Cretaceomachilis libanensis* Sturm and Poinar, 1998, Lebanese amber, unknown horizon, unknown locality, Lebanon.

F. Triassomachilidae T2(Anisian)

First and Last: *Triassomachilis uralensis* in Bitsch and Nel (1999), Bukobay Formation, Bashkortostan, Ural Mountains, Russian Federation. (Sinitshenkova 2000c considered *Triassomachilis* to be a mayfly nymph and synonymised it with *Mesoneta* [Mesonetidae], however Grimaldi and Engel 2005 retain this family in Archaeognatha though suggest it requires re-study.)

### Dicondylia

**O. Zygentoma** Börner, 1904 (Lepismatida, Thysanura *sensu stricto*)
Carboniferous(Moscovian)-Quaternary(Holocene)

F. Carbotripluridae Kluge, 1996 C2(Moscovian)

First and Last: *Carbotriplura kukalovae* Kluge, 1996, Whetstone horizon, Radnice Member, Radnice Basin, Bohemia, Czech Republic. (This nymph was originally designated as the paratype of *Bojophlebia prokopi* [Ephemeroptera: Bojophlebiidae]; see Kluge 1996.)

F. Lepidotrichidae (Lepidothrichidae, Lepidothricidae) K2(Santonian)-Eoc.(Priabonian)
Extant relic *Tricholepidion gertschi* assigned to Tricholepidiidae (Engel, 2006a).

First: Mentioned in Rasnitsyn (2002l), Yantardakh amber, Kheta Formation, Taimyr, Krasnoyarsk Krai, Siberian Federal District, Russian Federation.

Last: *Lepidothrix pilifera* in Engel (2006a), Baltic amber.

F. Lepismatidae K1(Aptian)-Holocene

First: Figured in Staniczek and Bechly (2007), Crato Formation, Araripe Basin, Ceará, Brazil.

F. Nicoletiidae (Ateluridae, Nicolettidae) Mio.(Burdigalian)-Holocene

First: e.g. *Hemitrinemura exstincta* Mendes and Poinar, 2004, Dominican amber, Cordillera Septentrional, near Santiago, Dominican Republic.

## Subclass Pterygota

**Pterygota** *incertae sedis*

F. Apheloneuridae P1(Artinskian)-P1(Kungurian)

First: e.g. *Apheloneura minutissima* in Novokshonov (2000), Wellington Formation, Elmo site, Dickinson County, Kansas, United States.

Last: *Apheloneura uralensis* Novokshonov, 2000, Koshelevka Formation, Tshekarda, Ural Mountains, Russian Federation.

F. Hadentomidae C2(Moscovian)-C2(Kasimovian)
*Palaeocixius* and *Protoblattina* were removed from Hadentomidae by Béthoux et al. (2005). *Hadentomum* is considered Pterygota *incertae sedis* by Rasnitsyn (2002a).

First: *Hadentomum americanum* in Carpenter (1992b), Carbondale Formation, Mazon Creek, Illinois, United States.

Last: e.g. *Fayoliella elongata* in Carpenter (1992b), Upper Coal Measures, Commentry, Allier, France.

F. Hebeigrammidae Hong, 2003(Mesogrammatidae) K1(Valanginian)
Originally described in the Caloneurodea, this family was considered by Ross and Jarzembowski (1993) and Labandeira (1994) as Orthoptera and by Rasnitsyn (2002d) as Pterygota *incertae sedis*, which is followed here.

First and Last: *Hebeigramma divaricata* in Hong (2003), greyish-black shale, Qingquang village, Weichang County, Hebei Province, China.

F. Herbstialidae C2(Bashkirian)
Rasnitsyn (2002a) considers *Herbstiala* to be Pterygota *incertae sedis*.

First and Last: *Herbstiala herbsti* in Brauckmann and Hahn (1980), seam 16 West, Sophia Jacoba coliery, Heinsberg, North Rhine-Westphalia, Germany.

F. Homoeodictyidae (Homeodictyidae) P2(Wordian)
Rasnitsyn (2002a) considers this family to be Pterygota *incertae sedis*.

First and Last: *Homoeodictyon elongatum* in Rasnitsyn (2002a), Amanak Formation, Kargala, Belozersky District, Orenburg Region, Russian Federation.

F. Montanuraliidae Novokshonov, 1998a P1(Kungurian)

First and Last: *Montanuralia aeria* Novokshonov, 1998a, Koshelevka Formation, Tshekarda, Ural Mountains, Russian Federation.

F. Permetatoridae Novokshonov, 1999 P1(Kungurian)

First and Last: *Permetator semitritus* Novokshonov, 1999, Koshelevka Formation, Tshekarda, Ural Mountains, Russian Federation.

F. Permoneuridae P1(Artinskian)
Beckemeyer (2000) and Sinitshenkova (2002a) both place this family in Pterygota *incertae sedis*.

First and Last: *Permoneura lameerei* in Beckemeyer (2000), Wellington Formation, Elmo site, Dickinson County, Kansas, United States.

F. Rectineuridae C2(Moscovian)
Sinitshenkova (2002a) places this family in Pterygota *incertae sedis*.

First and Last: *Rectineura lineata* in Carpenter (1992b), Yorkian Series, Chislet Colliery, Sturry, Kent, United Kingdom.

F. Stygneidae (Stygnidae) C2(Bashkirian)
Rasnitsyn (2002a) considers this family to be Pterygota *incertae sedis*. The name Stygnidae Handlirsch, 1906 is a junior homonym pre-occupied by the extant Opiliones family Stygnidae Simon, 1879, so the alternative spelling is used here.

First and Last: *Stygne roemeri* in Rasnitsyn (2002a), Alfred Mine, Alfred Mine, Upper Silesian Basin, Poland.

F. Sypharopteridae C2(Moscovian)
Rasnitsyn (2002d) included this family in Caloneurodea but this placement was rejected by Béthoux et al. (2004c).

First and Last: *Sypharoptera pneuma* in White (1995), Carbondale Formation, Mazon Creek, Illinois, United States.

F. Vogesonymphidae Sinitshenkova & Papier *in* Sinitshenkova et al., 2005 T2(Anisian)

First and Last: *Vogesonympha ludovici* Sinitshenkova & Papier *in* Sinitshenkova et al., 2005, Grès à Voltzia, Bas-Rhin/Moselle, Northern Vosges Mountains, France.

**O. Ephemeroptera** Hyatt and Arms, 1890 (Ephemerida, Ephemeridea, Syntonopterida, Syntonopterodea) Carboniferous(Moscovian)-Quaternary(Holocene)

*Mesogenesia* from the Uda Formation (Oxfordian, Buryatia) is considered unplaced in Ephemeroptera, leaving Palingeniidae without a fossil record (McCafferty, 1990, 2004; Kluge, 2004). *Myanmarella rossi* Sinitshenkova, 2000a is Ephemeroptera *incertae sedis*, leaving Prosopistomatidae without a fossil record (Kluge, 2004).

F. Acanthametropodidae (Analetrididae) Eoc.(Priabonian)-Holocene

First: *Analetris secundus* Godunko and Kłonowska-Olejnik, 2006, Baltic amber.

F. Aenigmephemeridae (Aenigmephemerdae) J3(Oxfordian)

First and Last: *Aenigmephemera demoulini* in Hubbard (1987), Karabastau Formation, Karatau Range, Tien Shan mountains, Kazakhstan.

F. Ameletidae McCafferty, 1991 Eoc.(Priabonian)-Holocene
Previously in Siphlonuridae.

First: e.g. *Baltameletus oligocaenicus* in Godunko et al. (2008), Baltic amber.

F. Ameletopsidae Eoc.(Priabonian)-Holocene

First: *Balticophlebia hennigi* in Wichard et al. (2009), Baltic amber.

F. Ametropodidae K2(Turonian)-Holocene

First: *Palaeometropus cassus* Sinitshenkova, 2000b, New Jersey amber, South Amboy Fire Clay (Raritan Formation), New Jersey, United States.

F. Arthropleidae Eoc.(Priabonian)-Holocene

First: *Electrogenia dewalschei* in Wichard et al. (2009), Baltic amber. (Kluge 2004 considers this species as family *incertae sedis*.)

F. Australiphemeridae McCafferty, 1991(Palaeoanthidae, Paleoanthidae) K1(Aptian)-K2(Santonian)

First: e.g. *Australiphemera revelata* in McCafferty and Santiago-Blay (2009), Crato Formation, Araripe Basin, Ceará, Brazil.

Last: e.g. *Palaeoanthus orthostylus* Kluge, 1994, Yantardakh amber, Kheta Formation, Taimyr, Krasnoyarsk Krai, Siberian Federal District, Russian Federation. (Originally described in Palaeoanthidae, McCafferty 1997 placed the genus in Australiphemeridae. While this attribution is not certain [see Kluge et al. 2006], it is followed in McCafferty and Santiago-Blay 2009 and here.)

F. Babidae Kluge et al., 2006 Eoc.(Priabonian)

First and Last: *Baba lapidea* Kluge et al., 2006, Baltic amber.

F. Baetidae K1(Barremian)-Holocene

First: Mentioned in McCafferty (1997), Lebanese amber, unknown horizon, unknown locality, Lebanon.

F. Baetiscidae K1(Aptian)-Holocene
*Caririephemera marquesi* Zamboni, 2001 shows no characters which identify it as an ephemeropteran (Staniczek, 2007). An unnamed specimen from the Lower Cretaceous of Australia shows affinities to Baetiscidae but has not been formally placed as such (Pescador et al., 2009).

First: *Protobaetisca bechlyi* Staniczek, 2007, Crato Formation, Araripe Basin, Ceará, Brazil.

F. Bojophlebiidae Kukalová-Peck, 1985 C2(Moscovian)

First and Last: *Bojophlebia prokopi* in Wootton and Kukalová-Peck (2000), Whetstone horizon, Radnice Member, Radnice Basin, Bohemia, Czech Republic.

F. Cretomitarcyidae Sinitshenkova, 2000b K2(Turonian)
Family status given in McCafferty (2004), however Staniczek (2007) considers it should belong in stemline of Baetiscidae and sees no reason for a separate family. McCafferty and Santiago-Blay (2009) retain it as a separate family.

First and Last: *Cretomitarcys luzzi* Sinitshenkova, 2000b, New Jersey amber, South Amboy Fire Clay (Raritan Formation), New Jersey, United States.

F. Epeoromimidae (Epeoromididae) J1(Pliensbachian)-K1(Berriasian)

First: *Epeoromimus kazlauskasi* in Sinitshenkova (2003), Osinovskiy Formation, Chernyi Etap, Kemerovo Region, Russian Federation. (May also occur in the Abashevo Formation.)

Last: e.g. *Epeoromimus* sp. in Sinitshenkova (2002d), Tsagan-Tsab, Khutel-Kara, Dornogovi (East Gobi) Aimag, Mongolia.

F. Ephemerellidae Eoc.(Priabonian)-Holocene
*Clephemera clava* and *Turfanerella tingi* should be considered Ephemeroptera *incertae sedis* (see Zhang and Kluge, 2007; Jacobus and McCafferty, 2008).

First: *Timpanoga viscata* in Weitschat and Wichard (2002), Baltic amber.

F. Ephemeridae K1(Aptian)-Holocene
Staniczek (2007) erroneously lists the australiphemerid genera *Australiphemera* and *Microphemera* in this family, without comment, while Huang et al. (2007b) list them in both Ephemeridae and Australiphemeridae, as well as listing *Ephemera* from the Jurassic Solnhofen Limestone where they probably meant *Mesephemera* of Mesephemeridae, a common mayfly in that deposit (Kluge and Sinitshenkova, 2002).

First: *Cratonympha microcelata* in Staniczek (2007), Crato Formation, Araripe Basin, Ceará, Brazil. (Staniczek 2007 considers the validity and status of this species doubtful.)

F. Euthyplociidae (Eutyplocidae, Pristiplociidae) K1(Barremian)-Holocene

First: Mentioned in Peñalver et al. (1999), Montsec lithographic limestones, Montsec Range, Lleida Province, Spain.

F. Fuyoidae Zhang and Kluge, 2007(Fujoidae) J2(Callovian)

First and Last: *Fuyous gregarius* Zhang and Kluge, 2007, Jiulongshan Formation, near Daohugou, Ningcheng county, Inner Mongolia, China. (This species was misidentified as *Mesoneta antiqua* in Ren et al. 2002.)

F. Heptageniidae (Ecdyonuridae, Ecdyuridae) K2(Turonian)-Holocene

First: *Amerogenia macrops* Sinitshenkova, 2000b, New Jersey amber, South Amboy Fire Clay (Raritan Formation), New Jersey, United States.

F. Hexagenitidae (Paedephemeridae, Stenodicranidae) J2(Callovian)-K1(Aptian)
Placement of *Siberiogenites* spp. in this family is ungrounded (see Zhang and Kluge, 2007).

First: *Shantous lacustris* Zhang and Kluge, 2007, Jiulongshan Formation, near Daohugou, Ningcheng county, Inner Mongolia, China. (This species was misidentified as *Mesobaetis sibirica* in Ren et al. 2002.)

Last: e.g. *Cratohexagenites longicercus* Staniczek, 2007, Crato Formation, Araripe Basin, Ceará, Brazil. (Huang et al. 2007b erroneously list *Protoligoneuria* (Crato Formation) as from the Baltic amber and date it as Upper Cretaceous.)

F. Isonychiidae Eoc.(Priabonian)-Holocene
Previously placed within Siphlonuridae (e.g. Carpenter, 1992b; Hubbard, 1987) or Oligoneuriidae (Ross and Jarzembowski, 1993), Isonychiidae is now considered a family (Ogden et al., 2009).

First: *Isonychia alderensis* Lewis, 1977, Passamari Formation, Ruby River Basin, Montana, United States.

F. Jarmilidae P1(Sakmarian)
Kluge (2004) appears to consider this a junior synonym of Protereismatidae but Grimaldi and Engel (2005) and Huang et al. (2007b) retain it as a separate family.

First and Last: *Jarmila elongata* in Hubbard (1987), Obora locality, Bačov Beds, Letovice Formation, Moravia, Czech Republic.

F. Leptophlebiidae (Leptophlebidae) K1(Barremian)-Holocene

First: e.g. *Conovirilus poinari* in Godunko and Krzemiński (2009), Lebanese amber, unknown horizon, unknown locality, Lebanon.

F. Litophlebiidae (Lithophlebiidae, Xenophlebiidae) T3(Carnian)

First and Last: *Litophlebia optata* in Huang et al. (2007b), Molteno Formation, KwaZulu-Natal, Karoo Basin, South Africa.

F. Mesephemeridae (Palingeniopsidae) P2(Roadian)-J3(Tithonian)

First: *Palingeniopsis praecox* in Hubbard (1987), Iva-Gora limestones, Soyana River, Arkhangelsk Region, Ural Mountains, Russian Federation.

Last: e.g. *Mesephemera lithophila* in Hubbard (1987), Solenhofen Lithographic Limestone, Solenhofen/Eichstadt, Bavaria, Germany.

F. Mesonetidae T2(Anisian)-J3(Tithonian)

First: e.g. *Mesoneta minuta* Sinitshenkova, 2000c, Varengayakha Formation, Urengoi District, Tyumen' Region, Russian Federation.

Last: e.g. *Furvoneta lucida* Sinitshenkova, 2002d, Shar-Teg Formation, Shar-Teg Ula, Gobi-Altai Aimag, Mongolia.

F. Mesoplectopteridae T2(Anisian)
An undescribed specimen from the Permian of Germany assigned to this family is more likely a protereismatid (Kluge and Sinitshenkova, 2002).

First and Last: *Mesoplectopteron longipes* in Sinitshenkova et al. (2005), Grès à Voltzia, Bas-Rhin/Moselle, Northern Vosges Mountains, France.

F. Metretopodidae (Metretopdidae) Eoc.(Priabonian)-Holocene

First: e.g. *Siphloplecton jaegeri* in Godunko and Neumann (2006), Baltic amber.

F. Miracopteridae Novokshonov, 1994b P1(Sakmarian)-P1(Kungurian)

First: Figured in Novokshonov and Aristov (2002), Obora locality, Bačov Beds, Letovice Formation, Moravia, Czech Republic.

Last: *Miracopteron mirabile* in Rasnitsyn (2002b), Koshelevka Formation, Tshekarda, Ural Mountains, Russian Federation.

F. Misthodotidae (Eudoteridae, Mistodothidae) P1(Asselian)-T2(Anisian)

First: *Misthodotes stapfi* Kinzelbach and Lutz, 1984, Jeckenbach layers, Niedermoschel, Donnersbergkreis district, Rhineland-Palatinate, Germany.

Last: *Triassodotes vogesiacus* Sinitshenkova & Papier *in* Sinitshenkova et al., 2005, Grès à Voltzia, Bas-Rhin/Moselle, Northern Vosges Mountains, France.

F. Neoephemeridae Eoc.(Ypresian)-Holocene

First: *Neoephemera antiqua* Sinitshenkova, 1999, Klondike Mountain Formation, Okanagan Highlands, Washington, United States.

F. Oboriphlebiidae P1(Sakmarian)
Kluge (2004) appears to consider this a junior synonym of Protereismatidae but Grimaldi and Engel (2005) and Huang et al. (2007b) retain it as a separate family.

e.g. *Oboriphlebia moravica* in Hubbard (1987), Obora locality, Bačov Beds, Letovice Formation, Moravia, Czech Republic.

F. Oligoneuriidae (Oligoneuridae) K1(Aptian)-Holocene

First: e.g. *Colocrus*? *magnum* Staniczek, 2007, Crato Formation, Araripe Basin, Ceará, Brazil.

F. Philolimniidae Jacobus and McCafferty, 2006 Eoc.(Ypresian)
Previously in Ephemerellidae.

First and Last: *Philolimnias sinica* in Jacobus and McCafferty (2006), Fushun amber, Guchengzi, Liaoning Province, China.

F. Polymitarcidae (Polymitarcyidae) K1(Barremian)-Holocene

First: *Mesopalingea leridae* in Peñalver et al. (1999), Montsec lithographic limestones, Montsec Range, Lleida Province, Spain. (Originally described by Whalley and Jarzembowski 1985 in Palingeniidae, this species is listed in Potamanthidae by Peñalver et al. 1999 but is provisionally placed in Polymitarcidae by McCafferty 2004.)

F. Potamanthidae (Pothamanthidae, Pothamantidae) K1(Aptian)-Holocene
McCafferty (2004) lists no fossil specimens in this family.

First: *Olindinella gracilis* in Staniczek (2007), Crato Formation, Araripe Basin, Ceará, Brazil. (Staniczek 2007 considers the status and validity of this species doubtful.)

F. Protereismatidae (Proteismatidae) C2(Gzhelian)-P2(Wordian)

First: Mentioned in Rowland (1997), Bursum Formation (Red Tanks Member), Carrizo Arroyo, New Mexico, United States.

Last: e.g. *Phthartus rossicus* in Hubbard (1987), Amanak Formation, Kargala, Belozersky District, Orenburg Region, Russian Federation.

F. Sharephemeridae Sinitshenkova, 2002d J3(Tithonian)

First and Last: *Sharephemera cubitalis* Sinitshenkova, 2002d, Shar-Teg Formation, Shar-Teg Ula, Gobi-Altai Aimag, Mongolia.

F. Siphlonuridae (Aphelophlebodidae) T2(Anisian)-Holocene

First: e.g. *Triassonurus doliiformis* Sinitshenkova & Papier *in* Sinitshenkova et al., 2005, Grès à Voltzia, Bas-Rhin/Moselle, Northern Vosges Mountains, France.

F. Siphluriscidae Zhou and Peters, 2003 J2(Aalenian)-Holocene

First: *Stackelbergisca shaburensis* in Zhang (2006b), Ichetuy Formation, Novospasskoye, Mukhorshibirsky District, Buryatia, Russian Federation. (Zhang and Kluge 2007 place *Stackelbergisca* in Anteritorna *incertae sedis* but Lin and Huang 2008 retain it in Siphluriscidae.)

F. Syntonopteridae (Synonopteridae) C2(Moscovian)-P2(Capitanian)

First: e.g. *Lithoneura lameerei* in Garrouste et al. (2009), Carbondale Formation, Mazon Creek, Illinois, United States.

Last: *Gallolithoneura butchlii* Garrouste et al., 2009, Pradineaux Formation, Petit Coulet Redon Hill, Bas-Argens Basin, Provence, France.

F. Tintorinidae Krzemiński and Lombardo, 2001 T2(Ladinian)

First and Last: *Tintorina meridensis* Krzemiński and Lombardo, 2001, Upper Meride Limestone, Val Mara, Canton Ticino, Switzerland.

F. Torephemeridae Sinitshenkova, 1989 T2(Anisian)-K1(Berriasian)

First: *Archaeobehningia mogutshevae* Sinitshenkova, 2000c, Varengayakha Formation, Urengoi District, Tyumen' Region, Russian Federation. (Kluge 2004 considers *Archaeobehningia* a junior synonym of *Mesogenesia* but Huang et al. 2007b retain it as a separate genus in Torephemeridae.)

Last: *Torephemera longipes* Sinitshenkova, 1989, Tsagan-Tsab, Khutel-Kara, Dornogovi (East Gobi) Aimag, Mongolia.

F. Toxodotidae Sinitshenkova & Papier *in* Sinitshenkova et al., 2005 T2(Anisian)

First and Last: *Taxodotes coloratus* Sinitshenkova & Papier *in* Sinitshenkova et al., 2005, Grès à Voltzia, Bas-Rhin/Moselle, Northern Vosges Mountains, France.

F. Triassoephemeridae Sinitshenkova & Papier *in* Sinitshenkova et al., 2005 T2(Anisian)

First and Last: *Triassoephemera punctata* Sinitshenkova & Papier *in* Sinitshenkova et al., 2005, Grès à Voltzia, Bas-Rhin/Moselle, Northern Vosges Mountains, France.

F. Triassomanthidae Sinitshenkova & Papier *in* Sinitshenkova et al., 2005 T2(Anisian)

First and Last: *Triassomanthus parvulus* Sinitshenkova & Papier *in* Sinitshenkova et al., 2005, Grès à Voltzia, Bas-Rhin/Moselle, Northern Vosges Mountains, France.

F. Voltziaephemeridae Sinitshenkova & Papier *in* Sinitshenkova et al., 2005 T2(Anisian)

First and Last: *Voltziaephemera fossoria* Sinitshenkova & Papier *in* Sinitshenkova et al., 2005, Grès à Voltzia, Bas-Rhin/Moselle, Northern Vosges Mountains, France.

## Metapterygota

## Palaeodictyopterida

**O. Diaphanopterodea** Handlirsch, 1919 (Diaphanopterida, Diaphanopteroidea, Palaeohymenoptera) Carboniferous(Moscovian)-Permian(Wuchiapingian)

F. Asthenohymenidae (Astenohymenidae, Doteridae) C2(Gzhelian)-P3(Wuchiapingian)

First: e.g. *Asthenohymen zonatus* Sinitshenkova *in* Rasnitsyn et al., 2004a, Bursum Formation (Red Tanks Member), Carrizo Arroyo, New Mexico, United States.

Last: e.g. *Karoohymen minutus* van Dijk and Geertsema, 1999, Normandien (Estcourt) Formation, Beaufort Group, KwaZulu-Natal, Karoo Basin, South Africa. (Although they acknowledge that Carpenter 1992b synonymised *Karoohymen* under *Asthenohymen*, thus removing it from Scytohymenidae and Megasecoptera, van Dijk and Geertsema 1999 describe this species under *Karoohymen* without any explanation for disagreeing with Carpenter 1992b. Later authors [Shcherbakov et al. e.g. 2009] follow Carpenter's arrangement, however if a new combination was created for this species it would be a junior homonym of *Asthenohymen minutus* Zimmerman, 1962 Tasch and Zimmerman in 1962.)

F. Biarmohymenidae P1(Artinskian)-P1(Kungurian)

First: *Anomalohymen dochmus* Beckemeyer and Engel, 2009, Wellington Formation, Midco, Oklahoma, United States.

Last: *Biarmohymen bardense* in Beckemeyer and Engel (2009), Koshelevka Formation, Tshekarda, Ural Mountains, Russian Federation.

F. Diaphanopteridae (Diaphanopteritidae) C2(Kasimovian)
*Philiasptilon* and *Diaphterum* are excluded from this family by Béthoux and Nel (2003b).

e.g. *Diaphanoptera munieri* in Béthoux and Nel (2003b), Upper Coal Measures, Commentry, Allier, France.

F. Elmoidae P1(Sakmarian)-P1(Artinskian)

First: e.g. *Elmodiapha ovata* in Zajíc and Štamberg (2004), Obora locality, Bačov Beds, Letovice Formation, Moravia, Czech Republic. (Béthoux and Nel (2003b) call for revision of these taxa with recognition of tectonic deformation.)

Last: *Elmoa trisecta* in Beckemeyer and Engel (2009), Wellington Formation, Elmo site, Dickinson County, Kansas, United States.

F. Kaltanelmoidae P2(Roadian)
Carpenter (1963b) doubted this family's affinities with Diaphanopterodea.

First and Last: *Kaltanelmoa sibirica* in Rohdendorf (1991), Kuznetsk Formation (Mitino Horizon), Kaltan, Kemerovo Region, Russian Federation.

F. Kulojidae P2(Roadian)
Regarded as separate from Eukulojidae by Sinitshenkova (1981b) and listed under Diaphanopterodea by Labandeira (1994).

First and Last: *Kuloja expansa* in Carpenter (1992b), Iva-Gora limestones, Soyana River, Arkhangelsk Region, Ural Mountains, Russian Federation.

F. Martynoviidae C2(Gzhelian)-P2(Wordian)

First: *Phaneroneura rineharti* Sinitshenkova *in* Rasnitsyn et al., 2004a, Bursum Formation (Red Tanks Member), Carrizo Arroyo, New Mexico, United States.

Last: e.g. *Salagouneura chimaira* Béthoux et al., 2003c, Salagou Formation (Mérifons Member), Lodève Basin, Hérault, France.

F. Parabrodiidae C2(Moscovian)-C2(Kasimovian)

First: *Piesbergala leipnerae* Brauckmann and Herd, 2003, Osnabrück Formation, Piesberg quarry, Lower Saxony, Germany.

Last: *Parabrodia carbonaria* in Brauckmann and Herd (2003), Stanton Limestone, Garnett, Anderson County, Kansas, United States.

F. Parelmoidae P1(Artinskian)-P1(Kungurian)

First: e.g. *Parelmoa obtusa* in Beckemeyer and Engel (2009), Wellington Formation, Midco, Oklahoma, United States. (Listed in Beckemeyer and Engel 2009 under Elmoidae in error (R.J. Beckemeyer pers. comm. 2009).)

Last: e.g. *Permuralia maculata* in Sinitshenkova (2002a), Koshelevka Formation, Tshekarda, Ural Mountains, Russian Federation. (Formerly *Uralia maculata*, nomen nudum.)

F. Paruraliidae Kukalová-Peck and Sinitshenkova, 1992 P1(Kungurian)

e.g. *Paruralia rohdendorfi* in Sinitshenkova (2002a), Koshelevka Formation, Tshekarda, Ural Mountains, Russian Federation.

F. Prochoropteridae C2(Moscovian)-C2(Kasimovian)

First: *Prochoroptera calopteryx* in Kukalová-Peck and Brauckmann (1990), Carbondale Formation, Mazon Creek, Illinois, United States.

Last: *Euchoroptera longipennis* in Carpenter (1997), Stanton Limestone, Garnett, Anderson County, Kansas, United States.

F. Rhaphidiopsidae (Raphidiopseidae) C2(Kasimovian)
Sinitshenkova (2002a) considers this family to belong in the Megasecoptera.

First and Last: *Rhaphidiopsis diversipenna* in Brauckmann and Herd (2003), Rhode Island Formation, Narragansett basin, Rhode Island, United States.

F. Triplosobidae C2(Kasimovian)

First and Last: *Triplosoba pulchella* in Prokop and Nel (2009), Upper Coal Measures, Commentry, Allier, France. (Prokop and Nel (2009) show that this fossil is closely related to the Diaphanopterodea but do not make a formal attribution to the order, preferring instead leave it unplaced within the Palaeodictyopterida.)

**O. Dicliptera** Grimaldi and Engel, 2005 (Archodonata, Permothemistida)
Permian(Artinskian)-Permian(Roadian)

The family Ogassidae, mentioned in Sinitshenkova (2002a) as in press, appears never to have been published and so is not included here.

F. Diathemidae P1(Kungurian)

e.g. *Diathema tenerum* in Sinitshenkova (2002a), Koshelevka Formation, Tshekarda, Ural Mountains, Russian Federation.

F. Kansasiidae P1(Artinskian)
Sinitshenkova (2002a) places this family in Permothemistida (=Dicliptera) although Grimaldi and Engel (2005) are more tentative about this attribution.

First and Last: *Kansasia pulchra* in Beckemeyer (2000), Wellington Formation, Elmo site, Dickinson County, Kansas, United States.

F. Permothemistidae P1(Kungurian)-P2(Roadian)

First: e.g. *Pauciramus demoulini* in Carpenter (1992b), Koshelevka Formation, Tshekarda, Ural Mountains, Russian Federation.

Last: e.g. *Permothemis libelluloides* in Wootton and Kukalová-Peck (2000), Iva-Gora limestones, Soyana River, Arkhangelsk Region, Ural Mountains, Russian Federation.

**O. Megasecoptera** Brongniart, 1885 (Eubleptidodea, Megasecopterida, Mischopterida, Protohymenoptera) Carboniferous(Bashkirian)-Permian(Roadian)

F. Alectoneuridae Kukalová-Peck, 1975(Allectoneuridae) P1(Sakmarian)

First and Last: *Alectoneura europaea* in Carpenter (1992b), Obora locality, Bačov Beds, Letovice Formation, Moravia, Czech Republic.

F. Anchineuridae Carpenter, 1963a C2(Kasimovian)

First and Last: *Anchineura hispanica* in Brauckmann (1993), Magdalena shales, La Magdalena, León Province, Spain.

F. Aspidohymenidae P2(Roadian)

First and Last: *Aspidohymen extensus* in Carpenter (1992b), Baitugan Formation, Tikhie Gory, Kama River, Tatarstan, Russian Federation.

F. Aspidothoracidae C2(Moscovian)-C2(Kasimovian)

First: e.g. *Aspidothorax tristrata* Brauckmann and Herd, 2003, Osnabrück Formation, Piesberg quarry, Lower Saxony, Germany.

Last: *Aspidothorax triangularis* in Brauckmann and Herd (2003), Upper Coal Measures, Commentry, Allier, France.

F. Aykhalidae Sinitshenkova, 1994 P1(Asselian)

First and Last: *Aykhal helenae* in Sinitshenkova (2002a), Aykhal Formation, Markha River, Aykhal, Sakha (Yakutia) Republic, Russian Federation.

F. Bardohymenidae C2(Bashkirian)-P1(Kungurian)

First: e.g. *Sylvohymen pintoi* Brauckmann et al., 2003, Vorhalle Beds, Hagen-Vorhalle, Schmiedestraße, Wuppertal, North Rhine-Westphalia, Germany.

Last: e.g. *Sylvohymen robustus* in Brauckmann et al. (2003), Koshelevka Formation, Tshekarda, Ural Mountains, Russian Federation.

F. Brodiidae C2(Bashkirian)-C2(Moscovian)

First: *Brodia priscotincta* in Brauckmann and Herd (2003), Dudley coal measures, South Staffordshire Coalfield, Staffordshire, United Kingdom.

Last: e.g. *Pyobrodia janseni* Zessin, 2006, Osnabrück Formation, Piesberg quarry, Lower Saxony, Germany.

F. Brodiopteridae C2(Bashkirian)

e.g. *Brodioptera stricklani* Nelson and Tidwell, 1987, Manning Canyon Shale Formation, Lehi, Utah, United States.

F. Carbonopteridae C2(Moscovian)

First and Last: *Carbonoptera furcaradii* in Brauckmann (1991), Borehole 38 (Hangard), Neunkirchen, Saarland, Germany.

F. Corydaloididae C2(Kasimovian)

First and Last: *Corydaloides scudderi* in Wootton and Kukalová-Peck (2000), Upper Coal Measures, Commentry, Allier, France.

F. Engisopteridae Kukalová-Peck, 1975 P1(Sakmarian)

First and Last: *Engisoptera simplices* in Carpenter (1992b), Obora locality, Bačov Beds, Letovice Formation, Moravia, Czech Republic.

F. Eubleptidae C2(Moscovian)

e.g. *Eubleptus danielsi* in Sinitshenkova (2002a), Carbondale Formation, Mazon Creek, Illinois, United States.

F. Foririidae C2(Kasimovian)

First and Last: *Foriria maculata* in Béthoux et al. (2004a), Upper Coal Measures, Commentry, Allier, France.

F. Ischnoptilidae Carpenter, 1951(Ichnoptilidae) C2(Kasimovian)

First and Last: *Ischnoptilus elegans* in Béthoux et al. (2004b), Upper Coal Measures, Commentry, Allier, France.

F. Mischopteridae C2(Moscovian)-C2(Kasimovian)

First: *Mischoptera douglassi* in Labandeira (2001), Carbondale Formation, Mazon Creek, Illinois, United States.

Last: e.g. *Mischoptera nigra* in Wootton and Kukalová-Peck (2000), Upper Coal Measures, Commentry, Allier, France.

F. Moravohymenidae P1(Sakmarian)

First and Last: *Moravohymen vitreus* in Zajíc and Štamberg (2004), Obora locality, Bačov Beds, Letovice Formation, Moravia, Czech Republic.

F. Namurodiaphidae Kukalová-Peck and Brauckmann, 1990 C2(Bashkirian)
This family was originally placed in the Diaphanopterodea. Although its systematic position remains uncertain, most authors now place it in Megasecoptera (Sinitshenkova, 2002a; Prokop and Ren, 2007).

First and Last: *Namurodiapha sippelorum* in Brauckmann et al. (2003), Vorhalle Beds, Hagen-Vorhalle, Schmiedestraße, Wuppertal, North Rhine-Westphalia, Germany.

F. Protagrionidae (Protagriidae) C2(Kasimovian)

First and Last: *Protagrion audouini* in Béthoux and Nel (2003a), Upper Coal Measures, Commentry, Allier, France.

F. Protohymenidae (Permohymenidae) P1(Asselian)-P2(Roadian)
Beckemeyer 2000 lists *Permohymen schucherti* in Protohymenidae and neither he nor Sinitshenkova 2002a mention Permohymenidae at all.

First: *Sunohymen xishanensis* Hong, 1985, Shanxi Formation (Taiyuan Entomassemblage), Xishan Mountain, Shanxi Province, China.

Last: *Ivahymen constrictus* in Rohdendorf (1991), Iva-Gora limestones, Soyana River, Arkhangelsk Region, Ural Mountains, Russian Federation.

F. Scytohymenidae P1(Kungurian)

e.g. *Oceanoptera elenae* Shcherbakov *in* Shcherbakov et al., 2009, Pospelovo Formation, Russky Island, Primorye, Russian Federation.

F. Sphecopteridae Carpenter, 1951 C2(Kasimovian)-P1(Kungurian)

First: e.g. *Sphecoptera gracilis* in Carpenter (1992b), Upper Coal Measures, Commentry, Allier, France.

Last: *Cyclocelis* sp. in Rasnitsyn et al. (2005), Lek-Vorkuta Formation, Vorkuta Group, Pechora Cola Basin, Komi Republic, Russian Federation.

F. Sphecorydaloididae Pinto, 1994(Sphecocorydaloididae) P1(Asselian)

First and Last: *Sphecorydaloides lucchesei* in Pinto and Adami-Rodrigues (1999), Bajo de Véliz Formation (Pallero Member), Paganzo Basin, Sierra Grande de San Luis, San Luis Province, Argentina.

F. Vorkutiidae C2(Kasimovian)-P1(Kungurian)

First: *Siberiohymen asiaticus* in Rohdendorf (1991), Alykaeva Formation, Kuznetsk Basin, Siberian Federal District, Russian Federation.

Last: e.g. *Vorkutia dimina* Novokshonov, 1998b, Koshelevka Formation, Tshekarda, Ural Mountains, Russian Federation.

F. 'Xenopteridae' Pinto, 1986 C2(Bashkirian)-P1(Asselian)
This family name is a junior homonym of Xenopteridae Riek (Orthoptera). A replacement name has been submitted to the ICZN Commission, case no. 3634.

First: *Xenoptera riojaensis* Pinto, 1986, Malanzán Formation, Malanzán, La Rioja Province, Argentina.

Last: '*Philiasptilon*' *huenickeni* in Sinitshenkova (2002a), Bajo de Véliz Formation (Pallero Member), Paganzo Basin, Sierra Grande de San Luis, San Luis Province, Argentina.

**O. Palaeodictyoptera** Goldenberg, 1877 (Anisaxia, Archaehymenoptera, Breyerida, Dictyoneurida, Eopalaeodictyoptera, Hemiodonata, Protocicadida, Protohemiptera, Synarmogoidea) Carboniferous(Serpukhovian)-Permian(Capitanian)

F. Aenigmatidiidae P2(Roadian)

First and Last: *Aenigmatidia kaltanica* in Prokop and Nel (2004), Kuznetsk Formation (Mitino Horizon), Kaltan, Kemerovo Region, Russian Federation.

F. Ancopteridae Kukalová-Peck, 1975 P1(Sakmarian)
Family transferred from Megasecoptera by Sinitshenkova (2002a).

First and Last: *Ancoptera permiana* in Sinitshenkova (2002a), Obora locality, Bačov Beds, Letovice Formation, Moravia, Czech Republic.

F. Archaemegaptilidae C2(Bashkirian)-C2(Kasimovian)

First: *Archaemegaptilus schloesseri* Brauckmann et al., 2003, Vorhalle Beds, Hagen-Vorhalle, Schmiedestraße, Wuppertal, North Rhine-Westphalia, Germany.

Last: *Arachaemegaptilus kiefferi* in Brauckmann et al. (2003), Upper Coal Measures, Commentry, Allier, France.

F. Archaeoptilidae C2(Kasimovian)
Considered by Carpenter (1992b) to be Palaeoptera *incertae sedis*, Sinitshenkova (2002a) considers Archaeoptilidae to be a distinct family in Palaeodictyoptera.

First and Last: *Archaeoptilus ingens* in Carpenter (1992b), Middle Upper Coal Measures, near Chesterfield, Derbyshire, United Kingdom.

F. Arcioneuridae Kukalová-Peck, 1975 P1(Sakmarian)
Family transferred from Megasecoptera by Sinitshenkova (2002a).

e.g. *Arcioneura juveniles* in Carpenter (1992b), Obora locality, Bačov Beds, Letovice Formation, Moravia, Czech Republic.

F. Breyeriidae C2(Bashkirian)-C2(Kasimovian)

First: *Jugobreyeria sippelorum* in Brauckmann et al. (2003), Vorhalle Beds, Hagen-Vorhalle, Schmiedestraße, Wuppertal, North Rhine-Westphalia, Germany.

Last: e.g. *Breyeria boulei* in Brauckmann et al. (1985), Upper Coal Measures, Commentry, Allier, France.

F. Calvertiellidae (Mongolianidae, Mongolodictyidae) C2(Gzhelian)-P2(Capitanian)
Mongolodictyidae is considered a separate family by Sinitshenkova (2002a) but a junior synonym by Béthoux et al. (2007b), however, the type genus is a junior homonym so the family was renamed Mongolianidae Özdikmen 2008b.

First: *Carrizopteryx arroyo* in Béthoux et al. (2007b), Bursum Formation (Red Tanks Member), Carrizo Arroyo, New Mexico, United States.

Last: *Mongolianus callidus* in Özdikmen (2008b), Tsankhi (Tsankhin) Formation, Bor-Tolgoy, Ömnögovi (South Gobi) Aimag, Mongolia. (Listed by Béthoux et al. 2007b under the original name of *Mongolodictya callida*, however this genus name is a junior homonym of *Mongolodictya* Gorjunova 1988, so was renamed by Özdikmen 2008b.)

F. Caulopteridae Kukalová-Peck, 1975 P1(Sakmarian)
Family transferred from Megasecoptera by Sinitshenkova (2002a).

First and Last: *Cauloptera colorata* in Carpenter (1992b), Obora locality, Bačov Beds, Letovice Formation, Moravia, Czech Republic.

F. Cryptoveniidae C2(Moscovian)
Placed in Palaeoptera *incertae sedis* by Carpenter (1992b), Sinitshenkova (2002a) places this family in the Palaeodictyoptera.

First and Last: *Cryptovenia moyseyi* in Carpenter (1992b), below the Top Hard Coal, Middle Coal Measures, Shipley Manor Claypit, Ilkeston, Derbyshire, United Kingdom.

F. Dictyoneurellidae C2(Kasimovian)
Placed in Palaeoptera *incertae sedis* by Carpenter (1992b), Sinitshenkova (2002a) places this family in the Palaeodictyoptera.

First and Last: *Dictyoneurella perfecta* in Carpenter (1992b), Upper Coal Measures, Commentry, Allier, France.

F. Dictyoneuridae C2(Bashkirian)-P1(Artinskian)

First: e.g. *Dictyoneura kemperi* in Brauckmann et al. (2003), Vorhalle Beds, Hagen-Vorhalle, Schmiedestraße, Wuppertal, North Rhine-Westphalia, Germany.

Last: e.g. *Goldenbergia formosa* Sharov and Sinitshenkova, 1977, Nizhnyaya Burguklya Formation, Fatyanikha River, Krasnoyarsk Krai, Siberian Federal District, Russian Federation.

F. Elmoboriidae (Elmoboridae) P1(Sakmarian)-P1(Artinskian)

First: *Oboria longa* in Carpenter (1992b), Obora locality, Bačov Beds, Letovice Formation, Moravia, Czech Republic.

Last: *Elmoboria piperi* in Beckemeyer (2000), Wellington Formation, Elmo site, Dickinson County, Kansas, United States.

F. Eubrodiidae Sinitshenkova, 2002a C2(Moscovian)
Type genus taken out of the megasecopteran family Brodiidae by Sinitshenkova (2002a).

First and Last: *Eubrodia dabasinskasi* in Carpenter (1997), Carbondale Formation, Mazon Creek, Illinois, United States.

F. Eugereonidae (Cockerelliellidae) C2(Kasimovian)-P1(Sakmarian)

First: e.g. *Dictyoptilus sepultus* in Wootton and Kukalová-Peck (2000), Upper Coal Measures, Commentry, Allier, France.

Last: *Eugereon boeckingi* in Sinitshenkova (2002a), Lebachian Shales (Lower Rotliegend), Birkenfeld, Rhineland-Palatinate, Germany.

F. Eukulojidae (Eokulojidae, Eukulojudae) P1(Kungurian)-P2(Roadian)

First: *Eukuloja uralica* Sinitshenkova, 1981b, Koshelevka Formation, Tshekarda, Ural Mountains, Russian Federation.

Last: e.g. *Eukuloja cubitalis* in Sinitshenkova (2002a), Iva-Gora limestones, Soyana River, Arkhangelsk Region, Ural Mountains, Russian Federation.

F. Fouqueidae C2(Moscovian)-C2(Kasimovian)

First: *Neofouquea suzannae* in Carpenter (1997), Carbondale Formation, Mazon Creek, Illinois, United States.

Last: e.g. *Fouquea lacroixi* in Carpenter (1992b), Upper Coal Measures, Commentry, Allier, France.

F. Frankenholziidae C2(Moscovian)
Family transferred from Megasecoptera by Sinitshenkova (2002a).

First and Last: *Frankenholzia culmanni* in Brauckmann (1991), Frankenholz Mine, Neunkirchen, Saarland, Germany.

F. Graphiptilidae C2(Bashkirian)-C2(Kasimovian)

First: e.g. *Petteiskya volmensis* in Brauckmann et al. (2003), Vorhalle Beds, Hagen-Vorhalle, Schmiedestraße, Wuppertal, North Rhine-Westphalia, Germany.

Last: e.g. *Graphiptilus heeri* in Brauckmann et al. (1985), Upper Coal Measures, Commentry, Allier, France.

F. Hanidae Kukalová-Peck, 1975 C2(Gzhelian)-P1(Sakmarian)
Family transferred from Megasecoptera by Sinitshenkova (2002a).

First: *Forcynthia cynthiae* Sinitshenkova *in* Rasnitsyn et al., 2004a, Bursum Formation (Red Tanks Member), Carrizo Arroyo, New Mexico, United States.

Last: e.g. *Hana filia* in Sinitshenkova (2002a), Obora locality, Bačov Beds, Letovice Formation, Moravia, Czech Republic.

F. Heolidae C2(Kasimovian)

First and Last: *Heolus providentiae* in Prokop and Nel (2004), Ten-mile Series, East Providence, Rhode Island, United States.

F. Homoiopteridae (Homiopterigidae, Rochlingiidae, Thesoneuridae) C2(Bashkirian)-C2(Gzhelian)

First: e.g. *Homoioptera vorhallensis* in Prokop et al. (2006), Vorhalle Beds, Hagen-Vorhalle, Schmiedestraße, Wuppertal, North Rhine-Westphalia, Germany.

Last: e.g. *Parathesoneura carpenteri* in Sinitshenkova (2002a), Kata Formation, Chunya, Siberian Federal District, Russian Federation.

F. Homothetidae C2(Bashkirian)
This family is not included in Carpenter (1992b) but is referred to by Labandeira (1994) and Sinitshenkova (2002a).

First and Last: *Homothetus fossilis* in Handlirsch (1906), Lancaster Formation, Saint John, New Brunswick, Canada.

F. Jongmansiidae C2(Bashkirian)
Considered by Carpenter (1992b) to be Palaeodictyoptera *incertae sedis*, Sinitshenkova (2002a) retains family rank for Jongmansiidae.

e.g. *Jongmansia tuberculata* in Carpenter (1992b), Faisceau de Hendrik, Emma Mine, Limbourg, Netherlands.

F. Lamproptilidae (Lamproptiliidae) C2(Kasimovian)
Synonymised with Spilapteridae by Kukalová (1969a), Lamproptilidae is considered a separate family by Sinitshenkova (2002a).

First and Last: *Lamproptilia grandeuryi* in Wootton and Kukalová-Peck (2000), Upper Coal Measures, Commentry, Allier, France.

F. Lithomanteidae (Lithomantidae, Lusiellidae, Macropteridae) C2(Bashkirian)-C2(Kasimovian)

First: e.g. *Lithomantis varius* in Brauckmann et al. (2003), Vorhalle Beds, Hagen-Vorhalle, Schmiedestraße, Wuppertal, North Rhine-Westphalia, Germany.

Last: *Macroptera fariai* in Brauckmann et al. (1985), Alto do Pejao, Douro, Norte Region, Portugal.

F. Lithoptilidae C2(Kasimovian)-C2(Gzhelian)
Previously considered as a junior synonym of Megaptilidae (e.g. Carpenter, 1992b), Sinitshenkova (2002a) considers Lithoptilidae to be a separate family.

First: *Lithoptilus boulei* in Carpenter (1992b), Upper Coal Measures, Commentry, Allier, France.

Last: "near *Lithoptilus*" in Rowland (1997), Bursum Formation (Red Tanks Member), Carrizo Arroyo, New Mexico, United States. (Listed by Rowland 1997 in Megaptilidae but here considered Lithoptilidae.)

F. Lycocercidae (Lycocericidae) C2(Bashkirian)-C2(Gzhelian)

First: *Lycocercus bouckaerti* in Kukalová (1969b), Vorhalle Beds, Hagen-Vorhalle, Schmiedestraße, Wuppertal, North Rhine-Westphalia, Germany.

Last: e.g. *Madera mamayi* in Carpenter (1992b), Madera Formation, Manzano Mountains, New Mexico, United States.

F. Mecynopteridae C2(Bashkirian)-C2(Moscovian)
The type genus of this family was listed by Carpenter (1992b) as Palaeodictyoptera, Family Uncertain. Labandeira (1994) lists the family in Megasecoptera after Kukalová-Peck (1975).

First: *Mecynoptera tuberculata* Bolton, 1921, Middle Coal Measures, Sparth Bottoms, Lancashire, United Kingdom.

Last: *Mecynoptera splendida* in Béthoux et al. (2007b), Flénu, Wallonia, Hainaut Province, Belgium.

F. Mecynostomatidae C2(Kasimovian)

First and Last: *Mecynostomata dohrni* in Wootton and Kukalová-Peck (2000), Upper Coal Measures, Commentry, Allier, France.

F. Megaptilidae C2(Kasimovian)

First and Last: *Megaptilus blanchardi* in Wootton and Kukalová-Peck (2000), Upper Coal Measures, Commentry, Allier, France.

F. Namuroningxiidae Prokop and Ren, 2007 C2(Bashkirian)

First and Last: *Namuroningxia elegans* Prokop and Ren, 2007, Tupo Formation, Qilianshan Mountains, Ningxia/Gansu/Inner Mongolia, China.

F. Peromapteridae C2(Kasimovian)
Formerly considered in Eugereonidae (e.g. Carpenter, 1992b), Sinitshenkova (2002a) considers Peromapteridae to be a seaparate family.

First and Last: *Peromaptera filholi* in Wootton and Kukalová-Peck (2000), Upper Coal Measures, Commentry, Allier, France.

F. Polycreagridae C2(Kasimovian)
Synonymised with Lycoceridae by Kukalová (1969b), Polycreagridae is considered a separate family by Sinitshenkova (2002a) and Prokop and Ren (2007).

First and Last: *Polycreagra elegans* in Carpenter (1992b), Rhode Island Formation, Narragansett basin, Rhode Island, United States.

F. Psychroptilidae C2(Gzhelian)

First and Last: *Psychroptilus burrettae* in Jell (2004), Wynyard Tillite, Hellyer Gorge, Tasmania, Australia.

F. Saarlandiidae C2(Moscovian)
Considered by Carpenter (1992b) to be Palaeodictyoptera *incertae sedis*, Sinitshenkova (2002a) considers Saarlandiidae to be a distinct family.

First and Last: *Saarlandia flexisubcostata* in Carpenter (1992b), Geisheck Formation, Saarbrücken, Saarland, Germany.

F. Spilapteridae (Neuburgiidae) C1(Serpukhovian)-P1(Kungurian)

First: *Delitzschala bitterfeldensis* Brauckmann and Schneider, 1996, Bitterfeld/Delizsch area, Bitterfeld/Delitzsch area, Saxony-Anhalt, Germany.

Last: e.g. *Dunbaria borealis* in Rasnitsyn et al. (2005), Lek-Vorkuta Formation, Vorkuta Group, Pechora Cola Basin, Komi Republic, Russian Federation.

F. 'Stobbsiidae (Stobsiidae)' C2(Moscovian)
The type genus was listed in Breyeriidae by Carpenter (1992b). Considered a separate family by Sinitshenkova (2002a), but this family has not been formally named.

First and Last: *Stobbsia woodwardiana* in Carpenter (1992b), Peacock marls, Foley, near Longton, Staffordshire, United Kingdom.

F. Straeleniellidae Laurentiaux-Vieira and Laurentiaux, 1986 C2(Bashkirian)
Family not mentioned at all by Sinitshenkova (2002a).

e.g. *Straeleniella namurensis* Laurentiaux-Vieira and Laurentiaux, 1986, grey-black schists, Amercoeur Colliery, Wallonia, Hainaut Province, Belgium.

F. Synarmogidae C2(Bashkirian)
Synonymised with Lithomantidae by Kukalová (1969b), Synarmogidae is considered a separate family by Sinitshenkova (2002a) and Prokop and Ren (2007).

First and Last: *Synarmoge ferrarii* in Carpenter (1992b), Wendeischen Mines, Ruhr, North Rhine-Westphalia, Germany.

F. Tchirkovaeidae C2(Kasimovian)-C2(Gzhelian)

First: e.g. *Paimbia fenestrata* in Carpenter (1992b), Lower Kata Formation, Paymbu, Siberian Federal District, Russian Federation.

Last: e.g. *Paimbia ultima* Sinitshenkova, 1981a, Kata Formation, Chunya, Siberian Federal District, Russian Federation.

## Odonatoptera

**O. Geroptera** Brodsky, 1994 Carboniferous(Bashkirian)-Carboniferous(Bashkirian)

F. Eugeropteridae C2(Bashkirian)

e.g. *Eugeropteron lunatum* in Gutiérrez et al. (2000), Malanzán Formation, Malanzán, La Rioja Province, Argentina.

**O. Odonata** Fabricius, 1793 (Libellulida, Permodonata)
Carboniferous(Moscovian)-Quaternary(Holocene)

To include all taxa within Nodialata. *Euarchistigma atrophium* from the Crato Formation is now included in Dysagrionidae and the genus *Cretarchistigma* is *incertae sedis* or Hemiphlebiidae, leaving Pseudostigmatidae without a fossil record. Fossils previously assigned to Amphipterygidae are now Steleopteridae (Fleck et al., 2001), leaving Amphipterygidae without a fossil record. Nel and Paicheler (1992) state that Chlorocyphidae have no fossil record.

F. Aeschnidiidae J3(Kimmeridgian)-K2(Cenomanian)
Fleck and Nel (2003) figure one specimen and mention another that belong to this family which could be from the Lias but could also be Lower Cretaceous.

First: e.g. *Brunetaeschnidium nusplingensis* in Fleck and Nel (2003), Nusplingen Lithographic Limestone, Westerberg/Grosser Heuberg, Baden-Württenburg, Germany.

Last: *Tauropteryx krassilovi* in Fleck and Nel (2003), Sel'bukhra near Prokhadnoye, Bakhchisarayskiy district, Crimea, Ukraine.

F. Aeshnidae (Aeschnidae) J3(Tithonian)-Holocene

First: *Morbaeschna muensteri* in Nel et al. (1994), Solenhofen Lithographic Limestone, Solenhofen/Eichstadt, Bavaria, Germany.

F. Aktassiidae J3(Oxfordian)-K1(Barremian)

First: *Aktassia magna* in Nel et al. (1998), Karabastau Formation, Karatau Range, Tien Shan mountains, Kazakhstan.

Last: *Pseudocymatophlebia hennigi* Nel et al., 1998, Upper Weald Clay Formation, Smokejacks Brickworks, Surrey, United Kingdom.

F. Allopetaliidae K1(Valanginian)-Holocene

First: e.g. *Baissaeshna zherikhini* Bechly et al., 2001, Zaza Formation, Baissa, Buryatia, Russian Federation.

F. Araripechlorogomphidae Bechly and Ueda, 2002 K1(Aptian)

First and Last: *Araripechlorogomphus muratai* in Bechly (2007b), Crato Formation, Araripe Basin, Ceará, Brazil.

F. Araripegomphidae Bechly, 1996 K1(Aptian)

e.g. *Araripegomphus hanseggeri* in Bechly (2007b), Crato Formation, Araripe Basin, Ceará, Brazil.

F. Araripelibellulidae Bechly, 1996 K1(Berriasian)-K1(Aptian)

First: e.g. *Araripelibellula britannica* Fleck et al., 2008, Lulworth Formation, Durlston Bay, Dorset, United Kingdom.

Last: e.g. *Araripelibellula martinsnetoi* in Bechly (2007b), Crato Formation, Araripe Basin, Ceará, Brazil.

F. Araripephlebiidae Bechly, 1998c K1(Aptian)

First and Last: *Araripephlebia mirabilis* in Bechly (2007b), Crato Formation, Araripe Basin, Ceará, Brazil.

F. Archithemistidae (Architemistidae) T3(Rhaetian)-J1(Toarcian)

First: *Archithemis liassina* in Jarzembowski (1999), Cotham Member, Lilstock Formation, Penarth Group2, near Axmouth, Dorset, United Kingdom. (Originally described as *Diastatommites liassina*.)

Last: *Sogdothemis modesta* in Sukatsheva and Rasnitsyn (2004), Sagul Formation, Sai-Sagul, Batkenskii District, Kyrgyzstan.

F. Asiopteridae (Oreopteridae) J1(Toarcian)-J3(Oxfordian)

First: e.g. *Amblyopteron breve* in Sukatsheva and Rasnitsyn (2004), Sagul Formation, Sai-Sagul, Batkenskii District, Kyrgyzstan.

Last: e.g. *Asiopteron antiquum* in Nel et al. (1993), Karabastau Formation, Karatau Range, Tien Shan mountains, Kazakhstan.

F. Austroperilestidae Petrulevičius and Nel, 2005 Eoc.(Ypresian)

First and Last: *Austroperilestes hunco* Petrulevičius and Nel, 2005, La Huitrera Formation, Laguna del Hunco, Chubut Province, Argentina.

F. Batkeniidae T2(Anisian)-T3(Carnian)

First: *Voltzialestes triasicus* Nel et al., 1996, Grès à Voltzia, Bas-Rhin/Moselle, Northern Vosges Mountains, France.

Last: e.g. *Batkenia pusilla* in Nel et al. (1999c), Madygen Formation, Madygen/Dzhailoucho, south Fergana Valley, Kyrgyzstan.

F. Bechlyidae Jarzembowski and Nel, 2002 C2(Moscovian)

First and Last: *Bechlya ericrobinsoni* in Zessin (2008), Farrington Formation, Writhlington, Somerset, United Kingdom.

F. Bolcacorduliidae Gentilini, 2002 Eoc.(Ypresian)

First and Last: *Bolcacordulia paradoxa* Gentilini, 2002, Pesciara site, Monte Bolca limestone, Province of Verona, Veneto, Italy.

F. Bolcathoridae Gentilini, 2002 Eoc.(Ypresian)

First and Last: *Bolcathore colorata* Gentilini, 2002, Pesciara site, Monte Bolca limestone, Province of Verona, Veneto, Italy.

F. Callimokaltaniidae P2(Roadian)

First and Last: *Callimokaltania martynovi* in Zessin (2008), Kuznetsk Formation (Mitino Horizon), Kaltan, Kemerovo Region, Russian Federation.

F. Calopterygidae (Agriidae) Eoc.(Priabonian)-Holocene

First: Figured in Fleck et al. (2009), Baltic amber.

F. Campterophlebiidae (Karatawiidae) J1(Sinemurian)-K1(Berriasian)

First: *Dorsettia laeta* in Nel et al. (1993), Black Ven Marls, Charmouth, Dorset, United Kingdom.

Last: *Pritykinia rasnitsyni* Nel et al., 2009a, Markha, deposit unknown, Markha River, Aykhal, Sakha (Yakutia) Republic, Russian Federation.

F. Camptotaxineuridae P1(Artinskian)
Huguet et al. (2002) suggest this family could belong in Palaeodictyoptera.

First and Last: *Camptotaxineura ephialtes* in Huguet et al. (2002), Wellington Formation, Elmo site, Dickinson County, Kansas, United States.

F. Coenagrionidae (Agrionidae, Coenagriidae, Protoneuridae *partim*) K1(Aptian)-Holocene

First: Figured in Jell (2004), Koonwarra Fossil Bed (Korumburra Group), South Gippsland, Victoria, Australia. (All other pre-Tertiary specimens attributed to this family have since been removed, so the attribution of this specimen to the Coenagrionidae remains tentative.)

F. Cordulegastridae Olig.(Rupelian)-Holocene

First: '*Petalura*' *acutipennis* in Nel and Paicheler (1992), Braunkhole, Sieblos, Hesse, Germany.

F. Cordulephyidae Pal.(Thanetian)-Holocene

First: *Palaeophya argentina* Petrulevičius and Nel, 2009, Maíz Gordo Formation, Salta Group, Salta/Jujuy provinces, Argentina.

F. Corduliidae (Synthemistidae, Sythemistidae) Eoc.(Ypresian)-Holocene

First: *Molercordulia karinae* Bechly, 2005a, Fur Formation (Mo Clay), Limfjord/Mors Peninsula/Fur Island, Jutland, Denmark.

F. Cretacoenagrionidae Bechly, 1996 K1(Hauterivian)

First and Last: *Cretacoenagrion alleni* in Jarzembowski et al. (1998), Lower Weald Clay Formation, Clockhouse Brickworks, Surrey, United Kingdom.

F. Cretapetaluridae Nel et al., 1998 K1(Berriasian)-K1(Aptian)

First: *Anglopetalura magnifica* Coram and Nel, 2009, Durlston Formation (Stair Hole Member), Durlston Bay, Dorset, United Kingdom.

Last: e.g. *Cratopetalura petruleviciusi* Nel and Bechly, 2009, Crato Formation, Araripe Basin, Ceará, Brazil.

F. Cyclothemistidae Bechly, 1997 T3(Carnian)-J1(Toarcian)

First: *Pseudotriassothemis nipponensis* in Bechly (1997), Momonoki Formation, Ominé Coal Field, Yamaguchi, Japan.

Last: e.g. *Cyclothemis sagulica* in Bechly (1997), Sagul Formation, Sai-Sagul, Batkenskii District, Kyrgyzstan. (This species, along with *Shurabiola nana*, were listed under Archithemistidae by Sukatsheva and Rasnitsyn 2004, in which they had been originally described.)

F. Cymatophlebiidae J2(Callovian)-K1(Barremian)

First: *Sinacymatophlebia mongolica* Nel and Huang, 2009, Jiulongshan Formation, near Daohugou, Ningcheng county, Inner Mongolia, China.

Last: e.g. *Cymatophlebia standingae* in Bechly et al. (2001), Upper Weald Clay Formation, Rudgwick Brickworks, near Horsham, West Sussex, United Kingdom.

F. Ditaxineuridae P1(Artinskian)-P1(Kungurian)

First: e.g. *Ditaxineura anomalostigma* in Zessin (2008), Wellington Formation, Elmo site, Dickinson County, Kansas, United States.

Last: *Proditaxineura pritykinae* in Huguet et al. (2002), Koshelevka Formation, Tshekarda, Ural Mountains, Russian Federation.

F. Dysagrionidae (Congqingiidae, Euarchistigmatidae, Thaumatoneuridae) K1(Barremian)-Holocene
For a discussion on the name of this family see Rust et al. (2008).

First: *Congqingia rhora* in Nel and Arillo (2006), Laiyang Formation, Laiyang County, Shandong Province, China.

F. Enigmaeshnidae Nel et al., 2008 K2(Cenomanian)

First and Last: *Enigmaeshna deprei* Nel et al., 2008, Puy-Puy quarry, Tonnay-Charente, Charente-Maritime, France.

F. Eocorduliidae Bechly, 1996 K1(Berriasian)

First and Last: *Eocordulia cretacea* Pritykina, 1986, Mogotuin Formation, Sum of Manlai, Mogotuin-Del-Ula mountain, Ömnögovi (South Gobi) Aimag, Mongolia.

F. Eosagrionidae J1(Toarcian)

First and Last: *Eosagrion risi* in Nel and Paicheler (1993), Upper Lias, Dobbertin, Mecklenburg-Vorpommern, Germany.

F. Epallagidae (Euphaeidae) Eoc.(Ypresian)-Holocene

First: *Labandeiraia europae* Petrulevičius et al., 2007, Fur Formation (Mo Clay), Limfjord/Mors Peninsula/Fur Island, Jutland, Denmark.

F. Erichschmidtiidae Bechly, 1996 J3(Oxfordian)
Fleck et al. (2003) move *Prostenophlebia* to Prostenophlebiidae, leaving Erichschmidtiidae with only one genus.

First and Last: *Erichschmidtia nigrimontana* in Bridges (1994), Karabastau Formation, Karatau Range, Tien Shan mountains, Kazakhstan.

F. Eumorbaeschnidae Bechly et al., 2001 J3(Tithonian)

First and Last: *Eumorbaeschna jurassica* in Bechly et al. (2001), Solenhofen Lithographic Limestone, Solenhofen/Eichstadt, Bavaria, Germany.

F. Euthemistidae J3(Oxfordian)
Bechly (1997) removed *Sphenophlebia*, *Mesoepiophlebia*, *Ensphingophlebia* and *Proeuthemis* to the Sphenophlebiidae, leaving Euthemistidae with only one genus.

e.g. *Euthemis multinervosa* in Jarzembowski (1990), Karabastau Formation, Karatau Range, Tien Shan mountains, Kazakhstan.

F. Frenguelliidae Petrulevičius and Nel, 2003a(Frengueliidae) Eoc.(Ypresian)

First and Last: *Frenguellia patagonica* in Petrulevičius and Nel (2007), La Huitrera Formation, Laguna del Hunco, Chubut Province, Argentina.

F. Gomphaeschnidae (Gomphoaeschnidae) K1(Berriasian)-Holocene

First: e.g. *Cretalloaeschna cliffordae* in Bechly et al. (2001), Durlston Formation (Stair Hole Member), Durlston Bay, Dorset, United Kingdom.

F. Gomphidae (Gomphinidae) Olig.(Rupelian)-Holocene

First: *Ictinogomphus*? sp. in Prokop and Fikaček (2007), Seifhennersdorf diatomite, Upper Lusatia, Free State of Saxony, Germany.

F. Gondvanogomphidae Bechly, 1996(Gondwanogomphidae) K1(Aptian)

First and Last: *Gondvanogomphus bartheli* in Schlüter (2003), Abu Ballas Formation, Abu Ballas, Gilf Kebir, Egypt.

F. Hemeroscopidae K1(Valanginian)-K1(Aptian)

First: *Hemeroscopus baissicus* in Bechly et al. (1998), Zaza Formation, Baissa, Buryatia, Russian Federation.

Last: *Abrohemeroscopus mengi* Ren et al., 2003, Jiufotang Formation, Beishan, Yixian County, Liaoning Province, China.

F. Hemiphlebiidae J3(Tithonian)-Holocene

First: *Mersituria ludmilae* Vasilenko, 2005, Doronino Formation, Chernovskie Kopi, Chita, Transbaikalia, Russian Federation.

F. Hemizygopteridae (Hemizypopteridae) P1(Kungurian)

e.g.? *Hemizygopteron* cf. *uralense* in Huguet et al. (2002), Koshelevka Formation, Tshekarda, Ural Mountains, Russian Federation. (The original description of *Hemizygopteron uralense* Zalessky, 1955 mentions only that it is from the "Upper Permian" of the Urals. Huguet et al. 2002 state that the specimen is missing but give the same vague locality and age data as the original description. Rohdendorf 1991 synonymises *Hemizygopteron* with *Ditaxineurella* from the Kungurian of Tshekarda, and mentions two included species from the "Lower Permian of Urals". Thus, it is assumed here that both *H. uralense* and *H.* cf. *uralense* came from the same deposit.)

F. Henrotayiidae Fleck et al., 2003(Henrotayidae) J1(Toarcian)

First and Last: *Henrotayia marci* Fleck et al., 2003, Upper Lias, Bascharage and Sanem, Luxembourg district, Luxembourg.

F. Heterophlebiidae J1(Sinemurian)-J1(Toarcian)

First: *Heterophlebia* sp. in Nel et al. (1993), Black Ven Marls, Charmouth, Dorset, United Kingdom.

Last: *Heterophlebia buckmani* in Ansorge (1999), Upper Lias, Dobbertin, Mecklenburg-Vorpommern, Germany.

F. Hypolestidae Eoc.(Priabonian)-Holocene

First: e.g.? Figured in Bechly and Wichard (2008), Baltic amber.

F. Idionychidae Mio.(Langhian)-Holocene

First: *Mioidionyx stavropolensis* Nel et al., 2005d, Vishnevaya Balka, near Senghileevskoye Lake, Stavropol Krai, Russian Federation.

F. Isophlebiidae J2(Aalenian)-K1(Valanginian)

First: Mentioned in Pritykina (2006), Ichetuy Formation, Novospasskoye, Mukhorshibirsky District, Buryatia, Russian Federation. (Based on the odontofauna, Pritykina 2006 considers the Ichetuy Formation to be of Upper Jurassic age, in which case the oldest isophlebiid would be *Hemerobioides giganteus* from the Bathonian (J2) Stonesfield Slate in England, listed by Nel et al. 1993.)

Last: *Nacholonda crassicosta* in Nel et al. (1993), Zaza Formation, Baissa, Buryatia, Russian Federation.

F. Isostictidae K1(Aptian)-Holocene

First: *Eoprotoneura hyperstigma* in Bechly (2007b), Crato Formation, Araripe Basin, Ceará, Brazil. (Bechly 2007b lists this species in Protoneuridae: Isostictinae but this subfamily has subsequently been restored to family level and Protoneuridae shown to be polyphyletic e.g. Bybee et al., 2008.)

F. Juracorduliidae Bechly and Ueda, 2002 J3(Tithonian)

First and Last: *Juracordulia schiemenzi* Bechly, 1998a, Solenhofen Lithographic Limestone, Solenhofen/Eichstadt, Bavaria, Germany.

F. Juragomphidae Nel et al., 2001b J3(Oxfordian)

First and Last: *Juragomphus karatauensis* Nel et al., 2001b, Karabastau Formation, Karatau Range, Tien Shan mountains, Kazakhstan.

F. Juraheterophlebiidae Fleck et al., 2003 J3(Oxfordian)

First and Last: *Juraheterophlebia kazakhstanensis* Fleck et al., 2003, Karabastau Formation, Karatau Range, Tien Shan mountains, Kazakhstan.

F. Juralibellulidae Huang and Nel, 2007b J2(Callovian)

First and Last: *Juralibellula ningchengensis* Huang and Nel, 2007b, Jiulongshan Formation, near Daohugou, Ningcheng county, Inner Mongolia, China.

F. Kaltanoneuridae P2(Roadian)

First and Last: *Kaltanoneura bartenevi* in Zessin (2008), Kuznetsk Formation (Mitino Horizon), Kaltan, Kemerovo Region, Russian Federation.

F. Kargalotypidae P2(Wordian)
Bechly (1996) places this family in the Meganisoptera but Nel et al. (2001c) consider it Triadophlebiomorpha, here listed in the Odonata.

First and Last: *Kargalotypus kargalensis* in Nel et al. (2001c), Amanak Formation, Kargala, Belozersky District, Orenburg Region, Russian Federation.

F. Kennedyidae P1(Artinskian)-T3(Carnian)

First: e.g. *Opter brongniarti* in Zessin (2008), Wellington Formation, Elmo site, Dickinson County, Kansas, United States.

Last: e.g. *Kennedya carpenteri* in Nel et al. (1999c), Madygen Formation, Madygen/Dzhailoucho, south Fergana Valley, Kyrgyzstan.

F. Latibasaliidae Petrulevičius and Nel, 2004 Pal.(Thanetian)

e.g. *Latibasalia elongata* in Petrulevičius and Nel (2007), Maíz Gordo Formation, Salta Group, Salta/Jujuy provinces, Argentina.

F. Lestidae Pal.(Thanetian)-Holocene

First: '*Lestes*' *zalesskyi* in Nel and Paicheler (1994a), spongo-diatomaceous maar, Menat, Puy-de-Dôme, Auvergne, France.

F. Liadotypidae J1(Toarcian)

First and Last: *Liadotypus relictus* in Nel et al. (2001c), Sagul Formation, Sai-Sagul, Batkenskii District, Kyrgyzstan.

F. Liassogomphidae (Gomphitidae) J1(Toarcian)
The genus *Chrysogomphus* does not belong in this family (see Huang et al., 2003).

e.g. *Liassogomphus brodiei* in Etter and Kuhn (2000), Posidonia Shale, Hemmiken, Basel-Country, Switzerland.

F. Liassophlebiidae J1(Hettangian)-J1(Toarcian)

First: *Bavarophlebia schmeissneri* Nel and Petrulevičius, 2005, Early Lias (alpha 1 & 2), Sandpit Küfner, south of Pechgraben, Kulmbach, Bavaria, Germany.

Last: e.g. *Ferganophlebia insignis* in Sukatsheva and Rasnitsyn (2004), Sagul Formation, Sai-Sagul, Batkenskii District, Kyrgyzstan.

F. Liassostenophlebiidae Fleck et al., 2003 J1(Toarcian)

First and Last: *Liassostenophlebia germanica* Fleck et al., 2003, "Epsilon" Liassic, Geodenlage 2, Rhine-Danube canal, Bavaria, Germany.

F. Libellulidae K2(Turonian)-Holocene
*Condalia woottoni* is not a libellulid (see Nel and Paicheler, 1994b).

First: *Palaeolibellula zherikhini* Fleck et al., 1999, Kzyl-Zhar, Karatau Range, Tien Shan mountains, Kazakhstan.

F. Lindeniidae K1(Aptian)-Holocene

First: *Cratolindenia knuepfae* Bechly, 2000, Crato Formation, Araripe Basin, Ceará, Brazil.

F. Liupanshaniidae Bechly et al., 2001 K1(Barremian)-K2(Turonian)

First: *Paraliupanshania britannica* Bechly et al., 2001, Upper Weald Clay Formation, Rudgwick Brickworks, near Horsham, West Sussex, United Kingdom.

Last: *Paraliupanshania torvaldsi* Bechly et al., 2001, Kzyl-Zhar, Karatau Range, Tien Shan mountains, Kazakhstan.

F. Macromiidae Mio.(Burdigalian)-Holocene

First: *Epophthalmia biordinata* in Nel and Paicheler (1994b), Latah Formation, Spokane, Washington, United States.

F. Megapodagrionidae (Megapodogrionidae) Pal.(Thanetian)-Holocene

First: e.g. *Thanetophilosina menatensis* in Azar and Nel (2008), spongo-diatomaceous maar, Menat, Puy-de-Dôme, Auvergne, France.

F. Mesochlorogomphidae Fleck et al., 2008 K1(Barremian)

e.g. *Mesochlorogomphus crabbi* Fleck et al., 2008, Upper Weald Clay Formation, Smokejacks Brickworks, Surrey, United Kingdom.

F. Mesomantidiidae T3(Carnian)

First and Last: *Mesomantidion queenslandicum* in Jell (2004), Blackstone Formation, Ipswich Basin, Queensland, Australia.

F. Mesuropetalidae Bechly, 1996 J3(Oxfordian)-K1(Valanginian)

First: e.g. *Mesuropetala auliensis* in Bechly et al. (2001), Karabastau Formation, Karatau Range, Tien Shan mountains, Kazakhstan.

Last: *Mesurapetala magna* Bechly et al., 2001, Zaza Formation, Baissa, Buryatia, Russian Federation.

F. Mitophlebiidae T3(Carnian)

e.g. *Promitophlebia modica* in Bechly (1996), Madygen Formation, Madygen/Dzhailoucho, south Fergana Valley, Kyrgyzstan.

F. Myopophlebiidae J1(Toarcian)

e.g. *Paraheterophlebia marcusi* in Fleck et al. (2003), Upper Lias, Bascharage and Sanem, Luxembourg district, Luxembourg.

F. Nannogomphidae Bechly, 1996 J3(Tithonian)

e.g. *Nannogomphus buergeri* Bechly, 2003, Solenhofen Lithographic Limestone, Solenhofen/Eichstadt, Bavaria, Germany.

F. Nodalulaidae Lin et al., 2007 K1(Aptian)

First and Last: *Nodalula dalinghensis* Lin et al., 2007, Jianshangou beds, Yixian Formation, Liaoning Province, China.

F. Nothomacromiidae Carle, 1995(Pseudomacromiidae) K1(Aptian)
*Pseudomacromia* is re-named *Nothomacromia* in Carle (1995).

First and Last: *Nothomacromia sensibilis* in Bechly (2007b), Crato Formation, Araripe Basin, Ceará, Brazil. (*Conan barbarica* is a junior synonym.)

F. Oboraneuridae Zessin, 2008 P1(Sakmarian)

First and Last: *Oboraneura kukalovae* Zessin, 2008, Obora locality, Bačov Beds, Letovice Formation, Moravia, Czech Republic.

F. Palaeomacromiidae Petrulevičius et al., 1999(Bolcathemidae) Pal.(Thanetian)-Eoc.(Ypresian)

First: e.g. *Curviarculia delicata* Petrulevičius and Nel, 2002, Maíz Gordo Formation, Salta Group, Salta/Jujuy provinces, Argentina.

Last: *Bolcathemis nervosa* in Petrulevičius and Nel (2007), Pesciara site, Monte Bolca limestone, Province of Verona, Veneto, Italy.

F. Paracymatophlebiidae Bechly et al., 2001 J3(Oxfordian)

First and Last: *Paracymatophlebia splendida* Bechly et al., 2001, Karabastau Formation, Karatau Range, Tien Shan mountains, Kazakhstan.

F. Paragonophlebiidae Nel, 2009 J3(Oxfordian)-J3(Tithonian)

First: *Paragonophlebia inexpectata* Nel, 2009, Karabastau Formation, Karatau Range, Tien Shan mountains, Kazakhstan.

Last: *Paragonophlebia patriciae* Nel, 2009, Shar-Teg Formation, Shar-Teg Ula, Gobi-Altai Aimag, Mongolia.

F. Parastenophlebiidae Bechly, 2005b J3(Tithonian)

First and Last: *Parastenophlebia casta* in Bechly (2005b), Solenhofen Lithographic Limestone, Solenhofen/Eichstadt, Bavaria, Germany.

F. Paurophlebiidae Bechly, 1996 T3(Carnian)

e.g. *Paurophlebia lepida* in Vasilenko and Rasnitsyn (2007), Madygen Formation, Madygen/Dzhailoucho, south Fergana Valley, Kyrgyzstan.

F. Permaeschnidae P1(Artinskian)-P2(Roadian)

First: *Gondvanoptilon brasiliense* in Huguet et al. (2002), Irati Formation, Paraná Basin, São Paulo, Brazil.

Last: *Permaeschna dolloi* in Huguet et al. (2002), Iva-Gora limestones, Soyana River, Arkhangelsk Region, Ural Mountains, Russian Federation. (*P. proxima* considered a junior synonym in Huguet et al. (2002).)

F. Permagrionidae (Permagriidae) P1(Sakmarian)

First and Last: *Permagrion falklandicus* in Nel et al. (1999c), Lafonia Formation, Bodie Creek Head, East Falkland, Falkland Islands (Malvinas).

F. Permepallagidae P2(Roadian)
Zessin (2008) removed *Lodevia* from this family.

First and Last: *Permepallage angustissima* in Zessin (2008), Iva-Gora limestones, Soyana River, Arkhangelsk Region, Ural Mountains, Russian Federation.

F. Permolestidae (Solikamptilonidae) P2(Roadian)-P2(Wordian)

First: e.g. *Permolestes gracilis* in Nel et al. (1999c), Iva-Gora limestones, Soyana River, Arkhangelsk Region, Ural Mountains, Russian Federation.

Last: *Epilestes gallica* Nel et al., 1999c, Salagou Formation (Mérifons Member), Lodève Basin, Hérault, France.

F. Permophlebiidae Nel et al., 2001c P3(Wuchiapingian)

First and Last: *Permophlebia uralica* Nel et al., 2001c, Vostochno-Novikbozhskay borehole, Vorkuta Basin, Ural Mountains, Russian Federation. (Age of deposit given as "Early Upper Permian".)

F. Petaluridae K1(Aptian)-Holocene

First: *Argentinopetala archangelskyi* Petrulevičius and Nel, 2003b, Anfiteatro de Ticó Formation, Bajo Grande, Santa Cruz Province, Argentina.

F. Pholidoptilidae P2(Roadian)

First and Last: *Pholidoptilon camense* in Huguet et al. (2002), Baitugan Formation, Tikhie Gory, Kama River, Tatarstan, Russian Federation.

F. Piroutetiidae Nel, 1989 T3(Rhaetian)

First and Last: *Piroutetia liasina* in Nel et al. (2001c), "Lower Lias", Fort-Mouchard, Arçures, Jura, France.

F. Platycnemididae (Platycnemidae, Protoneuridae *partim*) Eoc.(Priabonian)-Holocene

First: e.g. *Platycnemis antiqua* in Weitschat and Wichard (2002), Baltic amber.

F. Polytaxineuridae P3(Changhsingian)

First and Last: *Polytaxineura stanleyi* in Huguet et al. (2002), Belmont insect beds, Newcastle Coal Measures, Belmont/Warner's Bay, New South Wales, Australia. (This species is erroneously listed in Permaeschnidae by Jell (2004).)

F. Priscalestidae Petrulevičius & Wappler *in* Wappler and Petrulevičius, 2007 Eoc.(Lutetian)

First and Last: *Priscalestes germanica* Petrulevičius & Wappler *in* Wappler and Petrulevičius, 2007, Eckfeld maar, Manderscheid, Rhineland-Palatinate, Germany.

F. Progobiaeshnidae Bechly et al., 2001(Progobiaeschnidae) K1(Barremian)-K1(Aptian)

First: *Gobiaeshna occulta* in Bechly et al. (2001), Anda-Khuduk Formation, Anda-Khuduk, Övörkhangai (Ubur-Khangaisk) Aimag, Mongolia.

Last: *Progobiaeshna liaoningensis* Bechly et al., 2001, Yixian unspecified, Yixian Formation, Liaoning Province, China. (The precise locality and deposit of this specimen is unknown, according to Bechly et al. 2001.)

F. Prohemeroscopidae Bechly and Ueda, 2002 J3(Tithonian)

e.g. *Prohemeroscopus jurassicus* Bechly et al., 1998, Solenhofen Lithographic Limestone, Solenhofen/Eichstadt, Bavaria, Germany. (Originally described in the Hemeroscopidae.)

F. Prostenophlebiidae Fleck et al., 2003 J3(Tithonian)

First and Last: *Prostenophlebia jurassica* in Fleck et al. (2003), Solenhofen Lithographic Limestone, Solenhofen/Eichstadt, Bavaria, Germany.

F. Proterogomphidae Bechly et al., 1998 J3(Tithonian)-K1(Aptian)

First: *Proterogomphus renateae* Bechly et al., 1998, Solenhofen Lithographic Limestone, Solenhofen/Eichstadt, Bavaria, Germany.

Last: e.g. *Cordulagomphus winkelhoferi* Bechly, 2007b, Crato Formation, Araripe Basin, Ceará, Brazil.

F. Protolindeniidae J3(Tithonian)

e.g. *Protolindenia viohli* Nel et al., 2001a, Solenhofen Lithographic Limestone, Solenhofen/Eichstadt, Bavaria, Germany.

F. Protomyrmeleontidae (Protomyrmeleonidae, Triassagrionidae) T3(Carnian)-K1(Hauterivian)

First: e.g. *Ferganagrion kirghiziensis* Nel et al., 2005e, Madygen Formation, Madygen/Dzhailoucho, south Fergana Valley, Kyrgyzstan.

Last: *Protomyrmeleon cretacicus* Nel and Jarzembowski, 1998, Lower Weald Clay Formation, Clockhouse Brickworks, Surrey, United Kingdom.

F. Rudiaeschnidae Bechly et al., 2001 K1(Berriasian)-K1(Aptian)

First: *Fuxiaeschna hsiufunia* Lin et al., 2004, Luohandong Formation, Datai Valley, Huating County, Gansu Province, China.

Last: *Rudiaeshna limnobia* in Bechly et al. (2001), Jianshangou beds, Yixian Formation, Liaoning Province, China.

F. Saxonagrionidae Nel et al., 1999a P2(Wordian)

First and Last: *Saxonagrion minutus* in Zessin (2008), Salagou Formation (Mérifons Member), Lodève Basin, Hérault, France.

F. Selenothemistidae (Turanothemistidae) J1(Toarcian)-J3(Oxfordian)

First: *Selenothemis liadis* in Nel (2009), Upper Lias, Dobbertin, Mecklenburg-Vorpommern, Germany.

Last: *Turanothemis nodalis* in Zessin (2005), Karabastau Formation, Karatau Range, Tien Shan mountains, Kazakhstan.

F. Sieblosiidae (Sublosiidae) Olig.(Rupelian)-Mio.(Tortonian)

First: e.g. *Stenolestes jucunda* in Nel et al. (2005c), Braunkhole, Sieblos, Hesse, Germany.

Last: *Stenolestes hispanicus* in Peñalver et al. (1999), diatomites (Cerdanya), Bellver de Cerdanya, Lleida Province, Spain.

F. Sonidae Pritykina, 1986 K1(Hauterivian)

First and Last: *Sona nectes* Pritykina, 1986, Gurvan-Eren Formation, Myangad, Khovd Aimag, Mongolia. (This species contains only the larval specimens as the supposed adults were described as a new family Proterogomphidae Bechly et al. 1998.)

F. Sphenophlebiidae Bechly, 1997 J1(Toarcian)-K1(Hauterivian)

First: e.g. *Mesoepiophlebia veronicae* in Nel et al. (2002), Upper Lias, Bascharage and Sanem, Luxembourg district, Luxembourg.

Last: e.g. *Proeuthemis pritykinae* in Fleck et al. (2004), Lower Weald Clay Formation, Clockhouse Brickworks, Surrey, United Kingdom.

F. Steleopteridae J3(Oxfordian)-J3(Tithonian)

First: *Auliella crucigera* in Fleck et al. (2001), Karabastau Formation, Karatau Range, Tien Shan mountains, Kazakhstan.

Last: e.g. *Parasteleopteron guischardi* Fleck et al., 2001, Solenhofen Lithographic Limestone, Solenhofen/Eichstadt, Bavaria, Germany.

F. Stenophlebiidae (Stenophlebidae) J3(Oxfordian)-K1(Aptian)

First: *Stenophlebia karatavica* in Fleck et al. (2003), Karabastau Formation, Karatau Range, Tien Shan mountains, Kazakhstan.

Last: *Cratostenophlebia schwickerti* Bechly, 2007b, Crato Formation, Araripe Basin, Ceará, Brazil.

F. Synlestidae (Chlorolestidae, Chorismagrionidae) J3(Tithonian)-Holocene

First: *Gaurimacia sophiae* Vasilenko, 2005, Doronino Formation, Chernovskie Kopi, Chita, Transbaikalia, Russian Federation.

F. Tarsophlebiidae J3(Oxfordian)-K1(Aptian)
Previous Lower Jurassic records do not belong to this family (Fleck et al., 2004).

First: e.g. *Turanophlebia martynovi* in Fleck et al. (2004), Karabastau Formation, Karatau Range, Tien Shan mountains, Kazakhstan.

Last: *Turanophlebia sinica* Huang and Nel, 2009a, Yixian Formation, Liaoning Province, China.

F. Triadophlebiidae T3(Carnian)

e.g. *Triassophlebia madygenica* in Nel et al. (1999c), Madygen Formation, Madygen/Dzhailoucho, south Fergana Valley, Kyrgyzstan.

F. Triadotypidae (Reisiidae) T2(Anisian)-T3(Carnian)

First: e.g. *Triadotypus guillaumei* in Nel et al. (2001c), Bust outcrop, Bas-Rhin/Moselle, Northern Vosges Mountains, France.

Last: *Reisia sodgianus* in Nel et al. (2001c), Madygen Formation, Madygen/Dzhailoucho, south Fergana Valley, Kyrgyzstan.

F. Triassolestidae (Italophlebiidae, Mesophlebiidae, Progonophlebiidae, Triassoneuridae, Triassothemidae) T3(Carnian)-J1(Toarcian)

First: e.g. *Triassothemis mendozensis* in Martins-Neto et al. (2007b), Potrerillos Formation, Cerro Bayo, Mendoza Province, Argentina.

Last: *Sogdopterites legibile* in Nel et al. (2002), Sagul Formation, Sai-Sagul, Batkenskii District, Kyrgyzstan.

F. Valdicorduliidae Bechly, 1996 K1(Hauterivian)

First and Last: *Valdicordulia wellsorum* Jarzembowski and Nel, 1996, Lower Weald Clay Formation, Clockhouse Brickworks, Surrey, United Kingdom.

F. Xamenophlebiidae T3(Carnian)

First and Last: *Xamenophlebia ornata* in Nel et al. (2001c), Madygen Formation, Madygen/Dzhailoucho, south Fergana Valley, Kyrgyzstan.

F. Zacallitidae Eoc.(Ypresian)

First and Last: *Zacallites balli* in Bechly (1998b), Green River Formation, Unitas area, Colorado, United States.

F. Zygophlebiidae T3(Carnian)

e.g. *Zygophlebiella curta* in Nel et al. (2001c), Madygen Formation, Madygen/Dzhailoucho, south Fergana Valley, Kyrgyzstan.

**O. Protodonata** Brongniart, 1893 (Meganisoptera)
Carboniferous(Bashkirian)-Permian(Wordian)

Considered here as all Odonatoidea not falling within the Nodialata. Paranamurotypidae has not been formally established and so remains *nomen nudum*.

F. Campylopteridae C2(Kasimovian)
Placement is problematic - formerly in Megasecoptera, could now be Protodonata or Odonata.

First and Last: *Campyloptera eatoni* in Nel and Huguet (2002), Upper Coal Measures, Commentry, Allier, France.

F. Erasipteridae C2(Bashkirian)-C2(Moscovian)

First: e.g. *Erasipteroides valentini* in Zessin (2006), Vorhalle Beds, Hagen-Vorhalle, Schmiedestraße, Wuppertal, North Rhine-Westphalia, Germany.

Last: *Erasipterella piesbergensis* in Zessin (2006), Osnabrück Formation, Piesberg quarry, Lower Saxony, Germany.

F. Kohlwaldiidae C2(Moscovian)
Nel et al. (2009b) include *Solutotherates analis* (Moscovian, Allegheny Formation, Pennsylvania, United States) in this family.

e.g. *Kohlwaldia kuehni* in Zessin (2008), Grube Kohlwald, Neunkirchen, Saarland, Germany.

F. Lapeyriidae Nel et al., 1999b(Lapeyridae) P2(Wordian)

First and Last: *Lapeyria magnifica* in Béthoux (2008a), Salagou Formation (Mérifons Member), Lodève Basin, Hérault, France.

F. Meganeuridae C2(Bashkirian)-P2(Wordian)

First: e.g. *Sinomeganeura huangheensis* Ren et al., 2008, Tupo Formation, Qilianshan Mountains, Ningxia/Gansu/Inner Mongolia, China.

Last: e.g. *Permotupus minor* Nel et al., 2009b, Salagou Formation (Mérifons Member), Lodève Basin, Hérault, France.

F. Namurotypidae Bechly, 1996 C2(Bashkirian)

First and Last: *Namurotypus sippeli* in Zessin (2006), Vorhalle Beds, Hagen-Vorhalle, Schmiedestraße, Wuppertal, North Rhine-Westphalia, Germany.

F. Paralogidae C2(Moscovian)-P1(Artinskian)
The specimen listed in Sukatsheva and Rasnitsyn (2004) from the Sai Sagul locality (Sagul Formation) under Paralogidae as *Oligotypus relictus* is probably *Liadotypus relictus*, type of Liadotypidae. '*Oligotypus britannicus*' (nomen nudum) was transfered to Meganeuridae by Nel et al. (2009b).

First: *Oligotypus makowskii* in Nel et al. (2009b), Carbondale Formation, Mazon Creek, Illinois, United States. (Nel et al. 2009b state that the attribution of this species to Paralogidae is questionable and needs revision.)

Last: e.g. *Oligotypus tillyardi* in Rehn (2003), Wellington Formation, Elmo site, Dickinson County, Kansas, United States.

### Neoptera

**O. Miomoptera** Martynov, 1927 Carboniferous(Moscovian)-Jurassic(Sinemurian)

F. Metropatoridae C2(Moscovian)
This family was moved to Caloneurodea by Rasnitsyn (2002g) however this placement was rejected by Béthoux et al. (2004c), thus this family is traditionally retained in Miomoptera following Grimaldi and Engel (2005).

First and Last: *Metropator pusillus* in Rasnitsyn (2003), Allegheny Formation, Pennsylvania/Maryland/West Virginia, Ridge-and-Valley Appalachians, United States.

F. Uninervidae P3(Wuchiapingian)-J1(Sinemurian)

First: e.g. *Redactineura acuminata* in van Dijk and Geertsema (1999), Normandien (Estcourt) Formation, Beaufort Group, KwaZulu-Natal, Karoo Basin, South Africa.

Last: *Mononeura angustipennis* in Rohdendorf (1991), Dzhil Formation, Sogyuty, Issyk-Kul, Kyrgyzstan.

**O. Paoliida** Handlirsch, 1906 (Protoptera)
Carboniferous(Bashkirian)-Carboniferous(Bashkirian)

F. Katerinkidae Prokop and Nel, 2007 C2(Bashkirian)

First and Last: *Katerinka hilaris* Prokop and Nel, 2007, Suchá Beds, Karviná Formation, Upper Silesian Basin, Moravia, Czech Republic.

F. Paoliidae C2(Bashkirian)

e.g. *Mertovia sustai* in Prokop and Nel (2007), Suchá Beds, Karviná Formation, Upper Silesian Basin, Moravia, Czech Republic.

### Polyneoptera

**O. Blattodea** *sensu lato* Brunner von Wattenwyl, 1882 (Blattaria, Blattariae, Blattida, Blattidae, Blattoidea) Carboniferous(Bashkirian)-Quaternary(Holocene)

F. Archimylacridae (Archimylacrididae) C2(Bashkirian)-T3(Carnian)
*Kisylblatta unifasciata* from the Jurassic of Kyzyl-Kiya is Phyloblattidae and not Archimylacridae, according to Vršanský (2003a).

First: e.g. *Miroblattites costalis* in Özdikmen (2008b), passage beds, Rieu du Coeur, Wallonia, Hainaut Province, Belgium.

Last: Mentioned in Shcherbakov (2008b), Madygen Formation, Madygen/Dzhailoucho, south Fergana Valley, Kyrgyzstan. (The identification of Archimylacridae from the Madygen Formation is tentative.)

F. Argentinoblattidae Martins-Neto & Gallego *in* Martins-Neto et al., 2005 T2(Ladinian)
Martins-Neto et al. (2005) list several genera from the Middle Triassic of France and Lower Jurassic of England and Russia which may belong to this family but do not formally attribute them to it.

e.g. *Argentinoblatta herbsti* Martins-Neto & Gallego *in* Martins-Neto et al., 2005, Los Rastros Formation, Bermejo Basin, La Rioja Province, Argentina.

F. Blaberidae (Perisphaeriidae) Eoc.(Ypresian)-Holocene

First: e.g. *Hongoblatta orientalis* in Özdikmen (2008b), Fushun amber, Guchengzi, Liaoning Province, China.

F. Blattidae (Blattoidae) K1(Aptian)-Holocene
Liang et al. (2006) list *Zhujiblatta* Lin, 1980 as Triassic in age. This is likely a mistake as *Zhujiblatta* is from the Chaochuan Formation (Lin, 1994), which is Albian in age (Li et al., 2009).

First: e.g. *Mesoblattinopsis schneideri* in Bechly (2007c), Crato Formation, Araripe Basin, Ceará, Brazil.

F. Blattinopsidae (Blattinopseidae) C2(Kasimovian)-P1(Kungurian)
Béthoux et al. (2009) consider this family to be stem-Dictyoptera and, *contra* Hörnschemeyer and Stapf (2001), do not include *Protoblattinopsis stubblefieldi*. Rasnitsyn (2002c) does not consider *Glaphyrokoris mirandus* from the Moscovian Carbondale Formation (Mazon Creek) to be in this family.

First: e.g. *Blattinopsis* spp. in Béthoux and Nel (2002b), Upper Coal Measures, Commentry, Allier, France.

Last: *Glaphyrophlebia subcostalis* in Rasnitsyn et al. (2005), Inta Formation, Vorkuta Group, Pechora Cola Basin, Komi Republic, Russian Federation.

F. Blattulidae (Blattullidae) T2(Ladinian)-K2(Campanian)

First: *Argentinoblattula revelata* Martins-Neto et al., 2005, Los Rastros Formation, Bermejo Basin, La Rioja Province, Argentina.

Last: *Xonpepetla rinconensis* Cifuentes-Ruiz & Vršanský *in* Cifuentes-Ruiz et al., 2006, Cerro del Pueblo Formation, Rincón Colorado, Coahuila, Mexico.

F. Cainoblattinidae Eoc.(Ypresian)

First and Last: *Cainoblattinopsis fushunensis* in Liang et al. (2006), Fushun amber, Guchengzi, Liaoning Province, China.

F. Caloblattinidae Vršanský & Ansorge *in* Vršanský, 2000 T2(Anisian)-K2(Cenomanian)
Vršanský and Ansorge (2007, p.109) mention that the "latest known representatives are from the Late Cretaceous of Siberia (unpublished material)" and give no further details.

First: Mentioned in Vršanský et al. (2002), Grès à Voltzia, Bas-Rhin/Moselle, Northern Vosges Mountains, France.

Last: e.g. Mentioned in Vršanský et al. (2002), Obluchye tuffaceous mudstones, Jewish Autonomous Oblast, Far Eastern Federal District, Russian Federation.

F. Corydiidae (Euthyrrhaphidae, Holocompsidae, Homoeogamiidae, Poliphagidae, Polyphagidae, Vitismidae) K1(Berriasian)-Holocene

First: Figured in Vršanský and Ansorge (2001), Durlston Formation (Stair Hole Member), Durlston Bay, Dorset, United Kingdom.

F. Cratovitismidae Bechly, 2007c K1(Aptian)

First and Last: *Cratovitisma oldreadi* Bechly, 2007c, Crato Formation, Araripe Basin, Ceará, Brazil.

F. Delpuenteblattidae Martins-Neto et al., 2007b T2(Ladinian)-T3(Carnian)

First: *Lariojablatta chanarensis* in Martins-Neto et al. (2007b), Los Rastros Formation, Bermejo Basin, La Rioja Province, Argentina.

Last: e.g. *Delpuenteblatta dangeloi* Martins-Neto et al., 2007b, Potrerillos Formation, Cerro Bayo, Mendoza Province, Argentina.

F. Diechoblattinidae (Diechnoblattinidae) P1(Asselian)-K1(Berriasian)
Vršanský et al. (2002) synonymised Diechoblattinidae under Poroblattinidae without discussion. They also state that "Poroblattinidae probably failed to cross the Perm-Triassic boundary" (p. 266), yet show the family extending into the Upper Triassic in their range chart for the order, yet the type species of Diechoblattinidae is from the Cretaceous. To avoid further confusion, Diechoblattinidae is kept separate here.

First: e.g. *Nepioblatta intermedia* in Handlirsch (1937), Pony Springs Member, Maroon Formation, Fairplay, Colorado, United States.

Last: e.g. *Deichoblattina wallaci* in Clifford et al. (1994), Lower Purbeck Beds, Durlston Bay, Dorset, United Kingdom.

F. Eadiidae Vršanský, 2009 K1(Albian)
Vršanský (2009) tentatively placed *Raphidiomimula* from the Burmese amber in this family, however it was placed in Caloblattinidae by Liang et al. (2009).

First and Last: *Eadia aidae* Vršanský, 2009, Archingeay amber, Archingeay-Les Nouillers, Charente-Maritime, France.

F. Ectobiidae (Anaplectidae, Blatellidae, Blattellidae, Nyctiboridae, Phyllodromiidae) K1(Berriasian)-Holocene

First: e.g. *Rithma westwoodi* in Ross (2001), Lulworth Formation, Durlston Bay, Dorset, United Kingdom.

F. Fuziidae Vršanský et al., 2009 T3(Carnian)-J3(Oxfordian)

First: Mentioned in Vršanský et al. (2009), Madygen Formation, Madygen/Dzhailoucho, south Fergana Valley, Kyrgyzstan.

Last: Mentioned in Vršanský et al. (2009), Karabastau Formation, Karatau Range, Tien Shan mountains, Kazakhstan.

F. Latiblattidae J3(Oxfordian)

First and Last: *Latiblatta lativalvata* in Özdikmen (2008b), Karabastau Formation, Karatau Range, Tien Shan mountains, Kazakhstan.

F. Liberiblattinidae Vršanský, 2002b J3(Oxfordian)-K1(Albian)

First: e.g. *Liberiblattina ihringovae* Vršanský, 2002b, Karabastau Formation, Karatau Range, Tien Shan mountains, Kazakhstan.

Last: *Leptolythica vincenti* Vršanský, 2009, Archingeay amber, Archingeay-Les Nouillers, Charente-Maritime, France.

F. Mancusoblattidae Martins-Neto & Gallego *in* Martins-Neto et al., 2005 T2(Ladinian)
Martins-Neto et al. (2005) list several genera from the Triassic of France and Japan and Lower Jurassic of Russia (Irkutsk Oblast) which may belong to this family but do not formally attribute them to it.

e.g. *Mancusoblatta pulchella* Martins-Neto & Gallego *in* Martins-Neto et al., 2005, Los Rastros Formation, Bermejo Basin, La Rioja Province, Argentina.

F. Mesoblattinidae J1(Toarcian)-K2(Santonian)
Most previously included taxa were rejected from this family by Vršanský and Ansorge (2007).

First: e.g. *Mesoblattina protypa* in Vršanský and Ansorge (2007), Upper Lias, Dobbertin, Mecklenburg-Vorpommern, Germany.

Last: Mentioned in Vršanský (2008b), Yantardakh amber, Kheta Formation, Taimyr, Krasnoyarsk Krai, Siberian Federal District, Russian Federation.

F. Mylacridae (Archoblattinidae, Mylacrididae, Neorthroblattinidae, Opsiomylacridae) C2(Moscovian)-T3(Carnian)
Vršanský et al. (2002) synonymised Archoblattinidae under Mylacridae without discussion.

First: e.g. *Sooblatta* cf. *deanensis* in Jarzembowski and Schneider (2007), Farrington Formation, Writhlington, Somerset, United Kingdom.

Last: *Austromylacrites latus* in Jell (2004), Blackstone Formation, Ipswich Basin, Queensland, Australia. (This appears to be a plant fossil, which would make the last occurrence of this family *Cathayiblatta longata* Li et al., 2007 from the Ladinian Tongchuan Formation.)

F. Necymylacridae C2(Bashkirian)-C2(Gzhelian)
Vršanský et al. (2002) state that this family extended into the Lower Permian but provide no data on specimens.

First: e.g. *Necymylacris fascigera* in Schneider (1983), Pottsville Formation, Campbell Ledge, Pittston, Pennsylvania, United States.

Last: e.g.? *Necymylacris scudderi* in Schneider (1983), Lawrence Formation, Douglas County, Kansas, United States.

F. Paucineuridae Hong, 1980a P1(Asselian)
While Liang et al. (2006) list this monotypic family as having an Upper Carboniferous age (as per the original description in Hong 1980a), Zhang et al. (1997) showed the Shanxi Formation to be of lowermost Permian age - a view repeated by Hong (1998a).

First and Last: *Paucineura hsui* in Liang et al. (2006), Shanxi Formation (Xiangning Entomassemblage), Xiangning Region, Shanxi Province, China.

F. Phyloblattidae (Anthracoblattinidae) C2(Moscovian)-K1(Barremian)

First: e.g. *Phyloblatta*? sp. in Jarzembowski and Schneider (2007), Farrington Formation, Writhlington, Somerset, United Kingdom.

Last: Figured in Vršanský (2008c), Bon-Tsagaan Nuur, Bon-Tsagaan Group, Bayankhongor Aimag, Mongolia.

F. Poroblattinidae C2(Moscovian)-T3(Carnian)
Schneider et al. (2004) do not consider previous Mesozoic records to belong to this family. Vršanský et al. (2002) also express reservations about the affinities of Mesozoic records, stating that "Poroblattinidae probably failed to cross the Perm-Triassic boundary" (p. 266), yet show the family extending into the Upper Triassic in their range chart for the order.

First: *Poroblatta duffieuxi* in Schneider (1984), Assise de Bruay, Lens, Pas-de-Calais, France.

Last: Mentioned in Shcherbakov (2008b), Madygen Formation, Madygen/Dzhailoucho, south Fergana Valley, Kyrgyzstan.

F. Raphidiomimidae J1(Toarcian)-K1(Aptian)

First: e.g. *Liadoblattina blakei* in Vršanský and Ansorge (2007), Upper Lias, Alderton, Gloucestershire, United Kingdom.

Last: Mentioned in Bechly (2007c), Crato Formation, Araripe Basin, Ceará, Brazil.

F. Skokidae Vršanský, 2007 J3(Oxfordian)

First and Last: *Skok svaba* Vršanský, 2007, Karabastau Formation, Karatau Range, Tien Shan mountains, Kazakhstan.

F. Spiloblattinidae (Compsoblattidae, Compsoblattinidae, Spiloblattidae) C2(Moscovian)-T3(Carnian)
Vršanský et al. (2002) synonymised Compsoblattinidae under Spiloblattinidae without discussion.

First: "*Kinklidoblatta*" *morini* in Schneider and Werneburg (2006), Assise de Bruay, Lens, Pas-de-Calais, France. (Schneider and Werneburg 2006 are uncertain as to the spiloblattinid identity of this species and state that the earliest undoubted spiloblattinids are of Stephanian A (Kasimovian) age.)

Last: Mentioned in Shcherbakov (2008b), Madygen Formation, Madygen/Dzhailoucho, south Fergana Valley, Kyrgyzstan.

F. Subioblattidae T2(Anisian)-T3(Norian)
Papier and Nel (2001) state that this family is known only from the Triassic. Vršanský et al. (2002) indicate this family originates in the Upper Carboniferous but do not give any details. The species from the Sakmarian Letovice Formation at Obora often listed as *Subioblatta* sp. (e.g. in Zajíc and Štamberg, 2004) is listed as "*Syscioblatta* n.

sp. Obora" (Spiloblattinidae) by Schneider and Werneburg (2006), although they also suggest that Subioblattidae might be most closely related to *Syscioblatta* and therefore fall within the Spiloblattinidae.

First: *Subioblatta undulata* in Papier and Nel (2001), Grès à Voltzia, Bas-Rhin/Moselle, Northern Vosges Mountains, France.

Last: e.g. *Samaroblattella kenderlykensis* Papier and Nel, 2001, Tologoy Formation, Ak-Kolka River, Kenderlyk, Zaisan District, Kazakhstan.

F. Umenocoleidae K1(Valanginian)-K1(Albian)
Gorokhov (2006) restricted the composition of this family to the genera *Umenocoleus*, *Petropterix*, *Elytropterix* and *Ponopterix*. Vršanský (2008b) lists this family as present in the Turonian New Jersey amber but this is likely to be *Jantaropterix*, which was removed from this family by Gorokhov (2006). In the description of the type species of this family, *Umenocoleus sinuatus* Chen and Tan, 1973, the deposit it was found in was not reported. It may be from the Chijinbao Formation (Wang Bo pers. comm., 2011) but the stage-age of this specimen is not known for certain other than that it is Lower Cretaceous.

First: *Petropterix sibirix* Vršanský, 2003b, Zaza Formation, Baissa, Buryatia, Russian Federation.

Last: Mentioned in Perrichot et al. (2007), Archingeay amber, Archingeay-Les Nouillers, Charente-Maritime, France.

**O. Caloneurodea** Handlirsch, 1937 (Caloneurida, Caloneuroidea)
Carboniferous(Bashkirian)-Permian(Wordian)

Rasnitsyn et al. (2004a) synonymised many of the caloneurodid family names under Caloneuridae. This suggestion was followed by Beckemeyer (2009b) and is followed here. See also a review of the order by Béthoux et al. (2004c).

F. Caloneuridae (Amboneuridae, Anomalogrammatidae, Apsidoneuridae, Eohymenidae, Euthygrammatidae, Paleuthygrammatidae, Permobiellidae, Pleisiogrammatidae, Sthenaroceridae) C2(Moscovian)-P2(Wordian)

First: e.g. *Amboneura closei* in Rasnitsyn et al. (2004a), Allegheny Formation, Pennsylvania/Maryland/West Virginia, Ridge-and-Valley Appalachians, United States.

Last: *Eohymen maculipennis* in Rasnitsyn et al. (2004a), Amanak Formation, Kargala, Belozersky District, Orenburg Region, Russian Federation.

F. Hapalopteridae (Aenigmatodidae, Emphylopteridae, Protokollariidae) C2(Bashkirian)-C2(Gzhelian)
Ordinal placement and synonymies after Rasnitsyn et al. (2004a). *Tshecalculus inaspectus* is here considered in its own family in Grylloblattodea after Aristov (2009a).

First: *Geroneura wilsoni* in Rasnitsyn et al. (2004a), Lancaster Formation, Saint John, New Brunswick, Canada.

Last: e.g. *Carrizarroyo calopterus* Rasnitsyn *in* Rasnitsyn et al., 2004a, Bursum Formation (Red Tanks Member), Carrizo Arroyo, New Mexico, United States.

F. Permostridulidae Béthoux et al., 2003b P2(Wordian)

First and Last: *Permostridulus brongniarti* in Béthoux (2008a), Salagou Formation (Mérifons Member), Lodève Basin, Hérault, France. (Rasnitsyn et al. 2004a did not consider this taxon in their revision so separate family status is maintained here.)

**O. Cnemidolestodea** Handlirsch, 1937 Carboniferous(Moscovian)-Permian(Wordian)

Order reinstated and redefined by Béthoux (2005). Many of these taxa fall within the Ischnoneuroidea *sensu* Martins-Neto et al. (2007a), so this superfamily is considered here to fall within the Cnemidolestodea. Note, however, that this implies that the lobeattid insects belong here also, which has not yet been conclusively demonstrated (e.g. Béthoux, 2008b).

F. Cnemidolestidae C2(Kasimovian)

e.g. *Cnemidolestes woodwardi* in Béthoux and Nel (2005), Upper Coal Measures, Commentry, Allier, France.

F. Ischnoneuridae (Aetophlebiidae) C2(Kasimovian)
The composition and definition of this family is in a state of flux and in need of revision (Béthoux et al., 2003a). It is taken here *sensu* Rasnitsyn (2002j), with the removal of those taxa which have since been assigned to different, natural groups.

e.g. *Ischnoneura oustaleti* in Béthoux and Nel (2005), Upper Coal Measures, Commentry, Allier, France.

F. Proedischiidae (Narkeminidae, Narkemocagurgidae, Proedischidae) C2(Moscovian)-P1(Asselian)

First: e.g. *Narkema taeniatum* in Béthoux (2005), Carbondale Formation, Mazon Creek, Illinois, United States.

Last: e.g. *Paganzophlebia polyclada* Martins-Neto, Gallego & Brauckmann *in* Martins-Neto et al., 2007a, Bajo de Véliz Formation (Pallero Member), Paganzo Basin, Sierra Grande de San Luis, San Luis Province, Argentina.

F. Spanioderidae (Anthraconeuridae) C2(Moscovian)
The monospecific Anthraconeuridae was restored by Béthoux and Nel (2002b) but the type genus was synonymised with *Miamia* by Béthoux (2008b).

e.g. *Miamia bronsoni* in Béthoux (2008b), Carbondale Formation, Mazon Creek, Illinois, United States.

F. Taiophlebiidae Martins-Neto *in* Martins-Neto et al., 2007a C2(Moscovian)

e.g.? *Cacurgulopsis sanguinettiae* in Martins-Neto (2005), Boituva Formation (Ahrensisporites cristatus zone), Praça da Bandeira, Boituva City, São Paulo, Brazil. (This genus was moved to Taiophlebiidae by Martins-Neto et al. 2007a. The precise stratigraphic age of the other members attributed to this family are not currently known, although all are Upper Carboniferous.)

F. Tococladidae P1(Artinskian)-P2(Wordian)
This family was assigned to the Cnemidolestodea by Béthoux (2007c). Rasnitsyn (2002e) synonymized Heteroptilidae and Nugonioneuridae with this family without argument, which was rejected by Béthoux et al. (2003a).

First: e.g. *Tococladus rallus* in Béthoux et al. (2003a), Wellington Formation, Elmo site, Dickinson County, Kansas, United States.

Last: *Tococladus garrici* Béthoux et al., 2003a, Salagou Formation (Mérifons Member), Lodève Basin, Hérault, France.

**O. Dermaptera** de Geer, 1773 Triassic(Carnian)-Quaternary(Holocene)

Due to taxonomic changes, Spongiphoridae (Labiidae) no longer has a fossil record (see Anisolabididae and Semenoviolidae).

F. Anisolabididae K1(Aptian)-Holocene
Engel and Haas (2007) erect the anisolabidid subfamily Cretolabiinae for the genera *Cretolabia* and *Kotejalabis*, both from the Crato Formation, leaving Spongiphoridae without a fossil record.

First: e.g. *Cratoborellia gorbi* Haas, 2007, Crato Formation, Araripe Basin, Ceará, Brazil.

F. Dermapteridae (Sinopalaeodermatidae, Turanoviidae) J2(Callovian)-J3(Oxfordian)

First: e.g. *Sinopalaeodermata neimonggolensis* in Wappler et al. (2005), Jiulongshan Formation, near Daohugou, Ningcheng county, Inner Mongolia, China. (Originally described with *Jurassimedeola orientalis* Zhang, 2002a. Wappler et al. 2005 list these species in Sinopalaeodermatidae but Engel and Haas 2007 place it as a junior synonym of Dermapterinae.)

Last: e.g. *Turanovia incompleta* in Wappler et al. (2005), Karabastau Formation, Karatau Range, Tien Shan mountains, Kazakhstan.

F. Diplatyidae Mio.(Burdigalian)-Holocene

First: *Diplatys* (*Syndiplatys*) *protoflavicollis* in Wappler et al. (2005), Masaragawa Formation, Seki, Sado Island, Japan.

F. Forficulidae Eoc.(Ypresian)-Holocene

First: *Forficula paleocaenica* in Wappler et al. (2005), Fur Formation (Mo Clay), Limfjord/Mors Peninsula/Fur Island, Jutland, Denmark.

F. Labiduridae K1(Aptian)-Holocene

First: e.g. *Caririlabia berghoffi* Haas, 2007, Crato Formation, Araripe Basin, Ceará, Brazil.

F. Ocelliidae Spahr, 1990 Eoc.(Priabonian)
Originally thought to belong in Diplura, this family is considered *nomen dubium* by Engel and Haas (2007) as it is probably a junior synonym of another, as yet unidentified, common Baltic amber earwig family.

First and Last: *Ocellia articulicornis* in Wappler et al. (2005), Baltic amber.

F. Protodiplatyidae (Longicerciatidae, Protodiplateidae, Protodiplatidae) T3(Carnian)-K1(Barremian)

First: Mentioned in Shcherbakov (2008b), Madygen Formation, Madygen/Dzhailoucho, south Fergana Valley, Kyrgyzstan.

Last: e.g. *Longicerciata mesozoica* in Wappler et al. (2005), Laiyang Formation, Laiyang County, Shandong Province, China.

F. Pygidicranidae (Pygidiocranidae) K1(Albian)-Holocene

First: *Burmapygia resinata* Engel and Grimaldi, 2004b, Burmese amber (Burmite), Hukawng Valley, Kachin State, Myanmar. (Engel and Grimaldi 2004b consider this to be the oldest definitive Pygidicranidae.)

F. Semenoviolidae J3(Oxfordian)

e.g. *Semenovioloides capitatus* in Wappler et al. (2005), Karabastau Formation, Karatau Range, Tien Shan mountains, Kazakhstan.

F. Turanodermatidae Engel, 2003b(Turanodermidae) J3(Oxfordian)
This family may extend into the Cretaceous if *Archaeosoma* (Barremian, Laiyang Fm, China) turns out to be allied (Engel, 2003b).

First and Last: *Turanoderma sepultum* in Wappler et al. (2005), Karabastau Formation, Karatau Range, Tien Shan mountains, Kazakhstan.

**O. Embiodea** Kusnezov, 1903 (Embiida, Embiidina, Embioptera)
Jurassic(Callovian)-Quaternary(Holocene)

No Palaeozoic records of this family have been substantiated (Engel and Grimaldi, 2006a). The fossil *Clothonopsis miocenica* from the Miocene of China is a bibionid (Zhang, 1993), leaving the Clothodidae without a fossil record (Engel and Grimaldi, 2006a).

F. Anisembiidae Mio.(Burdigalian)-Holocene

First: e.g. *Glyphembia amberica* Ross, 2003, Dominican amber, Cordillera Septentrional, near Santiago, Dominican Republic.

F. Embiidae Eoc.(Priabonian)-Holocene

First: e.g. *Electroembia antiqua* in Engel and Grimaldi (2006a), Baltic amber.

F. Notoligotomidae (Burmitembiidae) K1(Albian)-Holocene

First: *Burmitembia venosa* in Engel and Grimaldi (2006a), Burmese amber (Burmite), Hukawng Valley, Kachin State, Myanmar.

F. Oligotomidae Pleist.(Upper Pleistocene)-Holocene

First: *Oligotoma westwoodi* in Spahr (1992), Tanzanian copal, Tanzanian copal, Tanzanian copal, Tanzania. (Handlirsch 1908 lists this specimen as from 'Zanzibar?'.)

F. Sinembiidae Huang and Nel, 2009b J2(Callovian)

e.g. *Sinembia rossi* Huang and Nel, 2009b, Jiulongshan Formation, near Daohugou, Ningcheng county, Inner Mongolia, China.

F. Sorellembiidae Engel and Grimaldi, 2006a K1(Albian)

First and Last: *Sorellembia estherae* Engel and Grimaldi, 2006a, Burmese amber (Burmite), Hukawng Valley, Kachin State, Myanmar.

F. Teratembiidae Mio.(Burdigalian)-Holocene

First: *Oligembia vetusta* in Engel and Grimaldi (2006a), Dominican amber, Cordillera Septentrional, near Santiago, Dominican Republic.

**O. Grylloblattodea** Brues and Melander, 1915 (Grylloblattida, Grylloblattoidea)
Carboniferous(Bashkirian)-Quaternary(Holocene)

F. Aliculidae Storozhenko, 1997 P1(Sakmarian)-P2(Wordian)

First: *Alicula acra* in Storozhenko (1997), Obora locality, Bačov Beds, Letovice Formation, Moravia, Czech Republic. (Listed as *Permula aera* by Zajíc and Štamberg 2004, however *Permula* is a junior synonym of *Alicula* by priority and '*aera*' is a misspelling of *acra* made by Kukalová 1964.)

Last: *Tshepanichoptera lacera* Aristov *in* Aristov and Bashkuev, 2008, Chepanikha locality, Rossokha River valley, Zavjalovskii District, Udmurt Republic, Russian Federation.

F. Archiprobnidae (Archiprobnisidae) P2(Roadian)

First and Last: *Archiprobnis repens* in Storozhenko (1997), Kuznetsk Formation (Mitino Horizon), Kaltan, Kemerovo Region, Russian Federation.

F. Atactophlebiidae (Bardapteridae) P1(Kungurian)-P2(Roadian)
*Triaseuryptilon accostai* from the Triassic of Argentina does not belong to this family and may not be a grylloblattid (Aristov, 2004a).

First: e.g. *Kirkorella mira* in Aristov (2004b), Koshelevka Formation, Tshekarda, Ural Mountains, Russian Federation.

Last: e.g. *Atactophlebia termitoides* in Béthoux et al. (2005), Baitugan Formation, Tikhie Gory, Kama River, Tatarstan, Russian Federation.

F. Bajanzhargalanidae Storozhenko 1992 *in* J3(Tithonian)

First and Last: *Bajanzhargalana magna* Storozhenko, 1988, Ulan-Ereg, Khoutiyn-Khotgor, Dund-Gobi Aimag, Mongolia.

F. Blattogryllidae P3(Changhsingian)-K1(Valanginian)
*Blattogryllus karatavicus* from the Oxfordian Karabastau Formation at Karatau (Kazakhstan) is a cockroach (Aristov et al., 2006).

First: e.g. *Protoblattogryllus zajsanicus* Storozhenko, 1990, Maichat/Ak-Kolka Formation, Karaungir River, Saur Mountains, Vostochno-Kazakhstanskaya oblast, Kazakhstan.

Last: *Parablattogryllus obscurus* Storozhenko, 1988, Zaza Formation, Baissa, Buryatia, Russian Federation.

F. Camptoneuritidae (Camptoneuridae) P2(Roadian)

First and Last: *Camptoneurites reticulata* in Storozhenko (1997), Baitugan Formation, Tikhie Gory, Kama River, Tatarstan, Russian Federation.

F. Chauliodititidae (Tomiidae) P2(Roadian)-T2(Anisian)

First: e.g. *Protomia proteus* in Aristov (2008a), Belebey Formation, Kityak, Kirov Region, Russian Federation. (*Protomia* and *Miralioma* were transferred to Chauliodititidae in Aristov et al. 2009a.)

Last: Mentioned in Aristov (2004c), Grès à Voltzia, Bas-Rhin/Moselle, Northern Vosges Mountains, France.

F. Chelopteridae P1(Artinskian)

First and Last: *Chelopterum peregrinum* in Beckemeyer (2004b), Wellington Formation, Elmo site, Dickinson County, Kansas, United States.

F. Daldubidae Storozhenko, 1996b C2(Gzhelian)

e.g. *Dalduba faticana* in Storozhenko (2002), Kata Formation, Chunya, Siberian Federal District, Russian Federation.

F. Demopteridae P1(Artinskian)

First and Last: *Demopterum gracile* Carpenter, 1950, Wellington Formation, Elmo site, Dickinson County, Kansas, United States.

F. Epideigmatidae (Paraphenopteridae, Phenopteridae, Sylvaphlebiidae) C2(Moscovian)-P3(Changhsingian)

First: *Epideigma elegans* in Béthoux (2007b), Carbondale Formation, Mazon Creek, Illinois, United States.

Last: *Belmophenopterum pectinatum* Rasnitsyn and Aristov, 2004, Belmont insect beds, Newcastle Coal Measures, Belmont/Warner's Bay, New South Wales, Australia.

F. Euremiscidae P1(Kungurian)-P2(Roadian)

First: e.g. *Euremisca elegans* Aristov, 2004b, Koshelevka Formation, Tshekarda, Ural Mountains, Russian Federation.

Last: *Euremisca kazanica* Aristov, 2009d, Iva-Gora limestones, Soyana River, Arkhangelsk Region, Ural Mountains, Russian Federation.

F. Euryptilonidae (Stereopteridae) P1(Sakmarian)-P2(Roadian)
*Karaungirella* from Karaungir (Changhsingian) belongs in the miomopteran family Permosialidae (Aristov, 2004a).

First: e.g. *Blania falsa* in Zajíc and Štamberg (2004), Obora locality, Bačov Beds, Letovice Formation, Moravia, Czech Republic. (This genus, along with *Karaungirella*, *Maculopterum*, *Oborella*, *Quercopterum*, *Sharovipterum*, *Torrentopterum* and *Villopterum*, were transferred from Lemmatophoridae to Euryptilonidae by Storozhenko 1997.)

Last: Mentioned in Aristov (2004b), Kuznetsk Formation (Mitino Horizon), Kaltan, Kemerovo Region, Russian Federation.

F. Geinitziidae (Prosepididontidae, Stegopteridae) P1(Kungurian)-J3(Tithonian)

First: *Stegopterum anteanatalis* Aristov, 2004a, Lek-Vorkuta Formation, Vorkuta Group, Pechora Cola Basin, Komi Republic, Russian Federation.

Last: *Shurabia shartegica* Aristov et al., 2009b, Shar-Teg Formation, Shar-Teg Ula, Gobi-Altai Aimag, Mongolia.

F. Gorochoviidae Storozhenko, 1994 T3(Carnian)

e.g. *Gorochovia individua* Storozhenko, 1994, Madygen Formation, Madygen/Dzhailoucho, south Fergana Valley, Kyrgyzstan.

F. Havlatiidae P1(Sakmarian)

e.g. *Havlatia annae* in Zajíc and Štamberg (2004), Obora locality, Bačov Beds, Letovice Formation, Moravia, Czech Republic.

F. Ideliidae P1(Kungurian)-T3(Norian)
The Carboniferous genus *Protoperla* was moved to Grylloblattodea *incertae sedis* in Béthoux et al. (2005).

First: e.g. *Micaidelia minutissima* Aristov, 2004b, Koshelevka Formation, Tshekarda, Ural Mountains, Russian Federation.

Last: *Ideliopsina kenderlykensis* in Aristov (2005), Tologoy Formation, Ak-Kolka River, Kenderlyk, Zaisan District, Kazakhstan.

F. Idelinellidae Storozhenko, 1997 P1(Kungurian)-P2(Roadian)

First: e.g. *Sylvastriga miranda* Aristov, 2004b, Koshelevka Formation, Tshekarda, Ural Mountains, Russian Federation.

Last: *Idelinella macroptera* Storozhenko, 1992c, Iva-Gora limestones, Soyana River, Arkhangelsk Region, Ural Mountains, Russian Federation. (Originally described in Ideliidae.)

F. Ivapteridae Aristov, 2009a P1(Kungurian)-P2(Roadian)

First: *Tshekardembia sharovi* in Aristov and Rasnitsyn (2009), Koshelevka Formation, Tshekarda, Ural Mountains, Russian Federation.

Last: *Ivaptera sharovi* Aristov, 2009a, Iva-Gora limestones, Soyana River, Arkhangelsk Region, Ural Mountains, Russian Federation.

F. Jabloniidae P1(Sakmarian)

First and Last: *Jablonia aestiva* in Zajíc and Štamberg (2004), Obora locality, Bačov Beds, Letovice Formation, Moravia, Czech Republic.

F. Juraperlidae Huang and Nel, 2007a J2(Callovian)

First and Last: *Juraperla daohugouensis* Huang and Nel, 2007a, Jiulongshan Formation, near Daohugou, Ningcheng county, Inner Mongolia, China.

F. Kargalopteridae Aristov, 2009b P2(Wordian)

e.g. *Kargaloptera connexa* Aristov, 2009b, Amanak Formation, Kargala, Belozersky District, Orenburg Region, Russian Federation.

F. Kortshakoliidae Storozhenko, 1997 P1(Kungurian)-P2(Roadian)

First: *Kortshakolia ideliformis* in Storozhenko (1997), Usyatsk Formation, Balakhonsk Series, Korchakol, Kemerovo Region, Russian Federation.

Last: *Paridelia pusilla* in Storozhenko (1997), Kuznetsk Formation (Mitino Horizon), Kaltan, Kemerovo Region, Russian Federation.

F. Liomopteridae (Khosaridae) C2(Gzhelian)-T3(Carnian)

First: e.g. *Tapopterum populus* Aristov *in* Rasnitsyn et al., 2004a, Bursum Formation (Red Tanks Member), Carrizo Arroyo, New Mexico, United States.

Last: Figured in Cairncross et al. (1995), Molteno Formation, KwaZulu-Natal, Karoo Basin, South Africa.

F. Madygenophlebiidae Storozhenko, 1992a T3(Carnian)

e.g. *Madygenophlebia bella* Storozhenko, 1992a, Madygen Formation, Madygen/Dzhailoucho, south Fergana Valley, Kyrgyzstan.

F. Megakhosaridae P1(Artinskian)-T3(Carnian)

First: Mentioned in Aristov (2009d), Petrolia (Belle-Plains) Formation, Wichita Group, Texas, United States.

Last: Mentioned in Aristov (2008b), Madygen Formation, Madygen/Dzhailoucho, south Fergana Valley, Kyrgyzstan.

F. Mesojabloniidae Storozhenko, 1992b T3(Carnian)

First and Last: *Mesojablonia kukalovae* Storozhenko, 1992b, Madygen Formation, Madygen/Dzhailoucho, south Fergana Valley, Kyrgyzstan.

F. Mesorthopteridae T2(Anisian)-T3(Norian)

First: *Austroidelia perplexa* in Jell (2004), Hawkesbury Sandstone, Brookvale Quarry, Beacon Hill, New South Wales, Australia. (Jell 2004 listed this species in Ideliidae but it was transferred to Mesorthopteridae by Storozhenko 1996a.)

Last: Mentioned in Aristov (2005), Tologoy Formation, Ak-Kolka River, Kenderlyk, Zaisan District, Kazakhstan.

F. Neleidae Ansorge, 1996a J1(Toarcian)

First and Last: *Nele jurassica* Ansorge, 1996a, Upper Lias, Grimmen, Mecklenburg-Vorpommern, Germany.

F. Oecanthoperlidae Storozhenko, 1988 K1(Valanginian)

First and Last: *Oecanthoperla sibirica* Storozhenko, 1988, Zaza Formation, Baissa, Buryatia, Russian Federation.

F. Permopectinidae Aristov *in* Rasnitsyn et al., 2005 P1(Kungurian)

e.g. *Permopectina tshekardensis* Aristov *in* Rasnitsyn et al., 2005, Koshelevka Formation, Tshekarda, Ural Mountains, Russian Federation.

F. Permotermopsidae P1(Kungurian)-P3(Changhsingian)

First: e.g. *Khosaridelia rigida* Aristov *in* Rasnitsyn et al., 2005, Lek-Vorkuta Formation, Vorkuta Group, Pechora Cola Basin, Komi Republic, Russian Federation.

Last: *Khosaridelia vyatica* Aristov, 2009d, Maichat/Ak-Kolka Formation, Karaungir River, Saur Mountains, Vostochno-Kazakhstanskaya oblast, Kazakhstan.

F. Pinideliidae Storozhenko, 1997 P1(Kungurian)

e.g. *Kishertia tricubitalis* in Aristov (2004b), Koshelevka Formation (Iren' Horizon), Kishert' locality, Ural Mountains, Russian Federation.

F. Plesioblattogryllidae Huang et al., 2008b J2(Callovian)

First and Last: *Plesioblattogryllus magnificus* Huang et al., 2008b, Jiulongshan Formation, near Daohugou, Ningcheng county, Inner Mongolia, China.

F. Probnidae (Probnisidae) C2(Gzhelian)-T3(Norian)

First: *Probnis fossor* Aristov *in* Rasnitsyn et al., 2004a, Bursum Formation (Red Tanks Member), Carrizo Arroyo, New Mexico, United States.

Last: *Triassoprobnis humilis* in Aristov (2005), Protopivka Formation, Garazhovka, Izyum District, Ukraine.

F. Protembiidae P1(Artinskian)

First and Last: *Protembia permiana* in Storozhenko (1997), Wellington Formation, Elmo site, Dickinson County, Kansas, United States.

F. Protoblattinidae (Protoblattidae) C2(Kasimovian)
*Protoblattina* brought out of synonymy from *Protoperla* in Béthoux et al. (2005).

First and Last: *Protoblattina bouvieri* in Béthoux et al. (2005), Upper Coal Measures, Commentry, Allier, France.

F. Protoperlidae C2(Kasimovian)

First and Last: *Protoperla westwoodi* in Béthoux et al. (2005), Upper Coal Measures, Commentry, Allier, France.

F. Raaschiidae Beckemeyer, 2004b P1(Artinskian)

First and Last: *Raaschia oklahomensis* Beckemeyer, 2004b, Wellington Formation, Midco, Oklahoma, United States.

F. Sinonamuropteridae Peng et al., 2005 C2(Bashkirian)
Originally described in Diaphanopterodea, this family was referred to the Grylloblattodea by Prokop and Ren (2007).

e.g. *Separatonerva qilianshanensis* Peng et al., 2005, Tupo Formation, Qilianshan Mountains, Ningxia/Gansu/Inner Mongolia, China.

F. Skaliciidae (Scalicidae, Skalicidae) P1(Sakmarian)-P2(Wordian)

First: e.g. *Skalicia rara* in Aristov (2009d), Obora locality, Bačov Beds, Letovice Formation, Moravia, Czech Republic.

Last: *Urzhumskalicia kargalensis* Aristov, 2009b, Amanak Formation, Kargala, Belozersky District, Orenburg Region, Russian Federation.

F. Sojanoraphidiidae P1(Artinskian)-P2(Roadian)

First: *Aibolitus minutus* Béthoux and Beckemeyer, 2007, Wellington Formation, Elmo site, Dickinson County, Kansas, United States. (Béthoux and Beckemeyer 2007 consider the family placement of this species as uncertain but Aristov 2009d lists it in this family.)

Last: *Sojanoraphidia rossica* in Storozhenko and Novokshonov (1994), Iva-Gora limestones, Soyana River, Arkhangelsk Region, Ural Mountains, Russian Federation.

F. Stenoneuritidae C2(Kasimovian)

First and Last: *Stenoneurites maximi* in Béthoux et al. (2005), Upper Coal Measures, Commentry, Allier, France.

F. Sylvabestiidae Aristov, 2000a P1(Kungurian)

First and Last: *Sylvabestia tenuis* Aristov, 2000a, Koshelevka Formation, Tshekarda, Ural Mountains, Russian Federation.

F. Sylvardembiidae Novokshonov, 2000 P1(Kungurian)-P2(Roadian)

First: e.g. *Sylvardembia matura* Aristov, 2000b, Koshelevka Formation, Tshekarda, Ural Mountains, Russian Federation.

Last: *Barmaleus* sp. in Aristov and Rasnitsyn (2009), Iva-Gora limestones, Soyana River, Arkhangelsk Region, Ural Mountains, Russian Federation.

F. Tillyardembiidae P1(Kungurian)

e.g. *Kunguremb ia brevicervix* in Aristov and Rasnitsyn (2009), Koshelevka Formation, Tshekarda, Ural Mountains, Russian Federation.

F. Tshecalculidae Novokshonov, 2000 P1(Kungurian)
Originally unplaced in Pterygota, Aristov (2009a) lists this family in the Grylloblattodea.

First and Last: *Tshecalculus inaspectus* Novokshonov, 2000, Koshelevka Formation, Tshekarda, Ural Mountains, Russian Federation. (Rasnitsyn et al. 2004a list this species in the Caloneurodea: Hapalopteridae but this reference is superceeded by Aristov 2009a.)

F. Tshekardominidae Novokshonov and Aristov, 2002 P1(Artinskian)-P2(Capitanian)

First: *Sigmophlebia engeli* in Aristov (2009d), Wellington Formation, Midco, Oklahoma, United States.

Last: *Tshekardomina mongolica* Aristov, 2009d, Tsankhi (Tsankhin) Formation, Bor-Tolgoy, Ömnögovi (South Gobi) Aimag, Mongolia.

F. Tunguskapteridae Storozhenko and Vršanský, 1995 T1(Induan)-T3(Carnian)

First: *Tunguskaptera eximia* Storozhenko and Vršanský, 1995, Bugarikhta Formation, Nizhnyaya Tunguska river, Krasnoyarsk Krai, Siberian Federal District, Russian Federation.

Last: *Ferganamadygenia plicata* Storozhenko and Vršanský, 1995, Madygen Formation, Madygen/Dzhailoucho, south Fergana Valley, Kyrgyzstan.

**O. Isoptera** Brullé, 1832 (Termitida, Termitoidae)
Cretaceous(Valanginian)-Quaternary(Holocene)

First: *Baissatermes lapideus* Engel et al., 2007a, Zaza Formation, Baissa, Buryatia, Russian Federation. A growing body of ichnological literature (e.g. Bordy et al. 2009 and references within) suggest the presence of termites in the Lower Jurassic and perhaps Upper Triassic. The classification of Engel et al. (2009a) is followed here. Therefore, Hodotermitidae is considered not to have a fossil record. Although Bechly (2007d) and earlier authors assigned termites from the Crato Formation to extant families, Grimaldi et al. (2008) consider them *incertae sedis* and Engel et al. (2009a) placed them elsewhere.

F. Archeorhinotermitidae Krishna and Grimaldi, 2003 K1(Albian)
Originally described as a subfamily of Rhinotermitidae but elevated to family in Engel et al. (2009a).

First and Last: *Archeorhinotermes rossi* in Engel et al. (2009a), Burmese amber (Burmite), Hukawng Valley, Kachin State, Myanmar.

F. Archotermopsidae Engel et al., 2009a Eoc.(Priabonian)-Holocene

First: e.g. *Archotermopsis tornquisti* in Engel et al. (2009a), Baltic amber.

F. Cratomastotermitidae Engel et al., 2009a K1(Aptian)

First and Last: *Cratomastotermes wolfschwenningeri* in Engel et al. (2009a), Crato Formation, Araripe Basin, Ceará, Brazil.

F. Kalotermitidae (Calotermitidae) K1(Albian)-Holocene
The Jordanian amber record figured in Kaddumi (2005) is doubtful.

First: e.g. *Kalotermes burmensis* Poinar, 2009a, Burmese amber (Burmite), Hukawng Valley, Kachin State, Myanmar.

F. Mastotermitidae K1(Hauterivian)-Holocene

First: *Valditermes brenanae* in Engel et al. (2009a), Lower Weald Clay Formation, Capel, Surrey, United Kingdom.

F. Rhinotermitidae Eoc.(Priabonian)-Holocene

First: e.g. *Heterotermes eocenicus* in Engel et al. (2009a), Baltic amber.

F. Stylotermitidae Eoc.(Priabonian)-Holocene

First: *Parastylotermes robustus* in Engel et al. (2009a), Baltic amber.

F. Termitidae Olig.(Rupelian)-Holocene

First: *Aiuruocatatermes piovezanae* Martins-Neto and Pesenti, 2006, Entre-Córregos Formation, Aiuruoca Basin, Minas Gerais, Brazil.

F. Termopsidae Eoc.(Priabonian)-Mio.(Serravallian)
Engel et al. (2009a) restrict the composition of this family to the type genus *Termopsis*.

First: e.g. *Termopsis ukapirmasi* in Engel et al. (2009a), Baltic amber.

Last: e.g. *Termopsis mallaszi* in Engel et al. (2007b), ”volcanic floras" deposit, Tállya, Eperges-Tokajer Mountains, Hungary.

**O. Mantodea** Burmeister, 1839 (Manteodea, Mantida)
Carboniferous(Kasimovian)-Quaternary(Holocene)

Note that recent molecular studies have shown that the current classification of mantids is not congruent with phylogeny (Ware et al., 2008; Svenson and Whiting, 2009). Grimaldi (2003b) revised the taxonomy of all Cretaceous mantid fossils, leaving no extant family with a Mesozoic fossil record, although some authors disagree with his interpretations (see below).

F. Ambermantidae Grimaldi, 2003b K2(Turonian)

First and Last: *Ambermantis wozniaki* Grimaldi, 2003b, New Jersey amber, South Amboy Fire Clay (Raritan Formation), New Jersey, United States. (Vršanský 2008a mistakenly states that this species is a junior synonym of *Jantarimantis zherikhini*.)

F. Baissomantidae Gratshev and Zherikhin, 1994 K1(Valanginian)

e.g. *Baissomantis picta* in Grimaldi (2003b), Zaza Formation, Baissa, Buryatia, Russian Federation.

F. Chaeteessidae (Archephemeridae, Chaeteessiidae) K1(Valanginian)-Holocene

First: *Cretophotina selenginensis* in Vršanský (2008c), Sharin-Gol Formation, Sharin-Gol, Selenge Aimag, Mongolia.

F. Cretomantidae Gratshev and Zherikhin, 1994 K1(Valanginian)
Grimaldi (2003b) removes *Electromantis* (Santonian amber from the Kheta Formation, Russia) to Mantodea *incertae sedis*, although he does not explicitly mention the position of *Cretomantis* in his revised system.

First and Last: *Cretomantis larvalis* in Grimaldi (2003b), Zaza Formation, Baissa, Buryatia, Russian Federation.

F. Hymenopodidae Eoc.(Ypresian)-Holocene

First: Figured in Zherikhin (2002b), Green River Formation, Unitas area, Colorado, United States. (Zherikhin's assignment of this specimen to Hymenopodidae was tentative.)

F. Jantarimantidae Vršanský, 2002a(Archimantidae) K2(Turonian)
Originally described as Archimantidae in Vršanský (2002b) but a replacement name was later given as this was a junior homonym. [NOTE: Need to find a way to change a and b around for Vrsansky 2002 refs.]

First and Last: *Jantarimantis zherichini* in Gorokhov (2006), New Jersey amber, South Amboy Fire Clay (Raritan Formation), New Jersey, United States.

F. Juramantidae Vršanský, 2002b J3(Tithonian)

First and Last: *Juramantis initialis* in Vršanský (2005), Shar-Teg Formation, Shar-Teg Ula, Gobi-Altai Aimag, Mongolia.

F. Liturgusidae Eoc.(Priabonian)-Holocene

First: Mentioned in Weitschat and Wichard (2002), Baltic amber.

F. Mantidae (Manteidae, Vatidae) Pal.(Thanetian)-Holocene

First: *Prochaeradodis enigmaticus* in Nel and Roy (1996), spongo-diatomaceous maar, Menat, Puy-de-Dôme, Auvergne, France.

F. Mantoididae Eoc.(Priabonian)-Holocene

First: *Mantoida matthiasglinki* Zompro, 2005, Baltic amber.

F. Santanmantidae Grimaldi, 2003b K1(Aptian)

First and Last: *Santanmantis axelrodi* in Grimaldi (2007), Crato Formation, Araripe Basin, Ceará, Brazil.

F. Strephocladidae (Strephoneuridae) C2(Kasimovian)-P2(Roadian)
Rasnitsyn and Aristov (2004) synonymise Strephocladidae and Strephoneuridae under Anthracoptilidae but the attribution to the total-group Mantodea of the 'strephocladidaeans' *sensu* Béthoux and Wieland (2009) (including *Mesoptilus* and *Strephoneura*) apart from the other anthracoptilid genera warrants listing the family group here.

First: e.g. *Mesoptilus dolloi* in Béthoux and Wieland (2009), Upper Coal Measures, Commentry, Allier, France.

Last: e.g. *Graticladus severus* in Béthoux and Wieland (2009), Iva-Gora limestones, Soyana River, Arkhangelsk Region, Ural Mountains, Russian Federation.

F. Tarachodidae Mio.(Burdigalian)-Holocene

First: Mentioned in Zherikhin (2002b), Dominican amber, Cordillera Septentrional, near Santiago, Dominican Republic.

**O. Mantophasmatodea** Klass et al., 2002 Jurassic(Callovian)-Quaternary(Holocene)

There are disagreements in the literature as to the rank of family-groups and the placement of fossils. Arillo and Engel (2006) have been followed here but see also Damgaard et al. (2008) for a counterview.

F. Mantophasmatidae Zompro, Klass, Kristensen & Adis *in* Klass et al., 2002(Austrophasmatidae, Ensiferophasmatidae, Raptophasmatidae, Tanzaniophasmatidae) J2(Callovian)-Holocene

First: *Juramantophasma sinica* Huang et al., 2008c, Jiulongshan Formation, near Daohugou, Ningcheng county, Inner Mongolia, China.

**O. Orthoptera** Olivier, 1789 (Gryllida, Titanoptera)
Carboniferous(Kasimovian)-Quaternary(Holocene)

Taxonomic system as that of the Orthoptera Species File Version 2.0/3.5, accessed at `http://orthoptera.speciesfile.org`. Archaeopneumoridae (Crato Formation, Brazil) must still be considered nomen nudum (Heads and Martins-Neto, 2007), so is not listed here.

F. Acrididae (Oedipodidae, Truxalidae) Eoc.(Ypresian)-Holocene

First: e.g. Mentioned in Selden and Penney (2009), Horsefly shales, Horsefly river, Cariboo, British Columbia, Canada.

F. Adumbratomorphidae Gorokhov, 1987a P1(Kungurian)

First and Last: *Adumbratomorpha tettigonioides* in Gorokhov (1995b), Koshelevka Formation, Tshekarda, Ural Mountains, Russian Federation.

F. Anelcanidae (Parelcanidae) P1(Artinskian)

e.g. *Anelcana dilatata* in Beckemeyer (2000), Wellington Formation, Elmo site, Dickinson County, Kansas, United States.

F. Anostostomatidae (Henicidae, Mimnermidae) K1(Aptian)-Holocene

First: *Euclydes ramosfernandesi* Martins-Neto, 2007, Crato Formation, Araripe Basin, Ceará, Brazil.

F. Araripelocustidae Martins-Neto, 1995a(Araripelocustopsidae) K1(Aptian)

e.g. *Araripelocusta brevis* in Heads and Martins-Neto (2007), Crato Formation, Araripe Basin, Ceará, Brazil.

F. Baissogryllidae Gorokhov, 1985(Cearagryllidae) J3(Tithonian)-K1(Aptian)

First: e.g. *Sharategia rasnitsyni* in Gorokhov et al. (2006), Shar-Teg Formation, Shar-Teg Ula, Gobi-Altai Aimag, Mongolia.

Last: e.g. *Notocearagryllus arturandradai* Martins-Neto *in* Martins-Neto and Tassi, 2009, Crato Formation, Araripe Basin, Ceará, Brazil.

F. Bintoniellidae T3(Carnian)-J1(Hettangian)

First: e.g. *Oshiellana primaria* in Gorokhov (2005a), Madygen Formation, Madygen/Dzhailoucho, south Fergana Valley, Kyrgyzstan.

Last: *Bintoniella brodiei* in Shcherbakov (2008a), Lower Lias, Binton, Warwickshire, United Kingdom.

F. Bouretidae Martins-Neto, 2001 K1(Aptian)

First and Last: *Bouretia elegans* in Heads and Martins-Neto (2007), Crato Formation, Araripe Basin, Ceará, Brazil.

F. Brauckmanniidae Martins-Neto, 2007 K1(Aptian)

First and Last: *Brauckmannia groeningae* Martins-Neto, 2007, Crato Formation, Araripe Basin, Ceará, Brazil.

F. Chorotypidae (Eruciidae) Eoc.(Priabonian)-Holocene

First: *Erucius? lewisi* in Martins-Neto (2003), Passamari Formation, Ruby River Basin, Montana, United States. (This species was not mentioned by Carpenter 1992b. This extant genus is listed under the Chorotypidae in the Orthoptera Species File.)

F. Dzhajloutshellidae Gorokhov, 1994 T3(Carnian)

e.g. *Dzhajloutshella flexuosa* Gorokhov, 2005b, Madygen Formation, Madygen/Dzhailoucho, south Fergana Valley, Kyrgyzstan.

F. Elcanidae T2(Anisian)-K1(Albian)

First: *Elcanopsis sydneiensis* in Jell (2004), Hawkesbury Sandstone, Brookvale Quarry, Beacon Hill, New South Wales, Australia. (This species is not mentioned in the Orthoptera Species File (Version 2.0/4.0).)

Last: e.g. *Longioculus burmensis* Poinar et al., 2007, Burmese amber (Burmite), Hukawng Valley, Kachin State, Myanmar.

F. Episactidae Mio.(Burdigalian)-Holocene

First: *Paleomastacris ambarinus* in Pérez-Gelabert and Rowell (2006), Dominican amber, Cordillera Septentrional, near Santiago, Dominican Republic.

F. Eumastacidae J3(Oxfordian)-Holocene

First: *Archaeomastax jurassicus* in Pérez et al. (1997), Karabastau Formation, Karatau Range, Tien Shan mountains, Kazakhstan. (Heads 2008a mistakenly lists this specimen as Lower Jurassic.)

F. Gryllacrididae (Gryllacridae) T3(Carnian)-Holocene

First: *Xenogryllacris reductus* in Jell (2004), Mount Crosby Formation, Ipswich Basin, Queensland, Australia.

F. Gryllavidae Gorokhov, 1986 T2(Anisian)-T3(Carnian)

First: *Galliagryllavus vogesiacus* Marchal-Papier et al., 2000, Grès à Voltzia, Bas-Rhin/Moselle, Northern Vosges Mountains, France.

Last: e.g. *Zagryllavus elongatus* in Gorokhov (2005a), Madygen Formation, Madygen/Dzhailoucho, south Fergana Valley, Kyrgyzstan.

F. Gryllidae (Eneopteridae, Oecanthidae, Trigonidiidae) K1(Hauterivian)-Holocene

First: *Araripegryllus*? *orientalis* Gorokhov et al., 2006, Lower Weald Clay Formation, Clockhouse Brickworks, Surrey, United Kingdom.

F. Gryllotalpidae K1(Aptian)-Holocene

First: e.g. *Archaeogryllotalpoides ornatus* in Heads and Martins-Neto (2007), Crato Formation, Araripe Basin, Ceará, Brazil.

F. Haglidae (Isfaropteridae) T2(Anisian)-K1(Barremian)
The extant genus *Cyphoderris* is considered here to be in the Prophalangopsidae, following the Orthoptera Species File.

First: *Prohagla superba* in Jell (2004), Hawkesbury Sandstone, Brookvale Quarry, Beacon Hill, New South Wales, Australia.

Last: Mentioned in Peñalver et al. (1999), Montsec lithographic limestones, Montsec Range, Lleida Province, Spain.

F. Hagloedischiidae Gorokhov, 1986 T2(Anisian)-T3(Carnian)

First: *Voltziahagla pseudoveinosa* Marchal-Papier et al., 2000, Grès à Voltzia, Bas-Rhin/Moselle, Northern Vosges Mountains, France. (Originally described in Haglidae but transferred to Hagloedischiidae by Gorokhov 2005a.)

Last: *Hagloedischia primitiva* in Gorokhov (2005a), Madygen Formation, Madygen/Dzhailoucho, south Fergana Valley, Kyrgyzstan.

F. Haglotettigoniidae Gorokhov, 1988a K1(Valanginian)

First and Last: *Haglotettigonia egregia* in Gorokhov (2005b), Zaza Formation, Baissa, Buryatia, Russian Federation.

F. Locustavidae T1(Induan)-T3(Carnian)

First: *Praelocustopsis mirabilis* in Gorokhov (2005b), Bugarikhta Formation, Nizhnyaya Tunguska river, Krasnoyarsk Krai, Siberian Federal District, Russian Federation.

Last: e.g. *Brevilocustavus microscopicus* Gorokhov, 2005b, Madygen Formation, Madygen/Dzhailoucho, south Fergana Valley, Kyrgyzstan.

F. Locustopseidae (Locustopsidae) T3(Carnian)-Eoc.(Priabonian)
Gorokhov (2005b) transferred the genera *Praelocustopsis* (Induan, Bugarikhta Formation, Siberia) and *Triassolocusta* (Carnian, Blackstone Formation, Australia) to the Locustavidae.

First: Mentioned in Martins-Neto (2003), Cow Branch Formation, Solite quarry, Virginia, United States.

Last: *Zeunerella*? *lewisi* Kevan and Wighton, 1981, Passamari Formation, Ruby River Basin, Montana, United States. (Although Gorokhov et al. 2006 state that the Locustopseidae "is known from the Early Triassic-Late Cretaceous" (p.657), this species has probably been missed because it was named in a footnote.)

F. Mesoedischiidae Gorokhov, 1987b T1(Induan)-T3(Carnian)

First: *Sonoedischia shmakovi* Gorokhov, 2005a, Babiy Kamen', Maltseva/Sosnovaya Fomation, Kuznetsk Basin, Siberian Federal District, Russian Federation.

Last: e.g. *Mesoedischia obliqua* in Gorokhov (2005a), Madygen Formation, Madygen/Dzhailoucho, south Fergana Valley, Kyrgyzstan.

F. Mesotitanidae (Clatrotitanidae, Gigatitanidae) P1(Kungurian)-T3(Carnian)

First: *Jubilaeus beybienkoi* in Béthoux and Nel (2002a), Koshelevka Formation, Tshekarda, Ural Mountains, Russian Federation. (Listed by Béthoux and Nel 2002a in Tcholmanvissiidae, Béthoux 2007a moves this genus to Mesotitanidae.)

Last: e.g. *Gigatitan vulgaris* in Gorokhov (2007), Madygen Formation, Madygen/Dzhailoucho, south Fergana Valley, Kyrgyzstan.

F. Mogoplistidae Mio.(Burdigalian)-Holocene

First: *Ornebius ambericus* in Heads (2009a), Dominican amber, Cordillera Septentrional, near Santiago, Dominican Republic.

F. Myrmecophilidae K1(Aptian)-Holocene

First: *Araripemyrmecophilops gracilis* in Martins-Neto (1995b), Crato Formation, Araripe Basin, Ceará, Brazil. (Heads and Martins-Neto 2007 did not mention this species as the section on it was omitted from the final print for unknown reasons [S. W. Heads pers. comm. 2011].)

F. Oedischiidae C2(Kasimovian)-P2(Wordian)

First: e.g. *Oedischia williamsoni* in Prokop et al. (2005), Upper Coal Measures, Commentry, Allier, France.

Last: e.g. *Iasvia secunda* Béthoux et al., 2002a, Salagou Formation (Mérifons Member), Lodève Basin, Hérault, France.

F. Paratitanidae T3(Carnian)

e.g. *Minititan zherichini* in Gorokhov (2007), Madygen Formation, Madygen/Dzhailoucho, south Fergana Valley, Kyrgyzstan.

F. Permelcanidae P1(Artinskian)-T3(Carnian)

First: *Promartynovia venicosta* in Beckemeyer (2000), Wellington Formation, Elmo site, Dickinson County, Kansas, United States.

Last: e.g. *Meselcana madygenica* in Gorokhov (2005a), Madygen Formation, Madygen/Dzhailoucho, south Fergana Valley, Kyrgyzstan.

F. Permoraphidiidae (Permoraphididae) P1(Artinskian)
Béthoux and Nel (2002b) described *Permoraphidia magnifica* from the Permian of Madagascar but as no further information on the origin or age is known, it has not been included in the range of this family here.

e.g. *Permoraphidia grandis* in Beckemeyer (2000), Wellington Formation, Elmo site, Dickinson County, Kansas, United States.

F. Phasmomimidae J3(Oxfordian)
Gorokhov (2000) restricts Phasmomimidae to the genera *Phasmomima* and *Jurophasmomima*.

e.g. *Phasmomima maculomarginata* in Gorokhov (2000), Karabastau Formation, Karatau Range, Tien Shan mountains, Kazakhstan.

F. Prezottophlebiidae Martins-Neto, 2007 K1(Aptian)

First and Last: *Prezotophlebia helbae* Martins-Neto, 2007, Crato Formation, Araripe Basin, Ceará, Brazil.

F. Promastacidae Eoc.(Ypresian)
Gorokhov (1988c) transferred the Palaeocene genus Promastacoides to the Phasmomimidae but later (Gorokhov, 2000) to Susumaniidae.

First and Last: *Promastax archaicus* in Kevan and Wighton (1981), Horsefly shales, Horsefly river, Cariboo, British Columbia, Canada.

F. Proparagryllacrididae T3(Carnian)

e.g. *Kashgarlimahmutia reducta* in Koçak and Kemal (2008), Madygen Formation, Madygen/Dzhailoucho, south Fergana Valley, Kyrgyzstan. (Both Koçak and Kemal 2008 and Özdikmen 2008a supplied replacement names for the junior homonym *Fergania* Sharov, however Koçak and Kemal 2008 has priority as it was published a month earlier.)

F. Prophalangopsidae (Prophalangopseidae) J1(Hettangian)-Holocene

First: *Aboilus tuzigouensis* Lin and Huang, 2006, Badaowan Formation, Kelamayi, Xinjiang Uyghur Autonomous Region, China.

F. Proscopiidae K1(Aptian)-Holocene

First: *Eoproscopia martilli* Heads, 2008a, Crato Formation, Araripe Basin, Ceará, Brazil.

F. Protogryllidae T3(Carnian)-J3(Oxfordian)
*Protogryllus minor* from the Berriasian Purbeck Beds (United Kingdom) is "Grylloidea incertae sedis" according to Gorokhov et al. (2006).

First: Mentioned in Gorokhov and Rasnitsyn (2002), Molteno Formation, KwaZulu-Natal, Karoo Basin, South Africa.

Last: e.g. *Karataogryllus gryllotalpiformis* in Perrichot et al. (2002), Karabastau Formation, Karatau Range, Tien Shan mountains, Kazakhstan.

F. Pruvostitidae (Kamiidae, Tettavidae) P1(Artinskian)-P2(Wordian)

First: *Paroedischia recta* in Béthoux and Nel (2002b), Wellington Formation, Elmo site, Dickinson County, Kansas, United States. (Family placement of this species is after Gorokhov 1995b and the Orthoptera Species File.)

Last: e.g. *Kargalaria maculata* in Gorokhov (1995b), Amanak Formation, Kargala, Belozersky District, Orenburg Region, Russian Federation.

F. Pseudelcanidae Gorokhov, 1987b P1(Kungurian)

e.g. *Pseudelcana permiana* Gorokhov, 1987b, Koshelevka Formation, Tshekarda, Ural Mountains, Russian Federation.

F. Pyrgomorphidae Mio.(Serravallian)-Holocene

First: *Miopyrgomorpha fischeri* in Zherikhin (2002c), Oeningen freshwater limestones, Schrotzburg, Baden-Württenburg, Germany.

F. Raphoglidae Béthoux et al., 2002b P2(Wordian)

First and Last: *Raphogla rubra* Béthoux et al., 2002b, Salagou Formation (Mérifons Member), Lodève Basin, Hérault, France.

F. Regiatidae Gorokhov, 1995a J1(Sinemurian)

e.g. *Regiata scutra* in Gorokhov (2005b), Black Ven Marls, Charmouth, Dorset, United Kingdom. (Originally described in the family Haglidae.)

F. Rhaphidophoridae (Raphidiophoridae, Raphidophoridae, Raphydophoridae) Eoc.(Priabonian)-Holocene

First: e.g. *Rhaphidophora antiqua* in Weitschat and Wichard (2002), Baltic amber.

F. Ripipterygidae (Rhipipterygidae) Mio.(Burdigalian)-Holocene

First: *Ripipteryx* sp. in Heads (2009b), Dominican amber, Cordillera Septentrional, near Santiago, Dominican Republic.

F. Tcholmanvissiidae P1(Kungurian)-P2(Roadian)

First: *Tcholmanvissia longipipes* in Béthoux and Nel (2002a), Koshelevka Formation, Tshekarda, Ural Mountains, Russian Federation.

Last: e.g. *Tcholmanvissia noinskii* in Béthoux and Nel (2002a), Iva-Gora limestones, Soyana River, Arkhangelsk Region, Ural Mountains, Russian Federation. (This species also occurs in the Baitugan Formation (Tikhie Gory); see Béthoux and Nel 2002a.)

F. Tetrigidae K1(Valanginian)-Holocene

First: e.g. *Prototetrix reductus* in Gorokhov and Rasnitsyn (2002), Zaza Formation, Baissa, Buryatia, Russian Federation. (Gorokhov and Rasnitsyn 2002 mistakenly figure this species under the name *P. reducta*.)

F. Tettigoniidae (Conocephalidae, Locustidae, Phaneropteridae, Tettigonidae) T2(Anisian)-Holocene

First: *Triassophyllum leopardii* Papier et al., 1997, Grès à Voltzia, Bas-Rhin/Moselle, Northern Vosges Mountains, France. (Gorokhov 2005b states that this species belongs in the homopteran family Ipsviciidae, however Gall and Grauvogel-Stamm 2005 maintain its position in Orthoptera and this is followed here.)

F. Tettoedischiidae P1(Kungurian)

e.g. *Tettoedischia minuta* in Béthoux (2007a), Koshelevka Formation, Tshekarda, Ural Mountains, Russian Federation.

F. Thueringoedischiidae Zessin, 1997 C2(Gzhelian)-P1(Asselian)

First: e.g.? *Hymenelcana initialis* Gorochov *in* Rasnitsyn et al., 2004a, Bursum Formation (Red Tanks Member), Carrizo Arroyo, New Mexico, United States.

Last: e.g. *Permoedischia moravica* in Zajíc and Štamberg (2004), Říčany Horizon, Padochov Formation, Moravia, Czech Republic.

F. Triassomanteidae (Triassomantidae) T3(Carnian)
*Triassomanteodes madygenicus* (Madygen Formation) is now considered to be in the Xenopteridae (Gorokhov, 2005a) and *Orichalcum ornatum* (Black Ven Marls) in Locustopseidae (Gorokhov et al., 2006).

First and Last: *Triassomantis pygmaeus* in Jell (2004), Blackstone Formation, Ipswich Basin, Queensland, Australia.

F. Tridactylidae K1(Berriasian)-Holocene
The exact position of Mongoloxyinae within Tridactyloidea is uncertain (Heads, 2009b) but is considered here to be in Tridactylidae until further study.

First: *Cretoxya rasnitsyni* Gorokhov et al., 2006, Lulworth Formation, Durlston Bay, Dorset, United Kingdom.

F. Tuphellidae Gorokhov, 1988b T2(Anisian)-J3(Tithonian)

First: *Triassoparacyrtophyllites bifurcatus* Marchal-Papier et al., 2000, Grès à Voltzia, Bas-Rhin/Moselle, Northern Vosges Mountains, France.

Last: *Paracyrtophyllites popovi* in Gorokhov (2005a), Shar-Teg Formation, Shar-Teg Ula, Gobi-Altai Aimag, Mongolia.

F. Vitimiidae (Vitimidae) K1(Valanginian)-K1(Barremian)

First: e.g. *Deinovitimia insolita* in Gorokhov et al. (2006), Zaza Formation, Baissa, Buryatia, Russian Federation.

Last: *Deinovitimia occidentalis* Gorokhov et al., 2006, Upper Weald Clay Formation, Capel, Surrey, United Kingdom.

F. Xenopteridae T3(Carnian)

e.g. *Axenopterum venosum* Gorokhov, 2005a, Madygen Formation, Madygen/Dzhailoucho, south Fergana Valley, Kyrgyzstan.

**O. Phasmatodea** Brunner von Wattenwyl, 1893 (Aeroplanoptera, Phasmatida, Phasmida, Timematodea) Permian(Capitanian)-Quaternary(Holocene)

The placement of pre-Cenozoic fossils assigned to Phasmatodea remains controversial (e.g. Tilgner, 2001) and are placed here as a matter of convenience.

F. Aerophasmatidae (Cretophasmatidae) J1(Sinemurian)-K2(Turonian)

First: *Durnovaria parallela* in Ansorge (1996b), Black Ven Marls, Charmouth, Dorset, United Kingdom.

Last: *Cretophasma raggei* in Heads and Martins-Neto (2007), Kzyl-Zhar, Karatau Range, Tien Shan mountains, Kazakhstan.

F. Aeroplanidae T3(Carnian)

e.g. *Aeroplana mirabilis* in Jell (2004), Blackstone Formation, Ipswich Basin, Queensland, Australia.

F. Agathemeridae Eoc.(Priabonian)-Holocene

First: *Agathemera reclusa* in Tilgner (2001), Florissant Formation, Florissant, Colorado, United States.

F. Archipseudophasmatidae Zompro, 2001 Eoc.(Priabonian)

e.g. *Dvergrphasma fafnir* Zompro, 2005, Baltic amber.

F. Diapheromeridae Mio.(Burdigalian)-Holocene

First: *Paraphanocles keratosqueleton* in Zompro (2001), Dominican amber, Cordillera Septentrional, near Santiago, Dominican Republic. (Fossil egg originally figured in Poinar and Poinar 1999.)

F. Necrophasmatidae J3(Oxfordian)

First and Last: *Necrophasma shabarovi* in Nel et al. (2004b), Karabastau Formation, Karatau Range, Tien Shan mountains, Kazakhstan.

F. Permophasmatidae P2(Capitanian)
Placement of this family in Phasmatodea *sensu lato* remains doubtful (Nel et al., 2004b).

First and Last: *Permophasma kovalevi* in Nel et al. (2004b), Tavan-Tolgoy, Bor-Tolgoy, Ömnögovi (South Gobi) Aimag, Mongolia.

F. Phasmatidae Mio.(Aquitanian)-Holocene

First: Mentioned in Solórzano Kraemer (2007), Mexican amber, Simojovel, Chiapas, Mexico.

F. Phylliidae (Phyllidae) Eoc.(Lutetian)-Holocene

First: *Eophyllium messelensis* Wedmann et al., 2007, Messel Formation, Grube Messel, Hesse, Germany.

F. Prochresmodidae T2(Anisian)-T3(Carnian)

First: *Palaeochresmoda grauvogeli* Nel et al., 2004b, Grès à Voltzia, Bas-Rhin/Moselle, Northern Vosges Mountains, France.

Last: e.g. *Triassophasma* sp. in Gorokhov and Rasnitsyn (2002), Madygen Formation, Madygen/Dzhailoucho, south Fergana Valley, Kyrgyzstan.

F. Pseudophasmatidae Eoc.(Lutetian)-Holocene

First: e.g. *Eophasmina manchesteri* in Tilgner (2001), Clarno Formation (Nut Beds), John Day Fossil Beds National Monument, Oregon, United States. (Tilgner 2001 expresses some doubt about the family placement of these fossil eggs as they resemble some Phasmatidae and the Pseudophasmatidae may not be monophyletic.)

F. Susumaniidae (Hagiphasmatidae) J3(Oxfordian)-Pal.(Thanetian)

First: e.g. *Phasmomimoides minutus* Gorokhov, 2000, Karabastau Formation, Karatau Range, Tien Shan mountains, Kazakhstan.

Last: e.g. *Promastacoides albertae* in Nel et al. (2004b), Paskapoo Formation, eastern foothills, Rocky Mountains, Alberta, Canada. (Originally placed in Phasmomimidae, Gorokhov 2000 moved this genus to Susumaniidae.)

F. Xiphopteridae T3(Carnian)

e.g. *Xiphopterum curvatum* in Gorokhov and Rasnitsyn (2002), Madygen Formation, Madygen/Dzhailoucho, south Fergana Valley, Kyrgyzstan.

**O. Plecoptera** Burmeister, 1839 (Perlaria, Perlida)
Permian(Kungurian)-Quaternary(Holocene)

F. Baleyopterygidae Sinitshenkova, 1987 J1(Pliensbachian)-K1(Valanginian)
Aristov and Rasnitsyn (2009) mistakenly state that *Plutopteryx beata* is of Middle Permian age, when in fact the Bayan-Teg locality is thought to be Middle Jurassic (Rasnitsyn and Zherikhin, 2002).

First: e.g. *Baleyopteryx orthoclada* in Sinitshenkova (2002b), Osinovskiy Formation, Chernyi Etap, Kemerovo Region, Russian Federation.

Last: e.g. *Baissoleuctra irinae* in Ansorge (1993), Zaza Formation, Baissa, Buryatia, Russian Federation.

F. Capniidae J1(Toarcian)-Holocene

First: *Dobbertiniopteryx capniomimus* in Liu et al. (2009), Upper Lias, Dobbertin, Mecklenburg-Vorpommern, Germany. (Liu et al. 2009 mistakenly state that this specimen is late Jurassic.)

F. Chloroperlidae J3(Tithonian)-Holocene

First: e.g. *Dipsoperla kunikanensis* Sinitshenkova, 1990, Glushkovo Formation, Unda, Transbaikalia, Russian Federation.

F. Eustheniidae P3(Changhsingian)-Holocene

First: e.g. *Stenoperlidium permianum* in Jell (2004), Belmont insect beds, Newcastle Coal Measures, Belmont/Warner's Bay, New South Wales, Australia.

F. Euxenoperlidae P2(Roadian)-T3(Carnian)

First: *Euxenoperla oliveri* in van Dijk and Geertsema (2004), Volksrust Formation, Ecca Group, KwaZulu-Natal, Karoo Basin, South Africa.

Last: e.g. *Gondwanoperlidium mendozensis* in Martins-Neto et al. (2007b), Potrerillos Formation, Cerro Bayo, Mendoza Province, Argentina.

F. Gripopterygidae J3(Tithonian)-Holocene

First: *Cardioperlisca tshitensis* Sinitshenkova, 1998, Doronino Formation, Chernovskie Kopi, Chita, Transbaikalia, Russian Federation.

F. Leuctridae (Leuctriidae) J3(Tithonian)-Holocene

First: *Lycoleuctra lupina* Sinitshenkova, 1987, Glushkovo Formation, Daya, Transbaikalia, Russian Federation.

F. Mesoleuctridae T3(Carnian)-K1(Aptian)
Mesoleuctridae do not occur in the Carnian Madygen Formation (Shcherbakov, 2008b).

First: *Capitiperla tonicopoda* Lin, 1992, Huangshanjie Formation, Kerjie, Toksun county, Xinjiang Uyghur Autonomous Region, China. (Originally described as Plecoptera *incertae familiae*, Liu and Ren 2006 list *Capitiperla* under Mesoleuctridae as does the Plecoptera Species File.)

Last: Mentioned in Liu et al. (2008b), Yixian Formation, Liaoning Province, China.

F. Nemouridae J2(Callovian)-Holocene

First: Mentioned in Liu et al. (2006), Jiulongshan Formation, near Daohugou, Ningcheng county, Inner Mongolia, China.

F. Palaeonemouridae Sinitshenkova, 1987 P1(Kungurian)-P3(Changhsingian)

First: e.g. *Uralonympha vorkutica* in Sinitshenkova (2004), Lek-Vorkuta Formation, Vorkuta Group, Pechora Cola Basin, Komi Republic, Russian Federation.

Last: e.g. *Palaeonemoura zwicki* in Sinitshenkova (2004), Maichat/Ak-Kolka Formation, Karaungir River, Saur Mountains, Vostochno-Kazakhstanskaya oblast, Kazakhstan.

F. Palaeoperlidae P2(Roadian)-P3(Changhsingian)

First: e.g. *Palaeoperla exacta* in Liu and Ren (2006), Kuznetsk Formation (Mitino Horizon), Kaltan, Kemerovo Region, Russian Federation.

Last: Mentioned in Sinitshenkova (2002b), Pelyatka Formation, Pelyatka River, Siberian Federal District, Russian Federation.

F. Perlariopseidae T3(Carnian)-K1(Barremian)

First: e.g. *Ramonemoura constricta* in Liu and Ren (2008), Madygen Formation, Madygen/Dzhailoucho, south Fergana Valley, Kyrgyzstan. (Liu and Ren 2008 call for the family placement of this species to be reassessed. Shcherbakov 2008b mentions there are five genera and thirteen species in this family from that deposit but does not name any of them.)

Last: e.g. *Accretonemoura radiata* Sinitshenkova, 1987, Khurilt Formation, Bon-Tsagaan Group, Bayankhongor Aimag, Mongolia.

F. Perlidae K1(Aptian)-Holocene

First: *Archaeoperla rarissimus* Liu, Ren & Sinitshenkova *in* Liu et al., 2008b, Jianshangou beds, Yixian Formation, Liaoning Province, China.

F. Perlodidae K1(Berriasian)-Holocene
The Mongolian locality of Khodont is considered here as lowermost Cretaceous, although those who consider it Upper Jurassic would therefore list *Derancheperla collaris* Sinitshenkova, 1990 as the oldest specimen in this family.

First: e.g. *Isoperlodes perstrictus* Sinitshenkova, 1992, Kempendyai locality, Suntar District, Sakha (Yakutia) Republic, Russian Federation.

F. Perlopseidae P1(Kungurian)

e.g. *Perlopsis filicornis* in Aristov and Rasnitsyn (2009), Koshelevka Formation, Tshekarda, Ural Mountains, Russian Federation.

F. Platyperlidae T3(Carnian)-K1(Aptian)

First: *Platyperla* sp. in Martins-Neto et al. (2008), Potrerillos Formation, Cerro Bayo, Mendoza Province, Argentina.

Last: Mentioned in Liu et al. (2007a), Yixian Formation, Liaoning Province, China.

F. Siberioperlidae T3(Carnian)-K1(Aptian)

First: *Siberioperla ovalis* in Shcherbakov (2008b), Madygen Formation, Madygen/Dzhailoucho, south Fergana Valley, Kyrgyzstan.

Last: *Sinosharaperla zhaoi* Liu et al., 2007a, Jianshangou beds, Yixian Formation, Liaoning Province, China.

F. Taeniopterygidae J2(Callovian)-Holocene

First: e.g. *Mengitaenioptera multiramis* Liu and Ren, 2008, Jiulongshan Formation, near Daohugou, Ningcheng county, Inner Mongolia, China.

F. Tshekardoperlidae Sinitshenkova, 1987(Tschekardoperlidae) P1(Kungurian)

e.g. *Sylvoperlodes zhiltzovae* in Sinitshenkova (2003), Koshelevka Formation, Tshekarda, Ural Mountains, Russian Federation.

**O. Protelytroptera** (Protelytrida) Permian(Sakmarian)-Permian(Changhsingian)

F. Archelytridae (Apachelytridae, Megelytridae) P1(Sakmarian)-P1(Artinskian)
Shcherbakov (2002) synonymised Apachelytridae and Megelytridae under this family without discussion.

First: e.g. *Ortelytron europeaum* in Zajíc and Štamberg (2004), Obora locality, Bačov Beds, Letovice Formation, Moravia, Czech Republic.

Last: e.g. *Archelytron superbum* in Beckemeyer (2000), Wellington Formation, Elmo site, Dickinson County, Kansas, United States.

F. Bardacoleidae P1(Kungurian)
This family was transfered to Protelytroptera and the type genus synonymised with *Uralelytron* by Shcherbakov (2002) without discussion.

e.g. *Uralelytron insignis* in Shcherbakov (2002), Koshelevka Formation, Tshekarda, Ural Mountains, Russian Federation.

F. Blattelytridae P1(Sakmarian)-P1(Artinskian)
Considered as a separate family by Shcherbakov (2002).

First: Mentioned in Shcherbakov (2002), Obora locality, Bačov Beds, Letovice Formation, Moravia, Czech Republic.

Last: e.g. *Parablattelytron latum* in Beckemeyer (2000), Wellington Formation, Elmo site, Dickinson County, Kansas, United States.

F. Dermelytridae P3(Changhsingian)

e.g. *Dermelytron conservativum* in Jell (2004), Belmont insect beds, Newcastle Coal Measures, Belmont/Warner's Bay, New South Wales, Australia.

F. Elytroneuridae P1(Sakmarian)-P1(Artinskian)

First: Mentioned in Shcherbakov (2002), Obora locality, Bačov Beds, Letovice Formation, Moravia, Czech Republic.

Last: *Elytroneura permiana* in Beckemeyer (2000), Wellington Formation, Elmo site, Dickinson County, Kansas, United States.

F. Labidelytridae (Stenelytridae) P3(Changhsingian)

e.g. *Labidelytron enervatum* in Jell (2004), Belmont insect beds, Newcastle Coal Measures, Belmont/Warner's Bay, New South Wales, Australia.

F. Permelytridae P1(Artinskian)
Beckemeyer (2000) lists *Blattelytron* and *Parablattelytron* under this family, that are considered to belong to the separate family Blattelytridae

First and Last: *Permelytron schucherti* in Beckemeyer (2000), Wellington Formation, Elmo site, Dickinson County, Kansas, United States.

F. Permofulgoridae P2(Roadian)-P3(Changhsingian)
Carpenter (1992b) does not mention this family nor the two genera assigned to it here. Shcherbakov (2002) places the families Labidelytridae, Permophilidae and Protocoleidae in Permofulgoridae without giving any argument. These families are kept separate here, following Jell (2004).

First: *Arctocoleus ivensis* in Shcherbakov (2002), Iva-Gora limestones, Soyana River, Arkhangelsk Region, Ural Mountains, Russian Federation.

Last: e.g. *Permofulgor belmontensis* in Jell (2004), Belmont insect beds, Newcastle Coal Measures, Belmont/Warner's Bay, New South Wales, Australia.

F. Permophilidae P3(Changhsingian)

e.g. *Permophilus pincombei* in Jell (2004), Belmont insect beds, Newcastle Coal Measures, Belmont/Warner's Bay, New South Wales, Australia.

F. Planelytridae P1(Sakmarian)

First and Last: *Planelytron planum* in Zajíc and Štamberg (2004), Obora locality, Bačov Beds, Letovice Formation, Moravia, Czech Republic.

F. Protelytridae P1(Sakmarian)-P1(Artinskian)

First: Mentioned in Shcherbakov (2002), Obora locality, Bačov Beds, Letovice Formation, Moravia, Czech Republic.

Last: e.g. *Protelytron permianum* in Beckemeyer (2000), Wellington Formation, Elmo site, Dickinson County, Kansas, United States.

F. Protocoleidae P3(Wuchiapingian)-P3(Changhsingian)

First: *Phyllelytron acuminatum* in van Dijk and Geertsema (1999), Normandien (Estcourt) Formation, Beaufort Group, KwaZulu-Natal, Karoo Basin, South Africa.

Last: e.g. *Austrelytron tillyardi* in Jell (2004), Belmont insect beds, Newcastle Coal Measures, Belmont/Warner's Bay, New South Wales, Australia.

**O. Protorthoptera** Handlirsch, 1906 (Blattinopseida, Eoblattida, Hypoperlida) Carboniferous(Moscovian)-Permian(Changhsingian)

F. Adeloneuridae C2(Moscovian)

First and Last: *Adeloneura thompsoni* in Carpenter (1992b), Carbondale Formation, Mazon Creek, Illinois, United States.

F. Anthracoptilidae (Permarrhaphidae) C2(Kasimovian)-P3(Changhsingian)

First: e.g. *Anthracoptilus* sp. in Rasnitsyn and Aristov (2004), Upper Coal Measures, Commentry, Allier, France.

Last: *Jarmilacladus variabilis* Rasnitsyn and Aristov, 2004, Belmont insect beds, Newcastle Coal Measures, Belmont/Warner's Bay, New South Wales, Australia.

F. Anthracothremmidae C2(Moscovian)

e.g. *Melinophlebia analis* in Brauckmann and Herd (2006), Carbondale Formation, Mazon Creek, Illinois, United States.

F. Apithanidae C2(Moscovian)

First and Last: *Apithanus jocularis* in Rasnitsyn (2002k), Carbondale Formation, Mazon Creek, Illinois, United States.

F. Asiopompidae C2(Kasimovian)

First and Last: *Asiopompus tomicus* in Rohdendorf (1991), Alykaeva Formation, Kuznetsk Basin, Siberian Federal District, Russian Federation.

F. Asiuropidae Novokshonov, 1997a P1(Kungurian)

First and Last: *Asiuropa uralensis* Novokshonov, 1997a, Koshelevka Formation, Tshekarda, Ural Mountains, Russian Federation.

F. Asyncritidae C2(Moscovian)

First and Last: *Asyncritus reticulatus* Handlirsch, 1911, Carbondale Formation, Mazon Creek, Illinois, United States.

F. Boltonocostidae Ross et al., 2013('Orthocostidae') C2(Moscovian)
The original family name, 'Orthocostidae' Bolton, 1912 is not valid as the type genus was renamed, due to homonomy, by Carpenter (1986). Labandeira (1994) lists this family in Palaeodictyoptera but Rasnitsyn (2002e) placed *Boltonocosta* in Hypoperlida.

First and Last: *Boltonocosta splendens* in Carpenter (1992b), below the Top Hard Coal, Middle Coal Measures, Shipley Manor Claypit, Ilkeston, Derbyshire, United Kingdom.

F. Cymbopsidae P1(Sakmarian)
Rasnitsyn (2002c) thinks that this monotypic family could be an abberant member of Blattinopsidae.

First and Last: *Cymbopsis excelsa* in Zajíc and Štamberg (2004), Obora locality, Bačov Beds, Letovice Formation, Moravia, Czech Republic.

F. Eucaenidae (Teneopteridae) C2(Moscovian)

e.g. *Eucaenus ovalis* in Labandeira (2001), Carbondale Formation, Mazon Creek, Illinois, United States.

F. Evenkidae C2(Gzhelian)
Not to be confused with Actinopterygii: Evenkiidae.

First and Last: *Evenka archaica* in Rasnitsyn (2002a), Kata Formation, Chunya, Siberian Federal District, Russian Federation.

F. Gerapompidae (Cheliphlebidae, Cheliphlebiidae) C2(Moscovian)
Rasnitsyn (2002k) tentatively included *Aenigmatella* in this family but Brauckmann and Herd (2006) consider it unplaced. Rasnitsyn (2002k) also includes *Cheliphblebia* in this family.

e.g. *Palaeocarria ornata* in Rasnitsyn (2002k), Carbondale Formation, Mazon Creek, Illinois, United States.

F. Herdinidae C2(Moscovian)

e.g. *Herdina mirificus* in Béthoux and Nel (2002b), Carbondale Formation, Mazon Creek, Illinois, United States.

F. Heteroptilidae P1(Artinskian)
Rasnitsyn (2002e) synonymized Heteroptilidae under Tococladidae without argument, which was rejected by Béthoux et al. (2003a).

First and Last: *Heteroptilon costale* in Rasnitsyn (2002e), Wellington Formation, Elmo site, Dickinson County, Kansas, United States.

F. Homalophlebiidae C2(Kasimovian)

e.g. *Parahomalophlebia courtini* in Rasnitsyn (2002k), Upper Coal Measures, Commentry, Allier, France.

F. Hypermegethidae C2(Moscovian)-C2(Gzhelian)
Previously placed in the Palaeodictyoptera, Sinitshenkova (2002a) places this family in the Hypoperlida.

First: *Hypermegethes schucherti* in Carpenter (1992a), Carbondale Formation, Mazon Creek, Illinois, United States.

Last: *Hypermegethes pilchi* Carpenter, 1992a, Lawrence Formation, Douglas County, Kansas, United States.

F. Hypoperlidae (Martynopsocidae) P1(Kungurian)-P2(Roadian)

First: e.g. *Idelopsocus incommendatus* Novokshonov et al., 2002, Solikamsk Formation, Vishera River, Mogil'nikovo, Ural Mountains, Russian Federation.

Last: e.g. *Hypoperla elegans* in Novokshonov (2001), Iva-Gora limestones, Soyana River, Arkhangelsk Region, Ural Mountains, Russian Federation.

F. Kliveriidae (Kliveridae) C2(Moscovian)

First and Last: *Kliveria incerta* in Brauckmann and Herd (2006), Richard shaft, Dudweiler mine, Saarbrücken, Saarland, Germany.

F. Nugonioneuridae (Nungonioneuridae) P1(Artinskian)
Rasnitsyn (2002e) synonymized Nugonioneuridae under Tococladidae without argument, which was rejected by Béthoux et al. (2003a).

First and Last: *Nugonioneura problematica* in Rasnitsyn (2002e), Wellington Formation, Elmo site, Dickinson County, Kansas, United States.

F. Perielytridae P1(Kungurian)

First and Last: *Perielytron mirabile* in Rasnitsyn (2002e), Koshelevka Formation, Tshekarda, Ural Mountains, Russian Federation.

F. Prototettigidae (Protettigae, Prototettigae) C2(Moscovian)
Rasnitsyn (2002k) places this family in 'Eoblattida'.

First and Last: *Prototettix lithanthraca* in Handlirsch (1908), Frankenholz Mine, Neunkirchen, Saarland, Germany.

F. Psoropteridae P1(Artinskian)

First and Last: *Psoroptera cubitalia* in Beckemeyer (2000), Wellington Formation, Elmo site, Dickinson County, Kansas, United States.

F. Rigattopteridae Pinto, 1996 P1(Asselian)
Béthoux and Nel (2002b) retain this family in the Protorthoptera.

First and Last: *Rigattoptera ornellasae* Pinto, 1996, Bajo de Véliz Formation (Pallero Member), Paganzo Basin, Sierra Grande de San Luis, San Luis Province, Argentina.

F. Sojanoperidae Novokshonov, 2002b P2(Roadian)

First and Last: *Sojanopus festivum* Novokshonov, 2002b, Iva-Gora limestones, Soyana River, Arkhangelsk Region, Ural Mountains, Russian Federation.

F. Stenoneuridae C2(Kasimovian)-C2(Gzhelian)

First: e.g. *Stenoneura fayoli* in Rasnitsyn et al. (2004a), Upper Coal Measures, Commentry, Allier, France.

Last: Mentioned in Rasnitsyn et al. (2004a), Bursum Formation (Red Tanks Member), Carrizo Arroyo, New Mexico, United States.

F. Synomaloptilidae P1(Kungurian)
Béthoux et al. (2004c) concurred with Rasnitsyn (2002e) in excluding this monobasic family from the Caloneurodea.

First and Last: *Synomaloptila longipes* in Rasnitsyn (2002e), Koshelevka Formation, Tshekarda, Ural Mountains, Russian Federation.

F. Thoronysididae (Thoronysidae) C2(Moscovian)

First and Last: *Thoronysis ingbertensis* in Rasnitsyn (2002k), St. Ingbert Formation, Saarbrücken, Saarland, Germany.

**O. Zoraptera** Silvestri, 1913 Cretaceous(Barremian)-Quaternary(Holocene)

F. Zorotypidae K1(Barremian)-Holocene

First: *Zorotypus* (*Octozoros*) *hudae* in Engel (2008a), Jordanian amber, Kurnub Sandstone Formation, Zarqua River, Jordan.

**Archaeorthoptera** *incertae sedis*

F. Ampelipteridae (Fatjanopteridae, Protoprosbolidae) C1(Serpukhovian)-P2(Roadian)
Supraordinal placement after Béthoux and Nel (2002b).

First: *Ampeliptera limburgica* in Prokop et al. (2005), Gulpen, Gulpen, Limbourg, Netherlands.

Last: e.g. *Tshekardobia magnifica* Novokshonov *in* Novokshonov and Aristov, 2004, Iva-Gora limestones, Soyana River, Arkhangelsk Region, Ural Mountains, Russian Federation.

F. Cacurgidae C2(Bashkirian)-C2(Moscovian)
Considered here to include those taxa assigned in Carpenter (1992b) until further revision is performed.

First: e.g. *Heterologopsis ruhrensis* in Brauckmann (2005), Vorhalle Beds, Hagen-Vorhalle, Schmiedestraße, Wuppertal, North Rhine-Westphalia, Germany.

Last: e.g. *Cacurgus spilopterus* in Béthoux (2006), Carbondale Formation, Mazon Creek, Illinois, United States.

F. Carpenteropteridae Pinto and Pinto de Ornellas, 1991(Cacurgonarkemidae) C2(Kasimovian)
The species comprising this family were assigned by Béthoux (2007a) as unplaced within Archaeorthoptera. *Carpenteroptera rochacamposi* (previously in *Narkemina*) is added to this family in Martins-Neto et al. (2007a).

e.g. *Carpenteroptera onzii* in Martins-Neto (2005), Anitápolis Formation, Itararé Subgroup, Parana Basin, Fazenda do Juca, Santa Catarina, Brazil.

F. Chresmodidae (Sternarthronidae) J2(Callovian)-K2(Cenomanian)

First: e.g. *Jurachresmoda sanyica* Zhang, Ren & Pang *in* Zhang et al., 2009b, Jiulongshan Formation, near Daohugou, Ningcheng county, Inner Mongolia, China.

Last: *Chresmoda libanica* in Delclòs et al. (2008), Nammoura "fish beds", El Ghabour valley, Caza Kesrouâne, Mouhafazet Jabal Loubnan, Lebanon.

F. Eoblattidae C2(Kasimovian)

e.g. *Eoblatta robusta* in Béthoux and Nel (2005), Upper Coal Measures, Commentry, Allier, France. (Béthoux and Nel 2005 remove this genus from the Stenoneuridae.)

F. Geraridae C2(Moscovian)-C2(Gzhelian)

First: e.g. *Gerarus vetus* in Béthoux and Briggs (2008), Carbondale Formation, Mazon Creek, Illinois, United States.

Last: *Ploetzgerarus krempieni* Zessin, 2009, Plötz coal seams, near Halle, Saxony-Anhalt, Germany.

F. 'Omaliidae' (Coseliidae) C2(Bashkirian)-C2(Kasimovian)
This family name is a junior homonym of the extant Coleoptera subfamily Omaliinae MacLeay, 1825. A replacement name has been submitted to the ICZN Commission, case no. 3634. Family status and position after Béthoux and Nel (2002b).

First: e.g. *Omalia macroptera* in Béthoux and Nel (2005), Sars-Lonchamps, Mons Basin, La Louvière, Wallonia, Hainaut Province, Belgium.

Last: *Omalia anae* Brauckmann et al., 2001, Magdalena shales, La Magdalena, León Province, Spain. (Béthoux and Nel 2005 dispute whether this species belongs in *Omalia*.)

F. Pachytylopsidae C2(Bashkirian)
Béthoux and Nel (2002b) remove all but the type genus from this family and assign it to the Archaeorthoptera *nec* Panorthoptera. However, Brauckmann and Herd (2006) appear to retain *Protopachytylopsis* in Pachytylopsidae.

e.g. *Protopachytylopsis leckwycki* in Brauckmann and Herd (2006), Tergnee colliery, Wallonia, Hainaut Province, Belgium.

F. Protophasmatidae C2(Moscovian)-C2(Kasimovian)

First: e.g. *Protophasma galtieri* Béthoux and Schneider, 2009, Carbondale Formation, Mazon Creek, Illinois, United States.

Last: *Protophasma dumasii* in Béthoux (2003), Upper Coal Measures, Commentry, Allier, France.

**Polyneoptera** *incertae sedis*

F. Brachyphyllophagidae Rasnitsyn *in* Rasnitsyn and Krassilov, 2000 J3(Oxfordian)

e.g. *Brachyphyllophagus phasma* Rasnitsyn *in* Rasnitsyn and Krassilov, 2000, Karabastau Formation, Karatau Range, Tien Shan mountains, Kazakhstan.

F. Gelasopteridae P1(Artinskian)

First and Last: *Gelasopteron gracile* in Béthoux et al. (2004c), Wellington Formation, Elmo site, Dickinson County, Kansas, United States.

F. Gryllomantidae Gorokhov, 2006 K1(Barremian)-K1(Albian)
Gorokhov (2006) notes that this family may include an undescribed nymph in Dominican amber.

First: e.g. *Gryllomantis lebanensis* in Gorokhov (2006), Bcharreh amber, Caza Bcharreh, Mouhafazet Loubnan Eshemali, Lebanon.

Last: e.g. *Burmantis burmitica* in Gorokhov (2006), Burmese amber (Burmite), Hukawng Valley, Kachin State, Myanmar.

F. Lemmatophoridae (Germanopriscidae) P1(Asselian)-P2(Wordian)
Beckemeyer (2009a) follows Grimaldi and Engel (2005) and Arillo and Engel (2006) in regarding this family as Polyneoptera *incertae sedis* while Aristov (2009c) retains it in Grylloblattodea. *Karaungirella minuta*, listed as last in Ross and Jarzembowski (1993) belongs in the miomopteran family Permosialidae (Aristov, 2004a)

First: e.g. *Artinska* sp. in Hörnschemeyer (1999), Jeckenbach layers, Niedermoschel, Donnersbergkreis district, Rhineland-Palatinate, Germany.

Last: *Kostovatoprisca acuminata* Aristov, 2008a, Galevo (Kostovaty) locality, Kama river, Udmurt Republic, Russian Federation.

F. Mantoblattidae Gorokhov, 2006 K1(Albian)

First and Last: *Mantoblatta mira* Gorokhov, 2006, Burmese amber (Burmite), Hukawng Valley, Kachin State, Myanmar.

F. Tshekarcephalidae Novokshonov and Rasnitsyn, 2000 P1(Kungurian)-P2(Roadian)

First: *Tshekarcephalus bigladipotens* Novokshonov and Rasnitsyn, 2000, Koshelevka Formation, Tshekarda, Ural Mountains, Russian Federation.

Last: *Tshekarcephalus sojanensis* in Aristov and Rasnitsyn (2008), Iva-Gora limestones, Soyana River, Arkhangelsk Region, Ural Mountains, Russian Federation.

## Eumetabola

**O. Glosselytrodea** Martynov, 1938 (Jurinida) Permian(Artinskian)-Jurassic(Callovian)

F. Archoglossopteridae P2(Roadian)

First and Last: *Archoglossopterum shoricum* in Béthoux et al. (2001), Kuznetsk Formation (Mitino Horizon), Kaltan, Kemerovo Region, Russian Federation.

F. Glosselytridae P2(Roadian)-P2(Capitanian)

First: *Glosselytron multivenosum* in Béthoux et al. (2001), Iva-Gora limestones, Soyana River, Arkhangelsk Region, Ural Mountains, Russian Federation.

Last: e.g. *Glosselytron linguale* Ponomarenko, 2000a, Tsankhi (Tsankhin) Formation, Bor-Tolgoy, Ömnögovi (South Gobi) Aimag, Mongolia.

F. Glossopteridae P1(Kungurian)

e.g. *Glossopterum sharovi* in Béthoux et al. (2001), Koshelevka Formation, Tshekarda, Ural Mountains, Russian Federation.

F. Jurinidae P2(Roadian)-P3(Changhsingian)
Rasnitsyn (2002h) proposed to synonymise Archoglossopteridae, Glosselytridae, Glossopteridae and Uskatelytridae under this family, however Grimaldi and Engel (2005), Hong (2007a) and Huang et al. (2007a) discuss them separately.

First: e.g. *Eoglosselytrum kaltanicum* in Béthoux et al. (2007a), Kuznetsk Formation (Mitino Horizon), Kaltan, Kemerovo Region, Russian Federation.

Last: e.g. *Eoglosselytrum perplexa* in Béthoux et al. (2007a), Belmont insect beds, Newcastle Coal Measures, Belmont/Warner's Bay, New South Wales, Australia.

F. Permoberothidae P1(Artinskian)
According to Béthoux et al. (2007a), Permoberothidae does belong to Glosselytrodea, *contra* Béthoux et al. (2001) and Grimaldi and Engel (2005).

e.g. *Permoberotha villosa* in Beckemeyer and Hall (2007), Wellington Formation, Elmo site, Dickinson County, Kansas, United States.

F. Polycytellidae P3(Changhsingian)-J2(Callovian)

First: *Karajurina unica* in Béthoux et al. (2001), Maichat/Ak-Kolka Formation, Karaungir River, Saur Mountains, Vostochno-Kazakhstanskaya oblast, Kazakhstan.

Last: *Mongolojurina altaica* in Béthoux et al. (2001), Togo-Khuduk Member, Bakhar Series, Bayankhongor Aimag, Mongolia.

F. Uskatelytridae P3(Wuchiapingian)-J1(Sinemurian)

First: *Uskatelytrum sibiricum* in Béthoux et al. (2001), Erunakovo Formation, Kuznetsk Basin, Siberian Federal District, Russian Federation.

Last: *Mesojurina sogjutensis* in Béthoux et al. (2001), Dzhil Formation, Sogyuty, Issyk-Kul, Kyrgyzstan.

**O. Miomoptera** Martynov, 1927 (Palaeomanteida)
Carboniferous(Bashkirian)-Jurassic(Toarcian)

F. Archaemiopteridae (Archaemionopteridae) C2(Bashkirian)-T2(Ladinian)

First: *Eodelopterum priscum* in Grimaldi and Engel (2005), Essen Formation, Ruhr, North Rhine-Westphalia, Germany.

Last: *Triasomiomopteris oblongata* Hong, 2009a, Tongchuan Formation, Hejiafang, Tongchuan District, Shaanxi Province, China.

F. Palaeomanteidae (Delopteridae, Epimastacidae, Palaeomantidae) C2(Moscovian)-P3(Wuchiapingian)

First: Mentioned in Novokshonov and Zhuzhgova (2004), Carbondale Formation, Mazon Creek, Illinois, United States.

Last: *Palaeomantis* sp. in van Dijk and Geertsema (1999), Normandien (Estcourt) Formation, Beaufort Group, KwaZulu-Natal, Karoo Basin, South Africa.

F. Palaeomantiscidae P1(Kungurian)

e.g. *Sellardsiopsis conspicua* in Novokshonov and Zhuzhgova (2004), Koshelevka Formation, Tshekarda, Ural Mountains, Russian Federation.

F. Permembiidae (Letopalopteridae, Sheimiidae, Visheriferidae) P1(Artinskian)-P2(Roadian)

First: *Permembia delicatula* in Aristov and Rasnitsyn (2008), Wellington Formation, Elmo site, Dickinson County, Kansas, United States.

Last: e.g. *Soyanembia sharovi* Aristov and Rasnitsyn, 2008, Iva-Gora limestones, Soyana River, Arkhangelsk Region, Ural Mountains, Russian Federation.

F. Permosialidae (Perloblattidae, Permonkidae, Permosialididae, Tologopteridae) P1(Kungurian)-J1(Toarcian)

First: *Permosialis punctimaculosa* in Novokshonov and Zhuzhgova (2004), Koshelevka Formation, Tshekarda, Ural Mountains, Russian Federation.

Last: *Permonka jurassica* in Novokshonov and Zhuzhgova (2004), Sagul Formation, Sai-Sagul, Batkenskii District, Kyrgyzstan.

## Paraneoptera

**O. Hemiptera** Linnaeus, 1758 (Cimicida, Hemipsocoptera, Palaeohemiptera)
Carboniferous(Gzhelian)-Quaternary(Holocene)

Andersen (1998) described *Daniavelia morsensis* from the Fur Formation in Macroveliidae but Andersen and Grimaldi (2001) and Damgaard (2008a) reject this placement, leaving Macroveliidae without a fossil record. Despite being listed in Ross and Jarzembowski (1993) and Labandeira (1994), Shcherbakov (2006) notes that Tettigometridae does not have a fossil record.

F. Acanthosomatidae Eoc.(Lutetian)-Holocene

First: Figured in Wappler (2003), Eckfeld maar, Manderscheid, Rhineland-Palatinate, Germany.

F. Achilidae K1(Barremian)-Holocene
The Jordanian amber record figured in Kaddumi (2005) is doubtful.

First: e.g. Mentioned in Szwedo (2008a), Bon-Tsagaan Nuur, Bon-Tsagaan Group, Bayankhongor Aimag, Mongolia.

F. Adelgidae K1(Albian)-Holocene

First: Mentioned in Koteja and Poinar (2001), Alaskan amber, Kuk deposits, Brooks Range, Alaska, United States.

F. Aetalionidae (Biturritidae, Biturritiidae) J1(Sinemurian)-Holocene

First: e.g. *Absoluta distincta* in Carpenter (1992b), Dzhil Formation, Sogyuty, Issyk-Kul, Kyrgyzstan.

F. Albicoccidae Koteja, 2004 K1(Albian)

First and Last: *Albicoccus dimai* Koteja, 2004, Burmese amber (Burmite), Hukawng Valley, Kachin State, Myanmar.

F. Aleyrodidae (Aleurodicidae, Bernaeidae) J3(Oxfordian)-Holocene

First: *Juleyrodes visnyai* Shcherbakov, 2000a, Karabastau Formation, Karatau Range, Tien Shan mountains, Kazakhstan.

F. Alydidae J3(Oxfordian)-Holocene

First: *Monstrocoreus quadrimaculatus* in Yao et al. (2008), Karabastau Formation, Karatau Range, Tien Shan mountains, Kazakhstan.

F. Anthocoridae K1(Hauterivian)-Holocene

First: e.g. *Eoanthocoris cretaceus* in Shcherbakov and Popov (2002), Turga Formation, Turga River, near Borzai, Transbaikalia, Russian Federation.

F. Aphalaridae (Paleoaphalaridae, Paleoaphalaridae) Eoc.(Priabonian)-Holocene

First: e.g. *Eogyropsylla magna* Klimaszewski, 1997, Baltic amber.

F. Aphelocheiridae (Atopositidae) Plio.(Piacenzian)-Holocene

First: *Aphelocheirus affinis* in Popov (2007), Willershausen, Harz mountains, Lower Saxony, Germany.

F. Aphididae (Anoeciidae, Aphidae, Callaphididae, Drepanosiphidae, Eriosomatidae, Greenideidae, Hormaphididae, Mindaridae, Pemphigidae, Phloemyzidae, Phloeomyzidae, Sinaphididae) K1(Barremian)-Holocene
*Jurocallis longipes* from the Upper Jurassic Karabastau Formation is considered Aphidoidea *incertae sedis* by Carpenter (1992b) and the Aphid Species File (Version 1.0/4.0).

First: e.g. *Sunaphis laiyanensis* in Wang et al. (2006b), Laiyang Formation, Laiyang County, Shandong Province, China.

F. Aphrophoridae K1(Albian)-Holocene

First: Mentioned in Rasnitsyn and Ross (2000), Burmese amber (Burmite), Hukawng Valley, Kachin State, Myanmar.

F. Aradidae J3(Oxfordian)-Holocene

First: e.g.? *Aradus* sp(p). in Popov and Bechly (2007), Karabastau Formation, Karatau Range, Tien Shan mountains, Kazakhstan.

F. Archegocimicidae (Archaegocimicidae, Diatillidae, Eonabidae) J1(Sinemurian)-K1(Aptian)

First: e.g. *Britannicola senilis* Popov et al., 1994, Apperley locality, Apperley, Gloucestershire, United Kingdom.

Last: Mentioned in Popov and Bechly (2007), Crato Formation, Araripe Basin, Ceará, Brazil.

F. Archescytinidae (Lithoscytinidae, Permothripidae) C2(Gzhelian)-T1(Induan)

First: *Arroyoscyta novaemexicana* Rasnitsyn *in* Rasnitsyn et al., 2004a, Bursum Formation (Red Tanks Member), Carrizo Arroyo, New Mexico, United States. (Specimen only tentatively assigned to Archescytinidae and to Hemiptera in general; see Rasnitsyn et al. 2004a.)

Last: Mentioned in Shcherbakov (2008a), Bugarikhta Formation, Nizhnyaya Tunguska river, Krasnoyarsk Krai, Siberian Federal District, Russian Federation.

F. Archiconiopterygidae Ansorge, 1996a J1(Toarcian)

First and Last: *Archiconiopteryx liasina* in Engel (2004c), Upper Lias, Grimmen, Mecklenburg-Vorpommern, Germany.

F. Archijassidae J1(Toarcian)-K1(Barremian)

First: e.g. *Ardela grimmenensis* in Ansorge (2003a), Upper Lias, Grimmen, Mecklenburg-Vorpommern, Germany.

Last: *Archijassus plurinervis* in Wang et al. (2006b), Laiyang Formation, Laiyang County, Shandong Province, China.

F. Arnoldidae Eoc.(Priabonian)

e.g. *Arnoldus capitatus* Koteja, 2008, Baltic amber.

F. Belostomatidae (Paranoikidae) T3(Carnian)-Holocene

First: Figured in Grimaldi and Engel (2005), Cow Branch Formation, Solite quarry, Virginia, United States.

F. Berytidae (Berythidae) Eoc.(Priabonian)-Holocene

First: Mentioned in Shcherbakov and Popov (2002), Baltic amber.

F. Boreoscytidae P1(Kungurian)-P2(Roadian)
The genus *Megaleurodes* (Aptian, Crato Formation) does not belong to this family (Szwedo, 2007a).

First: *Dinoscyta microcephala* Shcherbakov, 2007a, Koshelevka Formation, Tshekarda, Ural Mountains, Russian Federation.

Last: e.g. *Boreoscyta nefasta* in Shcherbakov (2007a), Iva-Gora limestones, Soyana River, Arkhangelsk Region, Ural Mountains, Russian Federation.

F. Burmacoccidae Koteja, 2004 K1(Albian)

First and Last: *Burmacoccus danyi* Koteja, 2004, Burmese amber (Burmite), Hukawng Valley, Kachin State, Myanmar.

F. Burmitaphidae Poinar and Brown, 2005 K1(Albian)

e.g. *Burmitaphis prolatum* Poinar and Brown, 2005, Burmese amber (Burmite), Hukawng Valley, Kachin State, Myanmar.

F. Caliscelidae K2(Campanian)-Holocene

First: Mentioned in McKellar et al. (2008), Canadian amber, Grassy Lake, Alberta, Canada.

F. Canadaphididae (Canadaphidae) K1(Barremian)-K2(Campanian)

First: *Nuuraphis gemma* Wegierek, 1991, Bon-Tsagaan Nuur, Bon-Tsagaan Group, Bayankhongor Aimag, Mongolia.

Last: e.g. *Alloambria infelicis* in McKellar et al. (2008), Canadian amber, Cedar Lake, Manitoba, Canada.

F. Carsidaridae Eoc.(Priabonian)-Holocene

First: e.g. *Carsidarina hooleyi* in Ross and Jarzembowski (1993), Bembridge Marls Insect Limestone, Gurnard/Thorness Bay, Isle of Wight, United Kingdom.

F. Ceratocombidae Eoc.(Priabonian)-Holocene

First: Mentioned in Weitschat and Wichard (2002), Baltic amber.

F. Cercopidae P3(Changhsingian)-Holocene

First: *Tychticoloides belmontensis* in Jell (2004), Belmont insect beds, Newcastle Coal Measures, Belmont/Warner's Bay, New South Wales, Australia.

F. Cercopionidae Hamilton, 1990 K1(Aptian)

First and Last: *Cercopion reticulata* in Menon et al. (2007), Crato Formation, Araripe Basin, Ceará, Brazil.

F. Ceresopseidae J1(Sinemurian)

e.g. *Ceresopsis costalis* in Shcherbakov (2008c), Dzhil Formation, Sogyuty, Issyk-Kul, Kyrgyzstan.

F. Chiliocyclidae T3(Carnian)

e.g. *Chiliocycla scolopoides* in Jell (2004), Blackstone Formation, Ipswich Basin, Queensland, Australia.

F. Cicadellidae (Aphrodidae, Ceolidiidae, Eurymelidae, Euscelidae, Iassidae, Jascopidae, Jassidae, Macropsidae, Spinidae, Tettigellidae) T3(Carnian)-Holocene
Shcherbakov and Popov (2002) consider this family to have first appeared near the Jurassic/Cretaceous boundary.

First: e.g. *Eurymelidium australe* in Jell (2004), Blackstone Formation, Ipswich Basin, Queensland, Australia.

F. Cicadidae (Tibicinidae) Pal.(Thanetian)-Holocene

First: *Davispia bearcreekensis* in Carpenter (1992b), shales near Eagle coal mine, Foster Gulch, Fort Union Group, Montana, United States. (Shcherbakov 2009 confirms this record as the oldest currently known Cicadidae.)

F. Cimicidae K1(Albian)-Holocene

First: *Quasicimex eilapinastes* Engel, 2008b, Burmese amber (Burmite), Hukawng Valley, Kachin State, Myanmar.

F. Ciriacremidae Mio.(Burdigalian)-Holocene

First: *Sulciana macroconi* in Pérez-Gelabert (2008), Dominican amber, Cordillera Septentrional, near Santiago, Dominican Republic.

F. Cixiidae (Cicixiidae) K1(Valanginian)-Holocene
Jell (2004) lists the Triassic genera *Mesocixiodes*, *Mesocixius* and *Triassocixius* in this family but these genera are placed as Fulgoromorpha *incertae sedis* by Szwedo et al. (2004).

First: Figured in Shcherbakov and Popov (2002), Zaza Formation, Baissa, Buryatia, Russian Federation.

F. Clastopteridae Eoc.(Priabonian)-Holocene

First: *Clastoptera comstocki* in Carpenter (1992b), Florissant Formation, Florissant, Colorado, United States.

F. Coccidae Eoc.(Priabonian)-Holocene

First: Mentioned in Koteja (2000a), Baltic amber.

F. Coleoscytidae P2(Roadian)

e.g. *Coleoscyta rotundata* in Szwedo et al. (2004), Iva-Gora limestones, Soyana River, Arkhangelsk Region, Ural Mountains, Russian Federation.

F. Coreidae (Corizidae) T3(Carnian)-Holocene

First: *Kerjiecoris oopsis* in Yao et al. (2008), Huangshanjie Formation, Kerjie, Toksun county, Xinjiang Uyghur Autonomous Region, China.

F. Corixidae T3(Carnian)-Holocene

First: e.g. *Crypsacorixa tachis* Lin, 1992, Huangshanjie Formation, Kerjie, Toksun county, Xinjiang Uyghur Autonomous Region, China.

F. Creaphididae Shcherbakov and Wegierek, 1991(Creaphidae) T3(Carnian)

First and Last: *Creaphis theodora* in Hong et al. (2009), Madygen Formation, Madygen/Dzhailoucho, south Fergana Valley, Kyrgyzstan.

F. Cretamyzidae Heie *in* Heie and Pike, 1992 K2(Campanian)

First and Last: *Cretamyzus pikei* in McKellar et al. (2008), Canadian amber, Grassy Lake, Alberta, Canada.

F. Cuneocoridae J1(Toarcian)

First and Last: *Cuneocoris geinitzi* in Carvalho (1985), Upper Lias, Dobbertin, Mecklenburg-Vorpommern, Germany.

F. Curvicubitidae Hong, 1984(Curvicicubitidae) T2(Anisian)-T3(Carnian)

First: e.g. *Beaconiella fennahi* in Jell (2004), Hawkesbury Sandstone, Brookvale Quarry, Beacon Hill, New South Wales, Australia. (Jell 2004 lists the two species of *Beaconiella* in the family Fulgoridae, however this genus is included in the family Curvicubitidae by Szwedo et al. 2004 following the work of Shcherbakov. Shcherbakov 2008a mentions this family as occurring in the Anisian of Australia, but does not mention the taxa.)

Last: Mentioned in Shcherbakov (2008b), Madygen Formation, Madygen/Dzhailoucho, south Fergana Valley, Kyrgyzstan.

F. Cydnidae (Latiscutellidae, Pricecoridae) J1(Toarcian)-Holocene

First: Mentioned in Grimaldi and Engel (2005), Upper Lias, Dobbertin, Mecklenburg-Vorpommern, Germany.

F. Dactylopiidae Mio.(Aquitanian)-Holocene

First: Mentioned in Engel (2004a), Mexican amber, Simojovel, Chiapas, Mexico.

F. Delphacidae (Araeopidae) Eoc.(Ypresian)-Holocene

First: *Delphax senilis* in Szwedo et al. (2004), Green River Formation, Unitas area, Colorado, United States.

F. Derbidae Eoc.(Priabonian)-Holocene

First: e.g. *Emeljanovedusa gentarna* Szwedo, 2006, Baltic amber. (Specimen from Poland.)

F. Diaspididae T3(Carnian)-Holocene

First: Mentioned in Wappler and Ben-Dov (2008), Molteno Formation, KwaZulu-Natal, Karoo Basin, South Africa. (This family record is doubtful.)

F. Dictyopharidae K2(Santonian)-Holocene

First: *Netutela annunciator* in Szwedo (2008c), Yantardakh amber, Kheta Formation, Taimyr, Krasnoyarsk Krai, Siberian Federal District, Russian Federation.

F. Dinidoridae Eoc.(Ypresian)-Holocene

First: *Megymenum* sp. in Greenwood et al. (2005), coldwater beds of the Kamloops Group, Quilchena, British Columbia, Canada.

F. Dipsocoridae K1(Barremian)-Holocene

First: Mentioned in Poinar and Milki (2001), Lebanese amber, unknown horizon, unknown locality, Lebanon.

F. Dracaphididae Hong et al., 2009 T2(Ladinian)

First and Last: *Dracaphis angustata* Hong et al., 2009, Tongchuan Formation, Hejiafang, Tongchuan District, Shaanxi Province, China.

F. Drepanochaitophoridae Zhang and Hong, 1999 Eoc.(Ypresian)

First and Last: *Drepanochaitophorus fushunensis* Zhang and Hong, 1999, Fushun amber, Guchengzi, Liaoning Province, China.

F. Dunstaniidae P2(Capitanian)-J3(Tithonian)

First: Mentioned in Shcherbakov (2008d), Tsankhi (Tsankhin) Formation, Bor-Tolgoy, Ömnögovi (South Gobi) Aimag, Mongolia.

Last: Mentioned in Dmitriev and Zherikhin (1988), Ulan-Ereg, Khoutiyn-Khotgor, Dund-Gobi Aimag, Mongolia. (For locality information, see `http://palaeoentomolog.ru/Collections/hutiinhotgor.html`.)

F. Dysmorphoptilidae (Dismorphoptilidae, Eoscartarellidae, Eoscartellidae, Eoscarterellidae, Fulgoringruidae) P1(Artinskian)-J2(Callovian)

First: *Fulgoringruo kukalovae* in Martins-Neto and Gallego (2006), Irati Formation, Paraná Basin, São Paulo, Brazil.

Last: *Dysmorphoptila notodon* in Martins-Neto and Gallego (2006), Togo-Khuduk Member, Bakhar Series, Bayankhongor Aimag, Mongolia.

F. Ebboidae Perrichot et al., 2006 K1(Albian)-K2(Cenomanian)

First: *Ebboa areolata* Perrichot et al., 2006, Archingeay amber, Archingeay-Les Nouillers, Charente-Maritime, France.

Last: *Ebboa areolata* Perrichot et al., 2006, Salignac/Sisteron amber, near Sisteron, Alpes-de-Haute-Provence, France.

F. Electrococcidae Koteja, 2000b K1(Barremian)-K2(Campanian)

First: *Apticoccus minutus* Koteja and Azar, 2008, Hammana/Mdeyrij amber, Caza Baabda, Mouhafazet Jabal Loubnan, Lebanon. (Koteja and Azar 2008 note that placement of this species in Electrococcidae is tentative.)

Last: *Electrococcus canadensis* in Koteja and Azar (2008), Canadian amber, Cedar Lake, Manitoba, Canada. (Originally placed in Pityococcidae, this specimen was transferred to Electrococcidae by Koteja 2000b.)

F. Elektraphididae (Electraphididae) K2(Santonian)-Plio.(Piacenzian)

First: *Tajmyrella cretacea* in Heie and Wegierek (1998), Yantardakh amber, Kheta Formation, Taimyr, Krasnoyarsk Krai, Siberian Federal District, Russian Federation.

Last: *Schizoneurites* sp. in Heie (1985), Willershausen, Harz mountains, Lower Saxony, Germany.

F. Enicocephalidae K1(Hauterivian)-Holocene

First: *Enicocephalinus acragrimaldii* in Azar (2007), Jezzine amber, Jouar Ess-Souss, Mouhafazet Loubnan El-Janoubi, Lebanon.

F. Eriococcidae K2(Turonian)-Holocene

First: e.g.? *Keithia luzzii* Koteja, 2000b, New Jersey amber, South Amboy Fire Clay (Raritan Formation), New Jersey, United States.

F. Eurybrachyidae Eoc.(Lutetian)-Holocene

First: *Amalaberga ostrogothiorum* Szwedo and Wappler, 2006, Messel Formation, Grube Messel, Hesse, Germany.

F. Flatidae (Flattidae) Mio.(Aquitanian)-Holocene
Shcherbakov (2006) rejects '*Lechaea*' *primigenia* (Fur Formation) from Flatidae.

First: Mentioned in Shcherbakov (2006), Mexican amber, Simojovel, Chiapas, Mexico.

F. Fulgoridae K1(Aptian)-Holocene

First: Figured in Szwedo (2007a), Crato Formation, Araripe Basin, Ceará, Brazil.

F. Fulgoridiidae J1(Sinemurian)-J3(Oxfordian)
This is a paraphyletic unit (Bourgoin and Szwedo, 2008).

First: *Fulgoridiella raetica* in Szwedo et al. (2004), Dzhil Formation, Sogyuty, Issyk-Kul, Kyrgyzstan.

Last: *Aulieezidium karatauense* Szwedo and Żyła, 2009, Karabastau Formation, Karatau Range, Tien Shan mountains, Kazakhstan.

F. Gelastocoridae K1(Aptian)-Holocene

First: e.g. *Cratonerthra corinthiana* in Popov and Bechly (2007), Crato Formation, Araripe Basin, Ceará, Brazil.

F. Genaphididae (Genaphidae) J3(Oxfordian)-K1(Berriasian)

First: *Juraphis crassipes* in Heie and Wegierek (1998), Karabastau Formation, Karatau Range, Tien Shan mountains, Kazakhstan.

Last: *Genaphis valdensis* in Heie and Wegierek (1998), Lulworth Formation, Dinton, Vale of Wardour, Wiltshire, United Kingdom.

F. Gerridae K1(Albian)-Holocene

First: *Cretogerris albianus* in Damgaard (2008a), Archingeay amber, Archingeay-Les Nouillers, Charente-Maritime, France.

F. Granulidae T2(Ladinian)

First and Last: *Granulus tongchuanensis* Hong, 1980b, Tongchuan Formation, Hejiafang, Tongchuan District, Shaanxi Province, China.

F. Grimaldiellidae Koteja, 2000b(Grimaldiidae) K2(Turonian)

e.g. *Grimaldiella resinophila* Koteja, 2000b, New Jersey amber, South Amboy Fire Clay (Raritan Formation), New Jersey, United States.

F. Grohnidae Koteja, 2008 Eoc.(Priabonian)

First and Last: *Grohnus eichmanni* Koteja, 2008, Baltic amber.

F. Hadrocoridae J1(Toarcian)
Although listed under *incertae sedis* by Carpenter (1992b), the family has not been synonymised.

First and Last: *Hadrocoris scutellaris* Handlirsch, 1939, Upper Lias, Dobbertin, Mecklenburg-Vorpommern, Germany.

F. Hammanococcidae Koteja and Azar, 2008 K1(Barremian)

e.g. *Hammanococcus setosus* Koteja and Azar, 2008, Hammana/Mdeyrij amber, Caza Baabda, Mouhafazet Jabal Loubnan, Lebanon.

F. Hebridae Mio.(Aquitanian)-Holocene

First: *Stenohebrus glaesarius* in Damgaard (2008a), Mexican amber, Simojovel, Chiapas, Mexico.

F. Hoploridiidae Popov and Shcherbakov, 1991 K1(Valanginian)
Sometimes treated as a subfamily of Karabasiidae. For discussion, see Heads (2008b) and Wang et al. (2009b).

First and Last: *Hoploridium dollingi* in Wang et al. (2009b), Zaza Formation, Baissa, Buryatia, Russian Federation.

F. Hydrometridae K1(Aptian)-Holocene

First: e.g. *Cretaceometra brasiliensis* in Damgaard (2008a), Crato Formation, Araripe Basin, Ceará, Brazil.

F. Hylicellidae T1(Induan)-K1(Barremian)
Although Jell (2004) lists *Eochiliocycla angusta* from the Upper Permian Belmont insect beds of Australia in Hylicellidae, Evans (1956) removed this species. Several sources (e.g. Shcherbakov and Popov 2002 and Shcherbakov 2008a) explicitly state that Hylicellidae first appear in the Triassic. Szwedo (2008d) mentions that this family goes extinct in the mid-Cretaceous.

First: Mentioned in Shcherbakov (2008a), Babiy Kamen', Maltseva/Sosnovaya Fomation, Kuznetsk Basin, Siberian Federal District, Russian Federation.

Last: Mentioned at `http://palaeoentomolog.ru/Collections/bontsagan.html`, Bon-Tsagaan Nuur, Bon-Tsagaan Group, Bayankhongor Aimag, Mongolia.

F. Hypsipterygidae Eoc.(Priabonian)-Holocene

First: *Hypsipteryx hoffeinsorum* Bechly and Wittmann, 2000, Baltic amber.

F. Ignotalidae (Ignatolidae) P3(Wuchiapingian)-T1(Induan)

First: e.g. *Megoniella multinerva* in van Dijk and Geertsema (1999), Normandien (Estcourt) Formation, Beaufort Group, KwaZulu-Natal, Karoo Basin, South Africa.

Last: Mentioned in Shcherbakov (2008a), Bugarikhta Formation, Nizhnyaya Tunguska river, Krasnoyarsk Krai, Siberian Federal District, Russian Federation.

F. Ignotingidae Zhang et al., 2005 K1(Barremian)

First and Last: *Ignotingis mirifica* Zhang et al., 2005, Laiyang Formation, Laiyang County, Shandong Province, China.

F. Ingruidae P1(Kungurian)-P2(Capitanian)

First: e.g. *Scytoneurella major* in Ross and Jarzembowski (1993), Koshelevka Formation, Tshekarda, Ural Mountains, Russian Federation.

Last: e.g. Mentioned in Shcherbakov (2000b), Tsankhi (Tsankhin) Formation, Bor-Tolgoy, Ömnögovi (South Gobi) Aimag, Mongolia.

F. Inkaidae Koteja, 1989 K2(Santonian)

First and Last: *Inka minuta* in Koteja (2000a), Yantardakh amber, Kheta Formation, Taimyr, Krasnoyarsk Krai, Siberian Federal District, Russian Federation.

F. Ipsviciidae T2(Anisian)-K1(Aptian)

First: e.g. Mentioned in Gall and Grauvogel-Stamm (2005), Grès à Voltzia, Bas-Rhin/Moselle, Northern Vosges Mountains, France.

Last: Mentioned in Shcherbakov and Popov (2002), Shar-Tolgoy Formation, Bon-Tsagaan Group, Bayankhongor Aimag, Mongolia. (Locality information for this specimen was kindly provided by Dr Dmitry Shcherbakov [pers. comm., 2011].)

F. Isometopidae Mio.(Aquitanian)-Holocene

First: Mentioned in Solórzano Kraemer (2007), Mexican amber, Simojovel, Chiapas, Mexico.

F. Issidae K2(Campanian)-Holocene
Szwedo et al. (2004) place the Jurassic *Tetragonidium* in Fulgoridiidae and *Elasmocelidium* as Fulgoroidea *incertae sedis*.

First: Mentioned in McKellar et al. (2008), Canadian amber, Grassy Lake, Alberta, Canada.

F. Jersicoccidae Koteja, 2000b K2(Turonian)

First and Last: *Jersicoccus kurthi* Koteja, 2000b, New Jersey amber, South Amboy Fire Clay (Raritan Formation), New Jersey, United States.

F. Karabasiidae J1(Sinemurian)-J3(Tithonian)

First: *Minuta heteropterata* in Wang et al. (2009b), Dzhil Formation, Sogyuty, Issyk-Kul, Kyrgyzstan.

Last: *Karabasia evansi* in Wang et al. (2009b), Glushkovo Formation, Daya, Transbaikalia, Russian Federation.

F. Karajassidae Shcherbakov, 1992 J1(Toarcian)-K1(Hauterivian)

First: Mentioned in Shcherbakov and Popov (2002), Germany. (The locality is not mentioned but presumably the record is from the Upper Lias.)

Last: e.g. *Gurvania inepta* in Ross and Jarzembowski (1993), Gurvan-Eren Formation (Gurvan-Eren), Gurvan-Eren, Khovd Aimag, Mongolia.

F. Kermesidae Eoc.(Priabonian)-Holocene

First: *Sucinikermes kulickae* in Koteja (2000a), Baltic amber.

F. Kinnaridae Mio.(Burdigalian)-Holocene

First: e.g. *Oeclidius browni* Bourgoin and Lefèbvre, 2002, Dominican amber, Cordillera Septentrional, near Santiago, Dominican Republic.

F. Kobdocoridae Popov, 1986 K1(Hauterivian)

First and Last: *Kobdocoris aradinus* Popov, 1986, Gurvan-Eren Formation, Myangad, Khovd Aimag, Mongolia.

F. Kukaspididae Koteja and Poinar, 2001 K1(Albian)

First and Last: *Kukaspis usingeri* Koteja and Poinar, 2001, Alaskan amber, Kuk deposits, Brooks Range, Alaska, United States.

F. Kuwaniidae Eoc.(Priabonian)-Holocene

First: *Hoffeinsia foldii* Koteja, 2008, Baltic amber.

F. Labiococcidae Koteja, 2000b K2(Turonian)

e.g. *Labiococcus joosti* Koteja, 2000b, New Jersey amber, South Amboy Fire Clay (Raritan Formation), New Jersey, United States.

F. Lachnidae Mio.(Langhian)-Holocene

First: e.g. *Stomaphis eupetes* in Wegierek and Peñalver (2002), Vishnevaya Balka, near Senghileevskoye Lake, Stavropol Krai, Russian Federation.

F. Lalacidae Hamilton, 1990 K1(Barremian)-K1(Aptian)

First: *Cretocixius stigmatosus* in Szwedo (2007a), Lushangfen Formation, Jingxi Basin, Beijing Municipality, China.

Last: e.g. *Lalax mutabilis* in Szwedo (2007a), Crato Formation, Araripe Basin, Ceará, Brazil.

F. Largidae K2(Santonian)-Holocene

First: Mentioned in Poinar (1992), Yantardakh amber, Kheta Formation, Taimyr, Krasnoyarsk Krai, Siberian Federal District, Russian Federation.

F. Lebanococcidae Koteja and Azar, 2008 K1(Barremian)

First and Last: *Lebanococcus longiventris* Koteja and Azar, 2008, Hammana/Mdeyrij amber, Caza Baabda, Mouhafazet Jabal Loubnan, Lebanon.

F. Leptaphelocheiridae Polhemus, 2000 J2(Callovian)

First and Last: *Leptaphelocheirus lenticulus* Polhemus, 2000, Todilto Formation (Luciano Mesa Member), Warm Springs site, New Mexico, United States.

F. Leptopodidae Mio.(Aquitanian)-Holocene

First: *Leptosalda chiapensis* in Solórzano Kraemer (2007), Mexican amber, Simojovel, Chiapas, Mexico.

F. Liadopsyllidae (Asientomidae, Lithentomidae) J1(Toarcian)-K1(Barremian)

First: e.g. *Liadopsylla obtusa* Ansorge, 1996a, Upper Lias, Grimmen, Mecklenburg-Vorpommern, Germany.

Last: *Liadopsylla mongolica* Shcherbakov, 1988, Bon-Tsagaan Nuur, Bon-Tsagaan Group, Bayankhongor Aimag, Mongolia.

F. Ligavenidae Hamilton, 1992 T3(Carnian)-K1(Aptian)

First: e.g. *Ligavena prosboloides* in Jell (2004), Blackstone Formation, Ipswich Basin, Queensland, Australia.

Last: *Ligavena gracilipes* in Jell (2004), Koonwarra Fossil Bed (Korumburra Group), South Gippsland, Victoria, Australia.

F. Lithuanicoccidae Koteja, 2008 Eoc.(Priabonian)

e.g. *Lithuanicoccus damzeni* Koteja, 2008, Baltic amber.

F. Lophopidae (Lophophidae) Eoc.(Lutetian)-Holocene
Szwedo et al. (2004) place the Lower Jurassic *Eofulgoridium* in the Fulgoridiidae. *Scoparidea nebulosa*, from the Ypresian Green River Formation, belongs in or close to Issidae (Shcherbakov, 2006).

First: *Baninus thuringiorum* Szwedo and Wappler, 2006, Messel Formation, Grube Messel, Hesse, Germany.

F. Lygaeidae Eoc.(Ypresian)-Holocene
No reliable records are known for Mesozoic occurrences. *Lygaenocoris* belongs to the Pachymeridiidae. Wappler (2003) indicates that the Mesozoic records require revision and questions if they are attributable.

First: e.g. Mentioned in Wappler (2003), Ølst Formation, Limfjord/Mors Peninsula/Fur Island, Jutland, Denmark.

F. Magnacicadiidae T2(Ladinian)

First and Last: *Magnacicadia shenciensis* in Wang et al. (2006b), Tongchuan Formation, Hejiafang, Tongchuan District, Shaanxi Province, China.

F. Malmopsyllidae (Neopsylloididae) J3(Oxfordian)
Shcherbakov and Popov (2002) state that Neopsylloididae is a synonym.

e.g. *Malmopsylla karatavica* in Ross and Jarzembowski (1993), Karabastau Formation, Karatau Range, Tien Shan mountains, Kazakhstan.

F. Margarodidae Eoc.(Priabonian)-Holocene

First: Figured in Weitschat and Wichard (2002), Baltic amber.

F. Matsucoccidae K1(Valanginian)-Holocene

First: e.g. *Eomatsucoccus sukachevae* in Koteja (2000a), Zaza Formation, Baissa, Buryatia, Russian Federation.

F. Membracidae K1(Albian)-Holocene

First: Mentioned in Perrichot (2004), Archingeay amber, Archingeay-Les Nouillers, Charente-Maritime, France.

F. Mesogereonidae T3(Carnian)

e.g. *Mesogereon superbum* in Jell (2004), Blackstone Formation, Ipswich Basin, Queensland, Australia.

F. Mesopentacoridae J1(Toarcian)-K1(Aptian)

First: aff. *Mesopentacoris* sp. in Popov (1990), Upper Lias, Dobbertin, Mecklenburg-Vorpommern, Germany.

Last: *Pauropentacoris macrurata* in Yao et al. (2004), Jiufotang Formation, Beishan, Yixian County, Liaoning Province, China.

F. Mesotrephidae K2(Turonian)

First and Last: *Mesotrephes striata* in Sinitshenkova (2002c), Kzyl-Zhar, Karatau Range, Tien Shan mountains, Kazakhstan.

F. Mesoveliidae (Karanabidae, Karanabiidae) J3(Oxfordian)-Holocene
Damgaard (2008a) preferred not to assign any fossils to this family pending a review of external morphological characters however Szwedo and Żyła (2009) list this family as present in the Karabastau Formation.

First: *Karanabis kiritschenkoi* in Damgaard (2008a), Karabastau Formation, Karatau Range, Tien Shan mountains, Kazakhstan.

F. Mesozoicaphididae Heie *in* Heie and Pike, 1992 K2(Campanian)

e.g. *Mesozoicaphis canadensis* in McKellar et al. (2008), Canadian amber, Grassy Lake, Alberta, Canada.

F. Microphysidae K2(Santonian)-Holocene

First: Mentioned in Poinar (1992), Yantardakh amber, Kheta Formation, Taimyr, Krasnoyarsk Krai, Siberian Federal District, Russian Federation.

F. Mimarachnidae Shcherbakov, 2007c K1(Valanginian)-K2(Turonian)

First: e.g. *Mimarachne mikhailovi* Shcherbakov, 2007c, Zaza Formation, Baissa, Buryatia, Russian Federation.

Last: Mentioned in Szwedo (2008b), Kzyl-Zhar, Karatau Range, Tien Shan mountains, Kazakhstan.

F. Miridae J3(Oxfordian)-Holocene
Shcherbakov (2008c) removed *Mirivena robusta* (Jiulongshan Formation, Daohugou, China) from this family.

First: e.g. *Scutellifer karatavicus* in Herczek and Popov (2001), Karabastau Formation, Karatau Range, Tien Shan mountains, Kazakhstan.

F. Monophlebidae (Monophlebiidae) Eoc.(Priabonian)-Holocene
Although Grimaldi and Engel (2005, p.299) record this family in Lebanese amber, it is not recorded by Koteja and Azar (2008).

First: *Monophlebus irregularis* in Koteja (2000a), Baltic amber.

F. Myerslopiidae K1(Aptian)-Holocene

First: e.g. *Ovojassus concavifer* in Menon et al. (2007), Crato Formation, Araripe Basin, Ceará, Brazil.

F. Nabidae (Velocipedidae, Vetanthocoridae) J1(Sinemurian)-Holocene

First: e.g. *Saldonabis proteus* Shcherbakov, 2008c, Dzhil Formation, Sogyuty, Issyk-Kul, Kyrgyzstan.

F. Naibiidae Shcherbakov, 2007a T3(Carnian)-Pal.(Thanetian)

First: *Coccavus supercubitus* Shcherbakov, 2007a, Madygen Formation, Madygen/Dzhailoucho, south Fergana Valley, Kyrgyzstan.

Last: e.g. *Naibia zherichini* Shcherbakov, 2007a, Sakhalin amber, Lower Due Formation, Starodubskoe, Sakhalin Region, Russian Federation.

F. Naucoridae (Aphlebocoridae, Apopnidae, Saucrolidae) T3(Carnian)-Holocene

First: Mentioned in Shcherbakov (2008a), Cow Branch Formation, Solite quarry, Virginia, United States.

F. Neazoniidae Szwedo, 2007b K1(Hauterivian)-K1(Albian)

First: *Neazonia imprinta* Szwedo, 2007b, Jezzine amber, Jouar Ess-Souss, Mouhafazet Loubnan El-Janoubi, Lebanon.

Last: *Akmazeina santonorum* Szwedo, 2009, Archingeay amber, Archingeay-Les Nouillers, Charente-Maritime, France.

F. Nepidae J3(Tithonian)-Holocene

First: Mentioned in Ponomarenko (1985), Solenhofen Lithographic Limestone, Solenhofen/Eichstadt, Bavaria, Germany.

F. Nogodinidae Pal.(Danian)-Holocene

First: Mentioned in Shcherbakov (2006), Tsagayan Formation, Arkhara locality, Amur Oblast, Russian Federation.

F. Notonectidae T3(Carnian)-Holocene

First: Mentioned in Shcherbakov (2008a), Cow Branch Formation, Solite quarry, Virginia, United States.

F. Ochteridae (Propreocoridae) J1(Sinemurian)-Holocene

First: *Propreocoris maculatus* in Yao et al. (2007), Black Ven Marls, Charmouth, Dorset, United Kingdom.

F. Ortheziidae K1(Hauterivian)-Holocene

First: *Cretorthezia*? sp. in Koteja and Azar (2008), Jezzine amber, Jouar Ess-Souss, Mouhafazet Loubnan El-Janoubi, Lebanon.

F. Oviparosiphidae J1(Toarcian)-K1(Aptian)

First: *Grimmenaphis magnifica* in Grimaldi and Engel (2005), Upper Lias, Grimmen, Mecklenburg-Vorpommern, Germany.

Last: *Sinoviparosiphum lini* in Ren (2002b), Yixian Formation, Liaoning Province, China.

F. Pachymeridiidae (Hypocimicidae, Psychrocoridae, Sisyrocoridae) T3(Rhaetian)-K1(Aptian)

First: "*Pachymerus*" *zucholdi* in Yao et al. (2008), Cotham Member, Lilstock Formation, Penarth Group1, Strensham, Worcestershire, United Kingdom.

Last: e.g. *Cratocoris schechenkoae* in Popov and Bechly (2007), Crato Formation, Araripe Basin, Ceará, Brazil.

F. Palaeoaphididae (Palaeoaphidae) K1(Valanginian)-K2(Campanian)

First: e.g. *Annulaphis mostovskini* Kania and Wegierek, 2008, Zaza Formation, Baissa, Buryatia, Russian Federation.

Last: e.g. *Longiradius foottitti* in McKellar et al. (2008), Canadian amber, Grassy Lake, Alberta, Canada.

F. Palaeoleptidae Poinar and Buckley, 2009 K1(Albian)

First and Last: *Palaeoleptus burmanicus* Poinar and Buckley, 2009, Burmese amber (Burmite), Hukawng Valley, Kachin State, Myanmar.

F. Palaeontinidae (Paleontinidae) T3(Carnian)-K1(Aptian)
*Fletcheriana triassica* is included in Dunstaniidae (Wang et al., 2009c). The Permian species *Palaeocicadopsis chinensis* is based on a cockroach clavus (Wang et al., 2006a).

First: '*Fletcheriana*' *magna* in Wang et al. (2009c), Molteno Formation, KwaZulu-Natal, Karoo Basin, South Africa.

Last: e.g. *Colossocossus giganticus* Menon & Heads *in* Menon et al., 2007, Crato Formation, Araripe Basin, Ceará, Brazil.

F. Paraknightiidae P3(Changhsingian)-T3(Carnian)

First: *Paraknightia magnifica* in Jell (2004), Belmont insect beds, Newcastle Coal Measures, Belmont/Warner's Bay, New South Wales, Australia.

Last: Mentioned in Shcherbakov (2008b), Madygen Formation, Madygen/Dzhailoucho, south Fergana Valley, Kyrgyzstan.

F. Parvaverrucosidae Poinar and Brown, 2006(Verrucosidae) K1(Albian)

First and Last: *Parvaverrucosa annulata* in Poinar and Brown (2006), Burmese amber (Burmite), Hukawng Valley, Kachin State, Myanmar.

F. Pennygullaniidae Koteja and Azar, 2008 K1(Barremian)

e.g. *Pennygullania electrina* Koteja and Azar, 2008, Hammana/Mdeyrij amber, Caza Baabda, Mouhafazet Jabal Loubnan, Lebanon.

F. Pentatomidae Pal.(Thanetian)-Holocene

First: Mentioned in Wappler (2003), France. (Wappler 2003 does not mention the locality but presumably it is Menat.)

F. Pereboriidae (Pereboridae) P1(Artinskian)-K1(Barremian)

First: *Gondwanoptera capsii* in Martins-Neto (2005), Irati Formation, Paraná Basin, São Paulo, Brazil.

Last: e.g. *Jiphara wangi* in Wang et al. (2006b), Lushangfen Formation, Jingxi Basin, Beijing Municipality, China.

F. Perforissidae Shcherbakov, 2007b K1(Barremian)-K2(Santonian)

First: *Tsaganema oshanini* Shcherbakov, 2007b, Khurilt Formation, Bon-Tsagaan Group, Bayankhongor Aimag, Mongolia.

Last: e.g. *Cixitettix yangi* Shcherbakov, 2007b, Yantardakh amber, Kheta Formation, Taimyr, Krasnoyarsk Krai, Siberian Federal District, Russian Federation.

F. Phylloxeridae Mio.(Aquitanian)-Holocene

First: Mentioned in Engel (2004a), Mexican amber, Simojovel, Chiapas, Mexico.

F. Piesmatidae (Piesmidae) K1(Albian)-Holocene

First: *Cretopiesma suukyiae* Grimaldi and Engel, 2008b, Burmese amber (Burmite), Hukawng Valley, Kachin State, Myanmar.

F. Pincombeidae (Pincombaeidae) P3(Changhsingian)-T3(Carnian)

First: e.g. *Pincombea mirabilis* in Jell (2004), Belmont insect beds, Newcastle Coal Measures, Belmont/Warner's Bay, New South Wales, Australia.

Last: *Madygenopsyllidium djailautshoense* in Shcherbakov (2007a), Madygen Formation, Madygen/Dzhailoucho, south Fergana Valley, Kyrgyzstan.

F. Pityococcidae Eoc.(Priabonian)-Holocene
*Electrococcus canadensis* was transferred to the Electrococcidae by Koteja (2000b).

First: *Cancerococcus apterus* in Koteja and Azar (2008), Baltic amber. (Foldi 2005 lists this species as the only fossil record of Coelostomidiidae.)

F. Plokiophilidae K2(Campanian)-Holocene

First: Mentioned in Popov (2008), Canadian amber, unspecified, Alberta, Canada.

F. Probascaniidae (Probascanionidae) J1(Toarcian)

e.g. *Probascanion megacephalum* in Popov (1992), Upper Lias, Dobbertin, Mecklenburg-Vorpommern, Germany.

F. Procercopidae (Procercopoidae) J1(Hettangian)-K1(Aptian)
Often cited as originating in the Triassic but the supposed Triassic records are from the Lower Jurassic Dzhil Formation. See http://palaeoentomolog.ru/Collections/jur_i.html. Wang et al. (2006b) list *Cretocercopis* as K2 but this must be a mistake as it is from the Lushangfen Formation, which is Lower Cretaceous.

First: e.g. *Procercopis shawanensis* Zhang et al., 2004, Badaowan Formation, Kelamayi, Xinjiang Uyghur Autonomous Region, China.

Last: e.g. *Anomoscytina anomola* Ren et al., 1998, Jianshangou beds, Yixian Formation, Liaoning Province, China.

F. Progonocimicidae (Actinescytinidae, Actinoscytinidae, Cicadocoridae, Eocimicidae, Progonomicidae) P3(Changhsingian)-K1(Aptian)

First: *Actinoscytina belmontensis* in Jell (2004), Belmont insect beds, Newcastle Coal Measures, Belmont/Warner's Bay, New South Wales, Australia.

Last: e.g. Mentioned in Bechly and Szwedo (2007), Crato Formation, Araripe Basin, Ceará, Brazil.

F. Prosbolidae (Cicadopsyllidae, Permocicadopsidae, Permoglyphidae, Prosbolecicadidae, Sojanoneuridae) P1(Artinskian)-K1(Valanginian)
Martins-Neto (2005) lists *Prosbolecicada gondwanica* in Dysmorphoptilidae, probably by mistake; indeed, Martins-Neto and Gallego (2006) do not mention it in their review of the family. Shcherbakov (2000b) synonymised Prosbolecicadidae under Prosbolidae.

First: e.g. *Prosbole iratiensis* in Martins-Neto (2005), Irati Formation, Paraná Basin, São Paulo, Brazil.

Last: *Longimaxilla sinica* in Wang et al. (2006b), Chijinqiao (=Chijinpu) Formation, Xiagou, Jiuquan Basin, Gansu Province, China.

F. Prosbolopseidae (Ivaiidae, Mundidae, Prosbolopsidae) P1(Kungurian)-P2(Capitanian)

First: e.g. *Cicadopsis*? sp. in Shcherbakov et al. (2009), Pospelovo Formation, Russky Island, Primorye, Russian Federation.

Last: Mentioned in Shcherbakov (2000b), Tsankhi (Tsankhin) Formation, Bor-Tolgoy, Ömnögovi (South Gobi) Aimag, Mongolia.

F. Protocoridae J1(Hettangian)-J1(Toarcian)
*Pallicoris* from the Shiti Formation in Guangxi, China, belongs to the Pachymeridiidae (Popov et al., 1994).

First: e.g. *Protocoris indistinctus* Popov et al., 1994, Lower Lias, Binton, Warwickshire, United Kingdom.

Last: Mentioned in Popov et al. (1994), Upper Lias, Dobbertin, Mecklenburg-Vorpommern, Germany.

F. Protopsyllidiidae (Eopsyllidiidae, Permaleurodidae, Permaleyrodidae, Permaphidopseidae, Permopsyllidae) P1(Kungurian)-K2(Turonian)
*Permaleurodes* and *Aleuronympha* (Permaleurodidae) probably belong to this family, or a related group of Psyllinea according to Shcherbakov (2000a).

First: Mentioned in Geertsema et al. (2002), carbonaceous shales, middle Ecca Group, Haakdoornfontein, near Pretoria, South Africa.

Last: *Postopsyllidium emilyae* Grimaldi, 2003a, New Jersey amber, South Amboy Fire Clay (Raritan Formation), New Jersey, United States.

F. Pseudococcidae Eoc.(Priabonian)-Holocene

First: Mentioned in Koteja (2000a), Baltic amber.

F. Pseudonerthridae Martins-Neto & Pérez Goodwyn Martins-Neto & Perez Good *in* López Ruf et al., 2005 K1(Aptian)

First and Last: *Pseudonerthra gigantea* in Popov and Bechly (2007), Crato Formation, Araripe Basin, Ceará, Brazil.

F. Psyllidae K1(Aptian)-Holocene

First: Figured in Jell (2004), Koonwarra Fossil Bed (Korumburra Group), South Gippsland, Victoria, Australia.

F. Pterocimicidae J1(Sinemurian)

First and Last: *Pterocimex jacksoni* in Popov et al. (1994), Black Ven Marls, Charmouth, Dorset, United Kingdom.

F. Putoidae K1(Barremian)-Holocene

First: *Palaeotupo danieleae* Koteja and Azar, 2008, Hammana/Mdeyrij amber, Caza Baabda, Mouhafazet Jabal Loubnan, Lebanon. (Koteja and Azar 2008 note that placement of this species in Putoidae is tentative.)

F. Pyrrhocoridae Eoc.(Priabonian)-Holocene
*Mesopyrrhocoris fasciata* from the Lower Cretaceous Laiyang Formation is Cimicomorpha *incertae sedis*, according to Shcherbakov (2008c).

First: e.g. *Dysdercus cinctus* in Meyer (2003), Florissant Formation, Florissant, Colorado, United States.

F. Reduviidae (Phymatidae, Reduvidae) K1(Albian)-Holocene
*Liaoxia longa* from the Lower Cretaceous Jiufotang Formation is now placed in Nabidae: Vetanthocorini (Yao et al., 2006a; Shcherbakov, 2008c).

First: Mentioned in Poinar and Poinar (2008), Burmese amber (Burmite), Hukawng Valley, Kachin State, Myanmar.

F. Rhinocolidae Eoc.(Priabonian)-Holocene
Sometimes treated as a subfamily of Psyllidae but kept separate in Pérez-Gelabert (2008).

First: *Protoscena baltica* in Klimaszewski (1997), Baltic amber. (This species was mistakenly listed by Weitschat and Wichard 2002 under 'Paleoaphalaridae' [=Aphalaridae: Palaeoaphalarinae].)

F. Rhinopsyllidae (Rhynopsyllidae) Mio.(Burdigalian)-Holocene

First: e.g. *Rhinopsyllida acutealla* in Pérez-Gelabert (2008), Dominican amber, Cordillera Septentrional, near Santiago, Dominican Republic.

F. Rhopalidae J2(Callovian)-Holocene

First: e.g. *Originicorizus pyriformis* Yao, Cai & Ren *in* Yao et al., 2006b, Jiulongshan Formation, near Daohugou, Ningcheng county, Inner Mongolia, China.

F. Ricaniidae Pal.(Thanetian)-Holocene
Szwedo et al. (2004) do not consider that the Mesozoic genera *Qiyangiricania* and *Ricaniites* belong to this family.

First: *Scolypopites bryani* in Jell (2004), Redbank Plains Formation, Ipswich Basin, Queensland, Australia.

F. Saldidae (Enicocoridae, Mesolygaeidae, Xishanidae) K1(Barremian)-Holocene

First: *Mesolygaeus laiyangensis* in Zhang et al. (2005), Laiyang Formation, Laiyang County, Shandong Province, China.

F. Scaphocoridae J3(Oxfordian)

First and Last: *Scaphocoris notatus* in Carpenter (1992b), Karabastau Formation, Karatau Range, Tien Shan mountains, Kazakhstan.

F. Schizopteridae K1(Barremian)-Holocene

First: Mentioned in Grimaldi and Engel (2005), Lebanese amber, unknown horizon, unknown locality, Lebanon.

F. Scutelleridae Eoc.(Ypresian)-Holocene

First: Mentioned in Rust (1998), Fur Formation (Mo Clay), Limfjord/Mors Peninsula/Fur Island, Jutland, Denmark.

F. Scytinopteridae (Seytinopteridae) C2(Gzhelian)-K1(Barremian)

First: Mentioned in Shcherbakov (2000b), Bursum Formation (Red Tanks Member), Carrizo Arroyo, New Mexico, United States. (Rasnitsyn in Shcherbakov 2000b considers the attribution of this specimen, referred to by Rowland 1997, to be doubtful.)

Last: *Sunoscytinopteris lushangfenensis* in Wang et al. (2006b), Lushangfen Formation, Jingxi Basin, Beijing Municipality, China.

F. Serafinidae Koteja, 2008 Eoc.(Priabonian)

First and Last: *Serafinus acutipterus* Koteja, 2008, Baltic amber.

F. Serpentivenidae (Serpenivenidae, Serpentiveniidae) P2(Wordian)-T3(Carnian)

First: Mentioned in Aristov and Bashkuev (2008), Chepanikha locality, Rossokha River valley, Zavjalovskii District, Udmurt Republic, Russian Federation.

Last: e.g. *Serpentivena tigrina* in Ross and Jarzembowski (1993), Madygen Formation, Madygen/Dzhailoucho, south Fergana Valley, Kyrgyzstan.

F. Shaposhnikoviidae J2(Aalenian)-K2(Santonian)

First: *Tinaphis sibirica* Wegierek, 1989, Itat Formation, Kubekovo, Krasnoyarsk Krai, Siberian Federal District, Russian Federation.

Last: *Shaposhnikovia electri* in Heie (1987), Yantardakh amber, Kheta Formation, Taimyr, Krasnoyarsk Krai, Siberian Federal District, Russian Federation.

F. Shurabellidae (Shuraveliidae) J1(Hettangian)-J3(Oxfordian)

First: *Shurabella lepyroniopsis*? in Shcherbakov (2008b), unnamed deposit overlying Madygen Formation, Madygen/Dzhailoucho, south Fergana Valley, Kyrgyzstan.

Last: *Shurabella* sp. in Grimaldi and Engel (2005), Karabastau Formation, Karatau Range, Tien Shan mountains, Kazakhstan.

F. Simulaphididae Shcherbakov, 2007a P3(Changhsingian)-T3(Norian)

First: *Simulaphis shaposhnikovi* Shcherbakov, 2007a, Belmont insect beds, Newcastle Coal Measures, Belmont/Warner's Bay, New South Wales, Australia.

Last: Mentioned in Shcherbakov (2007a), Protopivka Formation, Garazhovka, Izyum District, Ukraine. (This record is doubtful.)

F. Sinojuraphididae Huang and Nel, 2008 J2(Callovian)

First and Last: *Sinojuraphis ningchengensis* Huang and Nel, 2008, Jiulongshan Formation, near Daohugou, Ningcheng county, Inner Mongolia, China.

F. Steingeliidae K1(Barremian)-Holocene

First: e.g. *Palaeosteingelia acrai* Koteja and Azar, 2008, Hammana/Mdeyrij amber, Caza Baabda, Mouhafazet Jabal Loubnan, Lebanon.

F. Stenoviciidae P2(Capitanian)-K1(Barremian)

First: Mentioned in Shcherbakov (2000b), Tsankhi (Tsankhin) Formation, Bor-Tolgoy, Ömnögovi (South Gobi) Aimag, Mongolia.

Last: Mentioned in Poinar and Milki (2001), Lebanese amber, unknown horizon, unknown locality, Lebanon.

F. Surijokocixiidae Shcherbakov, 2000b(Surijokocixidae) P2(Wordian)-T3(Carnian)

First: e.g. *Surijokocixius tomiensis* in Szwedo et al. (2004), IlŠinskoe Formation, Suriyokova (Suriekova), Kemerovo Region, Russian Federation.

Last: e.g. *Tricrosbia minuta* in Szwedo et al. (2004), Mount Crosby Formation, Ipswich Basin, Queensland, Australia.

F. Tajmyraphididae (Taimyraphididae, Taymiraphididae) K1(Barremian)-K2(Campanian)

First: e.g. *Megarostrum azari* Heie *in* Heie and Azar, 2000, Hammana/Mdeyrij amber, Caza Baabda, Mouhafazet Jabal Loubnan, Lebanon.

Last: *Grassyaphis pikei* in McKellar et al. (2008), Canadian amber, Grassy Lake, Alberta, Canada.

F. Termitaphididae (Termitiaphididae) Mio.(Aquitanian)-Holocene
Grimaldi and Engel (2008a) suggest that this family may belong within Aradidae.

First: *Termitaradus protera* in Engel (2009b), Mexican amber, Simojovel, Chiapas, Mexico.

F. Tettigarctidae (Cicadoprosbolidae, Protabanidae, Tettigarcitidae) T3(Rhaetian)-Holocene

First: '*Liassocicada*' *ignotata* in Shcherbakov (2009), Cotham Member, Lilstock Formation, Penarth Group1, Strensham, Worcestershire, United Kingdom.

F. Thaumastellidae (Thaumestellidae) K1(Barremian)-Holocene
Considered by Shcherbakov and Popov (2002) to be a subfamily of Cydnidae, family status is maintained here after Grazia et al. (2008).

First: Mentioned in Poinar and Milki (2001), Lebanese amber, unknown horizon, unknown locality, Lebanon.

F. Thaumastocoridae K2(Turonian)-Holocene

First: Mentioned in Golub and Popov (2000), New Jersey amber, South Amboy Fire Clay (Raritan Formation), New Jersey, United States.

F. Thelaxidae Eoc.(Priabonian)-Holocene

First: *Palaeothelaxes setosa* in Carpenter (1992b), Baltic amber.

F. Tingidae (Cantacaderidae) K1(Valanginian)-Holocene

First: *Sinaldocader ponomarenkoi* Golub and Popov, 2008, Zaza Formation, Baissa, Buryatia, Russian Federation.

F. Triassoaphididae Heie, 1999(Triassoaphidae) T3(Carnian)

First and Last: *Triassoaphis cubitus* in Hong et al. (2009), Mount Crosby Formation, Ipswich Basin, Queensland, Australia. (Jell 2004 mistakenly lists this species in Aphididae.)

F. Triassocoridae T3(Carnian)-T3(Norian)

First: e.g. *Triassocoris myersi* in Jell (2004), Blackstone Formation, Ipswich Basin, Queensland, Australia.

Last: Mentioned in Shcherbakov and Popov (2002), Tologoy Formation, Ak-Kolka River, Kenderlyk, Zaisan District, Kazakhstan.

F. Triozidae Mio.(Burdigalian)-Holocene

First: e.g. *Trioacantha indocilia* in Arillo and Ortuño (2005), Dominican amber, Cordillera Septentrional, near Santiago, Dominican Republic.

F. Trisegmentatidae Zhang et al., 1994 Mio.(Langhian)

First and Last: *Trisegmentatus onymus* Zhang et al., 1994, Shanwang Formation, Linqu County, Shandong Province, China.

F. Tropiduchidae K2(Turonian)-Holocene

First: Mentioned in Szwedo (2009), Orapa diamond mines, Orapa, Orapa, Botswana. (Locality data provided by J. Szwedo pers. comm., 2011.)

F. Urostylididae Mio.(Langhian)-Holocene
Name changed by Berger et al. (2001) to correct the spelling and remove homonymy with Ciliophora: Urostylidae Bütschli, 1889.

First: e.g. *Urochela pardalina* in Yao et al. (2004), Shanwang Formation, Linqu County, Shandong Province, China.

F. Veliidae K1(Aptian)-Holocene
This family is paraphyletic with respect to Gerridae (Damgaard, 2008b).

First: Figured in Jell (2004), Koonwarra Fossil Bed (Korumburra Group), South Gippsland, Victoria, Australia. (Familial assignment of this fossil form remains provisional until further specimens are found, according to Andersen 1998.)

F. Vianaididae K2(Turonian)-Holocene

First: e.g. *Vianathauma pericarti* Golub and Popov, 2003, New Jersey amber, South Amboy Fire Clay (Raritan Formation), New Jersey, United States.

F. Weitschatidae Koteja, 2008 Eoc.(Priabonian)

e.g. *Weitschatus stigmatus* Koteja, 2008, Baltic amber.

F. Xylococcidae (Xyloccidae) K1(Valanginian)-Holocene

First: *Baisococcus victoriae* in Koteja (2000a), Zaza Formation, Baissa, Buryatia, Russian Federation.

**O. Phthiraptera** Haeckel, 1896 Palaeogene(Lutetian)-Quaternary(Holocene)

In reviewing purported fossils of this order, Dalgleish et al. (2006) reject all but two known specimens as belonging to Phthiraptera: a menoponid from the Eckfeld maar (see below) and eggs from the Baltic amber, however they did not consider Pleistocene (sub)fossils. The ordinal placement of Saurodectidae from the Zaza Formation remains unknown. The extinct families Mammalophagidae Kumar, 2004 and Khatamamammalophagidae Kumar, 2004 are now considered to be Acari (mites) (Dalgleish et al., 2006; Smith et al., 2007).

F. Menoponidae Eoc.(Lutetian)-Holocene

First: *Megamenopon rasnitsyni* Wappler et al., 2004, Eckfeld maar, Manderscheid, Rhineland-Palatinate, Germany.

F. Polyplacidae Pleist.(Upper Pleistocene)-Holocene

First: e.g. *Neohaematopinus relictus* in Mey (2005), permafrost, Indigirka, Sakha (Yakutia) Republic, Russian Federation. (Labandeira 1994 listed this occurrence under the family Hoplopleuridae.)

**O. Psocoptera** (Anoplura, Corrodentia, Mallophaga, Psocida)
Jurassic(Toarcian)-Quaternary(Holocene)

A paraphyletic group comprising all Psocodea except the Phthiraptera. Taxonomic system after the Psocodea Species File (Version 1.1/40).

F. Amphientomidae K2(Santonian)-Holocene
The specimens mentioned by Rasnitsyn (2002f) as "Amphientomidae: Electrentominae" from the Upper Jurassic Karabastau Formation (considered here as the separate family Electrentomidae [=Manicapsocidae]) belong to the Paramesopsocidae Azar et al., 2008.

First: *Proamphientomum cretaceum* in Nel et al. (2005f), Yantardakh amber, Kheta Formation, Taimyr, Krasnoyarsk Krai, Siberian Federal District, Russian Federation.

F. Amphipsocidae (Polypsocidae) Eoc.(Priabonian)-Holocene
The Jordanian amber record figured in Kaddumi (2005) is doubtful.

First: *Kolbia ava* in Lienhard and Smithers (2002), Baltic amber.

F. Arcantipsocidae Azar et al., 2009 K1(Albian)

First and Last: *Arcantipsocus courvillei* Azar et al., 2009, Archingeay amber, Archingeay-Les Nouillers, Charente-Maritime, France.

F. Archaeatropidae Baz and Ortuño, 2000(Archaetropidae) K1(Albian)
This family may also occur in Lower Cretaceous French and Lebanese amber (see Perrichot et al., 2003; Azar and Nel, 2004).

First and Last: *Archaeatropos alavensis* Baz and Ortuño, 2000, Álava amber, Escucha Formation, Basco-Cantabrian Basin, Álava Province, Spain.

F. Archipsocidae Eoc.(Ypresian)-Holocene

First: *Archipsocus* cf. *puber* in Brasero et al. (2009), Oise amber, Le Quesnoy, Houdancourt, Oise, Picardie, France.

F. Archipsyllidae J1(Toarcian)-K1(Barremian)
Considered by Grimaldi and Engel (2005) to be stem Paraneoptera, Huang et al. (2008a) demonstrated that Archipsyllidae are Psocoptera. Permian records of this family are erroneous (Rasnitsyn, 2002f).

First: *Archipsylla primitiva* in Nel et al. (2005f), Upper Lias, Grimmen, Mecklenburg-Vorpommern, Germany.

Last: Mentioned in Rasnitsyn (2002f), Bon-Tsagaan Nuur, Bon-Tsagaan Group, Bayankhongor Aimag, Mongolia.

F. Caeciliusidae (Caeciliidae) Eoc.(Ypresian)-Holocene

First: e.g. *Eopsocites fushunensis* Hong, 2002a, Fushun amber, Guchengzi, Liaoning Province, China.

F. Cladiopsocidae Mio.(Burdigalian)-Holocene

First: *Cladiopsocus* sp. in Pérez-Gelabert (2008), Dominican amber, Cordillera Septentrional, near Santiago, Dominican Republic.

F. Compsocidae K1(Albian)-Holocene

First: *Burmacompsocus perreaui* Nel and Waller, 2007, Burmese amber (Burmite), Hukawng Valley, Kachin State, Myanmar.

F. Dolabellopsocidae (Dolabellapsocidae) Mio.(Burdigalian)-Holocene

First: *Isthmopsocus* sp. in Pérez-Gelabert (2008), Dominican amber, Cordillera Septentrional, near Santiago, Dominican Republic.

F. Ectopsocidae Mio.(Aquitanian)-Holocene

First: *Ectopsocus* sp. in Engel (2004a), Mexican amber, Simojovel, Chiapas, Mexico.

F. Electrentomidae (Manicapsocidae) K1(Albian)-Holocene
Preference of family name after the Psocoptera Species File (Version 1.1/4.0).

First: *Manicapsocidus enigmaticus* in Delclòs et al. (2007), Álava amber, Escucha Formation, Basco-Cantabrian Basin, Álava Province, Spain.

F. Elipsocidae J3(Oxfordian)-Holocene

First: Mentioned in Grimaldi and Engel (2005), Karabastau Formation, Karatau Range, Tien Shan mountains, Kazakhstan. (Grimaldi and Engel 2005 list this occurrence as Psocidae however Rasnitsyn 2002f lists it as in the tribe Elipsocini, which would place it in the family Elipsocidae in the present classification.)

F. Empheriidae K1(Albian)-Eoc.(Priabonian)
Formerly considered a subfamily of Trogiidae (Baz and Ortuño, 2001).

First: e.g. *Empheropsocus arilloi* Baz and Ortuño, 2001, Álava amber, Escucha Formation, Basco-Cantabrian Basin, Álava Province, Spain.

Last: e.g. *Trichempheria villosa* in Engel and Perkovsky (2006), Rovno amber, Klesov/Dubrovitsa, Rivne Oblast, Ukraine.

F. Epipsocidae Pal.(Thanetian)-Holocene

First: Mentioned in Rasnitsyn (2002f), Sakhalin amber, Lower Due Formation, Starodubskoe, Sakhalin Region, Russian Federation.

F. Hemipsocidae Mio.(Burdigalian)-Holocene

First: *Hemipsocus* sp. in Pérez-Gelabert (2008), Dominican amber, Cordillera Septentrional, near Santiago, Dominican Republic.

F. Lachesillidae K2(Santonian)-Holocene

First: *Archaelachesis granulosa* in Nel et al. (2005f), Yantardakh amber, Kheta Formation, Taimyr, Krasnoyarsk Krai, Siberian Federal District, Russian Federation. (Nel et al. 2005f suggest that this species may not belong in this family, in which case *Eolachesilla eocenica* from the Oise amber would be the first occurrence.)

F. Lepidopsocidae Eoc.(Ypresian)-Holocene

First: *Thylacella eocenica* Nel et al., 2005f, Oise amber, Le Quesnoy, Houdancourt, Oise, Picardie, France.

F. Liposcelididae (Liposcelidae) K1(Albian)-Holocene

First: *Cretoscelis burmitica* Grimaldi and Engel, 2006b, Burmese amber (Burmite), Hukawng Valley, Kachin State, Myanmar.

F. Mesopsocidae Mio.(Burdigalian)-Holocene
Rasnitsyn (2002f, fig.163) assigns an undescribed specimen from the Upper Jurassic of Karatau to this family, however Azar et al. (2008) identify it as *Paramesopsocus adibi* (Paramesopsocidae).

First: *Mesopsocus* sp. in Peñalver et al. (1996), Ribesalbes, La Rinconada site, Ribesalbes-Alcora, Castellón Province, Spain.

F. Myopsocidae Mio.(Aquitanian)-Holocene

First: *Myopsocus* sp. in Solórzano Kraemer (2007), Mexican amber, Simojovel, Chiapas, Mexico.

F. Pachytroctidae K1(Albian)-Holocene
Although Nel et al. (2005f) removed *Psylloneura*? *perantiqua* (Burmese amber) from this family, a second unnamed specimen identified as belonging to this family remains.

First: Mentioned in Rasnitsyn and Ross (2000), Burmese amber (Burmite), Hukawng Valley, Kachin State, Myanmar.

F. Paramesopsocidae Azar et al., 2008 J3(Oxfordian)-K1(Barremian)

First: *Paramesopsocus adibi* Azar et al., 2008, Karabastau Formation, Karatau Range, Tien Shan mountains, Kazakhstan.

Last: *Paramesopsocus lu* Azar et al., 2008, Hammana/Mdeyrij amber, Caza Baabda, Mouhafazet Jabal Loubnan, Lebanon.

F. Peripsocidae Olig.(Chattian)-Holocene

First: Mentioned in Krumbiegel (1997), Bitterfeld amber, Bitterfeld, Saxony-Anhalt, Germany.

F. Philotarsidae Eoc.(Priabonian)-Holocene

First: e.g. *Philotarsopsis antiquus* in Mockford (2007), Baltic amber.

F. Prionoglarididae (Prionoglariidae) K1(Barremian)-Holocene

First: Figured in Grimaldi and Engel (2005), Lebanese amber, unknown horizon, unknown locality, Lebanon.

F. Pseudocaeciliidae (Pseudocaecilliidae) Eoc.(Priabonian)-Holocene

First: *Electropsocus unguidens* in Lienhard and Smithers (2002), Baltic amber.

F. Psocidae Eoc.(Priabonian)-Holocene

First: e.g. *Psocidus multiplex* in Engel and Perkovsky (2006), Rovno amber, Klesov/Dubrovitsa, Rivne Oblast, Ukraine.

F. Psoquillidae Eoc.(Ypresian)-Holocene

First: *Eorhyopsocus magnificus* Nel et al., 2005f, Oise amber, Le Quesnoy, Houdancourt, Oise, Picardie, France.

F. Psyllipsocidae K1(Albian)-Holocene

First: *Psyllipsocus*? *banksi* in Ross and York (2000), Burmese amber (Burmite), Hukawng Valley, Kachin State, Myanmar. (Nel et al. 2005f question the position of this species but do not remove it from from this family. *Parapsyllipsocus vergereaui* Perrichot et al. 2003 may also belong to this family.)

F. Ptiloneuridae Mio.(Burdigalian)-Holocene

First: Mentioned in Rasnitsyn (2002f), Dominican amber, Cordillera Septentrional, near Santiago, Dominican Republic.

F. Sphaeropsocidae K1(Hauterivian)-Holocene

First: *Sphaeropsocites lebanensis* Grimaldi and Engel, 2006a, Jezzine amber, Jouar Ess-Souss, Mouhafazet Loubnan El-Janoubi, Lebanon.

F. Spurostigmatidae Mio.(Burdigalian)-Holocene
Family reinstated by Casasola González (2006).

First: *Spurostigma* sp. in Pérez-Gelabert (2008), Dominican amber, Cordillera Septentrional, near Santiago, Dominican Republic. (This genus is listed by Pérez-Gelabert 2008 under Cladiopsocidae, however it is maintained in a separate family in the Psocodea Species File.)

F. Trichopsocidae Eoc.(Priabonian)-Holocene

First: *Palaeopsocus tener* in Lienhard and Smithers (2002), Baltic amber.

F. Troctopsocidae Mio.(Burdigalian)-Holocene

First: e.g. *Troctopsocopsis* sp. in Solórzano Kraemer (2007), Dominican amber, Cordillera Septentrional, near Santiago, Dominican Republic.

F. Trogiidae K1(Albian)-Holocene

First: Mentioned in Poinar and Poinar (2008), Burmese amber (Burmite), Hukawng Valley, Kachin State, Myanmar.

**O. Thysanoptera** Haliday, 1836 (Thripida) Triassic(Carnian)-Quaternary(Holocene)

The classification supported by Mound and Morris (2007) is followed here. Grimaldi et al. (2004) mention that *Permothrips longipennis* (Koshelevka Formation, Kungurian) is probably an archescytinid, so no definitive Palaeozoic thrips are known. Poinar and Poinar (2008) (plate 3, fig.A) figure a thrips under the name 'Ectinothripidae'. This family name has yet to be published (G. O. Poinar, Jr. pers. comm., 2011) so is not included here.

F. Adiheterothripidae (Neocomothripidae, Opadothripidae, Rhetinothripidae, Scaphothripidae, Scudderothripidae, Stenurothripidae) K1(Barremian)-Holocene

First: e.g. *Exitelothrips mesozoicus* in Poinar and Milki (2001), Lebanese amber, unknown horizon, unknown locality, Lebanon.

F. Aeolothripidae (Aeolopthripidae, Aeothripidae, Palaeothripidae) K1(Valanginian)-Holocene

First: *Fusithrips crassipes* Shmakov, 2009, Zaza Formation, Baissa, Buryatia, Russian Federation.

F. Heterothripidae Eoc.(Priabonian)-Holocene

First: e.g. *Heterothrips nani* Schliephake, 2001, Baltic amber.

F. Karataothripidae J3(Oxfordian)

First and Last: *Karataothrips jurassicus* in Shmakov (2008), Karabastau Formation, Karatau Range, Tien Shan mountains, Kazakhstan.

F. Liassothripidae J3(Oxfordian)

First and Last: *Liassothrips crassipes* in Shmakov (2008), Karabastau Formation, Karatau Range, Tien Shan mountains, Kazakhstan.

F. Melanthripidae Eoc.(Ypresian)-Holocene
Formerly a subfamily in Aeolothripidae.

First: Mentioned in Brasero et al. (2009), Oise amber, Le Quesnoy, Houdancourt, Oise, Picardie, France.

F. Merothripidae (Jezzinothripidae) K1(Barremian)-Holocene

First: *Jezzinothrips cretacicus* in Poinar and Milki (2001), Lebanese amber, unknown horizon, unknown locality, Lebanon.

F. Moundthripidae Nel et al., 2007b K1(Hauterivian)-K1(Barremian)
Shmakov (2009) thinks this might belong in Lophioneuridae.

First: *Moundthrips beatificus* Nel et al., 2007b, Jezzine amber, Jouar Ess-Souss, Mouhafazet Loubnan El-Janoubi, Lebanon.

Last: *Moundthrips beatificus* Nel et al., 2007b, Hammana/Mdeyrij amber, Caza Baabda, Mouhafazet Jabal Loubnan, Lebanon.

F. Phlaeothripidae (Phloeothripidae) Eoc.(Ypresian)-Holocene
Both Zherikhin (2002a) and Grimaldi and Engel (2005) state that the oldest Phlaeothripidae are from the Eocene Baltic amber, thus implying that the Siberian amber record of this family in Spahr (1992) was erroneous. Dr Alexey Shmakov (pers. comm., 2011) has confirmed this.

First: Mentioned in Brasero et al. (2009), Oise amber, Le Quesnoy, Houdancourt, Oise, Picardie, France.

F. Thripidae K1(Valanginian)-Holocene

First: *Convexithrips robustus* Shmakov, 2009, Zaza Formation, Baissa, Buryatia, Russian Federation.

F. Triassothripidae Grimaldi & Shmakov *in* Grimaldi et al., 2004 T3(Carnian)-T3(Norian)

First: *Triassothrips virginicus* Grimaldi & Fraser *in* Grimaldi et al., 2004, Cow Branch Formation, Solite quarry, Virginia, United States.

Last: *Kazachothrips triassicus* Shmakov *in* Grimaldi et al., 2004, Tologoy Formation, Ak-Kolka River, Kenderlyk, Zaisan District, Kazakhstan.

**Paraneoptera** *incertae sedis*

F. Lophioneuridae (Edgariekiidae) P1(Artinskian)-K2(Campanian)
Generally considered to be a paraphyletic stem-group of Thysanoptera (e.g. Grimaldi and Engel, 2005) however this relationship is questioned by Mound and Morris (2007).

First: e.g. *Cyphoneurodes patriciae* Beckemeyer, 2004a, Wellington Formation, Midco, Oklahoma, United States.

Last: Mentioned in McKellar et al. (2008), Canadian amber, Grassy Lake, Alberta, Canada.

F. Permopsocidae P1(Sakmarian)-P1(Artinskian)

First: Mentioned in Rasnitsyn (2002f), Obora locality, Bačov Beds, Letovice Formation, Moravia, Czech Republic.

Last: e.g. *Permopsocus ovatus* in Beckemeyer (2000), Wellington Formation, Elmo site, Dickinson County, Kansas, United States.

F. Psocidiidae (Dichentomidae) C2(Gzhelian)-J1(Toarcian)

First: e.g. *Dichentomum*? *arroyo* Rasnitsyn *in* Rasnitsyn et al., 2004a, Bursum Formation (Red Tanks Member), Carrizo Arroyo, New Mexico, United States.

Last: *Liassopsocus lanceolatus* in Ansorge (2003a), Upper Lias, Grimmen, Mecklenburg-Vorpommern, Germany.

F. Saurodectidae Rasnitsyn and Zherikhin, 2000 K1(Valanginian)
Originally interpreted as a phthirapteran, Wappler et al. (2004) and Dalgleish et al. (2006) remove it from that order. Grimaldi and Engel (2005) consider affinities with Phthiraptera to be plausible so it is retained here within Paraneoptera.

First and Last: *Saurodectes vrsanskyi* in Dalgleish et al. (2006), Zaza Formation, Baissa, Buryatia, Russian Federation.

F. Surijokopsocidae P2(Wordian)

First and Last: *Surijokopsocus radtshenkoi* in Rohdendorf (1991), IlŠinskoe Formation, Suriyokova (Suriekova), Kemerovo Region, Russian Federation.

F. Zygopsocidae P3(Changhsingian)

First and Last: *Zygopsocus permianus* in Jell (2004), Belmont insect beds, Newcastle Coal Measures, Belmont/Warner's Bay, New South Wales, Australia.

## Holometabola (= Endopterygota)

**O. Coleoptera** Linnaeus, 1758 (Scarabaeida)
Carboniferous(Moscovian)-Quaternary(Holocene)

F. Acanthocnemidae K2(Cenomanian)-Holocene

First: *Acanthocnemoides sukatshevae* in Carpenter (1992b), Begichev Formation retinite, Khatanga River basin, Taimyr, Russian Federation.

F. Ademosynidae T1(Induan)-K1(Barremian)

First: Mentioned in Shcherbakov (2008a), Babiy Kamen', Maltseva/Sosnovaya Fomation, Kuznetsk Basin, Siberian Federal District, Russian Federation.

Last: e.g. *Atalosyne sinuolata* in Tan et al. (2007), Lushangfen Formation, Jingxi Basin, Beijing Municipality, China.

F. Aderidae (Circaeidae, Euglenidae) K1(Barremian)-Holocene

First: Figured in Grimaldi and Engel (2005), Lebanese amber, unknown horizon, unknown locality, Lebanon.

F. Adiphlebidae C2(Moscovian)

First and Last: *Adiphlebia lacoana* in Béthoux (2009), Carbondale Formation, Mazon Creek, Illinois, United States.

F. Agyrtidae Thomson, 1859 K1(Hauterivian)-Holocene
Formerly treated as a subfamily within Silphidae.

First: *Ponomarenkia parva* in Perkovsky (2001), Turga Formation, Turga River, near Borzai, Transbaikalia, Russian Federation.

F. Anthicidae K1(Barremian)-Holocene

First: *Camelomorpha longicervix* Kirejtshuk, Azar & Telnov *in* Kirejtshuk and Azar, 2008, Hammana/Mdeyrij amber, Caza Baabda, Mouhafazet Jabal Loubnan, Lebanon.

F. Anthribidae (Urodontidae) K1(Barremian)-Holocene

First: *Cretochoragus pygmaeus* Soriano et al., 2006a, Montsec lithographic limestones, Montsec Range, Lleida Province, Spain.

F. Artematopodidae (Artematopidae) Eoc.(Priabonian)-Holocene

First: e.g. *Electribius balticus* in Kubisz (2000), Baltic amber.

F. Asiocoleidae P2(Roadian)-P3(Changhsingian)

First: *Asiocoleus novojilovi* in Carpenter (1992b), Kuznetsk Formation (Mitino Horizon), Kaltan, Kemerovo Region, Russian Federation.

Last: Mentioned in Beattie (2007), Belmont insect beds, Newcastle Coal Measures, Belmont/Warner's Bay, New South Wales, Australia.

F. Attelabidae (Rhynchitidae) K1(Valanginian)-Holocene

First: Mentioned in Zherikhin and Gratshev (2004), Zaza Formation, Baissa, Buryatia, Russian Federation.

F. Belidae (Oxycorynidae) K1(Barremian)-Holocene

First: e.g. *Distenorrhinoides simulator* in Legalov (2009b), Montsec lithographic limestones, Montsec Range, Lleida Province, Spain.

F. Berendtimiridae Winkler, 1987 Eoc.(Priabonian)

First and Last: *Berendtimirus progenitor* Winkler, 1987, Baltic amber.

F. Biphyllidae (Biphyliidae) K1(Barremian)-Holocene

First: Mentioned in Kirejtshuk and Azar (2008), Lebanese amber, unknown horizon, unknown locality, Lebanon. (This identification is doubtful.)

F. Boganiidae K1(Barremian)-Holocene

First: Mentioned in Kirejtshuk and Azar (2008), Lebanese amber, unknown horizon, unknown locality, Lebanon. (Identification of these specimens is tentative.)

F. Bostrichidae (Bostrychidae, Lyctidae) K1(Albian)-Holocene

First: Mentioned in Delclòs et al. (2007), Álava amber, Escucha Formation, Basco-Cantabrian Basin, Álava Province, Spain.

F. Bothrideridae Eoc.(Priabonian)-Holocene

First: e.g. *Ascetoderes* sp. in Kupryjanowicz (2001), Baltic amber.

F. Brachyceridae (Erirhinidae) Eoc.(Priabonian)-Holocene

First: e.g. *Oryctorhinus tenuirostris* in Zherikhin (2000), Florissant Formation, Florissant, Colorado, United States.

F. Brachypsectridae Mio.(Burdigalian)-Holocene

First: *Brachypsectra moronei* Costa et al., 2006, Dominican amber, Cordillera Septentrional, near Santiago, Dominican Republic.

F. Brentidae (Apionidae, Brenthidae, Ithyceridae, Nanophyidae) K1(Valanginian)-Holocene
Legalov (2009c) treats Ithyceridae as a separate family and puts together subfamilies which are treated differently by Bouchard et al. (2011).

First: Mentioned in Zherikhin and Gratshev (2004), Zaza Formation, Baissa, Buryatia, Russian Federation.

F. Buprestidae T3(Carnian)-Holocene

First: e.g. *Mesostigmodera typica* in Jell (2004), Blackstone Formation, Ipswich Basin, Queensland, Australia.

F. Byrrhidae T1(Induan)-Holocene

First: Mentioned in Shcherbakov (2008a), Babiy Kamen', Maltseva/Sosnovaya Fomation, Kuznetsk Basin, Siberian Federal District, Russian Federation.

F. Byturidae K1(Berriasian)-Holocene

First: Figured in Jarzembowski (1992), Durlston Formation (Stair Hole Member), Durlston Bay, Dorset, United Kingdom. (This record is tentative.)

F. Callirhipidae (Callirhypidae) K2(Santonian)-Holocene

First: Mentioned in Ponomarenko (2002a), unknown horizon, unknown locality. (Ponomarenko 2002a does not actually state which Upper Cretaceous amber this family is known from and this record is absent from his on-line catalogue [`http://www.zin.ru/animalia/coleoptera/eng/paleosys.htm`], so the age for Siberian amber is tentatively used here.)

F. Cantharidae K1(Aptian)-Holocene

First: Figured in Jell (2004), Koonwarra Fossil Bed (Korumburra Group), South Gippsland, Victoria, Australia.

F. Carabidae (Carabaeidae, Cicindelidae, Nebriidae, Paussidae) T3(Carnian)-Holocene

First: Figured in Grimaldi and Engel (2005), Cow Branch Formation, Solite quarry, Virginia, United States.

F. Caridae K1(Valanginian)-Holocene

First: e.g. *Baissorhynchus tarsalis* in Legalov (2009c), Zaza Formation, Baissa, Buryatia, Russian Federation. (Ponomarenko's online catalogue [`http://www.zin.ru/animalia/coleoptera/eng/paleosys.htm`] lists specimens from Semen/Semyon [Argun' Formation] as Upper Jurassic but they are actually of uncertain Lower Cretaceous age.)

F. Catiniidae (Catinidae) T3(Carnian)-K1(Albian)

First: e.g. *Catinoides rotundatus* in Tan and Ren (2007), Madygen Formation, Madygen/Dzhailoucho, south Fergana Valley, Kyrgyzstan.

Last: e.g. *Catinus ovatus* in Tan and Ren (2007), Dalazi Formation, Zhixin Basin, Liaoning Province, China.

F. Cerambycidae (Cerambicidae, Pseudonepidae) K1(Albian)-Holocene
Grimaldi and Engel (2005) (p.393) consider *Cerambyomima* (which they misspell) from the Karabastau Formation as the oldest member of this family, although it is traditionally listed in Chrysomelidae, such as by Zhang (2005) and Ponomarenko's online catalogue (`http://www.zin.ru/animalia/coleoptera/eng/paleosys.htm`). *Willcoxia* from the Upper Triassic of Australia (in Jell, 2004) probably belongs to the Tricoleidae (see Ponomarenko, 2008).

First: Mentioned in Rasnitsyn and Ross (2000), Burmese amber (Burmite), Hukawng Valley, Kachin State, Myanmar.

F. Cerophytidae J2(Callovian)-Holocene

First: Mentioned in Chang et al. (2009), Jiulongshan Formation, near Daohugou, Ningcheng county, Inner Mongolia, China.

F. Cerylonidae Eoc.(Priabonian)-Holocene

First: e.g. *Philothermopsis*? sp. in Kupryjanowicz (2001), Baltic amber.

F. Chelonariidae K1(Barremian)-Holocene

First: Mentioned in Kirejtshuk et al. (2009a), Lebanese amber, unknown horizon, unknown locality, Lebanon.

F. Chrysomelidae (Bruchidae) J2(Callovian)-Holocene

First: *Tarsomegamerus mesozoicus* Zhang, 2005, Jiulongshan Formation, near Daohugou, Ningcheng county, Inner Mongolia, China.

F. Ciidae (Cisidae, Cisiidae, Cissidae) K1(Albian)-Holocene

First: Figured in Grimaldi et al. (2002), Burmese amber (Burmite), Hukawng Valley, Kachin State, Myanmar.

F. Cistelidae Mio.(Aquitanian)-Holocene

First: Mentioned in Engel (2004a), Mexican amber, Simojovel, Chiapas, Mexico.

F. Clambidae K1(Barremian)-Holocene

First: *Eoclambus rugidorsum* Kirejtshuk and Azar, 2008, Hammana/Mdeyrij amber, Caza Baabda, Mouhafazet Jabal Loubnan, Lebanon.

F. Cleridae K1(Barremian)-Holocene

First: Figured in Kaddumi (2005), Jordanian amber, Kurnub Sandstone Formation, Zarqua River, Jordan.

F. Coccinellidae Eoc.(Ypresian)-Holocene
Ponomarenko (2002a) mentions this family occurring in Upper Cretaceous amber, but according to his website (http://www.zin.ru/animalia/coleoptera/eng/paleosys.htm) this record is doubtful.

First: e.g. Mentioned in Kirejtshuk and Nel (2008), Oise amber, Le Quesnoy, Houdancourt, Oise, Picardie, France.

F. Colonidae Pleist.(Gelasian)-Holocene

First: *Colon* sp. in Böcher (1995), Kap København Formation, Peary Land, Northeast Greenland National Park, Greenland.

F. Colymbotethidae Ponomarenko, 1994(Colymbothetidae) T3(Norian)

First and Last: *Colymbotethis antecessor* in Sinitshenkova (2002c), Tologoy Formation, Ak-Kolka River, Kenderlyk, Zaisan District, Kazakhstan.

F. Coptoclavidae T3(Carnian)-K1(Aptian)

First: e.g. *Agrascapha curta* Lin, 1992, Huangshanjie Formation, Kerjie, Toksun county, Xinjiang Uyghur Autonomous Region, China.

Last: Mentioned in Wang et al. (2009a), Yixian Formation, Liaoning Province, China. (According to Wolf-Schwenninger and Schawaller 2007 and Bechly 2007b, *Conan barbarica* Martins-Neto is a dragonfly nymph.)

F. Corylophidae (Orthoperidae) K2(Campanian)-Holocene

First: Mentioned in McKellar et al. (2008), Canadian amber, Grassy Lake, Alberta, Canada.

F. Cossonidae Mio.(Aquitanian)-Holocene

First: Mentioned in Engel (2004a), Mexican amber, Simojovel, Chiapas, Mexico.

F. Cryptophagidae K1(Barremian)-Holocene

First: Mentioned in Kirejtshuk and Azar (2008), Lebanese amber, unknown horizon, unknown locality, Lebanon. (This identification is doubtful.)

F. Cucujidae K1(Barremian)-Holocene

First: Mentioned in Poinar and Poinar (2008), Lebanese amber, unknown horizon, unknown locality, Lebanon.

F. Cupedidae (Cupesidae) T2(Anisian)-Holocene

First: Mentioned in Shcherbakov (2008a), Grès à Voltzia, Bas-Rhin/Moselle, Northern Vosges Mountains, France.

F. Curculionidae (Platypodidae, Scolytidae) T2(Anisian)-Holocene
Gratshev and Zherikhin (2003) place *Paleoscolytus sussexensis* from the Wadhurst Clay as Coleoptera *incertae sedis*.

First: e.g. *Mesorhynchophora dunstani* in Jell (2004), Ashfield Formation, St. Peters, Sydney, New South Wales, Australia.

F. Dascillidae T3(Carnian)-Holocene

First: e.g. *Leioodes plana* in Jell (2004), Blackstone Formation, Ipswich Basin, Queensland, Australia.

F. Dermestidae J3(Tithonian)-Holocene
The oldest known body-fossils of Dermestidae are found in Lebanese amber (Kirejtshuk et al., 2009b). The Triassic taxa in Jell (2004) are considered to be family uncertain (Hava et al., 2006).

First: ichnofossils in Britt et al. (2008), Morrison Formation (upper), Carbon County, Wyoming, United States.

F. Derodontidae Pleist.(Gelasian)-Holocene

First: *Laricobius* cf. *caucasicus* in Böcher (1995), Kap København Formation, Peary Land, Northeast Greenland National Park, Greenland.

F. Discolomatidae (Discolomidae) Mio.(Aquitanian)-Holocene
Engel (2004a) notes that this family was listed in Mexican amber by Poinar (1992) as a hemipteran. Solórzano Kraemer (2007) also lists this family under Hemiptera.

First: Mentioned in Solórzano Kraemer (2007), Mexican amber, Simojovel, Chiapas, Mexico.

F. Dryophthoridae Eoc.(Priabonian)-Holocene

First: e.g. *Stenommatomorphus hexarthrus* Nazarenko *in* Nazarenko and Perkovsky, 2009, Rovno amber, Klesov/Dubrovitsa, Rivne Oblast, Ukraine.

F. Dryopidae K1(Aptian)-Holocene

First: Mentioned in Wolf-Schwenninger and Schawaller (2007), Crato Formation, Araripe Basin, Ceará, Brazil.

F. Dytiscidae (Dytisicdae) J3(Oxfordian)-Holocene
Ponomarenko's website (http://www.zin.ru/animalia/coleoptera/eng/paleosys.htm) lists the Lower Jurassic *Angaragabus* (Ust-Baley, Toarcian) in Liadytidae, however Grimaldi and Engel (2005) consider it as a putative dytiscid.

First: *Palaeodytes gutta* in Ross and Jarzembowski (1993), Karabastau Formation, Karatau Range, Tien Shan mountains, Kazakhstan.

F. Elateridae T2(Ladinian)-Holocene

First: e.g.? *Gemelina triangularis* Martins-Neto & Gallego *in* Martins-Neto et al., 2006, Los Rastros Formation, Bermejo Basin, La Rioja Province, Argentina.

F. Elmidae Eoc.(Lutetian)-Holocene

First: *Potamophilites angustifrons* , Geiseltal, near Halle, Saxony-Anhalt, Germany.

F. Elodophthalmidae Kirejtshuk and Azar, 2008 K1(Barremian)

e.g. *Elodophthalmus harmonicus* Kirejtshuk and Azar, 2008, Hammana/Mdeyrij amber, Caza Baabda, Mouhafazet Jabal Loubnan, Lebanon.

F. Endomychidae K1(Barremian)-Holocene
*Palaeoendomychus gymnus* (Barremian, Laiyang Formation, China) is now placed in Trogossitidae (Schmied et al., 2009).

First: Mentioned in Poinar and Poinar (2008), Lebanese amber, unknown horizon, unknown locality, Lebanon.

F. Erotylidae (Languriidae) K1(Barremian)-Holocene

First: Mentioned in Kirejtshuk and Azar (2008), Lebanese amber, unknown horizon, unknown locality, Lebanon.

F. Eucinetidae J3(Oxfordian)-Holocene

First: *Mesocinetus* sp. in http://www.zin.ru/animalia/coleoptera/eng/paleosys.htm, Karabastau Formation, Karatau Range, Tien Shan mountains, Kazakhstan. (Referred to by Ponomarenko 2002a as occurring in the Late Jurassic, without details.)

F. Eucnemidae K1(Barremian)-Holocene

First: Mentioned in Kirejtshuk et al. (2009a), Lebanese amber, unknown horizon, unknown locality, Lebanon.

F. Geotrupidae (Bolboceratidae) J3(Tithonian)-Holocene

First: *Geotrupoides lithographicus* in Krell (2007), Solenhofen Lithographic Limestone, Solenhofen/Eichstadt, Bavaria, Germany. (This record is doubtful.)

F. Glaphyridae K1(Valanginian)-Holocene

First: e.g. *Cretoglaphyrus rohdendorfi* in Krell (2007), Zaza Formation, Baissa, Buryatia, Russian Federation.

F. Glaresidae J1(Hettangian)-Holocene

First: *Aphodiites protogaeus* in Krell (2007), Schambelen Member, Staffelegg Formation, Brugg, Aargau, Switzerland. (The family identity is doubtful.)

F. Gyrinidae J1(Pliensbachian)-Holocene

First: e.g. *Mesogyrus sibiricus* in Prokop et al. (2004), Osinovskiy Formation, Chernyi Etap, Kemerovo Region, Russian Federation.

F. Haliplidae K1(Aptian)-Holocene

First: e.g. *Cretihaliplus chifengensis* in Prokop et al. (2004), Jiufotang Formation, Beishan, Yixian County, Liaoning Province, China.

F. Haplochelidae Kirejtshuk and Poinar, 2006 K1(Albian)

First and Last: *Haplochelus georissoides* Kirejtshuk and Poinar, 2006, Burmese amber (Burmite), Hukawng Valley, Kachin State, Myanmar.

F. Helotidae Mio.(Langhian)-Holocene
Not to be confused with Helodidae (see Scirtidae).

First: e.g. *Helota zhangi* Wegrzynowicz, 2007, Shanwang Formation, Linqu County, Shandong Province, China.

F. Heteroceridae K1(Hauterivian)-Holocene

First: *Heterocerites kobdoensis* Ponomarenko, 1986, Gurvan-Eren Formation, Myangad, Khovd Aimag, Mongolia.

F. Histeridae K1(Albian)-Holocene

First: *Pantostictus burmanicus* Poinar and Brown, 2009, Burmese amber (Burmite), Hukawng Valley, Kachin State, Myanmar.

F. Hybosoridae J2(Callovian)-Holocene

First: *Jurahybosorus mongolicus* in Krell (2007), Bayan-Teg, Bayan-Teg Coal Quarry, Övörkhangai (Ubur-Khangaisk) Aimag, Mongolia.

F. Hydraenidae J2(Aalenian)-Holocene

First: *Ochtebiites altus* in Ponomarenko (2003a), Ichetuy Formation, Novospasskoye, Mukhorshibirsky District, Buryatia, Russian Federation.

F. Hydrophilidae (Epimetopidae, Georissidae, Georyssidae, Helophoridae, Hydrochidae, Hydrophyllidae, Spercheidae) T1(Induan)-Holocene

First: Mentioned in Shcherbakov (2008a), Babiy Kamen', Maltseva/Sosnovaya Fomation, Kuznetsk Basin, Siberian Federal District, Russian Federation.

F. Hygrobiidae Olig.(Chattian)-Holocene

First: *Hygrobia cretzschmari* , Rott paper shales, Bonn, North Rhine-Westphalia, Germany.

F. Jurodidae (Sikhotealiniidae) J2(Aalenian)-Holocene

First: *Jurodes ignoramus* in Hörnschemeyer (2005), Ichetuy Formation, Novospasskoye, Mukhorshibirsky District, Buryatia, Russian Federation.

F. Kateretidae (Brachypteridae) K1(Barremian)-Holocene

First: *Lebanoretes andelmani* Kirejtshuk and Azar, 2008, Hammana/Mdeyrij amber, Caza Baabda, Mouhafazet Jabal Loubnan, Lebanon.

F. Labradorocoleidae K2(Cenomanian)
Ponomarenko (2000b) notes that without investigating the body of the specimen for cryptosterny, it is not possible to say for certain if this family belongs to Coleoptera or Blattodea.

First and Last: *Labradorocoleus carpenteri* in Carpenter (1992b), Redmond Formation, Knob Lake District, Labrador, Canada.

F. Lampyridae Eoc.(Priabonian)-Holocene

First: e.g. "*Lucidota*" *prima* in Meyer (2003), Florissant Formation, Florissant, Colorado, United States.

F. Latridiidae (Lathridiidae) K1(Barremian)-Holocene

First: e.g. *Tetrameropsis mesozoica* Kirejtshuk and Azar, 2008, Hammana/Mdeyrij amber, Caza Baabda, Mouhafazet Jabal Loubnan, Lebanon.

F. Leiodidae (Catopidae, Cholevidae, Leiodesidae, Liodidae) J2(Aalenian)-Holocene

First: e.g. *Mesecanus communis* in Perkovsky (2001), Ichetuy Formation, Novospasskoye, Mukhorshibirsky District, Buryatia, Russian Federation.

F. Liadytidae (Lyadytidae) T3(Carnian)-J3(Tithonian)

First: Mentioned in Shcherbakov (2008a), Cow Branch Formation, Solite quarry, Virginia, United States. (Shcherbakov 2008a lists this as a possible occurrence.)

Last: e.g. *Liadytes longus* in Ponomarenko (2002a), Glushkovo Formation, Unda, Transbaikalia, Russian Federation.

F. Limnichidae Eoc.(Priabonian)-Holocene

First: e.g. *Palaeoersachus bicarinatus* Pütz et al., 2004, Baltic amber.

F. Limulodidae Mio.(Aquitanian)-Holocene

First: Mentioned in Engel (2004a), Mexican amber, Simojovel, Chiapas, Mexico.

F. Lucanidae (Paralucanidae) J3(Tithonian)-Holocene

First: *Paralucanus mesozoicus* in Krell (2007), Shar-Teg Formation, Shar-Teg Ula, Gobi-Altai Aimag, Mongolia.

F. Lycidae Eoc.(Priabonian)-Holocene

First: e.g. *Miocaenia pectinicornis* in Meyer (2003), Florissant Formation, Florissant, Colorado, United States.

F. Lymexylidae (Lymexilidae, Lymexylonidae) K1(Barremian)-Holocene

First: Mentioned in Kirejtshuk et al. (2009a), Lebanese amber, unknown horizon, unknown locality, Lebanon.

F. Magnocoleidae Hong, 1998b K1(Barremian)

First and Last: *Magnocoleus huangjiapuensis* Hong, 1998b, Qingshila Formation, Huangjiapu, Hebei Province, China.

F. Melandryidae (Serropalpidae) K1(Barremian)-Holocene

First: Mentioned in Kirejtshuk et al. (2009a), Lebanese amber, unknown horizon, unknown locality, Lebanon.

F. Meloidae Pal.(Thanetian)-Holocene

First: e.g. *Zonabris immaculatus* in Engel (2005a), spongo-diatomaceous maar, Menat, Puy-de-Dôme, Auvergne, France.

F. Melyridae (Dasytidae, Malachiidae) K1(Barremian)-Holocene

First: Mentioned in Kirejtshuk et al. (2009a), Lebanese amber, unknown horizon, unknown locality, Lebanon.

F. Micromalthidae J3(Oxfordian)-Holocene

First: Mentioned in Kirejtshuk and Azar (2008), Karabastau Formation, Karatau Range, Tien Shan mountains, Kazakhstan.

F. Monotomidae (Rhizophagidae) K1(Barremian)-Holocene

First: *Rhizophtoma elateroides* Kirejtshuk & Azar *in* Kirejtshuk et al., 2009a, Lebanese amber, unknown horizon, unknown locality, Lebanon.

F. Mordellidae (Liaoximordellidae, Praemordellidae) J3(Oxfordian)-Holocene

First: *Praemordella martynovi* in Liu et al. (2008a), Karabastau Formation, Karatau Range, Tien Shan mountains, Kazakhstan.

F. Mycetophagidae K1(Barremian)-Holocene

First: Figured in Poinar and Milki (2001), Lebanese amber, unknown horizon, unknown locality, Lebanon.

F. Mycteridae Eoc.(Ypresian)-Holocene

First: *Bertinotus gallicus* Kirejtshuk and Nel, 2009, Oise amber, Le Quesnoy, Houdancourt, Oise, Picardie, France.

F. Nemonychidae (Eccoptarthridae, Eobelidae) J3(Oxfordian)-Holocene
Soriano (2009) considers Eobelinae in Belidae but Ponomarenko's website, Legalov (2009a) and Bouchard et al. (2011) do not.

First: e.g. *Megabrenthorrhinus grandis* in Legalov (2009a), Karabastau Formation, Karatau Range, Tien Shan mountains, Kazakhstan.

F. Nitidulidae (Cybocephalidae) K1(Valanginian)-Holocene

First: e.g. *Crepuraea archaica* in Kirejtshuk (2008), Zaza Formation, Baissa, Buryatia, Russian Federation.

F. Nosodendridae Eoc.(Ypresian)-Holocene

First: *Nosodendron tritavum* in Handlirsch (1908), Green River Formation, Unitas area, Wyoming, United States.

F. Noteridae (Phreatodytidae) Pal.(Thanetian)-Holocene

First: Mentioned in Sinitshenkova (2002c), Paskapoo Formation, eastern foothills, Rocky Mountains, Alberta, Canada.

F. Oborocoleidae P1(Sakmarian)

e.g. *Oborocoleus rohdendorfi* in Zajíc and Štamberg (2004), Obora locality, Bačov Beds, Letovice Formation, Moravia, Czech Republic.

F. Obrieniidae Zherikhin and Gratshev, 1994 T3(Carnian)-J3(Oxfordian)

First: e.g. *Obrienia kuscheli* in Ponomarenko (2002a), Madygen Formation, Madygen/Dzhailoucho, south Fergana Valley, Kyrgyzstan.

Last: *Kararhynchus occiduus* Zherikhin and Gratshev, 1994, Karabastau Formation, Karatau Range, Tien Shan mountains, Kazakhstan.

F. Ochodaeidae K1(Barremian)-Holocene

First: e.g. *Cretochodaeus mongolicus* in Krell (2007), Khurilt Formation, Bon-Tsagaan Group, Bayankhongor Aimag, Mongolia.

F. Oedemeridae K1(Albian)-Holocene

First: Mentioned in Grimaldi and Engel (2005), Burmese amber (Burmite), Hukawng Valley, Kachin State, Myanmar.

F. Ommatidae (Brochocoleidae, Tetraphaleridae) T2(Ladinian)-Holocene

First: *Notocupes* sp. in Krzemiński and Lombardo (2001), Upper Meride Limestone, Val Mara, Canton Ticino, Switzerland.

F. Parahygrobiidae J3(Oxfordian)

First and Last: *Parahygrobia natans* in Grimaldi and Engel (2005), Uda Formation, Uda River, Buryatia, Russian Federation.

F. Parandrexidae Kirejtshuk, 1994 J2(Callovian)-K1(Barremian)

First: *Parandrexis beipiaoensis* in Zhang (2005), Haifanggou Formation, Beipiao, Liaoning Province, China.

Last: *Martynopsis laticollis* Soriano et al., 2006b, Calizas de la Huérguina Formation, Las Hoyas, Cuénca Province, Spain.

F. Passalidae Olig.(Chattian)-Holocene

First: *Passalus indormitus* in Krell (2007), Post, John Day series, Oregon, United States.

F. Passandridae Eoc.(Priabonian)-Holocene

First: e.g. *Passandra* sp. in `http://www.zin.ru/animalia/coleoptera/eng/paleosys.htm`, Baltic amber.

F. Permocupedidae (Kaltanocoleidae) P1(Artinskian)-P3(Changhsingian)

First: e.g. *Kaltanicupes ponomarenkoi* in Geertsema et al. (2002), Irati Formation, Paraná Basin, São Paulo, Brazil.

Last: Mentioned in Beattie (2007), Belmont insect beds, Newcastle Coal Measures, Belmont/Warner's Bay, New South Wales, Australia.

F. Permosynidae P2(Roadian)-T3(Carnian)

First: e.g. *Permosyne elongata* Ponomarenko *in* Ponomarenko and Mostovski, 2005, Volksrust Formation, Ecca Group, KwaZulu-Natal, Karoo Basin, South Africa.

Last: e.g. *Pseudorhynchophora olliffi* in Ponomarenko (2008), Blackstone Formation, Ipswich Basin, Queensland, Australia.

F. Phalacridae Eoc.(Ypresian)-Holocene

First: Mentioned in Kirejtshuk and Nel (2008), Oise amber, Le Quesnoy, Houdancourt, Oise, Picardie, France.

F. Phloeostichidae K1(Barremian)-Holocene

First: Mentioned in Kirejtshuk and Azar (2008), Lebanese amber, unknown horizon, unknown locality, Lebanon. (Identification of these specimens is tentative.)

F. Pleocomidae K1(Valanginian)-Holocene

First: e.g. *Proteroscarabaeus magnus* in Krell (2007), Zaza Formation, Baissa, Buryatia, Russian Federation. (This record is doubtful.)

F. Praelateriidae (Praelateridae) J1(Hettangian)-J1(Sinemurian)

First: *Megacentrus tristis* in `http://www.zin.ru/animalia/coleoptera/eng/paleosys.htm`, Schambelen Member, Staffelegg Formation, Brugg, Aargau, Switzerland.

Last: e.g. *Praelaterium problematicum* in Carpenter (1992b), Dzhil Formation, Sogyuty, Issyk-Kul, Kyrgyzstan.

F. Prionoceridae Eoc.(Ypresian)-Holocene

First: *Prionocerites tattriei* Lawrence et al., 2008, Hat Creek amber, Kamploops Group, British Columbia, Canada.

F. Propalticidae Eoc.(Priabonian)-Holocene

First: *Propalticus* sp. in http://www.zin.ru/animalia/coleoptera/eng/paleosys.htm, Baltic amber. (This appears to be an unpublished record. Next oldest is in Pliocene Kenyan copal.)

F. Prostomidae K1(Albian)-Holocene

First: *Vetuprostomis consimilis* Engel and Grimaldi, 2008b, Burmese amber (Burmite), Hukawng Valley, Kachin State, Myanmar.

F. Psephenidae K1(Barremian)-Holocene

First: Mentioned in Soriano et al. (2007), Montsec lithographic limestones, Montsec Range, Lleida Province, Spain.

F. Ptiliidae (Ptilidae) K1(Barremian)-Holocene

First: Mentioned in Kirejtshuk et al. (2009a), Lebanese amber, unknown horizon, unknown locality, Lebanon.

F. Ptilodactylidae K1(Barremian)-Holocene

First: e.g. Figured in Soriano et al. (2007), Montsec lithographic limestones, Montsec Range, Lleida Province, Spain.

F. Ptinidae (Anobiidae) K1(Albian)-Holocene
Zherikhin (2002c) mentions that undescribed specimens of this family (as Anobiidae) are known from the "Early Cretaceous of Transbaikalia" (p.354), which could be a number of different deposits.

First: Mentioned in Alonso et al. (2000), Álava amber, Escucha Formation, Basco-Cantabrian Basin, Álava Province, Spain. (Not mentioned in Delclòs et al. 2007.)

F. Pyrochroidae (Pedilidae, Pirochoidae, Pyreochroidae) K1(Aptian)-Holocene

First: *Cretaceimelittomoides cearensis* (nomen nudum) in Wolf-Schwenninger and Schawaller (2007), Crato Formation, Araripe Basin, Ceará, Brazil. (This record is doubtful.)

F. Pythidae Eoc.(Priabonian)-Holocene

First: e.g. *Pythoceropsis singularis* in Carpenter (1992b), Florissant Formation, Florissant, Colorado, United States. (Family also occurs in Baltic amber.)

F. Rhombocoleidae P2(Roadian)-K1(Aptian)

First: e.g. *Rhombocoleites danutae* Ponomarenko and Mostovski, 2005, Volksrust Formation, Ecca Group, KwaZulu-Natal, Karoo Basin, South Africa.

Last: *Sinorhombocoleus papposus* in Tan and Ren (2009), Jianshangou beds, Yixian Formation, Liaoning Province, China.

F. Rhysodidae Eoc.(Priabonian)-Holocene

First: Mentioned in Poinar (1992), Baltic amber.

F. Ripiphoridae (Rhipiphoridae) K1(Albian)-Holocene

First: e.g. *Paleoripiphorus deploegi* Perrichot et al., 2004, Archingeay amber, Archingeay-Les Nouillers, Charente-Maritime, France.

F. Salpingidae (Inopeplidae) K1(Barremian)-Holocene

First: Mentioned in Poinar and Milki (2001), Lebanese amber, unknown horizon, unknown locality, Lebanon.

F. Scarabaeidae (Aphodiidae, Cetoniidae, Lithoscarabaeidae, Melolonthidae, Melonthidae, Rutelidae) J3(Oxfordian)-Holocene

First: e.g. *Juraclopus rohdendorfi* in Krell (2007), Karabastau Formation, Karatau Range, Tien Shan mountains, Kazakhstan.

F. Schizocoleidae P2(Roadian)-J2(Bathonian)

First: e.g. *Palademosyne natalensis* Ponomarenko *in* Ponomarenko and Mostovski, 2005, Volksrust Formation, Ecca Group, KwaZulu-Natal, Karoo Basin, South Africa.

Last: *Mimemala punctatum* in Carpenter (1992b), Stonesfield Slate, Taynton Limestone Formation, Oxfordshire, United Kingdom. (Listed on PonomarenkoŠs website [http://www.zin.ru/animalia/coleoptera/eng/paleosys.htm], under the junior homonym *Mimema*.)

F. Schizophoridae P2(Capitanian)-K1(Barremian)

First: *Dikerocoleus divisus* in Tan et al. (2007), Yinping Formation, Houdong, SW Chaohu City, Anhui Province, China.

Last: Figured in Soriano et al. (2007), Calizas de la Huérguina Formation, Las Hoyas, Cuénca Province, Spain.

F. Schizopodidae (Electrapatidae) Eoc.(Priabonian)-Holocene

First: *Electrapate martynovi* in Bellamy (1995), Baltic amber.

F. Scirtidae (Helodidae, Sinodryopitidae) J3(Oxfordian)-Holocene

First: Mentioned in Kirejtshuk and Azar (2008), Karabastau Formation, Karatau Range, Tien Shan mountains, Kazakhstan.

F. Scraptiidae (Scaraptiidae, Scraptidae) J3(Oxfordian)-Holocene

First: Mentioned in Ponomarenko (2002a), Karabastau Formation, Karatau Range, Tien Shan mountains, Kazakhstan.

F. Scydmaenidae K1(Barremian)-Holocene

First: Mentioned in Kirejtshuk et al. (2009a), Lebanese amber, unknown horizon, unknown locality, Lebanon.

F. Silphidae Eoc.(Lutetian)-Holocene
Ponomarenko's website (`http://www.zin.ru/animalia/coleoptera/eng/paleosys.htm`) places *Mercata festira* (oldest in FR2) in Elateridae, although he misspells it.

First: e.g. *Eosilphites decoratus* in Carpenter (1992b), Geiseltal, near Halle, Saxony-Anhalt, Germany.

F. Silvanidae K1(Barremian)-Holocene

First: Mentioned in Kirejtshuk et al. (2009a), Lebanese amber, unknown horizon, unknown locality, Lebanon.

F. Sinisilvanidae Hong, 2002a(Sinislavanidae) Eoc.(Ypresian)

First and Last: *Sinisilvana fushunensis* Hong, 2002a, Fushun amber, Guchengzi, Liaoning Province, China.

F. Smicripidae Eoc.(Ypresian)-Holocene

First: *Smicrips europeus* Kirejtshuk and Nel, 2008, Oise amber, Le Quesnoy, Houdancourt, Oise, Picardie, France.

F. Sojanocoleidae P2(Roadian)

First and Last: *Sojanocoleus reticulatus* in Rohdendorf (1991), Iva-Gora limestones, Soyana River, Arkhangelsk Region, Ural Mountains, Russian Federation.

F. Sphaeriusidae (Microsporidae, Spaeriidae, Sphaeriidae) K1(Albian)-Holocene

First: *Burmasporum rossi* Kirejtshuk, 2009, Burmese amber (Burmite), Hukawng Valley, Kachin State, Myanmar.

F. Sphindidae (Aspidiphoridae) Eoc.(Priabonian)-Holocene

First: Mentioned in Weitschat and Wichard (2002), Baltic amber.

F. Staphylinidae (Micropeplidae, Pselaphidae, Scaphidiidae, Staphyllinidae) T3(Carnian)-Holocene

First: Figured in Grimaldi and Engel (2005), Cow Branch Formation, Solite quarry, Virginia, United States.

F. Synchroidae Eoc.(Priabonian)-Holocene

First: "*Synchroa*" *quiescent* in Meyer (2003), Florissant Formation, Florissant, Colorado, United States.

F. Taldycupedidae (Taldycupidae) P2(Roadian)-K1(Barremian)

First: e.g. *Taldycupes africanus* Ponomarenko *in* Ponomarenko and Mostovski, 2005, Volksrust Formation, Ecca Group, KwaZulu-Natal, Karoo Basin, South Africa.

Last: *Yiyangicupes huobashanense* in Tan and Ren (2009), Lengshuiwu Formation, Yiyang County, Jianxi Province, China.

F. Tenebrionidae (Alleculidae, Lagriidae) T2(Anisian)-Holocene

First: *Adelidium cordatum* in Jell (2004), Ashfield Formation, St. Peters, Sydney, New South Wales, Australia.

F. Throscidae (Trixagidae) K1(Barremian)-Holocene

First: Mentioned in Kirejtshuk et al. (2009a), Lebanese amber, unknown horizon, unknown locality, Lebanon.

F. Trachypachidae (Leptopodocoleidae, Trachypacheidae, Trachypachyidae) T1(Induan)-Holocene

First: Mentioned in Shcherbakov (2008a), Babiy Kamen', Maltseva/Sosnovaya Fomation, Kuznetsk Basin, Siberian Federal District, Russian Federation.

F. Triadocupedidae T3(Carnian)
Ponomarenko's website (`http://www.zin.ru/animalia/coleoptera/eng/paleosys.htm`) lists this as a subfamily of Cupedidae but Kirejtshuk and Azar (2008) and Bouchard et al. (2011) maintain it as a separate family.

e.g. *Moltenocupes townrowi* in Ponomarenko (2008), Molteno Formation, KwaZulu-Natal, Karoo Basin, South Africa.

F. Triaplidae T1(Induan)-J2(Callovian)

First: Mentioned in Shcherbakov (2008a), Babiy Kamen', Maltseva/Sosnovaya Fomation, Kuznetsk Basin, Siberian Federal District, Russian Federation.

Last: *Mesapus beipiaoensis* in Tan et al. (2007), Haifanggou Formation, Beipiao, Liaoning Province, China. (Ponomarenko's website [http://www.zin.ru/animalia/coleoptera/eng/paleosys.htm] lists this species under Hydrophilidae, although it is misspelt. Next youngest would be in Madygen.)

F. Tricoleidae P3(Changhsingian)-J2(Callovian)

First: e.g. Mentioned in Ponomarenko (2008), Belmont insect beds, Newcastle Coal Measures, Belmont/Warner's Bay, New South Wales, Australia.

Last: e.g. *Loculitricoleus flatus* Tan and Ren, 2009, Jiulongshan Formation, near Daohugou, Ningcheng county, Inner Mongolia, China.

F. Tritarsidae Hong, 2002a(Tritarsusidae) Eoc.(Ypresian)

First and Last: *Tritarsus latus* Hong, 2002a, Fushun amber, Guchengzi, Liaoning Province, China.

F. Trogidae K1(Valanginian)-Holocene

First: e.g. *Trox sibericus* in Krell (2007), Zaza Formation, Baissa, Buryatia, Russian Federation.

F. Trogossitidae (Lophocateridae, Ostomatidae, Ostomidae, Peltidae, Trogositidae) J1(Toarcian)-Holocene

First: *Thoracotes dubius* in Schmied et al. (2009), Upper Lias, Dobbertin, Mecklenburg-Vorpommern, Germany.

F. Tshekardocoleidae (Uralocoleidae) P1(Asselian)-J2(Aalenian)

First: e.g. Mentioned in Hörnschemeyer and Stapf (1999), Jeckenbach layers, Niedermoschel, Donnersbergkreis district, Rhineland-Palatinate, Germany.

Last: *Dictyocoleus jurassicus* in Tan and Ren (2009), Dashankou Group, Subei County, Jiuquan, Gansu Province, China.

F. Ulyanidae Zherikhin, 1993 K1(Valanginian)-K1(Albian)

First: Mentioned in Zherikhin and Gratshev (2004), Zaza Formation, Baissa, Buryatia, Russian Federation.

Last: *Ulyana nobilis* in Oberprieler et al. (2007), Emanra Formation, Khetana River, Khabarovsk Province, Russian Federation.

F. Zopheridae (Colydiidae) K1(Barremian)-Holocene

First: Figured in Poinar and Milki (2001), Lebanese amber, unknown horizon, unknown locality, Lebanon.

**O. Diptera** Linnaeus, 1758 (Muscida) Triassic(Anisian)-Quaternary(Holocene)

*Pareuthychaeta electrica* and *P. minuta* belong in Campichoetidae (Grimaldi, 2008), leaving Diastatidae without a fossil record. Much of the locality information from Evenhuis (1994) has been supplemented with information from http://www.palaeoentomolog.ru/Collections/diptera_e.html.

F. Acartophthalmidae Eoc.(Priabonian)-Holocene

First: e.g. *Acartophthalmites tertiaria* in von Tschirnhaus and Hoffeins (2009), Baltic amber.

F. Acroceridae (Archocyrtidae) J3(Oxfordian)-Holocene

First: e.g. *Juracyrtus kovalevi* in Hauser and Winterton (2007), Karabastau Formation, Karatau Range, Tien Shan mountains, Kazakhstan.

F. Agromyzidae Eoc.(Ypresian)-Holocene

First: *Foliofossor cranei* in Evenhuis (1994), Reading Formation, Cold Ash, Berkshire, United Kingdom. (This trace fossil record is tentative. Flies figured by Zlobin 2007 from Bembridge Marls, Isle of Wight.)

F. Anisopodidae (Anisopidae, Anisopodiae, Eopleciidae, Mycetobiidae, Olbiogastridae, Protolbiogastridae, Rhyphidae) J1(Sinemurian)-Holocene

First: *Mesorhyphus rhaeticus* in Evenhuis (1994), Dzhil Formation, Sogyuty, Issyk-Kul, Kyrgyzstan.

F. Ansorgiidae Krzemiński and Lukashevitch, 1993 J3(Oxfordian)

First and Last: *Ansorgius predictus* in Krzemiński and Evenhuis (2000), Karabastau Formation, Karatau Range, Tien Shan mountains, Kazakhstan.

F. Antefungivoridae (Antiquamediidae, Pleciomimidae, Sinemediidae) J1(Sinemurian)-K2(Santonian)

First: Mentioned in Ansorge (1996a), Dzhil Formation, Sogyuty, Issyk-Kul, Kyrgyzstan.

Last: Mentioned in Evenhuis (1994), Yantardakh amber, Kheta Formation, Taimyr, Krasnoyarsk Krai, Siberian Federal District, Russian Federation.

F. Anthomyiidae Eoc.(Priabonian)-Holocene

First: e.g. *Protanthomyia minuta* Michelsen, 2000, Baltic amber.

F. Anthomyzidae Eoc.(Priabonian)-Holocene

First: e.g. *Protanthomyza collarti* in von Tschirnhaus and Hoffeins (2009), Baltic amber.

F. Apioceridae K1(Valanginian)-Holocene

First: Mentioned in Grimaldi and Engel (2005), Zaza Formation, Baissa, Buryatia, Russian Federation.

F. Apsilocephalidae Nagatomi et al., 1991 K1(Albian)-Holocene
Gaimari and Mostovski (2000) do not consider this family to be a synonym of Rhagionempididae.

First: e.g. *Burmapsilocephala cockerelli* Gaimari and Mostovski, 2000, Burmese amber (Burmite), Hukawng Valley, Kachin State, Myanmar.

F. Apystomyiidae Nagatomi and Liu, 1994 J3(Oxfordian)-Holocene

First: Mentioned in Mostovski (2009), Karabastau Formation, Karatau Range, Tien Shan mountains, Kazakhstan.

F. Archisargidae (Mesophantasmatidae) J2(Callovian)-J3(Tithonian)

First: e.g. *Archirhagio zhangi* Zhang et al., 2009a, Jiulongshan Formation, near Daohugou, Ningcheng county, Inner Mongolia, China.

Last: *Mesosolva longivena* in Nagatomi and Yang (1998), Shar-Teg Formation, Shar-Teg Ula, Gobi-Altai Aimag, Mongolia.

F. Asilidae J3(Oxfordian)-Holocene
Dikow (2009) notes that putative specimens of this family from the Karabastau Formation may prove to be stem-Asiloidea and that the oldest definitive Asilidae is *Araripogon axelrodi* from the Crato Formation.

First: Mentioned in Mostovski (2009), Karabastau Formation, Karatau Range, Tien Shan mountains, Kazakhstan.

F. Asiochaoboridae Hong and Wang, 1990 K1(Barremian)

e.g. *Asiochaoborus tenuous* in Evenhuis (1994), Laiyang Formation, Laiyang County, Shandong Province, China.

F. Asteiidae Eoc.(Priabonian)-Holocene

First: e.g. *Succinasteia carpenteri* in von Tschirnhaus and Hoffeins (2009), Baltic amber.

F. Atelestidae K1(Berriasian)-Holocene

First: *Dianafranksia fisheri* in Grimaldi and Engel (2005), Lulworth Formation, Durlston Bay, Dorset, United Kingdom.

F. Athericidae K1(Berriasian)-Holocene

First: *Athericites sellwoodi* Mostovski et al., 2003a, Lulworth Formation, Durlston Bay, Dorset, United Kingdom.

F. Aulacigastridae Eoc.(Priabonian)-Holocene

First: e.g. *Protaulacigaster electrica* in von Tschirnhaus and Hoffeins (2009), Baltic amber.

F. Axymyiidae J2(Callovian)-Holocene

First: e.g. *Psocites fossilis* Zhang, 2004, Jiulongshan Formation, near Daohugou, Ningcheng county, Inner Mongolia, China.

F. Bibionidae (Hesperinidae, Penthetriidae, Pleciidae) J1(Toarcian)-Holocene

First: *Penthetria dubia* in Evenhuis (1994), Upper Lias, Dobbertin, Mecklenburg-Vorpommern, Germany.

F. Blephariceridae (Blepharoceridae) J2(Callovian)-Holocene

First: e.g. *Brianina longitibialis* Zhang and Lukashevitch, 2007, Jiulongshan Formation, near Daohugou, Ningcheng county, Inner Mongolia, China.

F. Boholdoyidae (Boholdoyiidae) J2(Aalenian)-K1(Hauterivian)

First: *Boholdoya alata* in Krzemiński and Evenhuis (2000), Ichetuy Formation, Novospasskoye, Mukhorshibirsky District, Buryatia, Russian Federation.

Last: *Boholdoya thoracica* in Evenhuis (1994), Turga Formation, Turga River, near Borzai, Transbaikalia, Russian Federation.

F. Bolitophilidae (Mangasidae) K1(Hauterivian)-Holocene

First: e.g. *Mangas exilis* in Blagoderov and Grimaldi (2004), Gurvan-Eren, Boro-Nuru, Khovd Aimag, Mongolia.

F. Bombyliidae (Phthiriidae, Systropodidae, Usiidae) K1(Hauterivian)-Holocene
*Palaeoplatypygus zaitzevi* is included in the Mythicomyiidae following Evenhuis (2002).

First: e.g.? Mentioned in Mostovski (2009), Gurvan-Eren, Boro-Nuru, Khovd Aimag, Mongolia.

F. Calliphoridae Eoc.(Lutetian)-Holocene
Rognes (1997) considers this family as not monophyletic, however, use of the name remains common in recent literature. Grimaldi and Cumming (1999), Zherikhin (2002c) and Grimaldi and Engel (2005) consider *Cretaphormia fowleri* from the Upper Cretaceous Edmonton Formation to be unplaced within Cyclorrhapha.

First: Mentioned in Evenhuis (1994), Geiseltal, near Halle, Saxony-Anhalt, Germany.

F. Camillidae Eoc.(Priabonian)-Holocene

First: e.g. *Protocamilla groehni* Grimaldi, 2008, Baltic amber.

F. Campichoetidae Eoc.(Priabonian)-Holocene

First: e.g. *Pareuthychaeta electrica* in von Tschirnhaus and Hoffeins (2009), Baltic amber.

F. Canthyloscelidae (Canthyloscelididae, Hyperoscelidae, Hyperoscelididae, Synneuridae) J2(Aalenian)-Holocene

First: *Prohyperoscelis jurassicus* in Evenhuis (1994), Itat Formation, Kubekovo, Krasnoyarsk Krai, Siberian Federal District, Russian Federation.

F. Carnidae Eoc.(Priabonian)-Holocene

First: e.g. *Meoneurites enigmatica* in von Tschirnhaus and Hoffeins (2009), Baltic amber.

F. Cecidomyiidae (Cecidomiidae, Lestremiidae) J3(Tithonian)-Holocene

First: *Catotricha mesozoica* in Jaschhof (2007), Glushkovo Formation, Daya, Transbaikalia, Russian Federation.

F. Ceratopogonidae (Leptoconopidae) K1(Hauterivian)-Holocene
*Simulidium priscum* from the Lulworth Formation belongs in Rhagionidae (Mostovski et al., 2003b).

First: e.g. *Minyohelea casca* Borkent, 1997, Austrian amber, Golling, Salzburg, Austria.

F. Chamaemyiidae Eoc.(Priabonian)-Holocene

First: *Procremifania electrica* in von Tschirnhaus and Hoffeins (2009), Baltic amber.

F. Chaoboridae (Chironomapteridae, Dixamimidae, Mesotendipedidae, Rhaetomyidae, Rhaetomyiidae) J1(Sinemurian)-Holocene

First: *Rhaetomyia necopinata* in Borkent (2008), Dzhil Formation, Sogyuty, Issyk-Kul, Kyrgyzstan.

F. Chimeromyiidae Grimaldi & Cumming *in* Grimaldi et al., 2009 K1(Hauterivian)-K1(Albian)

First: *Chimeromyia reducta* in Grimaldi et al. (2009), Jezzine amber, Jouar Ess-Souss, Mouhafazet Loubnan El-Janoubi, Lebanon.

Last: e.g. *Chimeromyia burmitica* Grimaldi & Cumming *in* Grimaldi et al., 2009, Burmese amber (Burmite), Hukawng Valley, Kachin State, Myanmar.

F. Chironomidae (Tendipedidae) T3(Rhaetian)-Holocene

First: *Aenne triassica* in Blagoderov et al. (2007), Cotham Member, Lilstock Formation, Penarth Group1, Strensham, Worcestershire, United Kingdom.

F. Chloropidae K1(Barremian)-Holocene

First: Mentioned in Solórzano Kraemer (2007), Lebanese amber, unknown horizon, unknown locality, Lebanon.

F. Chyromyidae (Chyromyiidae) Eoc.(Priabonian)-Holocene

First: e.g. *Gephyromyiella electrica* in von Tschirnhaus and Hoffeins (2009), Baltic amber.

F. Clusiidae Eoc.(Priabonian)-Holocene

First: e.g. *Electroclusiodes meunieri* in von Tschirnhaus and Hoffeins (2009), Baltic amber.

F. Conopidae Eoc.(Ypresian)-Holocene

First: *Poliomyia recta* in Stuke (2003), Green River Formation, Unitas area, Wyoming, United States.

F. Corethrellidae K1(Barremian)-Holocene

First: *Corethrella cretacea* in Borkent (2008), Lebanese amber, unknown horizon, unknown locality, Lebanon.

F. Cratomyiidae Mazzarolo and Amorim, 2000 K1(Aptian)
This could be a junior synonym of Zhangsolvidae (Willkommen and Grimaldi, 2007).

e.g. *Cratomyoides cretacicus* Wilkommen *in* Willkommen and Grimaldi, 2007, Crato Formation, Araripe Basin, Ceará, Brazil.

F. Crosaphididae (Crosaphidae) T3(Carnian)-J3(Oxfordian)

First: e.g. *Crosaphis anomala* in Martin (2008), Mount Crosby Formation, Ipswich Basin, Queensland, Australia. (Jell 2004 mistakenly lists this species under Aphididae.)

Last: Mentioned in Evenhuis (1994), Karabastau Formation, Karatau Range, Tien Shan mountains, Kazakhstan.

F. Cryptochetidae (Cryptochaetidae) Eoc.(Priabonian)-Holocene

First: *Phanerochaetum tuxeni* in von Tschirnhaus and Hoffeins (2009), Baltic amber.

F. Culicidae K1(Albian)-Holocene
Evenhuis (1994) lists seven doubtfully placed taxa from the Mesozoic of Germany and China, which are considered not to belong to this family by Poinar et al. (2000).

First: *Burmaculex antiquus* in Harbach (2007), Burmese amber (Burmite), Hukawng Valley, Kachin State, Myanmar.

F. Curtonotidae Eoc.(Priabonian)-Holocene
Kirk-Spriggs (2007) removed "*Curtonotum*" *gigas* (Gypse d'Aix, France) from this family.

First: Mentioned in Haenni (2003), Baltic amber.

F. Cylindrotomidae Eoc.(Ypresian)-Holocene

First: e.g. *Cylindrotoma borealis* in Evenhuis (1994), Fur Formation (Mo Clay), Limfjord/Mors Peninsula/Fur Island, Jutland, Denmark.

F. Cypselosomatidae Eoc.(Priabonian)-Holocene

First: *Cypselosomatites succini* in von Tschirnhaus and Hoffeins (2009), Baltic amber.

F. Diadocidiidae K1(Albian)-Holocene

First: *Docidiadia burmitica* Blagoderov and Grimaldi, 2004, Burmese amber (Burmite), Hukawng Valley, Kachin State, Myanmar.

F. Diopsidae Eoc.(Priabonian)-Holocene

First: e.g. *Prosphyracephala kerneggeri* Kotrba, 2009, Baltic amber.

F. Diplopolyneuridae J1(Sinemurian)
Krzemiński (1992) considered this to belong in Limoniidae but Evenhuis (1994) prefered to keep it separate, pending further study of the type species.

First and Last: *Diplopoyneura mirabilis* in Evenhuis (1994), Dzhil Formation, Sogyuty, Issyk-Kul, Kyrgyzstan.

F. Ditomyiidae (Ditomyidae) Pal.(Thanetian)-Holocene

First: *Australosymmerus imperfecta* in Jell (2004), Redbank Plains Formation, Ipswich Basin, Queensland, Australia.

F. Dixidae J1(Sinemurian)-Holocene

First: *Syndixa? liasina* Lukashevitch, 1996, Dzhil Formation, Sogyuty, Issyk-Kul, Kyrgyzstan.

F. Dolichopodidae (Microphoridae) K1(Hauterivian)-Holocene

First: e.g. *Microphorites similis* Grimaldi and Cumming, 1999, Jezzine amber, Jouar Ess-Souss, Mouhafazet Loubnan El-Janoubi, Lebanon.

F. Drosophilidae Eoc.(Priabonian)-Holocene

First: e.g. *Electrophortica succini* in von Tschirnhaus and Hoffeins (2009), Baltic amber.

F. Dryomyzidae Eoc.(Priabonian)-Holocene

First: e.g. *Prodryomyza electrica* in von Tschirnhaus and Hoffeins (2009), Baltic amber.

F. Elliidae Krzemińska et al., 1993(Eliidae) J3(Oxfordian)-K1(Valanginian)

First: *Polyanka minuta* in Krzemiński and Evenhuis (2000), Karabastau Formation, Karatau Range, Tien Shan mountains, Kazakhstan.

Last: *Ellia colorissima* in Blagoderov et al. (2002), Zaza Formation, Baissa, Buryatia, Russian Federation.

F. Empididae (Protempididae) J3(Oxfordian)-Holocene
Some disagreement exists on whether or not to put Protempididae as a subfamily of Empididae but Mostovski (2009) keeps it here, although he does not mention the species.

First: e.g. *Protempis antennata* in Grimaldi and Engel (2005), Karabastau Formation, Karatau Range, Tien Shan mountains, Kazakhstan.

F. Eoditomyidae (Eoditomyiidae) J1(Toarcian)

First and Last: *Eoditomyia primitiva* Ansorge, 1996a, Upper Lias, Grimmen, Mecklenburg-Vorpommern, Germany.

F. Eomyiidae J3(Oxfordian)-K2(Santonian)

First: *Eomyia veterrima* in Nagatomi and Yang (1998), Karabastau Formation, Karatau Range, Tien Shan mountains, Kazakhstan.

Last: Mentioned in Evenhuis (1994), Yantardakh amber, Kheta Formation, Taimyr, Krasnoyarsk Krai, Siberian Federal District, Russian Federation.

F. Eophlebomyiidae Eoc.(Ypresian)

First and Last: *Eophlebomyia claripennis* in Evenhuis (1994), Green River Formation, Unitas area, Colorado, United States.

F. Eopolyneuridae J1(Sinemurian)

e.g. *Eopolyneura tenuinervis* in Evenhuis (1994), Dzhil Formation, Sogyuty, Issyk-Kul, Kyrgyzstan.

F. Eostratiomyiidae J3(Oxfordian)

First and Last: *Eostratiomyia avia* in Mostovski et al. (2003a), Karabastau Formation, Karatau Range, Tien Shan mountains, Kazakhstan.

F. Ephydridae Eoc.(Priabonian)-Holocene

First: e.g. *Protoscinus perparvus* in Zlobin (2007), Bembridge Marls Insect Limestone, Gurnard/Thorness Bay, Isle of Wight, United Kingdom.

F. Eremochaetidae (Bremochaetidae) J3(Oxfordian)-K1(Aptian)

First: e.g. *Pareremochaetus minor* in Nagatomi and Yang (1998), Karabastau Formation, Karatau Range, Tien Shan mountains, Kazakhstan.

Last: e.g. *Alleremonomus liaoningensis* Ren and Guo, 1995, Jianshangou beds, Yixian Formation, Liaoning Province, China.

F. Gasterophilidae Eoc.(Ypresian)-Holocene
Some authors regard this as a subfamily of Oestridae.

First: Mentioned in Rognes (1997), Green River Formation, Unitas area, Colorado, United States.

F. Glossinidae Eoc.(Priabonian)-Holocene

First: e.g. *Glossina oligocena* in Grimaldi and Engel (2005), Florissant Formation, Florissant, Colorado, United States.

F. Gracilitipulidae Hong and Wang, 1990 K1(Barremian)
Blagoderov et al. (2002) note that a re-examination of the type material may result in synonymisation with Limoniidae, whereas Zhang (2006a) considers it could belong to the Chaoboridae.

First and Last: *Gracilitipula asiatica* in Evenhuis (1994), Laiyang Formation, Laiyang County, Shandong Province, China.

F. Grauvogeliidae Krzemiński et al., 1994(Grauvogelidae) T2(Anisian)

e.g. *Louisa nova* in Blagoderov et al. (2007), Grès à Voltzia, Bas-Rhin/Moselle, Northern Vosges Mountains, France.

F. Heleomyzidae (Helomyzidae, Trixoscelidae, Trixoscelididae) Eoc.(Ypresian)-Holocene

First: *Heteromyza detecta* in Evenhuis (1994), Green River Formation, Unitas area, Colorado, United States.

F. Hennigmatidae Shcherbakov *in* Shcherbakov et al., 1995(Hennigmoatidae, Kuperwoodiidae) T3(Carnian)-K1(Berriasian)
Although the Kuperwoodiinae Lukashevitch, 1995 was elevated to family status by Krzemiński and Krzemińska (2003), this was not accepted by Lukashevitch et al. (2006).

First: e.g. *Kuperwoodia benefica* in Blagoderov et al. (2007), Madygen Formation, Madygen/Dzhailoucho, south Fergana Valley, Kyrgyzstan.

Last: *Hennigma cladistorum* in Lukashevitch et al. (2006), Tsagan-Tsab, Khutel-Kara, Dornogovi (East Gobi) Aimag, Mongolia.

F. Heterorhyphidae Ansorge and Krzemiński, 1995 J1(Toarcian)

e.g. *Heterorhyphus triangularis* in Krzemiński and Evenhuis (2000), Upper Lias, Grimmen, Mecklenburg-Vorpommern, Germany.

F. Hilarimorphidae J3(Oxfordian)-Holocene

First: *Apystomima zaitzevi* in Grimaldi and Engel (2005), Karabastau Formation, Karatau Range, Tien Shan mountains, Kazakhstan.

F. Hippoboscidae Olig.(Rupelian)-Holocene

First: Figured in Prokop and Fikaček (2007), Seifhennersdorf diatomite, Upper Lusatia, Free State of Saxony, Germany. (The family placement of this species is tentative.)

F. Hoffeinsmyiidae Michelsen, 2009 Eoc.(Priabonian)

First and Last: *Hoffeinsmyia enigmatica* Michelsen, 2009, Baltic amber.

F. Hongocaloneuridae Hong, 2002a Eoc.(Ypresian)

First and Last: *Hongocaloneura plectilis* in Zhang (2007b), Fushun amber, Guchengzi, Liaoning Province, China.

F. Huaxiasciaritidae Hong, 2002a Eoc.(Ypresian)

e.g. *Huaxiasciarites longus* Hong, 2002a, Fushun amber, Guchengzi, Liaoning Province, China.

F. Hybotidae (Hybothidae) K1(Albian)-Holocene

First: e.g. *Alavesia prietoi* Peñalver and Arillo, 2007, El Caleyu amber, Ullaga Formation, central Asturian Depression, Asturias Province, Spain.

F. Hyperpolyneuridae J1(Sinemurian)

First and Last: *Hyperpolyneura phryganeoides* in Krzemiński (1992), Dzhil Formation, Sogyuty, Issyk-Kul, Kyrgyzstan.

F. Ironomyiidae K1(Valanginian)-Holocene

First: e.g. *Hermaeomyia baisica* Mostovski, 1995, Zaza Formation, Baissa, Buryatia, Russian Federation.

F. Keroplatidae (Arachnocampidae, Macroceridae) K1(Berriasian)-Holocene

First: Mentioned in Jarzembowski and Coram (1996), Purbeck Limestone Group, Dorset, England, United Kingdom.

F. Kovalevisargidae Mostovski, 1997 J3(Oxfordian)

e.g. *Kovalevisargus clarigenus* Mostovski, 1997, Karabastau Formation, Karatau Range, Tien Shan mountains, Kazakhstan.

F. Lauxaniidae (Lausaniidae) Eoc.(Priabonian)-Holocene
*Trypaneoides ellipticus* from Fushun amber probably belongs in Dolichopodidae (Blagoderov et al., 2002).

First: e.g. *Chamaelauxania succini* in von Tschirnhaus and Hoffeins (2009), Baltic amber.

F. Limnorhyphidae J2(Callovian)

First and Last: *Limnorhyphus haifanggouensis* in Zhang (2007b), Haifanggou Formation, Beipiao, Liaoning Province, China.

F. Limoniidae (Archilimoniidae, Architipulidae, Eoasilidae, Gnomuscidae) T2(Anisian)-Holocene

First: *Archilimonia vegesiana* in Blagoderov et al. (2007), Grès à Voltzia, Bas-Rhin/Moselle, Northern Vosges Mountains, France.

F. Lonchaeidae Mio.(Messinian)-Holocene

First: e.g. cf. *Dasiops* sp. in Grimaldi and Triplehorn (2008), Grubstake Formation, Suntrana Creek, Alaska, United States.

F. Lonchopteridae K1(Barremian)-Holocene

First: e.g. *Lonchopterites prisca* Grimaldi and Cumming, 1999, Bcharreh amber, Caza Bcharreh, Mouhafazet Loubnan Eshemali, Lebanon.

F. Luanpingitidae Zhang, 1986 J2(Callovian)

First and Last: *Luanpingites flavus* in Zhang (2002b), Xiahuayuan Formation, Luanping County, Hebei Province, China.

F. Lygistorrhinidae K1(Hauterivian)-Holocene

First: *Lebanognoriste prima* Blagoderov and Grimaldi, 2004, Jezzine amber, Jouar Ess-Souss, Mouhafazet Loubnan El-Janoubi, Lebanon.

F. Megamerinidae Eoc.(Priabonian)-Holocene

First: e.g. *Palaeotanypeza spinosa* in von Tschirnhaus and Hoffeins (2009), Baltic amber.

F. Mesosciophilidae J2(Aalenian)-K1(Aptian)

First: e.g. *Mesosciophilina irinae* in Li and Ren (2009), Itat Formation, Kubekovo, Krasnoyarsk Krai, Siberian Federal District, Russian Federation.

Last: "*Pseudalysiinia*" *fragmenta* in Li and Ren (2009), Koonwarra Fossil Bed (Korumburra Group), South Gippsland, Victoria, Australia.

F. Micropezidae (Calobatidae) Eoc.(Priabonian)-Holocene

First: e.g. *Electrobata tertiaria* in von Tschirnhaus and Hoffeins (2009), Baltic amber.

F. Milichiidae (Milichidae, Phyllomyzidae) K2(Maastrichtian)-Holocene

First: Mentioned in Engel (2000), Kinkora amber, formation unknown, New Jersey, United States.

F. Muscidae Eoc.(Ypresian)-Holocene

First: *Acanthomyites aldrichi* in Evenhuis (1994), Green River Formation, Unitas area, Colorado, United States.

F. Musidoromimidae J1(Sinemurian)

First and Last: *Musidoromima crassinervis* in Evenhuis (1994), Dzhil Formation, Sogyuty, Issyk-Kul, Kyrgyzstan.

F. Mycetophilidae (Sciophilidae) K1(Valanginian)-Holocene
'*Prodocidia spectra*' Whalley, 1985 from the Lower Lias of Charmouth was moved to Ptychopteridae: Eoptychopterinae (Lukashevitch, 2000, 2008).

First: e.g. *Ipsaneusidalys communis* Blagoderov, 1998, Zaza Formation, Baissa, Buryatia, Russian Federation.

F. Mydidae (Mydaidae, Mydasidae) K1(Valanginian)-Holocene

First: Mentioned in Mostovski (2009), Zaza Formation, Baissa, Buryatia, Russian Federation.

F. Mythicomyiidae J2(Aalenian)-Holocene

First: *Palaeoplatypygus zaitzevi* in Evenhuis (2002), Itat Formation, Kubekovo, Krasnoyarsk Krai, Siberian Federal District, Russian Federation.

F. Nadipteridae Lukashevitch *in* Shcherbakov et al., 1995 T2(Anisian)-J1(Sinemurian)

First: *Tanus triassicus* in Blagoderov et al. (2007), Grès à Voltzia, Bas-Rhin/Moselle, Northern Vosges Mountains, France.

Last: *Nadiptera anachrona* in Krzemiński and Krzemińska (2003), Dzhil Formation, Sogyuty, Issyk-Kul, Kyrgyzstan.

F. Natalimyzidae Barraclough and McAlpine, 2006 Eoc.(Priabonian)-Holocene

First: *Natalimyza* sp. in von Tschirnhaus and Hoffeins (2009), Baltic amber.

F. Nemestrinidae J1(Toarcian)-Holocene

First: Mentioned in Grimaldi and Engel (2005), Upper Lias, Grimmen, Mecklenburg-Vorpommern, Germany.

F. Neriidae Mio.(Aquitanian)-Holocene

First: Mentioned in Engel (2004a), Mexican amber, Simojovel, Chiapas, Mexico.

F. Neurochaetidae Eoc.(Priabonian)-Holocene

First: e.g. *Anthoclusia gephyrea* in von Tschirnhaus and Hoffeins (2009), Baltic amber.

F. Nymphomyiidae Eoc.(Priabonian)-Holocene

First: *Nymphomyia succina* Wagner et al., 2000, Baltic amber.

F. Odiniidae Eoc.(Priabonian)-Holocene

First: e.g. *Protodinia electrica* in von Tschirnhaus and Hoffeins (2009), Baltic amber.

F. Oestridae Eoc.(Ypresian)-Holocene

First: e.g. *Cuterebra ascarides* in Rognes (1997), Green River Formation, Unitas area, Colorado, United States.

F. Oligophrynidae (Oligophryneidae) J1(Sinemurian)

e.g. *Oligophryne britannica* in Krzemiński and Ansorge (2005), Black Ven Marls, Charmouth, Dorset, United Kingdom.

F. Opetiidae K1(Berriasian)-Holocene

First: *Opetiala shatalkini* Coram et al., 2000, Durlston Formation (Stair Hole Member), Durlston Bay, Dorset, United Kingdom. (Although Grimaldi and Engel 2005 (p.533) suggest this species may be too primitive to be placed here, Mostovski 2009 maintains it in Opetiidae.)

F. Opomyzidae Olig.(Chattian)-Holocene

First: e.g. *Opomyza pelidua* in Evenhuis (1994), Rott paper shales, Bonn, North Rhine-Westphalia, Germany.

F. Pachyneuridae (Cramptonomyiidae) J3(Oxfordian)-Holocene

First: e.g. *Tega karatavica* in Krzemiński and Evenhuis (2000), Karabastau Formation, Karatau Range, Tien Shan mountains, Kazakhstan.

F. Palaeophoridae (Paleophoridae) J3(Oxfordian)

First and Last: *Palaeophora ancestrix* in Mostovski (1999), Karabastau Formation, Karatau Range, Tien Shan mountains, Kazakhstan.

F. Pallopteridae Eoc.(Priabonian)-Holocene

First: e.g. *Glaesolonchea electrica* in Grimaldi and Triplehorn (2008), Baltic amber.

F. Parapleciidae J2(Callovian)

First and Last: *Paraplecia ovata* in Zhang (2002b), Haifanggou Formation, Beipiao, Liaoning Province, China.

F. Paraxymyiidae (Eomycetophilidae) T3(Carnian)-J3(Tithonian)
Mentions of Cretaceous specimens are referring to the Glushkovo Fm., as some authors consider it J3/K1.

First: e.g. *Veriplecia rugosa* Blagoderov & Grimaldi *in* Blagoderov et al., 2007, Cow Branch Formation, Solite quarry, Virginia, United States.

Last: *Eomycetophila asymmetrica* in Blagoderov (1999), Glushkovo Formation, Daya, Transbaikalia, Russian Federation.

F. Pediciidae J2(Aalenian)-Holocene

First: *Praearchitipula notabilis* in Krzemiński and Evenhuis (2000), Itat Formation, Kubekovo, Krasnoyarsk Krai, Siberian Federal District, Russian Federation.

F. Periscelididae (Periscelidae, Stenomicridae) Eoc.(Priabonian)-Holocene

First: e.g. *Procyamops succini* in von Tschirnhaus and Hoffeins (2009), Baltic amber.

F. Perissommatidae J2(Aalenian)-Holocene

First: *Palaeoperissomma collessi* in Lukashevitch et al. (2006), Itat Formation, Kubekovo, Krasnoyarsk Krai, Siberian Federal District, Russian Federation.

F. Phoridae (Sciadoceridae) K1(Albian)-Holocene

First: e.g. *Euliphora grimaldii* in Delclòs et al. (2007), Álava amber, Escucha Formation, Basco-Cantabrian Basin, Álava Province, Spain.

F. Piophilidae Eoc.(Priabonian)-Holocene

First: *Mycetaulus incretus* in Meyer (2003), Florissant Formation, Florissant, Colorado, United States.

F. Pipunculidae K2(Campanian)-Holocene

First: Mentioned in Poinar and Poinar (2008), Canadian amber, Cedar Lake, Manitoba, Canada. (This is not mentioned in McKellar et al. 2008.)

F. Platypezidae K1(Valanginian)-Holocene

First: e.g. *Proplatypeza parva* in Grimaldi and Cumming (1999), Zaza Formation, Baissa, Buryatia, Russian Federation.

F. Platystomatidae Pleist.(Upper Pleistocene)-Holocene

First: e.g. *Scholastes foordi* in Gentilini et al. (2006), Tanzanian copal, Tanzanian copal, Tanzanian copal, Tanzania.

F. Pleciodictyidae J1(Sinemurian)

First and Last: *Pleciodictya modesta* in Evenhuis (1994), Dzhil Formation, Sogyuty, Issyk-Kul, Kyrgyzstan.

F. Pleciofungivoridae (Fungivoritidae) J1(Sinemurian)-J3(Tithonian)
Allactoneuridae is not a junior synonym of this family and is Recent-only, according to Sabrosky et al. (1999).

First: e.g. *Archihesperinus phryneoides* in Evenhuis (1994), Dzhil Formation, Sogyuty, Issyk-Kul, Kyrgyzstan.

Last: e.g. *Bryanka lepida* in Evenhuis (1994), Glushkovo Formation, Daya, Transbaikalia, Russian Federation.

F. Procramptonomyiidae (Alinkidae) T3(Carnian)-K1(Berriasian)

First: e.g. *Yalea rectimedia* Blagoderov & Grimaldi *in* Blagoderov et al., 2007, Cow Branch Formation, Solite quarry, Virginia, United States.

Last: e.g. *Procramptonomyia zigzagensis* Coram and Jarzembowski, 1999, Durlston Formation (Stair Hole Member), Durlston Bay, Dorset, United Kingdom.

F. Proneottiophilidae Eoc.(Priabonian)

e.g. *Proneottiophilum extinctum* in von Tschirnhaus and Hoffeins (2009), Baltic amber.

F. Prosechamyiidae Blagoderov et al., 2007 T3(Carnian)

e.g. *Prosechamyia trimedia* Blagoderov & Grimaldi *in* Blagoderov et al., 2007, Cow Branch Formation, Solite quarry, Virginia, United States.

F. Protapioceridae Ren, 1998 K1(Aptian)

e.g. *Protapiocera convergens* Zhang et al., 2007, Jianshangou beds, Yixian Formation, Liaoning Province, China.

F. Protendipedidae J3(Oxfordian)-K1(Hauterivian)

First: *Protendipes dasypterus* in Evenhuis (1994), Karabastau Formation, Karatau Range, Tien Shan mountains, Kazakhstan. (Evenhuis 1994 mistakenly states that this species was found in the Lower Jurassic of Issyk-Kul, Kyrgyzstan. Rohdendorf 1991 lists it in Karatau as do Blagoderov et al. 2002.)

Last: *Priscotendipes mirus* in Zhang et al. (2010), Dabeigou Formation, Luanping County, Hebei Province, China.

F. Protobibionidae J3(Oxfordian)-K1(Barremian)
Usually considered to belong within Chironomidae, Evenhuis (1994) treats Protobibionidae as a separate family.

First: *Protobibio jurassicus* in Evenhuis (1994), Karabastau Formation, Karatau Range, Tien Shan mountains, Kazakhstan.

Last: *Protobibio orientalis* in Evenhuis (1994), Laiyang Formation, Laiyang County, Shandong Province, China. (Evenhuis 1994 notes that this species requires additional study to confirm its generic placement.)

F. Protobrachyceridae (Protobrachycerontidae) J1(Toarcian)-J2(Callovian)

First: e.g. *Protobrachyceron zessini* in Zhang et al. (2008), Upper Lias, Dobbertin, Mecklenburg-Vorpommern, Germany.

Last: *Protobrachyceron sinensis* Zhang et al., 2008, Jiulongshan Formation, near Daohugou, Ningcheng county, Inner Mongolia, China.

F. Protomphralidae J3(Oxfordian)
Nagatomi and Yang (1998) rejected *Mesomphrale asiaticum* from this family.

First and Last: *Protomphrale martynovi* in Nagatomi and Yang (1998), Karabastau Formation, Karatau Range, Tien Shan mountains, Kazakhstan.

F. Protopleciidae (Dyspolyneuridae, Palaeopleciidae, Phragmneuridae, Phragmoligoneuridae, Protoligoneuridae) J1(Sinemurian)-J3(Tithonian)
Zhang (2007a) mentions that *Lichnoplecia kovalevi* is likely Protopleciidae but then leaves it in Bibionidae.

First: e.g. *Palaeoplecia rhaetica* in Zhang (2007a), Dzhil Formation, Sogyuty, Issyk-Kul, Kyrgyzstan.

Last: *Mesoplecia oleynikovi* in Zhang (2007a), Glushkovo Formation, Savina, Transbaikalia, Russian Federation.

F. Protorhyphidae (Vimrhyphidae) T2(Anisian)-J3(Tithonian)

First: *Vymrhyphus blagoderovi* in Martin (2008), Grès à Voltzia, Bas-Rhin/Moselle, Northern Vosges Mountains, France.

Last: *Protorhyphus major* in Zhang (2007b), Glushkovo Formation, Daya, Transbaikalia, Russian Federation.

F. Protoscatopsidae J2(Aalenian)-J3(Oxfordian)

First: *Mesoscatopse rohdendorfi* in Amorim (2008), Ichetuy Formation, Novospasskoye, Mukhorshibirsky District, Buryatia, Russian Federation.

Last: *Protoscatopse jurassica* in Amorim (2008), Karabastau Formation, Karatau Range, Tien Shan mountains, Kazakhstan.

F. Pseudopomyzidae Eoc.(Priabonian)-Holocene

First: e.g. *Eopseudopomyza kuehnei* in von Tschirnhaus and Hoffeins (2009), Baltic amber.

F. Psilidae Eoc.(Priabonian)-Holocene

First: e.g. *Electrochyliza succini* in von Tschirnhaus and Hoffeins (2009), Baltic amber.

F. Psychodidae (Phlebotomidae) T3(Carnian)-Holocene

First: *Triassopsychoda olseni* Blagoderov & Grimaldi *in* Blagoderov et al., 2007, Cow Branch Formation, Solite quarry, Virginia, United States.

F. Psychotipidae Shcherbakov *in* Shcherbakov et al., 1995 T3(Carnian)
Elevated to family status by Krzemiński and Krzemińska (2003). Although *Psychotipa* was listed under Limoniidae by Blagoderov et al. (2007), this family has not been formally synonymised.

e.g. *Psychotipa predicta* in Krzemiński and Krzemińska (2003), Madygen Formation, Madygen/Dzhailoucho, south Fergana Valley, Kyrgyzstan.

F. Ptychopteridae (Architendipedidae, Eolimnobiidae, Eoptychopteridae) J1(Sinemurian)-Holocene
The family Eoptychopteridae was synonymised by Lukashevitch (2008). Lukashevitch (2008) doubts the assignment to this family of a specimen from the Triassic (Carnian) Cow Branch Formation, Virginia, USA.

First: e.g. *Eoptychoptera? spectra* in Lukashevitch (2000), Dzhil Formation, Sogyuty, Issyk-Kul, Kyrgyzstan.

F. Pyrgotidae Eoc.(Priabonian)-Holocene

First: e.g. Mentioned in von Tschirnhaus and Hoffeins (2009), Baltic amber.

F. Rangomaramidae Jaschhof and Didham, 2002 Eoc.(Priabonian)-Holocene
*Heterotricha* was included in this family by Rindal (2007).

First: e.g. *Heterotricha hirta* in Chandler (2002), Baltic amber.

F. Rhaetaniidae Krzemiński and Krzemińska, 2002 T3(Rhaetian)

First and Last: *Rhaetania dianae* in Blagoderov et al. (2007), Cotham Member, Lilstock Formation, Penarth Group1, Strensham, Worcestershire, United Kingdom.

F. Rhagionemestriidae J3(Oxfordian)-K1(Barremian)

First: e.g. *Nagatommukha karabas* Mostovski and Martínez-Delclòs, 2000, Karabastau Formation, Karatau Range, Tien Shan mountains, Kazakhstan.

Last: *Iberomosca kakoeima* Mostovski and Martínez-Delclòs, 2000, Montsec lithographic limestones, Montsec Range, Lleida Province, Spain.

F. Rhagionempididae J3(Oxfordian)-J3(Tithonian)
There seems to be some confusion over whether this family is extant or not. Evenhuis (1994) makes it clear this is because of homonomy of an extant genus of Apsilocephalidae with the type genus of Rhagionempididae (*Rhagionempis*). Specimens in Evenhuis (1994) listed as Middle Jurassic are from the Uda Formation (Oxfordian).

First: e.g. *Probolbomyia modesta* in Mostovski (2009), Karabastau Formation, Karatau Range, Tien Shan mountains, Kazakhstan.

Last: *Shevioptera sinitsae* in Evenhuis (1994), Ukurey Formation (=Glushkovo?), Olov Depression, Transbaikalia, Russian Federation.

F. Rhagionidae (Palaeostratiomyidae, Palaeostratiomyiidae) J1(Pliensbachian)-Holocene
Blagoderov et al. (2007) do not consider the Middle Triassic species *Gallia alsatica* Krzemiński and Krzemińska, 2003 to belong to this family.

First: *Palaeobrachyceron nagatomii* in Nagatomi and Yang (1998), Abashevo Formation, Chernyi Etap, Kemerovo Region, Russian Federation.

F. Richardiidae Eoc.(Priabonian)-Holocene

First: e.g. *Pachysomites inermis* in Meyer (2003), Florissant Formation, Florissant, Colorado, United States.

F. Sarcophagidae Eoc.(Priabonian)-Holocene
Zherikhin (2002c) mentions the "complete absense of fossil" Sarcophagidae.

First: Mentioned in Wichard and Weitschat (1996), Baltic amber.

F. Scathophagidae (Scatophagidae) Eoc.(Priabonian)-Holocene
Zherikhin (2002c) doubts the records of this family from Baltic amber and Florrisant.

First: e.g. *Cordylura exhumata* in Meyer (2003), Baltic amber.

F. Scatopsidae K1(Barremian)-Holocene

First: Figured in Azar (2007), Lebanese amber, unknown horizon, unknown locality, Lebanon.

F. Scenopinidae J3(Oxfordian)-Holocene

First: Mentioned in Mostovski (2009), Karabastau Formation, Karatau Range, Tien Shan mountains, Kazakhstan.

F. Sciaridae (Archizelmiridae, Sciaroidae) J3(Oxfordian)-Holocene

First: *Archizelmira kazachstanica* in Grimaldi et al. (2003), Karabastau Formation, Karatau Range, Tien Shan mountains, Kazakhstan.

F. Sciomyzidae K1(Barremian)-Holocene

First: e.g. Mentioned in Blagoderov and Martínez-Delclòs (2001), Montsec lithographic limestones, Montsec Range, Lleida Province, Spain. (Zherikhin 2002c considers the family placement of these species as doubtful.)

F. Sepsidae Eoc.(Priabonian)-Holocene

First: e.g. *Themira saxifica* in Meyer (2003), Florissant Formation, Florissant, Colorado, United States.

F. Serendipidae Evenhuis, 1994(Paratendipedidae) K1(Barremian)

e.g. *Serendipa laiyangensis* in Brooks and Evenhuis (1995), Laiyang Formation, Laiyang County, Shandong Province, China.

F. Siberhyphidae Kovalev *in* Kalugina and Kovalev, 1985(Syberhyphidae) J2(Aalenian)

First and Last: *Siberhyphus lebedevi* in Krzemiński and Evenhuis (2000), Itat Formation, Kubekovo, Krasnoyarsk Krai, Siberian Federal District, Russian Federation.

F. Simuliidae (Simulidae) J2(Aalenian)-Holocene

First: *Simulimima grandis* in Lukashevitch (2008), Ichetuy Formation, Novospasskoye, Mukhorshibirsky District, Buryatia, Russian Federation.

F. Sinoditomyiidae Hong, 2002a Eoc.(Ypresian)

e.g. *Sinoditomyia maculosa* Hong, 2002a, Fushun amber, Guchengzi, Liaoning Province, China.

F. Sinonemestriidae Nagatomi and Yang, 1998 K1(Barremian)

First and Last: *Sinonemestrius tuanwangensis* in Nagatomi and Yang (1998), Laiyang Formation, Laiyang County, Shandong Province, China.

F. Sinotendipedidae Hong and Wang, 1990(Sinotendipidae) K1(Barremian)

First and Last: *Sinotendipes tuanwangensis* in Evenhuis (1994), Laiyang Formation, Laiyang County, Shandong Province, China.

F. Spaniidae K1(Albian)-Holocene

First: *Litoleptis fossilis* Arillo et al., 2009, San Just amber, Escucha Formation, Maestrat Basin, Teruel Province, Spain.

F. Sphaeroceridae (Borboridae) Eoc.(Priabonian)-Holocene

First: e.g. *Sphaerocera sepultula* in Evenhuis (1994), Bembridge Marls Insect Limestone, Gurnard/Thorness Bay, Isle of Wight, United Kingdom.

F. Stratiomyidae (Stratiomyiidae, Stratiomyriidae) J3(Oxfordian)-Holocene

First: Mentioned in Mostovski (2009), Karabastau Formation, Karatau Range, Tien Shan mountains, Kazakhstan.

F. Syringogastridae Mio.(Burdigalian)-Holocene

First: e.g. *Syringogaster miocenecus* Grimaldi *in* Marshall et al., 2009, Dominican amber, Cordillera Septentrional, near Santiago, Dominican Republic.

F. Syrphidae K2(Santonian)-Holocene
The Jordanian amber record figured in Kaddumi (2005) is doubtful.

First: Mentioned in Grimaldi and Engel (2005), Yantardakh amber, Kheta Formation, Taimyr, Krasnoyarsk Krai, Siberian Federal District, Russian Federation.

F. Tabanidae K1(Berriasian)-Holocene

First: *Eotabanoid lordi* Mostovski et al., 2003a, Durlston Formation (Stair Hole Member), Durlston Bay, Dorset, United Kingdom.

F. Tachinidae Eoc.(Ypresian)-Holocene
Zherikhin (2002c) considers Palaeogene finds "highly questionable" (p.384).

First: *Vinculomusca vinculata* in Rognes (1997), Green River Formation, Unitas area, Colorado, United States.

F. Tanyderidae J1(Toarcian)-Holocene

First: e.g. *Nannotanyderus grimmenensis* Ansorge and Krzemiński, 2002, Upper Lias, Grimmen, Mecklenburg-Vorpommern, Germany.

F. Tanyderophrynidae (Tanyderophryneidae) J3(Oxfordian)

First and Last: *Tanyderophryne multinervis* in Evenhuis (1994), Karabastau Formation, Karatau Range, Tien Shan mountains, Kazakhstan.

F. Tephritidae Mio.(Burdigalian)-Holocene

First: e.g. *Ceratodaucus priscus* in Arillo and Ortuño (2005), Dominican amber, Cordillera Septentrional, near Santiago, Dominican Republic.

F. Tethepomyiidae Grimaldi and Arillo, 2008 K1(Albian)-K2(Turonian)

First: e.g. *Tethepomima holomma* Grimaldi and Arillo, 2008, Álava amber, Escucha Formation, Basco-Cantabrian Basin, Álava Province, Spain.

Last: *Tethepomyia thauma* in Grimaldi and Arillo (2008), New Jersey amber, South Amboy Fire Clay (Raritan Formation), New Jersey, United States.

F. Tethinidae Mio.(Aquitanian)-Holocene

First: Mentioned in Solórzano Kraemer (2007), Mexican amber, Simojovel, Chiapas, Mexico.

F. Thaumaleidae (Thaumalaeidae) J3(Tithonian)-Holocene

First: *Mesothaumalea fossilis* in Wagner et al. (2008), Glushkovo Formation, Daya, Transbaikalia, Russian Federation.

F. Therevidae J3(Oxfordian)-Holocene

First: *Rhagiophryne bianalis* in Mostovski (2009), Karabastau Formation, Karatau Range, Tien Shan mountains, Kazakhstan.

F. Tillyardipteridae Lukashevitch and Shcherbakov, 1999 T3(Carnian)

First and Last: *Tillyardiptera prima* in Blagoderov et al. (2007), Mount Crosby Formation, Ipswich Basin, Queensland, Australia.

F. Tipulidae K1(Albian)-Holocene
Considered here in the strict sense, not including Limoniidae or Cylindrotomidae.

First: e.g. Mentioned in Perrichot (2004), Archingeay amber, Archingeay-Les Nouillers, Charente-Maritime, France. (It is not certain from the text if these specimens are Tipulidae *sensu stricto*.)

F. Tipulodictyidae J1(Sinemurian)

First and Last: *Tipulodictya minima* in Evenhuis (1994), Dzhil Formation, Sogyuty, Issyk-Kul, Kyrgyzstan.

F. Tipulopleciidae J3(Oxfordian)

First and Last: *Tipuloplecia breviventris* in Evenhuis (1994), Karabastau Formation, Karatau Range, Tien Shan mountains, Kazakhstan.

F. Trichoceridae J1(Toarcian)-Holocene

First: e.g. *Mailotrichocera mikereichi* Krzemińska, Krzemiński & Ansorge *in* Krzemińska et al., 2009, Upper Lias, Dobbertin, Mecklenburg-Vorpommern, Germany.

F. Ulidiidae (Otitidae, Pterocallidae) Eoc.(Priabonian)-Holocene

First: e.g. *Melieria atavina* in Meyer (2003), Florissant Formation, Florissant, Colorado, United States.

F. Valeseguyidae Amorim and Grimaldi, 2006 K1(Albian)-Holocene

First: *Cretoseguya burmitica* Amorim and Grimaldi, 2006, Burmese amber (Burmite), Hukawng Valley, Kachin State, Myanmar.

F. Vermileonidae J2(Aalenian)-Holocene
*Protobrachyceron* spp. (Toarcian, Grimmen) are in the Protobrachyceridae. See Krzemiński and Ansorge (2000) for details.

First: Mentioned in Evenhuis (1994), Itat Formation, Kubekovo, Krasnoyarsk Krai, Siberian Federal District, Russian Federation.

F. Vladipteridae Shcherbakov *in* Shcherbakov et al., 1995 T2(Ladinian)-T3(Norian)
Considered to be mecopteran by Krzemiński and Krzemińska (2003).

First: *Triassochoristites jinsuoguanensis* in Blagoderov et al. (2007), Tongchuan Formation, Hejiafang, Tongchuan District, Shaanxi Province, China. (This genus and species was originally described by Hong and Guo 2003 in Mecoptera: Mesopanorpodidae.)

Last: *Vladiptera kovalevi* in Blagoderov et al. (2007), Tologoy Formation, Ak-Kolka River, Kenderlyk, Zaisan District, Kazakhstan.

F. Xylomyidae (Solvidae) J3(Oxfordian)-Holocene

First: *Xylomya? shcherbakovi* in Grimaldi and Engel (2005), Karabastau Formation, Karatau Range, Tien Shan mountains, Kazakhstan.

F. Xylophagidae (Coenomyiidae, Rachiceridae) J3(Oxfordian)-Holocene

First: *Ganeopteromyia calypso* in Grimaldi and Engel (2005), Karabastau Formation, Karatau Range, Tien Shan mountains, Kazakhstan.

F. Zhangobiidae Evenhuis, 1994(Palaeolimnobiidae) K1(Barremian)
Blagoderov et al. (2002) note that a re-examination of the type material may result in synonymisation with Limoniidae.

e.g. *Zhangobia laiyangensis* in Sabrosky et al. (1999), Laiyang Formation, Laiyang County, Shandong Province, China.

F. Zhangsolvidae Nagatomi and Yang, 1998 K1(Barremian)

First and Last: *Zhangsolva cupressa* in Nagatomi and Yang (1998), Laiyang Formation, Laiyang County, Shandong Province, China.

**O. Hymenoptera** Linnaeus, 1758 (Vespida) Triassic(Carnian)-Quaternary(Holocene)

Plumariidae are presently unknown in the fossil record (Engel and Grimaldi, 2006b). The hierarchical classification of Evanoidea given in Zhang and Rasnitsyn (2008) is followed here but see Engel (2006b) and Jennings and Korgmann (2009) for alternative views.

F. Agaonidae (Agaontidae) Mio.(Burdigalian)-Holocene
"*Tetrapus*" *mayri* from the Florissant Formation does not belong in this family (Lopez-Vaamonde et al., 2009).

First: e.g. *Tetrapus delclosi* in Pérez-Gelabert (2008), Dominican amber, Cordillera Septentrional, near Santiago, Dominican Republic.

F. Ampulicidae K1(Barremian)-Holocene

First: Mentioned in Ohl (2004), Lebanese amber, unknown horizon, unknown locality, Lebanon.

F. Anaxyelidae J2(Callovian)-Holocene

First: Mentioned in Ortega-Blanco et al. (2008), Jiulongshan Formation, near Daohugou, Ningcheng county, Inner Mongolia, China.

F. Andreneliidae Rasnitsyn and Martínez-Delclòs, 2000 K1(Barremian)

First and Last: *Andrenelia pinnata* in Zhang and Rasnitsyn (2008), Montsec lithographic limestones, Montsec Range, Lleida Province, Spain.

F. Andrenidae Eoc.(Priabonian)-Holocene
Engel (2001) considered species attributed to this family from Florissant and the Baltic amber to be dubiously assigned and requiring further work.

First: e.g. *Libellulapis antiquorum* in Engel (2001), Florissant Formation, Florissant, Colorado, United States.

F. Angarosphecidae Rasnitsyn, 1975(Baissodidae) K1(Berriasian)-Eoc.(Ypresian)
Previously treated as a subfamily of Sphecidae *sensu lato* and represents a paraphyletic grade leading to other apoid families (Bennett and Engel, 2006).

First: e.g. *Pompilopterus wimbledoni* Rasnitsyn & Jarzembowski *in* Rasnitsyn et al., 1998, Lulworth Formation, Durlston Bay, Dorset, United Kingdom.

Last: *Eosphecium naumanni* Pulawski et al., 2000, coldwater beds of the Kamloops Group, Quilchena, British Columbia, Canada. (Bennett and Engel 2006 consider that this species could be a plesiomorphic species of Sphecidae or Crabronidae.)

F. Aphelinidae Eoc.(Priabonian)-Holocene

First: Mentioned in Perkovsky et al. (2007), Rovno amber, Klesov/Dubrovitsa, Rivne Oblast, Ukraine.

F. Apidae (Anthophoridae, Bombidae, Ctenoplectridae, Xylocopidae) K1(Aptian)-Holocene
*Ctenoplectra*, the type genus of Ctenoplectrini, was previously placed in Mellitidae with *Ctenoplectrella*. However, *Ctenoplectrella* belongs in Apidae (Engel, 2001).

First: Figured in Osten (2007), Crato Formation, Araripe Basin, Ceará, Brazil.

F. Archaeocynipidae Rasnitsyn and Kovalev, 1988 K1(Valanginian)

e.g. *Archaeocynips villosa* Rasnitsyn and Kovalev, 1988, Zaza Formation, Baissa, Buryatia, Russian Federation.

F. Argidae Eoc.(Priabonian)-Holocene
An older fossil potentially of this family is *Manevalia pachyliformis* from the Thanetian of Menat, France, belonging either to Argidae or Pterygophoridae (Nel, 2004).

First: *Sterictiphora konowi* in Nel (2004), Florissant Formation, Florissant, Colorado, United States.

F. Armaniidae K1(Albian)-K2(Turonian)
The status of this taxon remains controversial. Some authors (e.g Archibald et al., 2006) consider it to be a subfamily of Formicidae.

First: e.g. *Khetania mandibulata* in Engel and Grimaldi (2005), Emanra Formation, Khetana River, Khabarovsk Province, Russian Federation.

Last: e.g. *Orapia minor* in Engel and Grimaldi (2005), Orapa diamond mines, Orapa, Orapa, Botswana.

F. Austroniidae (Trupochalcididae, Trupochalcidiidae) K1(Valanginian)-Holocene

First: Figured in Rasnitsyn (2002i), Zaza Formation, Baissa, Buryatia, Russian Federation.

F. Bethylidae K1(Valanginian)-Holocene

First: *Cretobethylellus lucidus* in Perrichot and Nel (2008a), Gidari (Ghidari) Formation, Pavlovka, Transbaikalia, Russian Federation.

F. Bethylonymidae J3(Oxfordian)-K2(Turonian)

First: e.g. *Bethylonymellus cervicalis* in Rasnitsyn (2002i), Karabastau Formation, Karatau Range, Tien Shan mountains, Kazakhstan.

Last: Mentioned in Brothers and Rasnitsyn (2003), Orapa diamond mines, Orapa, Orapa, Botswana.

F. Blasticotomidae Eoc.(Priabonian)-Holocene

First: *Runaria ostenta* in Meyer (2003), Florissant Formation, Florissant, Colorado, United States.

F. Brachyceritidae Hong, 2002a Eoc.(Ypresian)

First and Last: *Brachycerites furvus* Hong, 2002a, Fushun amber, Guchengzi, Liaoning Province, China.

F. Braconidae (Aphidiidae, Brachonidae, Branconidae, Eoichneumonidae) K1(Berriasian)-Holocene
The Eoichneumonidae were synonymised with Braconidae by Perrichot et al. (2009).

First: e.g. *Purichneumon britannicus* Rasnitsyn & Jarzembowski *in* Rasnitsyn et al., 1998, Durlston Formation (Stair Hole Member), Durlston Bay, Dorset, United Kingdom.

F. Cephidae K1(Valanginian)-Holocene

First: *Mesocephus sibiricus* in Zherikhin (2002c), Zaza Formation, Baissa, Buryatia, Russian Federation.

F. Ceraphronidae K1(Barremian)-Holocene

First: Figured in Poinar and Milki (2001), Lebanese amber, unknown horizon, unknown locality, Lebanon.

F. Chalcididae (Chalcidae) Eoc.(Priabonian)-Holocene
Heraty and Darling (2009) state that there are no Chalcididae known from the Cretaceous and that a record of this family from Lebanese amber probably belongs to the Tetracampidae.

First: e.g. *Chalcis perdita* in Meyer (2003), Florissant Formation, Florissant, Colorado, United States.

F. Chrysididae K1(Hauterivian)-Holocene

First: *Dahurochrysis veta* in Ross and Jarzembowski (1993), Turga Formation, Turga River, near Borzai, Transbaikalia, Russian Federation.

F. Cimbicidae Pal.(Thanetian)-Holocene

First: *Cenocimbex menatensis* Nel, 2004, spongo-diatomaceous maar, Menat, Puy-de-Dôme, Auvergne, France.

F. Cleistogastridae (Brachycleistogastridae, Sinoryssidae) J2(Aalenian)-K2(Turonian)
The position of this family remains uncertain but is not placed in Megalyridae (Perrichot, 2009). "*Mesaulacinus*" *rasnitsyni* (Yixian Formation, Chengde) is considered Apocrita *incertae sedis* until re-study of the type specimen Rasnitsyn (2008).

First: *Cleistogaster buriatica* in Rasnitsyn et al. (2003), Ichetuy Formation, Novospasskoye, Mukhorshibirsky District, Buryatia, Russian Federation.

Last: Mentioned in Brothers and Rasnitsyn (2003), Orapa diamond mines, Orapa, Orapa, Botswana.

F. Colletidae (Stenotritidae) Mio.(Burdigalian)-Holocene

First: e.g. *Chilicola electrodominicana* in Arillo and Ortuño (2005), Dominican amber, Cordillera Septentrional, near Santiago, Dominican Republic.

F. Crabronidae (Astatidae, Larridae, Pemphredonidae, Philanthidae, Trypoxylidae) K1(Berriasian)-Holocene

First: *Iwestia provecta* Rasnitsyn & Jarzembowski *in* Rasnitsyn et al., 1998, Lulworth Formation, Durlston Bay, Dorset, United Kingdom. (Rasnitsyn et al. 1998 note that this specimen may lie close to Pemphredonina which here is considered in Crabronidae. The Catalog of Sphecidae [`http://research.calacademy.org/ent/catalog_sphecidae`] lists this specimen in Crabronidae.)

F. Cynipidae K2(Campanian)-Holocene

First: *Tanaoknemus ecarinatus* Liu & Engel *in* Liu et al., 2007b, Canadian amber, Medicine Hat, Alberta, Canada.

F. Daohugoidae Rasnitsyn and Zhang, 2004b J2(Callovian)

First and Last: *Daohugoa tobiasi* Rasnitsyn and Zhang, 2004b, Jiulongshan Formation, near Daohugou, Ningcheng county, Inner Mongolia, China.

F. Diapriidae K1(Aptian)-Holocene
*Cretacoformica explicata* (Koonwarra fossil beds) and *Coramia minuta* (Durlston Formation) do not belong to this family (Perrichot and Nel, 2008b).

First: *Cretapria tsukadai* in Perrichot and Nel (2008b), Choshi amber, Toriakeura Formation, Chiba, Japan.

F. Diprionidae Eoc.(Ypresian)-Holocene

First: Mentioned in Nel (2004), Green River Formation, Unitas area, Colorado, United States.

F. Dryinidae K1(Barremian)-Holocene

First: *Aphelopus palaeophoenicius* in Engel (2003a), Lebanese amber, unknown horizon, unknown locality, Lebanon.

F. Electrotomidae Eoc.(Priabonian)

First and Last: *Electrotoma succini* in Zherikhin (2002c), Baltic amber.

F. Embolemidae K1(Valanginian)-Holocene

First: e.g. *Baissobius minimus* Rasnitsyn, 1996, Zaza Formation, Baissa, Buryatia, Russian Federation.

F. Encyrtidae Eoc.(Priabonian)-Holocene

First: e.g. *Eocencnemus vichrenkoi* Simutnik *in* Simutnik and Perkovsky, 2006, Rovno amber, Klesov/Dubrovitsa, Rivne Oblast, Ukraine.

F. Eostephanitidae Hong, 2002a Eoc.(Ypresian)

First and Last: *Eostephanites tenuis* Hong, 2002a, Fushun amber, Guchengzi, Liaoning Province, China.

F. Ephialtitidae (Karataidae) J1(Toarcian)-K1(Aptian)

First: e.g. *Thilopterus lampei* Rasnitsyn et al., 2003, Upper Lias, Schandelah, Lower Saxony, Germany.

Last: *Cratephialtites kourios* in Osten (2007), Crato Formation, Araripe Basin, Ceará, Brazil.

F. Eucharitidae Eoc.(Priabonian)-Holocene

First: *Palaeocharis rex* Heraty and Darling, 2009, Baltic amber.

F. Eulophidae (Aphelidae) K1(Albian)-Holocene

First: Mentioned in Koteja and Poinar (2001), Alaskan amber, Kuk deposits, Brooks Range, Alaska, United States.

F. Eupelmidae K2(Campanian)-Holocene
The Jordanian amber record figured in Kaddumi (2005) is doubtful.

First: Mentioned in McKellar et al. (2008), Canadian amber, Grassy Lake, Alberta, Canada.

F. Eurytomidae Eoc.(Ypresian)-Holocene

First: e.g. *Eoeurytomites badius* Hong, 2002a, Fushun amber, Guchengzi, Liaoning Province, China.

F. Evaniidae (Cretevaniidae) K1(Hauterivian)-Holocene

First: e.g. *Lebanevia azari* Basibuyuk et al., 2002, Jezzine amber, Jouar Ess-Souss, Mouhafazet Loubnan El-Janoubi, Lebanon.

F. Expansicornidae Hong, 2002a(Expansicornrdae) Eoc.(Ypresian)

First and Last: *Expansicornia conulata* Hong, 2002a, Fushun amber, Guchengzi, Liaoning Province, China.

F. Falsiformicidae (Falciformicidae) K1(Barremian)-K2(Cenomanian)

First: Mentioned in Rasnitsyn (2002i), Lebanese amber, unknown horizon, unknown locality, Lebanon.

Last: e.g. *Falsiformica cretacea* in Ross and Jarzembowski (1993), Agapa amber, Dolganian Formation, Nizhnyaya Agapa River, West Taimyr Peninsula, Siberian Federal District, Russian Federation.

F. Figitidae (Charipidae, Eucoilidae, Palaeocynipidae, Rasnicynipidae, 'Rasnitsynidae') K2(Turonian)-Holocene

First: e.g. *Syneucoila magnifica* Liu & Engel *in* Liu et al., 2007b, New Jersey amber, South Amboy Fire Clay (Raritan Formation), New Jersey, United States.

F. Formicidae (Dolichoderidae, Megapteritidae, Paleosminthuridae, Sphecomyrmidae) K1(Aptian)-Holocene

First: *Cariridris bipetiolata* in Osten (2007), Crato Formation, Araripe Basin, Ceará, Brazil.

F. Fushunochrysidae Hong, 2002b Eoc.(Ypresian)

First and Last: *Fushunochrysites eocenicus* Hong, 2002b, Fushun amber, Guchengzi, Liaoning Province, China.

F. Gallorommatidae Gibson et al., 2007 K1(Albian)-K2(Cenomanian)

First: e.g. *Galloromma kachinensis* Engel and Grimaldi, 2007c, Burmese amber (Burmite), Hukawng Valley, Kachin State, Myanmar.

Last: *Galloromma agapa* in Gibson et al. (2007), Agapa amber, Dolganian Formation, Nizhnyaya Agapa River, West Taimyr Peninsula, Siberian Federal District, Russian Federation. (Formerly *Palaeomymar apaga*, placed in Mymarommatidae.)

F. Gasteruptiidae (Aulacidae, Baissidae, Kotujellidae, Manlayidae) K1(Berriasian)-Holocene

First: e.g. *Manlaya anglica* in Zhang and Rasnitsyn (2004), Lulworth Formation, Durlston Bay, Dorset, United Kingdom.

F. Gerocynipidae Liu & Engel *in* Liu et al., 2007b K2(Cenomanian)

e.g. *Gerocynips sibirica* in Liu et al. (2007b), Ola Formation, Obeshchayushchii Creek, Madagan Region, Russian Federation.

F. Halictidae (Rhophitidae) Eoc.(Ypresian)-Holocene
Cretaceous trace fossils previously attributed to Halictidae can not be placed so precisely to family, according to Engel and Archibald (2003).

First: *Halictus*? *savenyei* Engel and Archibald, 2003, coldwater beds of the Kamloops Group, Quilchena, British Columbia, Canada.

F. Heloridae J2(Callovian)-Holocene

First: Mentioned in Rasnitsyn and Zhang (2004a), Jiulongshan Formation, near Daohugou, Ningcheng county, Inner Mongolia, China.

F. Ibaliidae Eoc.(Priabonian)-Holocene

First: *Protoibalia connexiva* in Liu et al. (2007b), Florissant Formation, Florissant, Colorado, United States.

F. Ichneumonidae K1(Valanginian)-Holocene

First: e.g. *Palaeoichneumon freja* Kopylov, 2009, Zaza Formation, Baissa, Buryatia, Russian Federation.

F. Jurapriidae J3(Oxfordian)-K2(Turonian)

First: *Jurapria sibirica* in Rasnitsyn and Brothers (2007), Uda Formation, Uda River, Buryatia, Russian Federation.

Last: *Chalscelio orapa* Rasnitsyn and Brothers, 2007, Orapa diamond mines, Orapa, Orapa, Botswana.

F. Karatavitidae J1(Toarcian)-J3(Oxfordian)

First: *Grimmaratavites mirabilis* Rasnitsyn et al., 2006a, Upper Lias, Grimmen, Mecklenburg-Vorpommern, Germany.

Last: e.g. *Karatavites angustus* in Carpenter (1992b), Karabastau Formation, Karatau Range, Tien Shan mountains, Kazakhstan.

F. Khutelchalcididae Rasnitsyn et al., 2004b K1(Berriasian)

First and Last: *Khutelchalcis gobiensis* Rasnitsyn et al., 2004b, Tsagan-Tsab, Khutel-Kara, Dornogovi (East Gobi) Aimag, Mongolia.

F. Leucospidae Mio.(Burdigalian)-Holocene

First: *Leucospis glaesaria* in Arillo and Ortuño (2005), Dominican amber, Cordillera Septentrional, near Santiago, Dominican Republic.

F. 'Limnetidae' Hong, 1983 J2(Callovian)
This family name is a junior homonym of the conchostracan family Limnetidae (itself a junior subjective synonym of Lynceidae) mentioned in Simon (1886).

First and Last: *Limnetus wangyingziensis* Hong, 1983, Jiulongshan Formation, near Daohugou, Ningcheng county, Inner Mongolia, China.

F. Liopteridae K2(Campanian)-Holocene

First: e.g. *Proliopteron redactus* Liu & Engel *in* Liu et al., 2007b, Canadian amber, Medicine Hat, Alberta, Canada.

F. Maimetshidae (Maimetsheidae) K1(Barremian)-K2(Santonian)

First: *Andyrossia joyceae* in Rasnitsyn and Brothers (2009), Upper Weald Clay Formation, Capel, Surrey, United Kingdom.

Last: *Maimetsha artica* in Rasnitsyn and Brothers (2009), Yantardakh amber, Kheta Formation, Taimyr, Krasnoyarsk Krai, Siberian Federal District, Russian Federation.

F. Megachilidae Pal.(Thanetian)-Holocene

First: *Probombus hirsutus* in Michez et al. (2009), spongo-diatomaceous maar, Menat, Puy-de-Dôme, Auvergne, France.

F. Megalodontesidae (Megalodontidae) K1(Aptian)-Holocene

First: *Jibaissodes giganteus* in Blank et al. (2009), Yixian Formation, Chengde, Hebei Province, China.

F. Megalyridae (Megaliridae) K1(Albian)-Holocene

First: e.g. *Valaa delclosi* Perrichot, 2009, Álava amber, Escucha Formation, Basco-Cantabrian Basin, Álava Province, Spain.

F. Megaspilidae K1(Albian)-Holocene

First: Mentioned in Grimaldi et al. (2002), Burmese amber (Burmite), Hukawng Valley, Kachin State, Myanmar.

F. Melittidae Eoc.(Ypresian)-Holocene

First: *Palaeomacropis eocenicus* Michez & Nel *in* Michez et al., 2007, Oise amber, Le Quesnoy, Houdancourt, Oise, Picardie, France.

F. Melittosphecidae Poinar and Danforth, 2006 K1(Albian)

First and Last: *Melittosphex burmensis* in Poinar (2009b), Burmese amber (Burmite), Hukawng Valley, Kachin State, Myanmar.

F. Mesoserphidae J2(Callovian)-K1(Aptian)
Rasnitsyn (2002i) lists this family as occurring in the Lower Jurassic but does not provide any supporting information.

First: e.g. *Karatoserphus* sp. in Rasnitsyn and Zhang (2004a), Jiulongshan Formation, near Daohugou, Ningcheng county, Inner Mongolia, China.

Last: Figured in Osten (2007), Crato Formation, Araripe Basin, Ceará, Brazil.

F. Monomachidae K1(Aptian)-Holocene

First: Mentioned in Rasnitsyn and Martínez-Delclòs (2000), Koonwarra Fossil Bed (Korumburra Group), South Gippsland, Victoria, Australia.

F. Mutillidae (Cretavidae) K2(Campanian)-Holocene
Brothers (2003) prefers not to include *Cretavus sibiricus* and several other fossils from this family, which would leave the earliest records as from the Priabonian Baltic amber.

First: *Cretavus sibiricus* in Manley and Poinar (2003), Kass suite, Krasnoyarsk Krai, Siberian Federal District, Russian Federation.

F. Mymaridae K1(Barremian)-Holocene
The Jordanian amber record figured in Kaddumi (2005) is doubtful.

First: Mentioned in Poinar and Milki (2001), Lebanese amber, unknown horizon, unknown locality, Lebanon.

F. Mymarommatidae K1(Albian)-Holocene

First: e.g. Mentioned in Delclòs et al. (2007), Álava amber, Escucha Formation, Basco-Cantabrian Basin, Álava Province, Spain.

F. Ormyridae K2(Campanian)-Holocene

First: Mentioned in Gumovsky (2001), Canadian amber, Grassy Lake, Alberta, Canada. (McKellar et al. 2008 do not list this family in Canadian amber.)

F. Orussidae K1(Albian)-Holocene

First: Mentioned in Delclòs et al. (2007), Álava amber, Escucha Formation, Basco-Cantabrian Basin, Álava Province, Spain.

F. Paleomelittidae Engel, 2001 Eoc.(Priabonian)

First and Last: *Paleomelitta nigripennis* Engel, 2001, Baltic amber.

F. Pamphiliidae (Pamphilidae) J2(Callovian)-Holocene
*Mesolyda* (*Pesarinia*) *rara* from the Middle Jurassic Jiulongshan Formation (Liaoning), China, more likely belongs in either Siricidae or Sepulcidae according to Blank et al. (2009).

First: Mentioned in Rasnitsyn and Zhang (2004a), Jiulongshan Formation, near Daohugou, Ningcheng county, Inner Mongolia, China. (These specimens are not named as *Mesolyda rara*, so are unaffected by the comment above.)

F. Paroryssidae (Parorysidae) J3(Oxfordian)
The specimen figured by Rasnitsyn and Zhang (2004a) as Paroryssidae gen. et sp. nov. from the Callovian Daohugou beds was later described as *Praeparyssites orientalis* in Karatavitidae by Rasnitsyn et al. (2006a).

e.g. *Microryssus antennatus* in Vilhelmsen (2004), Karabastau Formation, Karatau Range, Tien Shan mountains, Kazakhstan.

F. Paxylommatidae K2(Campanian)-Holocene

First: Mentioned in McKellar et al. (2008), Canadian amber, Grassy Lake, Alberta, Canada.

F. Pelecinidae (Iscopinidae, Pelecinopteridae) J2(Callovian)-Holocene

First: e.g. *Archaeopelecinus tebbei* Shih et al., 2009, Jiulongshan Formation, near Daohugou, Ningcheng county, Inner Mongolia, China.

F. Peradeniidae Naumann and Masner, 1985 Eoc.(Priabonian)-Holocene

First: *Peradenia galerita* Johnson et al., 2001, Baltic amber.

F. Perilampidae Eoc.(Priabonian)-Holocene
Putative Perliampidae described by Hong (2002a) in Fushun amber are suspect in their placement and require further study, according to Heraty and Darling (2009).

First: e.g. *Perilampus pisticus* Heraty and Darling, 2009, Baltic amber.

F. Platygastridae K2(Turonian)-Holocene

First: Mentioned in Rasnitsyn (2000b), New Jersey amber, South Amboy Fire Clay (Raritan Formation), New Jersey, United States.

F. Pompilidae K1(Albian)-Holocene

*Pompilopterus ciliatus* from the Lower Cretaceous Zaza Formation is an angarosphecid (Rasnitsyn et al., 1998; Engel and Grimaldi, 2006c).

First: *Bryopompilus interfector* Engel and Grimaldi, 2006c, Burmese amber (Burmite), Hukawng Valley, Kachin State, Myanmar.

F. Praeaulacidae (Anomopterellidae) J2(Callovian)-K1(Aptian)

First: e.g. *Praeaulacus daohugouensis* Zhang and Rasnitsyn, 2008, Jiulongshan Formation, near Daohugou, Ningcheng county, Inner Mongolia, China.

Last: e.g. *Wesratia nana* in Zhang and Rasnitsyn (2008), Koonwarra Fossil Bed (Korumburra Group), South Gippsland, Victoria, Australia.

F. Praeichneumonidae K1(Berriasian)-K1(Aptian)

First: *Praeichneumon townesi* in Carpenter (1992b), Tsagan-Tsab, Khutel-Kara, Dornogovi (East Gobi) Aimag, Mongolia.

Last: *Scolichneumon rectivenius* in Ren (2002b), Yixian Formation, Chengde, Hebei Province, China.

F. Praesiricidae J3(Oxfordian)-K1(Aptian)

First: *Aulidontes mandibulatus* in Carpenter (1992b), Karabastau Formation, Karatau Range, Tien Shan mountains, Kazakhstan.

Last: *Sinosepulca gigathoracalis* in Blank et al. (2009), Dawangzhangzi beds, Yixian Formation, Liaoning Province, China.

F. Proctotrupidae (Proctitrupidae, Serphidae) K1(Berriasian)-Holocene

First: e.g. *Pallenites calcarius* Rasnitsyn & Jarzembowski *in* Rasnitsyn et al., 1998, Lulworth Formation, Durlston Bay, Dorset, United Kingdom.

F. Protimaspidae Liu & Engel *in* Liu et al., 2007b K2(Campanian)

First and Last: *Protimaspis costalis* in Liu et al. (2007b), Canadian amber, Cedar Lake, Manitoba, Canada.

F. Protosiricidae Rasnitsyn and Zhang, 2004a J1(Toarcian)-J3(Oxfordian)

First: *Liasirex sogdianus* in Sukatsheva and Rasnitsyn (2004), Sagul Formation, Sai-Sagul, Batkenskii District, Kyrgyzstan. (Family placement after Rasnitsyn and Zhang 2004a.)

Last: e.g. *Protosirex xyelopterus* in Rasnitsyn (2006), Karabastau Formation, Karatau Range, Tien Shan mountains, Kazakhstan.

F. Pteromalidae (Cleonymidae) Eoc.(Priabonian)-Holocene
The fossils described as *Eopteromalites fushunensis*, *Leptogasterites brunneus* and *L. furvus* by Hong (2002a) belong in Scelionidae according to Johnson et al. (2008). The Siberian amber record in Poinar (1992) is erroneous.

First: e.g. Figured in Weitschat and Wichard (2002), Baltic amber.

F. Rhopalosomatidae K1(Albian)-Holocene
Engel (2008c) considers *Mesorhopalosoma cearae* from the Aptian Crato Formation (Brazil) not to show characters sufficient for a placement in Rhopalosomatidae but may represent a stem-group to this family. Osten (2007) considers it to belong to Angarosphecidae.

First: *Eorhopalosoma gorgyra* Engel, 2008c, Burmese amber (Burmite), Hukawng Valley, Kachin State, Myanmar.

F. Roproniidae (Beipiaosiricidae) J2(Callovian)-Holocene

First: e.g. *Beipiaosirex parva* in Blank et al. (2009), Haifanggou Formation, Beipiao, Liaoning Province, China.

F. Sapygidae K1(Barremian)-Holocene

First: Mentioned in Peñalver et al. (1999), Montsec lithographic limestones, Montsec Range, Lleida Province, Spain. (Neither Bennett and Engel 2005 or Osten 2007 mention this occurrence.)

F. Scelionidae K1(Valanginian)-Holocene

First: Figured in Rasnitsyn (2002i), Zaza Formation, Baissa, Buryatia, Russian Federation.

F. Sclerogibbidae K1(Barremian)-Holocene

First: *Sclerogibbodes embioleia* Engel and Grimaldi, 2006b, Lebanese amber, unknown horizon, unknown locality, Lebanon.

F. Scolebythidae K1(Barremian)-Holocene

First: e.g. *Uliobythus terpsichore* Engel and Grimaldi, 2007a, Hammana/Mdeyrij amber, Caza Baabda, Mouhafazet Jabal Loubnan, Lebanon.

F. Scoliidae (Scolidae) K1(Barremian)-Holocene

First: e.g. *Cretoscolia conquensis* Rasnitsyn and Martínez-Delclòs, 2000, Calizas de la Huérguina Formation, Las Hoyas, Cuénca Province, Spain.

F. Sepulcidae (Parapamphiliidae) J1(Sinemurian)-K2(Cenomanian)

First: *Sogutia liassica* in Rasnitsyn et al. (2003), Dzhil Formation, Sogyuty, Issyk-Kul, Kyrgyzstan.

Last: *Prosyntexis okhotensis* in Rasnitsyn et al. (1998), Ola Formation, Obeshchayushchii Creek, Madagan Region, Russian Federation.

F. Serphitidae K1(Albian)-K2(Campanian)

First: e.g. *Serphites* sp. in Rasnitsyn (2002i), Burmese amber (Burmite), Hukawng Valley, Kachin State, Myanmar.

Last: e.g. *Serphites doxus* in McKellar et al. (2008), Canadian amber, Cedar Lake, Manitoba, Canada.

F. Sierolomorphidae K1(Albian)-Holocene

First: Mentioned in Poinar and Poinar (2008), Burmese amber (Burmite), Hukawng Valley, Kachin State, Myanmar.

F. Signiphoridae Eoc.(Priabonian)-Holocene

First: Mentioned in Perkovsky et al. (2003), Rovno amber, Klesov/Dubrovitsa, Rivne Oblast, Ukraine.

F. Siricidae (Gigasiricidae, Myrmiciidae, Pararchexyelidae, Pseudosiricidae, Sinosiricidae) J2(Callovian)-Holocene
Previous reports of this family in the Lower Jurassic of Kyrgyzstan were erroneous (Rasnitsyn and Zhang, 2004a).

First: e.g. *Gigasirex* spp. in Rasnitsyn and Zhang (2004a), Jiulongshan Formation, near Daohugou, Ningcheng county, Inner Mongolia, China.

F. Sphecidae Eoc.(Priabonian)-Holocene
Grimaldi and Engel (2005) state that the first definitive sphecids are not known until the mid-Cretaceous, however Bennett and Engel (2006) subsequently move all the amber sphecids to Ampulicidae, Crabronidae or incertae sedis. See also the Catalog of Sphecidae (`http://research.calacademy.org/ent/catalog_sphecidae`).

First: e.g. *Ammophila antiquella* in Meyer (2003), Florissant Formation, Florissant, Colorado, United States.

F. Stephanidae K2(Turonian)-Holocene
*Chosia yamadai* Fujiyama, 1994 is not a stephanid (see Engel and Grimaldi, 2004a).

First: *Archaeostephanus corae* Engel and Grimaldi, 2004a, New Jersey amber, South Amboy Fire Clay (Raritan Formation), New Jersey, United States.

F. Stigmaphronidae K1(Valanginian)-K2(Campanian)

First: *Aphrostigmon vitimense* in Engel and Grimaldi (2009), Zaza Formation, Baissa, Buryatia, Russian Federation.

Last: *Tagsmiphron canadense* Engel and Grimaldi, 2009, Canadian amber, Cedar Lake, Manitoba, Canada.

F. Stolamissidae Liu & Engel *in* Liu et al., 2007b K2(Turonian)

First and Last: *Stolamissus mirabilis* Liu & Engel *in* Liu et al., 2007b, New Jersey amber, South Amboy Fire Clay (Raritan Formation), New Jersey, United States.

F. Tanaostigmatidae Eoc.(Priabonian)-Holocene

First: *Leptoomus janzeni* Gibson, 2008, Baltic amber.

F. Tenthredinidae K1(Barremian)-Holocene

First: *Palaeathalia laiyangensis* in Nyman et al. (2006), Laiyang Formation, Laiyang County, Shandong Province, China.

F. Tetracampidae K2(Campanian)-Holocene
Gumovsky and Perkovsky (2005) rejected other amber species, however this family may occur in Lebanese amber, see Heraty and Darling (2009).

First: e.g. *Baeomorpha distincta* in McKellar et al. (2008), Canadian amber, Cedar Lake, Manitoba, Canada.

F. Thysanidae Mio.(Aquitanian)-Holocene

First: Mentioned in Solórzano Kraemer (2007), Mexican amber, Simojovel, Chiapas, Mexico.

F. Tiphiidae (Methocidae, Tiphidae) K1(Aptian)-Holocene

First: *Architiphia rasnitsyni* in Engel et al. (2009b), Crato Formation, Araripe Basin, Ceará, Brazil.

F. Torymidae K2(Campanian)-Holocene

First: Mentioned in McKellar et al. (2008), Canadian amber, Grassy Lake, Alberta, Canada.

F. Trichogrammatidae Eoc.(Priabonian)-Holocene
Huber (2005) transferred the Canadian amber *Enneagmus pristinus* to Mymaridae. McKellar et al. (2008) appear not to have seen this and list it in Trichogrammatidae, citing only the original description by Yoshimoto (1975).

First: Mentioned in Perkovsky et al. (2007), Rovno amber, Klesov/Dubrovitsa, Rivne Oblast, Ukraine.

F. Trigonalidae K1(Albian)-Holocene
Nel et al. (2003b) remove all previously described Lower Cretaceous species from this family.

First: *Albiogonalys elongatus* Nel et al., 2003b, Archingeay amber, Archingeay-Les Nouillers, Charente-Maritime, France.

F. Vespidae (Eumenidae, Masaridae, Vespoidae) K1(Valanginian)-Holocene

First: e.g. *Curiosivespa antiqua* in Brothers and Rasnitsyn (2008), Zaza Formation, Baissa, Buryatia, Russian Federation.

F. Xyelidae T3(Carnian)-Holocene

First: e.g. *Archexyela ipswichensis* Engel, 2005b, Mount Crosby Formation, Ipswich Basin, Queensland, Australia.

F. Xyelotomidae J1(Toarcian)-K1(Aptian)
Nel et al. (2004c) consider this family to likely be paraphyletic.

First: *Pseudoxyelocerus bascharagensis* Nel et al., 2004c, Upper Lias, Bascharage and Sanem, Luxembourg district, Luxembourg.

Last: e.g. *Synaptotoma limi* Gao et al., 2009, Dawangzhangzi beds, Yixian Formation, Liaoning Province, China.

F. Xyelydidae (Xyelididae) J1(Toarcian)-K1(Aptian)

First: e.g. *Sagulyda arcuata* in Rasnitsyn et al. (2006b), Sagul Formation, Sai-Sagul, Batkenskii District, Kyrgyzstan.

Last: *Sinoprolyda meileyingensis* in Ross and Jarzembowski (1993), Jiufotang Formation, Beishan, Yixian County, Liaoning Province, China. (Rasnitsyn et al. 2006b do not mention this species.)

**O. Lepidoptera** Linnaeus, 1758 (Papilionida) Jurassic(Sinemurian)-Quaternary(Holocene)

*Scythropites balticella* was listed under Plutellidae by Kozlov (1988), so the family Scythriidae (Scythridae, Scythrididae) does not have a fossil record.

F. Acrolophidae Mio.(Burdigalian)-Holocene

First: e.g. *Acrolophus* sp. in Peñalver and Grimaldi (2006), Dominican amber, Cordillera Septentrional, near Santiago, Dominican Republic.

F. Adelidae Eoc.(Priabonian)-Holocene

First: e.g. *Adela kuznetzovi* in Fernández-Rubio (1999), Baltic amber.

F. Archaeolepidae J1(Sinemurian)

First and Last: *Archaeolepis mane* in de Jong (2007), Black Ven Marls, Charmouth, Dorset, United Kingdom.

F. Blastobasidae Mio.(Burdigalian)-Holocene

First: Mentioned in Peñalver and Grimaldi (2006), Dominican amber, Cordillera Septentrional, near Santiago, Dominican Republic.

F. Bucculatricidae K2(Turonian)-Holocene

First: *Bucculatrix platani* in Lopez-Vaamonde et al. (2006), Kzyl-Zhar, Karatau Range, Tien Shan mountains, Kazakhstan.

F. Castniidae Eoc.(Priabonian)-Holocene

First: *Dominickus castnioides* in de Jong (2007), Florissant Formation, Florissant, Colorado, United States. (de Jong 2007 expresses some doubt about the placement of this fossil.)

F. Coleophoridae (Coelophoridae) K2(Turonian)-Holocene

First: Figured (ichnofossil) in Krassilov (2007), Ora Formation, Arava Valley, Negev, Israel.

F. Copromorphidae Eoc.(Priabonian)-Holocene

First: e.g. *Copromorpha fossilis* in Fernández-Rubio (1999), Bembridge Marls Insect Limestone, Gurnard/Thorness Bay, Isle of Wight, United Kingdom.

F. Cosmopterigidae (Cosmopterygidae, Walshiidae) Mio.(Aquitanian)-Holocene

First: Mentioned in Grimaldi and Engel (2005), Mexican amber, Simojovel, Chiapas, Mexico.

F. Cossidae Eoc.(Priabonian)-Holocene

First: e.g. *Gurnetia durranti* in Fernández-Rubio (1999), Bembridge Marls Insect Limestone, Gurnard/Thorness Bay, Isle of Wight, United Kingdom.

F. Elachistidae (Ethmiidae, Stenomidae) Eoc.(Ypresian)-Holocene

First: *Hexerites primalis* in Skalski (1992), Green River Formation, Colorado, United States.

F. Eolepidopterigidae J3(Oxfordian)-K1(Aptian)

First: e.g. *Eolepidopteryx jurassica* in Kozlov et al. (2002), Uda Formation, Uda River, Buryatia, Russian Federation.

Last: *Xena nana* in Bechly (2007a), Crato Formation, Araripe Basin, Ceará, Brazil.

F. Eriocraniidae Mio.(Tortonian)-Holocene
There is no body-fossil record of this family as '*Dyseriocrania*' *perveta* (Burmese amber) belongs in *Sabatinca* (Ross and York, 2000) and '*Electrocrania*' *immensipalpa* (Baltic amber) belongs in *Micropterix* (Kozlov, 1988) (both Micropterigidae).

First: Mentioned (mines) in Grimaldi and Engel (2005), Payette Formation, Thorn Creek, Idaho, United States. (The mention of leaf mines from the Bembridge Marls, UK by Grimaldi and Engel 2005 is erroneous.)

F. Gelechiidae K1(Albian)-Holocene

First: Mentioned in Poinar and Poinar (2008), Burmese amber (Burmite), Hukawng Valley, Kachin State, Myanmar.

F. Geometridae K2(Turonian)-Holocene

First: Figured in Harris and Raine (2002), Monro Conglomerate, Rakaia Gorge, Canterbury, New Zealand.

F. Gracillariidae (Phyllocnistidae) K1(Albian)-Holocene

First: Mentioned in Poinar and Poinar (2008), Burmese amber (Burmite), Hukawng Valley, Kachin State, Myanmar.

F. Heliodinidae Eoc.(Priabonian)-Holocene

First: *Baltonides roeselliformis* in Fernández-Rubio (1999), Baltic amber.

F. Heliozelidae Eoc.(Ypresian)-Holocene

First: Mentioned (mines) in Grimaldi and Engel (2005), Klondike Mountain Formation, Okanagan Highlands, Washington, United States.

F. Hepialidae Pal.(Thanetian)-Holocene

First: *Prohepialus incertus* in Fernández-Rubio (1999), spongo-diatomaceous maar, Menat, Puy-de-Dôme, Auvergne, France.

F. Hesperiidae Eoc.(Ypresian)-Holocene

First: Mentioned in Kristensen and Skalski (1999), Fur Formation (Mo Clay), Limfjord/Mors Peninsula/Fur Island, Jutland, Denmark.

F. Incurvariidae K1(Barremian)-Holocene

First: *Incurvarites* sp. in Poinar and Milki (2001), Lebanese amber, unknown horizon, unknown locality, Lebanon.

F. Lophocoronidae (Lophiocoronidae) K2(Santonian)-Holocene

First: Mentioned in Grimaldi (1999), Yantardakh amber, Kheta Formation, Taimyr, Krasnoyarsk Krai, Siberian Federal District, Russian Federation. (Doubt exists as to the placement of this fossil according to Grimaldi 1999.)

F. Lycaenidae Mio.(Aquitanian)-Holocene
*Riodinella nympha* (Green River Formation) and *Lithopsyche antiqua* (Bembridge Marls Insect Limestone) do not belong in this family but are unplaced within Rhopalocera (Hall et al., 2004).

First: *Aquisextana irenaei* in Braby et al. (2005), Gypse d'Aix, Aix-Basin, Provence, France.

F. Lyonetiidae (Prolyonetiidae) Eoc.(Priabonian)-Holocene

First: *Prolyonetia cockerelli* in Fernández-Rubio (1999), Baltic amber.

F. Micropterigidae (Micropterygidae) J3(Oxfordian)-Holocene

First: *Auliepterix mirabilis* in Kozlov et al. (2002), Karabastau Formation, Karatau Range, Tien Shan mountains, Kazakhstan.

F. Mnesarchaeidae K2(Santonian)-Holocene

First: Mentioned in Kristensen and Skalski (1999), Yantardakh amber, Kheta Formation, Taimyr, Krasnoyarsk Krai, Siberian Federal District, Russian Federation.

F. Nepticulidae K2(Cenomanian)-Holocene
Jurassic trace fossil records are doubtful.

First: Mentioned (mines) in Grimaldi and Engel (2005), Dakota Formation, Rose Creek, Kansas, United States.

F. Noctuidae (Arctiidae, Ctenuchidae, Lymantriidae, Syntomidae) Olig.(Chattian)-Holocene
Placement of the fossil egg from the Campanian Magothy Formation, Massachusetts (Gáll and Tiffney, 1983) in Noctuoidea is highly doubtful (Kristensen and Skalski, 1999; Kozlov et al., 2002).

First: *Philodarchia cigana* in Grimaldi and Engel (2005), Tremembé Formation, Taubaté Basin, São Paulo, Brazil.

F. Notodontidae Mio.(Aquitanian)-Holocene

First: Mentioned in Kvaček et al. (2004), Most Formation, Bílina, Bohemia, Czech Republic.

F. Nymphalidae (Danaidae, Libytheidae, Satyridae) Eoc.(Ypresian)-Holocene

First: Mentioned in Peñalver and Grimaldi (2006), Green River Formation, Unitas area, Colorado, United States.

F. Oecophoridae Eoc.(Ypresian)-Holocene

First: e.g. Mentioned in Brasero et al. (2009), Oise amber, Le Quesnoy, Houdancourt, Oise, Picardie, France.

F. Papilionidae Eoc.(Ypresian)-Holocene

First: e.g. *Praepapilio colorado* in de Jong (2007), Green River Formation, Unitas area, Colorado, United States.

F. Pieridae Eoc.(Priabonian)-Holocene

First: *Stolopsyche libytheoides* in de Jong (2007), Florissant Formation, Florissant, Colorado, United States.

F. Plutellidae (Plutelidae) Eoc.(Priabonian)-Holocene

First: e.g. *Epinomeuta truncatipennella* in Fernández-Rubio (1999), Baltic amber.

F. Psychidae Eoc.(Priabonian)-Holocene

First: e.g. *Palaeopsyche secundum* Sobczyk and Kobbert, 2009, Baltic amber.

F. Pterophoridae Mio.(Aquitanian)-Holocene

First: *Pterophorus oligocenicus* in Fernández-Rubio (1999), Gypse d'Aix, Aix-Basin, Provence, France.

F. Pyralidae (Pyralididae) Eoc.(Ypresian)-Holocene
Possible earlier records of this family come from feeding traces from the Klondike Mountain Formation (Labandeira, 2002).

First: Mentioned in Bonde et al. (2008), Fur Formation (Mo Clay), Limfjord/Mors Peninsula/Fur Island, Jutland, Denmark.

F. Riodinidae Mio.(Burdigalian)-Holocene

First: e.g. *Voltina dramba* in Peñalver and Grimaldi (2006), Dominican amber, Cordillera Septentrional, near Santiago, Dominican Republic.

F. Saturniidae Eoc.(Priabonian)-Holocene

First: *Attacus*? *fossilis* in Meyer (2003), Florissant Formation, Florissant, Colorado, United States.

F. Sesiidae (Aegeriidae) Eoc.(Priabonian)-Holocene

First: Mentioned in Weitschat and Wichard (2002), Baltic amber.

F. Sphingidae Eoc.(Priabonian)-Holocene

First: Mentioned in Weitschat and Wichard (2002), Baltic amber.

F. Symmocidae Eoc.(Priabonian)-Holocene

First: e.g. *Oegoconiites borisjaki* in Poinar (1992), Baltic amber.

F. Thyrididae Eoc.(Priabonian)-Holocene

First: Mentioned in Kristensen and Skalski (1999), Baltic amber.

F. Tineidae Eoc.(Ypresian)-Holocene

First: Mentioned in Brasero et al. (2009), Oise amber, Le Quesnoy, Houdancourt, Oise, Picardie, France.

F. Tortricidae Eoc.(Priabonian)-Holocene

First: e.g. *Tortricites skalskii* in Zherikhin (2002c), Baltic amber.

F. Undopterigidae (Undopterygidae) J3(Tithonian)-K1(Aptian)

First: *Undopterix sukatshevae* in Grimaldi and Engel (2005), Glushkovo Formation, Unda, Transbaikalia, Russian Federation.

Last: *Undopterix caririensis* in Bechly (2007a), Crato Formation, Araripe Basin, Ceará, Brazil.

F. Yponomeutidae (Argyresthiidae) Eoc.(Priabonian)-Holocene

First: Mentioned in Weitschat and Wichard (2002), Baltic amber.

F. Zygaenidae Olig.(Rupelian)-Holocene

First: *Neurosymploca*? *oligocenica* Fernández-Rubio and Nel, 2000, Céreste, Lubéron, Alpes-de-Haute-Provence, France.

**O. Mecoptera** Packard, 1886 (Mecaptera, Nannomecoptera, Panorpida, Paramecoptera, Paratrichoptera) Carboniferous(Bashkirian)-Quaternary(Holocene)

Taxonomic system generally follows Novokshonov (2002a). Any differences are noted in the text. Englathaumatidae, mentioned in Novokshonov (2002a), is a *nomen nudum* as the description has not yet been published.

F. Aneuretopsychidae Rasnitsyn and Kozlov, 1990(Aneuropsychidae) J3(Oxfordian)-K1(Barremian)

First: e.g. *Aneuretopsyche rostrata* in Labandeira et al. (2007), Karabastau Formation, Karatau Range, Tien Shan mountains, Kazakhstan.

Last: *Jeholopsyche liaoningensis* Ren, Shih & Labandeira *in* Ren et al., 2009, Yixian Formation, Huangbanjiguo Village, Beipiao, Liaoning Province, China.

F. Anormochoristidae P1(Artinskian)

First and Last: *Anormochorista oligoclada* in Novokshonov (2004), Wellington Formation, Elmo site, Dickinson County, Kansas, United States.

F. Archipanorpidae T3(Carnian)

First and Last: *Archipanorpa magnifica* in Jell (2004), Blackstone Formation, Ipswich Basin, Queensland, Australia.

F. Austropanorpidae (Austropanorpodidae) Pal.(Thanetian)
Novokshonov (2002a) tentatively places this family within Orthophlebiidae but Archibald (2005) mentions it as a separate family.

First and Last: *Austropanorpa australis* in Jell (2004), Redbank Plains Formation, Ipswich Basin, Queensland, Australia. (Jell 2004 lists this species in Panorpidae.)

F. Belmontiidae (Parabelmontiidae) P3(Changhsingian)

e.g. *Belmontia mitchelli* in Jell (2004), Belmont insect beds, Newcastle Coal Measures, Belmont/Warner's Bay, New South Wales, Australia.

F. Bittacidae J2(Callovian)-Holocene
Without the inclusion of Neorthophlebiidae, Bittacidae does not range down into the Upper Triassic as is often reported (e.g. Novokshonov, 2002a; Krzemiński, 2007).

First: e.g. *Formosibittacus macularis* Li et al., 2008, Jiulongshan Formation, near Daohugou, Ningcheng county, Inner Mongolia, China.

F. Boreidae J3(Tithonian)-Holocene

First: *Palaeoboreus zherichini* in Grimaldi and Engel (2005), Ulan-Ereg, Khoutiyn-Khotgor, Dund-Gobi Aimag, Mongolia.

F. Choristidae K1(Aptian)-Holocene

First: *Cretacochorista parva* in Jell (2004), Koonwarra Fossil Bed (Korumburra Group), South Gippsland, Victoria, Australia.

F. Cimbrophlebiidae J1(Toarcian)-Eoc.(Ypresian)
Novokshonov (2002a) considered this to be a junior synonym of Bittacidae, however Archibald (2009) maintains it as a sister group.

First: Mentioned in Archibald (2009), Upper Lias, Grimmen, Mecklenburg-Vorpommern, Germany.

Last: e.g. *Cimbrophlebia brooksi* Archibald, 2009, Klondike Mountain Formation, Okanagan Highlands, Washington, United States.

F. Dinopanorpidae Pal.(Thanetian)-Olig.(Rupelian)

First: *Dinopanorpa* sp. in Archibald (2005), Tadushi Formation, Sikhote Alin Range, Primorye, Russian Federation.

Last: *Dinopanorpa megarche* in Archibald (2005), Khutsin Formation, Amgu (Amagu), Terney District, Primorye, Russian Federation.

F. Eomeropidae (Eomeropeidae, Notiothaumidae) J2(Callovian)-Holocene
The Triassic families formerly placed here are now considered to form the separate family Thaumatomeropidae (Novokshonov, 2002a; Archibald et al., 2005).

First: *Tsuchingothauma shihi* Ren and Shih, 2005, Jiulongshan Formation, near Daohugou, Ningcheng county, Inner Mongolia, China.

F. Holcorpidae Eoc.(Priabonian)

First and Last: *Holcorpa maculosa* in Grimaldi and Engel (2005), Florissant Formation, Florissant, Colorado, United States.

F. Kaltanidae (Cyclopteridae, Cyclopterinidae, Cycloristidae, Cycochoristidae) C2(Gzhelian)-P3(Changhsingian)

First: e.g. Figured in Rasnitsyn et al. (2004a), Bursum Formation (Red Tanks Member), Carrizo Arroyo, New Mexico, United States. (These specimens may belong to a new family rather than Kaltanidae according to Rasnitsyn et al. 2004a, however Ren et al. 2009 [supporting online material] accept their placement here.)

Last: e.g. *Pinnachorista problematica* Novokshonov, 1994c, Maichat/Ak-Kolka Formation, Karaungir River, Saur Mountains, Vostochno-Kazakhstanskaya oblast, Kazakhstan.

F. Liassophilidae (Laurentipteridae, Pseudodipteridae) T2(Anisian)-J2(Aalenian)

First: *Laurentiptera gallica* in Krzemiński and Krzemińska (2003), Grès à Voltzia, Bas-Rhin/Moselle, Northern Vosges Mountains, France.

Last: e.g. *Ijapsyche sibirica* in Novokshonov (2002a), Cheremkhora Formation, Iya River, Irkutsk Region, Siberian Federal District, Russian Federation.

F. Meropeidae T2(Ladinian)-Holocene

First: *Sinothauma ladinica* Hong and Li, 2007, Tongchuan Formation, Hejiafang, Tongchuan District, Shaanxi Province, China. (This record is doubtful.)

F. Mesopanorpodidae P3(Wuchiapingian)-K1(Aptian)
Novokshonov (2002a) considered this a junior synonym of Permochoristidae but Hong (2007b) and Sun et al. (2007b) maintain it as a separate family.

First: e.g. *Prochoristella balgowanensis* van Dijk and Geertsema, 1999, Normandien (Estcourt) Formation, Beaufort Group, KwaZulu-Natal, Karoo Basin, South Africa.

Last: *Prochoristella leongatha* in Jell (2004), Koonwarra Fossil Bed (Korumburra Group), South Gippsland, Victoria, Australia.

F. Mesopsychidae T3(Carnian)-K1(Barremian)

First: e.g. *Mesopsyche triareolata* in Jell (2004), Blackstone Formation, Ipswich Basin, Queensland, Australia.

Last: *Vitimopsyche kozlovi* Ren, Labandeira & Shih *in* Ren et al., 2009, Yixian Formation, Shimen Village, Yangshulin Township, Hebei Province, China.

F. Muchoriidae Willmann, 1989(Munchoriidae) J2(Aalenian)

First and Last: *Muchoria reducta* in Willmann (1989), Ichetuy Formation, Novospasskoye, Mukhorshibirsky District, Buryatia, Russian Federation.

F. Nannochoristidae P3(Wuchiapingian)-Holocene
This family is treated as the separate order Nannomecoptera by Beutel and Baum (2008).

First: *Neochoristella goodalli* van Dijk and Geertsema, 1999, Normandien (Estcourt) Formation, Beaufort Group, KwaZulu-Natal, Karoo Basin, South Africa.

F. Neorthophlebiidae T2(Ladinian)-J3(Tithonian)
Novokshonov (2002a) considered this a junior synonym of Bittacidae but Hong (2009b) maintains it as a separate family. *Yanorthophlebia hebeiensis* from the Lower Cretaceous Yixian formation was tranferred to *Liassochorista* (Permochoristidae) by Novokshonov (1997b).

First: e.g. *Ctenophlebia tongchuanensis* Hong, 2009b, Tongchuan Formation, Hejiafang, Tongchuan District, Shaanxi Province, China.

Last: *Neorthophlebia yunnanensis* Zhang & Hong *in* Zhang et al., 2003, Tuodian Formation, Lufeng, Yunnan Province, China.

F. Orthophlebiidae T2(Ladinian)-K1(Aptian)
Hong and Zhang (2007) followed Carpenter (1992b) in only including three genera in this family. Thus *Choristopanorpa drinnani* and *Neoparachorista clarkae* from the Aptian Koonwarra Fossil Beds of Australia (see Jell, 2004) are not included. See also Willmann and Novokshonov (1998).

First: e.g. *Protorthophlebia* (*Psomophlebia*) *curta* Hong, 2009b, Tongchuan Formation, Hejiafang, Tongchuan District, Shaanxi Province, China.

Last: *Orthophlebia fangshanensis* in Hong and Zhang (2007), Lushangfen Formation, Xishan, Fangshan County of Beijing, China.

F. Panorpidae K1(Albian)-Holocene

First: *Solusipanorpa gibbdorsa* in Sun et al. (2007a), Chaochuan Formation, Zhuji, Zhejiang Province, China.

F. Panorpodidae Eoc.(Ypresian)-Holocene

First: *Austropanorpodes gennaken* Petrulevičius, 2009, Laguna del Hunco Formation, Tufolitas Laguna del Hunco, Chubut, Patagonia, Argentina. (Placement in this family is tentative.)

F. Parachoristidae (Choristopanorpidae, Neoparachoristidae, Triassochoristidae) P2(Roadian)-K1(Aptian)
*Parachorista uralensis* from the Kungurian Koshelvka Formation was transferred to *Kamopanorpa* (Trichoptera: Microptysmatidae) by Novokshonov (1992).

First: *Parachorista opposita* in Willmann (1978), Iva-Gora limestones, Soyana River, Arkhangelsk Region, Ural Mountains, Russian Federation.

Last: e.g. *Choristopanorpa drinnani* in Jell (2004), Koonwarra Fossil Bed (Korumburra Group), South Gippsland, Victoria, Australia. (Jell 2004 lists *Choristopanorpa* and *Neoparachorista* in Orthophlebiidae, where they were originally placed but have since been removed from and placed in Parachoristidae, according to the system in Novokshonov 2002a.)

F. Permocentropidae P2(Roadian)

First and Last: *Permocentropus philopotamoides* in Ross and Jarzembowski (1993), Iva-Gora limestones, Soyana River, Arkhangelsk Region, Ural Mountains, Russian Federation.

F. Permochoristidae (Agetopanorpidae, Caenoptilonidae, Choristopsychidae, Eosetidae, Idelopanorpidae, Mesochoristidae, Petrochoristidae, Petromantidae, Protochoristidae, Protopanorpidae, Tychtodelopteridae, Tychtopsychidae, Xenochoristidae) P1(Artinskian)-K1(Aptian)
This concept of the family is probably paraphyletic, according to the findings of Ren et al. (2009).

First: e.g. *Protopanorpa permiana* in Beckemeyer and Hall (2007), Wellington Formation, Elmo site, Dickinson County, Kansas, United States.

Last: *Liassochorista hebeiensis* in Novokshonov and Novokshonova (1997), Yixian Formation, Chengde, Hebei Province, China.

F. Permopanorpidae (Lithopanorpidae, Martynopanorpidae, Trachopterygidae) P1(Artinskian)-T3(Carnian)

First: e.g. *Permopanorpa inaequalis* in Beckemeyer and Hall (2007), Wellington Formation, Midco, Oklahoma, United States.

Last: e.g. *Neopermopanorpa mesembria* in Jell (2004), Mount Crosby Formation, Ipswich Basin, Queensland, Australia.

F. Permotanyderidae P3(Changhsingian)
Jell (2004) lists *Mesotanyderus jonesi* from the Upper Triassic Mount Crosby Formation in this family but Carpenter (1992b) placed it in Mecoptera *incertae sedis* and Ren et al. (2009) show the family occurring only in the Upper Permian.

e.g. *Permotanyderus ableptus* in Jell (2004), Belmont insect beds, Newcastle Coal Measures, Belmont/Warner's Bay, New South Wales, Australia.

F. Permotipulidae (Permilidae) P2(Wordian)-P3(Changhsingian)

First: *Permila borealis* in Krzemiński and Krzemińska (2003), IlŠinskoe Formation, Suriyokova (Suriekova), Kemerovo Region, Russian Federation.

Last: *Permotipula patricia* in Jell (2004), Belmont insect beds, Newcastle Coal Measures, Belmont/Warner's Bay, New South Wales, Australia.

F. Protomeropidae (Marimerobiidae, Permomeropidae, Platychoristidae, Protomeropeidae) C2(Bashkirian)-P3(Changhsingian)
The ordinal placement of this family remains contentious (e.g. Nel et al., 2007a; Sukatsheva et al., 2007).

First: *Westphalomerope maryvonneae* Nel et al., 2007a, Veine Maroc, Faisceau de Modeste, Bruay-la-Bussière, Pas-de-Calais, France.

Last: e.g. *Permomerope australis* in Sukatsheva et al. (2007), Belmont insect beds, Newcastle Coal Measures, Belmont/Warner's Bay, New South Wales, Australia.

F. Pseudopolycentropodidae (Pseudopolycentropidae, Pseudopolycentropididae) T2(Anisian)-K1(Albian)

First: *Pseudopolycentropus triasicus* in Grimaldi et al. (2005a), Grès à Voltzia, Bas-Rhin/Moselle, Northern Vosges Mountains, France.

Last: e.g. *Parapolycentropus burmiticus* Grimaldi & Rasnitsyn *in* Grimaldi et al., 2005a, Burmese amber (Burmite), Hukawng Valley, Kachin State, Myanmar.

F. Robinjohniidae P3(Changhsingian)
Novokshonov (2002a) mentions that a species of this family has been found in Krasnoyarsk Province of Siberia but does not give any further information.

First and Last: *Robinjohnia tillyardi* in Grimaldi and Engel (2005), Belmont insect beds, Newcastle Coal Measures, Belmont/Warner's Bay, New South Wales, Australia.

F. Sibiriothaumatidae Sukatsheva and Novokshonov, 1998 K1(Berriasian)

First and Last: *Sibiriothauma jakutensis* Sukatsheva and Novokshonov, 1998, Kempendyai locality, Suntar District, Sakha (Yakutia) Republic, Russian Federation.

F. Thaumatomeropidae (Thaumatomeropeidae) T3(Carnian)
Comprising the six species from the Madygen Formation formerly placed in Eomeropidae (Archibald et al., 2005).

e.g. *Thaumatomerope sogdiana* in Shcherbakov (2008b), Madygen Formation, Madygen/Dzhailoucho, south Fergana Valley, Kyrgyzstan.

F. Tomiochoristidae P2(Roadian)-T2(Ladinian)
Novokshonov (2002a) considered this a junior synonym of Kaltanidae but Hong (2006) maintains it as a separate family.

First: e.g. *Tomiochorista minuta* in Ross and Jarzembowski (1993), Kuznetsk Formation (Mitino Horizon), Kaltan, Kemerovo Region, Russian Federation.

Last: e.g. *Glyptochorista martynovae* Hong, 2006, Tongchuan Formation, Hejiafang, Tongchuan District, Shaanxi Province, China.

F. Volitorididae (Voltidorididae) K1(Aptian)

First and Last: *Volitoridia fulvis* in Sun et al. (2007a), Xiguayuan Formation, Fengning, Hebei Province, China.

**O. Megaloptera** Latreille, 1802 (Cordydalida)
Permian(Kungurian)-Quaternary(Holocene)

F. Corydalidae J3(Tithonian)-Holocene

First: Mentioned in Ponomarenko (2002b), Shar-Teg Formation, Shar-Teg Ula, Gobi-Altai Aimag, Mongolia.

F. Corydasialidae Wichard et al., 2005 Eoc.(Priabonian)

First and Last: *Corydasialis inexspectatus* Wichard et al., 2005, Baltic amber.

F. Euchauliodidae T3(Carnian)

First and Last: *Euchauliodes distinctus* in Wichard et al. (2005), Molteno Formation, KwaZulu-Natal, Karoo Basin, South Africa. (Ansorge 2001 suggested that this family may belong in Polyneoptera near to Grylloblattodea while Engel 2004b suggested it could represent stem-group Corydalidae.)

F. Parasialidae P1(Kungurian)-P2(Capitanian)

First: *Parasialis rozhkovi* Novokshonov, 1994b, Koshelevka Formation, Tshekarda, Ural Mountains, Russian Federation.

Last: *Parasialis ovata* Ponomarenko, 2000a, Tsankhi (Tsankhin) Formation, Bor-Tolgoy, Ömnögovi (South Gobi) Aimag, Mongolia.

F. Sialidae (Dobbertiniidae) J1(Toarcian)-Holocene

First: *Dobbertinia reticulata* in Engel and Grimaldi (2008a), Upper Lias, Dobbertin, Mecklenburg-Vorpommern, Germany.

**O. Neuroptera** Linnaeus, 1758 (Myrmeleontida, Planipennia, Schwickertoptera)
Permian(Artinskian)-Quaternary(Holocene)

F. Aetheogrammatidae Ren and Engel, 2008 K1(Aptian)

First and Last: *Aetheogramma speciosa* Ren and Engel, 2008, Jianshangou beds, Yixian Formation, Liaoning Province, China.

F. Araripeneuridae Martins-Neto, 2002 K1(Aptian)
Engel and Grimaldi (2008a) consider this to be a primitive subfamily of Myrmeleontidae.

e.g. *Caririneura regia* in Martins-Neto et al. (2007c), Crato Formation, Araripe Basin, Ceará, Brazil.

F. Archeosmylidae (Archaeosmylidae) P3(Wuchiapingian)-J1(Toarcian)
Engel and Grimaldi (2008a) include this family in Permithonidae but it is considered separate by Ponomarenko and Shcherbakov (2004) and Shcherbakov et al. (2009).

First: cf. *Archeosmylus* sp. in van Dijk and Geertsema (1999), Normandien (Estcourt) Formation, Beaufort Group, KwaZulu-Natal, Karoo Basin, South Africa.

Last: e.g. *Archeosmylus complexus* in Jarzembowski (1999), Upper Lias, Alderton, Gloucestershire, United Kingdom.

F. Ascalaphidae K1(Aptian)-Holocene
*Mesascalaphus* from the Yixian Formation belongs in Mesochrysopidae (Makarkin and Menon, 2005; Ren and Makarkin, 2009).

First: *Cratoscalapha electroneura* in Martill et al. (2007), Crato Formation, Araripe Basin, Ceará, Brazil.

F. Ascalochrysidae Ren and Makarkin, 2009 K1(Aptian)

First and Last: *Ascalochrysa megaptera* Ren and Makarkin, 2009, Jianshangou beds, Yixian Formation, Liaoning Province, China.

F. Babinskaiidae Martins-Neto and Vulcano, 1989 K1(Valanginian)-K1(Aptian)

First: e.g. *Baisonelia vitimica* Ponomarenko, 1992, Zaza Formation, Baissa, Buryatia, Russian Federation.

Last: e.g. *Babinskaia pulchra* in Martins-Neto et al. (2007c), Crato Formation, Araripe Basin, Ceará, Brazil.

F. Berothidae K1(Barremian)-Holocene

First: *Banoberotha enigmatica* in Engel and Grimaldi (2008a), Lebanese amber, unknown horizon, unknown locality, Lebanon.

F. Brongniartiellidae J3(Tithonian)-K1(Valanginian)
Makarkin (2010) restricts the composition of this family to the type genus and *Pseudopsychopsis*.

First: e.g. *Brongniartiella gigas* in Makarkin (2010), Solenhofen Lithographic Limestone, Solenhofen/Eichstadt, Bavaria, Germany.

Last: e.g. *Pseudopsychopsis gradata* Makarkin, 2010, Zaza Formation, Baissa, Buryatia, Russian Federation.

F. Chrysopidae (Limaiidae) J3(Oxfordian)-Holocene
Placement of Limaiidae within Chrysopidae after Ren and Makarkin (2009).

> First: e.g. *Mesypochrysa latipennis* in Nel et al. (2005a), Karabastau Formation, Karatau Range, Tien Shan mountains, Kazakhstan.

F. Coniopterygidae J3(Oxfordian)-Holocene
*Archiconiopteryx liasina* from the Upper Lias of Mecklenburg is a hemipteran (see Ansorge, 1996a).

> First: *Juraconiopteryx zherichini* in Engel and Grimaldi (2007b), Karabastau Formation, Karatau Range, Tien Shan mountains, Kazakhstan.

F. Dilaridae Eoc.(Priabonian)-Holocene

> First: *Cascadilar eocenicus* in Engel and Grimaldi (2008a), Baltic amber.

F. Epigambriidae J1(Toarcian)
This family is considered valid by Engel and Grimaldi (2008a). Makarkin and Archibald (2003) consider the type genus to be Neuroptera *incertae sedis*.

> First and Last: *Epigambria longipennis* in Makarkin and Archibald (2003), Upper Lias, Dobbertin, Mecklenburg-Vorpommern, Germany.

F. Grammolingiidae Ren, 2002a J2(Callovian)

> e.g. *Grammolingia boi* Ren, 2002a, Jiulongshan Formation, near Daohugou, Ningcheng county, Inner Mongolia, China.

F. Hemerobiidae (Promegalomidae) J3(Oxfordian)-Holocene

> First: *Promegalomus anomalus* in Engel and Grimaldi (2007b), Karabastau Formation, Karatau Range, Tien Shan mountains, Kazakhstan.

F. Ithonidae (Rapismatidae) K1(Barremian)-Holocene

> First: *Principiala rudgwickensis* Jepson et al., 2009, Upper Weald Clay Formation, Rudgwick Brickworks, near Horsham, West Sussex, United Kingdom.

F. Kalligrammatidae (Makarkiniidae) J1(Toarcian)-K1(Aptian)
Andersen (2001) moved *Paractinophlebia* (Upper Lias, Alderton, Gloucestershire, England) to Prohemerobiidae. *Makarkinia* is included here following Makarkin et al. (2009).

> First: Mentioned in Makarkin et al. (2009), Upper Lias, Kerkhofen, Bavaria, Germany.

Last: e.g. *Makarkinia adamsi* in Martill et al. (2007), Crato Formation, Araripe Basin, Ceará, Brazil.

F. Mantispidae (Liassochrysidae, Liassochrysopidae) J1(Toarcian)-Holocene
Wedmann and Makarkin (2007) consider *Mantispidiptera* and *Whalfera* not to belong to this family.

First: *Liassochrysa stigmatica* in Wedmann and Makarkin (2007), Upper Lias, Dobbertin, Mecklenburg-Vorpommern, Germany.

F. Mesithonidae J1(Toarcian)-K1(Valanginian)

First: *Sibithone prodroma* in Ansorge (1996a), Upper Lias, Grimmen, Mecklenburg-Vorpommern, Germany.

Last: e.g. *Mesithone angusta* Makarkin, 1999, Zaza Formation, Baissa, Buryatia, Russian Federation.

F. Mesoberothidae (Proberothidae) T3(Carnian)
Jell (2004) was apparently unaware that *Proberotha* Riek, 1955 was a junior homonym of *Proberotha* Krüger,1923 and was replaced with *Mesoberotha* by Carpenter (1991).

e.g. *Mesoberotha superba* in Jell (2004), Mount Crosby Formation, Ipswich Basin, Queensland, Australia. (As *Proberotha*.)

F. Mesochrysopidae (Allopteridae, Mesochrysopsidae, Tachinymphidae) J1(Toarcian)-K1(Aptian)
Allopteridae and Tachinymphidae placed here after Makarkin and Menon (2005), Menon and Makarkin (2008) and Ren and Makarkin (2009).

First: *Protoaristenymphes bascharagensis* in Nel et al. (2005a), Upper Lias, Bascharage and Sanem, Luxembourg district, Luxembourg.

Last: e.g. *Dryellina placida* Martins-Neto and Rodrigues, 2009, Crato Formation, Araripe Basin, Ceará, Brazil.

F. Myrmeleontidae (Myrmeleonidae, Myrmeliontidae) K1(Barremian)-Holocene

First: Mentioned in Engel and Grimaldi (2007b), Lebanese amber, unknown horizon, unknown locality, Lebanon. (This record requires confirmation.)

F. Nemopteridae (Roeslerianidae) K1(Aptian)-Holocene

First: e.g. *Roesleria exotica* in Martins-Neto et al. (2007c), Crato Formation, Araripe Basin, Ceará, Brazil.

F. Nevrorthidae (Neurorthidae) Eoc.(Priabonian)-Holocene
The placement in this family of a specimen in Burmese amber by Grimaldi et al. (2002) is not clear, according to Makarkin and Perkovsky (2009).

First: e.g. *Rophalis relicta* in Makarkin and Perkovsky (2009), Baltic amber.

F. Nymphidae (Nymphitidae) J2(Callovian)-Holocene
*Epigambria*, from the Lower Jurassic of Germany, belongs to its own family according to Engel and Grimaldi (2008a).

First: *Liminympha makarkini* Ren and Engel, 2007, Jiulongshan Formation, near Daohugou, Ningcheng county, Inner Mongolia, China.

F. Osmylidae (Epiosmylidae) J1(Sinemurian)-Holocene
The species of the Upper Triassic genus *Lithosmylidia* listed by Jell (2004)), are regarded as belonging to the Polystoechotidae or *Incertae sedis* by Engel and Grimaldi (2008a).

First: e.g. *Sogjuta speciosa* in Makarkin and Archibald (2003), Dzhil Formation, Sogyuty, Issyk-Kul, Kyrgyzstan.

F. Osmylitidae J3(Oxfordian)-K1(Valanginian)
Makarkin and Menon (2005) redefined the family as comprising *Chrysoleonites*, *Baissoleon* and *Osmylites* and considered it a monophyletic grouping separate from Mesochrysopidae, *contra* Ponomarenko (2003b). Similarly, Nel et al. (2005a) rejected the placement of *Osmylites* in Mesochrysopidae.

First: e.g. *Chrysoleonites ocellatus* in Carpenter (1992b), Karabastau Formation, Karatau Range, Tien Shan mountains, Kazakhstan.

Last: *Baissoleon cretaceus* Makarkin, 1990, Zaza Formation, Baissa, Buryatia, Russian Federation.

F. Osmylopsychopidae (Osmylopsychopsidae) T3(Carnian)-J1(Toarcian)

First: e.g. *Petropsychops superba* in Grimaldi and Engel (2005), Blackstone Formation, Ipswich Basin, Queensland, Australia.

Last: e.g. *Actinophlebia* sp. in Ansorge (1996a), Upper Lias, Dobbertin, Mecklenburg-Vorpommern, Germany. (Generic placement following Makarkin and Archibald 2005.)

F. Palaeoleontidae Martins-Neto, 1992 K1(Aptian)-K2(Coniacian)
Engel and Grimaldi (2008a) consider this as the basalmost subfamily of Myrmeleontidae.

First: e.g. *Parapalaeoleon magnus* Menon and Makarkin, 2008, Crato Formation, Araripe Basin, Ceará, Brazil.

Last: *Metahemerobius kalligrammus* in Menon and Makarkin (2008), Antibes Formation, Antibes, Kemerovo Region, Russian Federation. (The age of this species is often cited as Maastrichtian-Danian, however the deposit it is from is Coniacian [V. A. Makarkin pers. comm. 2011].)

F. Panfiloviidae (Grammosmylidae, Panfilovidae) J3(Oxfordian)-K1(Berriasian)
*Makarkinia* is considered to belong to the Kalligrammatidae by Makarkin et al. (2009).

First: *Panfilovia acuminata* in Makarkin and Archibald (2003), Karabastau Formation, Karatau Range, Tien Shan mountains, Kazakhstan.

Last: *Osmylogramma martinsoni* in Makarkin and Archibald (2003), Tsagan-Tsab, Khutel-Kara, Dornogovi (East Gobi) Aimag, Mongolia.

F. Permithonidae (Palaemerobiidae, Parasisyridae, Permegalomidae, Permopsychopsidae, Permosisyridae, Sialidopseidae, Sialidopsidae) P1(Artinskian)-T1(Induan)
The Upper Jurassic (Solenhofen) record in Jepson and Penney (2007) is an incorrect indirect referral to *Archeosmylus* from the Upper Lias (Toarcian) of England in Whalley (1988), which belongs to the Archeosmylidae.

First: e.g. *Permipsythone panfilovi* in Martins-Neto (2005), Irati Formation, Paraná Basin, São Paulo, Brazil.

Last: *Permantispa emelyanovi* Ponomarenko and Shcherbakov, 2004, Limptekon Formation, Tunguska Basin, Krasnoyarsk Krai, Siberian Federal District, Russian Federation.

F. Polystoechotidae (Mesopolystoechotidae) T3(Carnian)-Holocene

First: *Lithosmylidia lineata* in Engel and Grimaldi (2008a), Mount Crosby Formation, Ipswich Basin, Queensland, Australia. (The additional species of this genus in Jell 2004 are regarded as doubtful polystoechotids or *Incertae sedis* by Engel and Grimaldi 2008a.)

F. Prohemerobiidae J1(Toarcian)-J2(Callovian)
This family is in need of revision (Makarkin and Menon, 2007). *Prohemerobius aldertonensis* Whalley, 1988 is from the Upper Lias (Toarcian), not the Lower Lias as stated by Ponomarenko (1996).

First: e.g. *Prohemerobius dilaroides* in Makarkin and Menon (2007), Upper Lias, Dobbertin, Mecklenburg-Vorpommern, Germany.

Last: *Sinosmylites pectinatus* in Makarkin and Archibald (2005), Haifanggou Formation, Beipiao, Liaoning Province, China.

F. Psychopsidae T3(Carnian)-Holocene

First: *Triassopsychops superba* in Engel and Grimaldi (2008a), Blackstone Formation, Ipswich Basin, Queensland, Australia.

F. Rafaelidae (Rafaeliidae) K1(Aptian)
Engel and Grimaldi (2008a) do not consider the order Schwickertoptera Bechly, 2008 to be valid and maintain the position of this family in Neuroptera.

e.g. *Rafaeliana maxima* in Nel et al. (2006), Crato Formation, Araripe Basin, Ceará, Brazil.

F. Rhachiberothidae (Rachiberothidae) K1(Hauterivian)-Holocene

First: e.g. *Chimerhachiberotha acrasarii* Nel et al., 2005b, Jezzine amber, Jouar Ess-Souss, Mouhafazet Loubnan El-Janoubi, Lebanon.

F. Sisyridae Eoc.(Ypresian)-Holocene
*Cratosisyrops gonzagi* from the Aptian Crato Formation (Brazil) does not belong to this family (Nel et al., 2003a; Grimaldi and Engel, 2005).

First: *Paleosisyra eocenica* Nel et al., 2003a, Oise amber, Le Quesnoy, Houdancourt, Oise, Picardie, France.

F. Solenoptilidae J1(Toarcian)-Eoc.(Priabonian)
Makarkin (1998) restricted the composition of this family to the type species and tentatively *Oligogetes*.

First: *Solenoptilon kochi* in Makarkin and Archibald (2003), Upper Lias, Dobbertin, Mecklenburg-Vorpommern, Germany.

Last: *Oligogetes relictum* Makarkin, 1998, Bol'shaya Svetlovodnaya (Biamo), Barachek Creek, Pozharsky District, Primorye, Russian Federation.

**O. Raphidioptera** Navás, 1916 (Raphidiida, Raphidiodea, Raphidioidea)
Jurassic(Sinemurian)-Quaternary(Holocene)

F. Alloraphidiidae K1(Valanginian)-K2(Cenomanian)

First: e.g. *Alloraphidia asiatica* in Jepson and Jarzembowski (2008), Zaza Formation, Baissa, Buryatia, Russian Federation.

Last: *Alloraphidia dorfi* in Jepson and Jarzembowski (2008), Redmond Formation, Knob Lake District, Labrador, Canada.

F. Baissopteridae (Baissoraphidiidae) K1(Valanginian)-K1(Aptian)

First: e.g. *Baissoptera elongata* in Jepson and Jarzembowski (2008), Zaza Formation, Baissa, Buryatia, Russian Federation.

Last: e.g. *Baissoptera brasiliensis* in Jepson and Jarzembowski (2008), Crato Formation, Araripe Basin, Ceará, Brazil.

F. Inocelliidae (Inocellidae) J2(Callovian)-Holocene

First: *Sinoinocellia liaoxiensis* in Jepson and Jarzembowski (2008), Haifanggou Formation, Beipiao, Liaoning Province, China. (Jepson and Jarzembowski 2008 list this species as Lower Cretaceous in age but the original description clearly attributes it to the Haifanggou Formation which is taken here to be Callovian.)

F. Mesoraphidiidae (Huaxiaraphidiidae, Jilinoraphidiidae, Mesoraphidae, Sinoraphidiidae) J1(Sinemurian)-K2(Campanian)

First: *Metaraphidia confusa* in Jepson and Jarzembowski (2008), Black Ven Marls, Charmouth, Dorset, United Kingdom.

Last: Figured in Engel and Grimaldi (2008a), Canadian amber, Grassy Lake, Alberta, Canada.

F. Priscaenigmatidae Engel, 2002(Eomantispidae) J1(Sinemurian)-J1(Toarcian)
Aspöck and Aspöck (2004) consider this family not to belong to this order, however Perrichot and Engel (2007) defend the placement.

First: *Priscaenigma obtusa* in Engel (2002), Black Ven Marls, Charmouth, Dorset, United Kingdom.

Last: *Hondelagia reticulata* in Engel (2002), Upper Lias, Hondelage, Braunschweig, Lower Saxony, Germany.

F. Raphidiidae (Raphididae) K2(Campanian)-Holocene
*Austroraphidia brasiliensis* from the Crato Formation is listed under Baissopteridae by Jepson and Jarzembowski (2008).

First: Mentioned in McKellar et al. (2008), Canadian amber, Grassy Lake, Alberta, Canada.

**O. Siphonaptera** Latreille, 1825 (Pulicida) Cretaceous(Aptian)-Quaternary(Holocene)

First: *Tarwinia australis* in Grimaldi and Engel (2005), Koonwarra Fossil Bed (Korrumburra Group), South Gippsland, Victoria, Australia. *Palaeopsylla* belongs in Ctenophthalmidae, leaving Hystrichopsyllidae without a fossil record.

F. Ctenophthalmidae Eoc.(Priabonian)-Holocene

First: e.g. *Palaeopsylla baltica* in Whiting et al. (2008), Baltic amber.

F. Pulicidae Mio.(Burdigalian)-Holocene
The specimen figured as "Pulicid indet." by Jell (2004) is too fragmentary to identify, according to Grimaldi and Engel (2005).

First: *Pulex larimerius* Lewis and Grimaldi, 1997, Dominican amber, Cordillera Septentrional, near Santiago, Dominican Republic.

F. Rhopalopsyllidae (Rhopallopsyllidae) Mio.(Burdigalian)-Holocene

First: *Rhopalopsyllus* sp. in Whiting et al. (2008), Dominican amber, Cordillera Septentrional, near Santiago, Dominican Republic.

**O. Strepsiptera** Kirby, 1815b (Stylopida) Cretaceous(Albian)-Quaternary(Holocene)

First: *Cretostylops engeli* (Grimaldi et al., 2005b) and an undescribed mengeid, both from Burmese amber.

F. Bohartillidae Mio.(Burdigalian)-Holocene

First: e.g. *Bohartilla kinzelbachi* in Pérez-Gelabert (2008), Dominican amber, Cordillera Septentrional, near Santiago, Dominican Republic.

F. Elenchidae Mio.(Burdigalian)-Holocene

First: *Protelencholax schleei* in Pérez-Gelabert (2008), Dominican amber, Cordillera Septentrional, near Santiago, Dominican Republic.

F. Mengeidae K1(Albian)-Eoc.(Priabonian)
This family is likely paraphyletic (Grimaldi et al., 2005b).

First: Mentioned in Poinar and Poinar (2008), Burmese amber (Burmite), Hukawng Valley, Kachin State, Myanmar.

Last: e.g. *Mengea tertiaria* in Pohl et al. (2005), Baltic amber.

F. Myrmecolacidae Eoc.(Lutetian)-Holocene
*Pseudococcites eocaenicus* from the Eocene brown coal of the Geisel valley near Halle (Saale, Germany) is Strepsiptera *incertae sedis* (Pohl, 2009).

First: *Stichotrema* sp. in Grimaldi et al. (2005b), Messel Formation, Grube Messel, Hesse, Germany.

F. Protoxenidae Pohl et al., 2005 Eoc.(Priabonian)

First and Last: *Protoxenos janzeni* Pohl et al., 2005, Baltic amber.

F. Stylopidae Eoc.(Priabonian)-Holocene

First: *Jantarostylops kinzelbachi* in Grimaldi et al. (2005b), Baltic amber.

**O. Trichoptera** Kirby, 1815a (Phryganaeida, Phryganeida)
Permian(Sakmarian)-Quaternary(Holocene)

Here including stem group Amphiesmenoptera.

F. Baissoferidae J3(Oxfordian)-K1(Valanginian)

First: *Baissoferus udaensis* in Sukatsheva (1985), Uda Formation, Uda River, Buryatia, Russian Federation.

Last: e.g. *Baissoferus latus* in Ivanov and Sukatsheva (2002), Zaza Formation, Baissa, Buryatia, Russian Federation.

F. Beraeidae Eoc.(Priabonian)-Holocene

First: e.g. *Bereodes pectinatus* in Wichard and Weitschat (1996), Baltic amber.

F. Brachycentridae K1(Valanginian)-Holocene

First: *Baissoplectrum separatum* Ivanov, 2006, Zaza Formation, Baissa, Buryatia, Russian Federation.

F. Calamoceratidae J3(Tithonian)-Holocene

First: e.g. Mentioned in Ponomarenko et al. (2009), Doronino Formation, Chernovskie Kopi, Chita, Transbaikalia, Russian Federation.

F. Cladochoristidae P2(Wordian)-T3(Carnian)

First: *Cladochorista* sp. in Aristov and Bashkuev (2008), Chepanikha locality, Rossokha River valley, Zavjalovskii District, Udmurt Republic, Russian Federation.

Last: e.g. *Cladochorista multivenosa* in Ivanov and Sukatsheva (2002), Madygen Formation, Madygen/Dzhailoucho, south Fergana Valley, Kyrgyzstan.

F. Dipseudopsidae K2(Turonian)-Holocene

First: e.g. *Phylocentropus swolenskyi* Wichard and Lüer, 2003, New Jersey amber, South Amboy Fire Clay (Raritan Formation), New Jersey, United States.

F. Dysoneuridae (Disoneuridae) J2(Aalenian)-K1(Berriasian)

First: *Oncovena borealis* in Sukatsheva (2000), Itat Formation, Kubekovo, Krasnoyarsk Krai, Siberian Federal District, Russian Federation. (Ansorge 2002 synonymised *Oncovena* with *Liadotaulius* in family uncertain, but refers to them separately in Ansorge 2003b. *Oncovena* is included in this family by Ivanov and Sukatsheva 2002, which is followed here.)

Last: e.g. *Palaeoludus popovi* Sukatsheva and Jarzembowski, 2001, Durlston Formation (Stair Hole Member), Durlston Bay, Dorset, United Kingdom.

F. Ecnomidae Eoc.(Priabonian)-Holocene

First: e.g. *Archaeotinodes igneusaper* Melnitsky, 2009, Baltic amber.

F. Electralbertidae K2(Campanian)

First and Last: *Electralberta cretacica* in McKellar et al. (2008), Canadian amber, Grassy Lake, Alberta, Canada.

F. Glossosomatidae J3(Tithonian)-Holocene

First: *Dajella tenera* in Ivanov and Melnitsky (2006), Glushkovo Formation, Daya, Transbaikalia, Russian Federation.

F. Goeridae Eoc.(Priabonian)-Holocene

First: e.g. *Lithax herrlingi* in Weitschat and Wichard (2002), Baltic amber.

F. Helicophidae K1(Barremian)-Holocene

First: Figured in Sukatsheva and Jarzembowski (2001), Upper Weald Clay Formation, Capel, Surrey, United Kingdom. (This specimen was only tentatively placed in Helicophidae by Sukatsheva and Jarzembowski 2001.)

F. Helicopsychidae Eoc.(Priabonian)-Holocene

First: e.g. *Electrohelicopsyche taeniata* in Weitschat and Wichard (2002), Baltic amber.

F. Hydrobiosidae (Atopsychidae) J3(Tithonian)-Holocene

First: *Bullivena grandis* in Sukatsheva (2000), Shar-Teg Formation, Shar-Teg Ula, Gobi-Altai Aimag, Mongolia.

F. Hydropsychidae Eoc.(Priabonian)-Holocene

First: e.g. *Hydropsyche viduata* in Wichard and Weitschat (1996), Baltic amber.

F. Hydroptilidae K1(Aptian)-Holocene

First: e.g. *Cratorella media* in Bechly (2007a), Crato Formation, Araripe Basin, Ceará, Brazil.

F. Lepidostomatidae K1(Barremian)-Holocene

First: *Eucrunoecia ridicula* Sukatsheva and Jarzembowski, 2001, Upper Weald Clay Formation, Capel, Surrey, United Kingdom.

F. Leptoceridae K1(Valanginian)-Holocene

First: *Creterotesis coprolithica* Ivanov, 2006, Zaza Formation, Baissa, Buryatia, Russian Federation.

F. Limnephilidae Eoc.(Priabonian)-Holocene

First: Mentioned in Ivanov and Sukatsheva (2002), Passamari Formation, Ruby River Basin, Montana, United States.

F. Microptysmatidae P1(Sakmarian)-P3(Changhsingian)

First: *Microptysmella moravica* in Zajíc and Štamberg (2004), Obora locality, Bačov Beds, Letovice Formation, Moravia, Czech Republic.

Last: e.g. *Kamopanorpa latipennata* Novokshonov, 1994a, Maichat/Ak-Kolka Formation, Karaungir River, Saur Mountains, Vostochno-Kazakhstanskaya oblast, Kazakhstan.

F. Molannidae Eoc.(Priabonian)-Holocene

First: e.g. *Molanna crassicornis* in Wichard and Weitschat (1996), Baltic amber.

F. Necrotauliidae (Necrotaulidae) T3(Carnian)-K1(Hauterivian)
This paraphyletic family is sometimes considered to be stem-Amphiesmenoptera (Ansorge, 2003b) or stem-Trichoptera (Grimaldi and Engel, 2005).

First: e.g. *Necrotaulius proximus* in Kozlov et al. (2002), Madygen Formation, Madygen/Dzhailoucho, south Fergana Valley, Kyrgyzstan.

Last: *Necrotaulius mantellorum* in Jarzembowski (1995), Lower Weald Clay Formation, Capel, Surrey, United Kingdom.

F. Ningxiapsychidae (Ningsiapschidae) Hong and Li, 2004 K1(Albian)

First and Last: *Ningxiapsyche fangi* Hong and Li, 2004, Naijiahe Formation, Liupanshan, Ningxia Province, China.

F. Odontoceridae (Odontoceratidae) K2(Santonian)-Holocene

First: Mentioned in Ivanov and Sukatsheva (2002), Yantardakh amber, Kheta Formation, Taimyr, Krasnoyarsk Krai, Siberian Federal District, Russian Federation.

F. Philopotamidae (Phylopotamidae) T3(Carnian)-Holocene

First: *Prophilopotamus asiaticus* in Wang et al. (2009d), Madygen Formation, Madygen/Dzhailoucho, south Fergana Valley, Kyrgyzstan. (This record is doubtful according to Shcherbakov 2008b.)

F. Phryganeidae (Phryganaeidae) J3(Tithonian)-Holocene

First: e.g. Mentioned in Ponomarenko et al. (2009), Glushkovo Formation, Unda, Transbaikalia, Russian Federation.

F. Plectrotarsidae (Plectotarsidae) J3(Tithonian)-Holocene

First: e.g. Mentioned in Ponomarenko et al. (2009), Doronino Formation, Chernovskie Kopi, Chita, Transbaikalia, Russian Federation.

F. Polycentropodidae K1(Berriasian)-Holocene

First: e.g. Mentioned in Ponomarenko et al. (2009), Kempendyai locality, Suntar District, Sakha (Yakutia) Republic, Russian Federation.

F. Prorhyacophilidae T3(Carnian)

e.g. *Prorhyacophila furcata* in Ivanov and Sukatsheva (2002), Madygen Formation, Madygen/Dzhailoucho, south Fergana Valley, Kyrgyzstan.

F. Psychomyiidae (Psychomyidae) K2(Campanian)-Holocene

First: Mentioned in McKellar et al. (2008), Canadian amber, Grassy Lake, Alberta, Canada.

F. Rhyacophilidae J3(Oxfordian)-Holocene

First: *Rhyacophila*? sp. in Ross and Jarzembowski (1993), Bada (Zun-Nemetey) Formation, Mogzon, Transbaikalia, Russian Federation. (Locality record also in Ponomarenko et al. 2009.)

F. Sericostomatidae K2(Santonian)-Holocene
Ivanov and Sukatsheva (2002) suggest that specimens from Bon-Tsagan could belong to this family, which would extend the record back to the Barremian.

First: Mentioned in Sinitshenkova (2002c), Yantardakh amber, Kheta Formation, Taimyr, Krasnoyarsk Krai, Siberian Federal District, Russian Federation.

F. Stenopsychidae Eoc.(Priabonian)-Holocene

First: *Stenopsyche imitata* in Wichard and Weitschat (1996), Baltic amber.

F. Stereochoristidae T3(Carnian)

First and Last: *Stereochorista frustrata* in Jell (2004), Blackstone Formation, Ipswich Basin, Queensland, Australia. (Note that Carpenter 1992b considered this genus unplaced within Neoptera.)

F. Taymyrelectronidae (Taimyrelectronidae) K2(Santonian)

First and Last: *Taymyrelectron sukatshevae* in Ross and Jarzembowski (1993), Yantardakh amber, Kheta Formation, Taimyr, Krasnoyarsk Krai, Siberian Federal District, Russian Federation.

F. Uraloptysmatidae Ivanov, 1992 P1(Kungurian)

First and Last: *Uraloptysma maculata* in Ivanov and Sukatsheva (2002), Koshelevka Formation, Tshekarda, Ural Mountains, Russian Federation.

F. Vitimotauliidae J3(Tithonian)-K2(Cenomanian)

First: e.g. *Multimodus* sp. in Ponomarenko et al. (2009), Ulan-Ereg, Khoutiyn-Khotgor, Dund-Gobi Aimag, Mongolia.

Last: *Multimodus bureensis* in Sinitshenkova (2002c), Kyndal Formation, Urgal River Basin, Far Eastern Federal District, Russian Federation.

F. Xiphocentronidae Mio.(Aquitanian)-Holocene

First: *Xiphocentron chiapasi* Wichard et al., 2006, Mexican amber, Simojovel, Chiapas, Mexico.

**Holometabola** *incertae sedis*

F. Dictyodipteridae J1(Sinemurian)

e.g. *Dictyodiptera multinervis* in Carpenter (1992b), Dzhil Formation, Sogyuty, Issyk-Kul, Kyrgyzstan.

F. Saurophthiridae (Saurophthiriidae) K1(Valanginian)
*Saurophthiroides mongolicus* was included in Chresmodidae by Nel et al. (2004a).

First and Last: *Saurophthirus longipes* in Grimaldi and Engel (2005), Zaza Formation, Baissa, Buryatia, Russian Federation.

F. Strashilidae Rasnitsyn, 1993 J3(Oxfordian)

First and Last: *Strashila incredibilis* in Grimaldi and Engel (2005), Bada (Zun-Nemetey) Formation, Mogzon, Transbaikalia, Russian Federation.

## References

Alonso, J., Arillo, A., Barrón, E., Corral, J. C., Grimalt, J., López, J. F., López, R., Martínez-Delclòs, X., Ortuño, V., Peñalver, E., and Trincão, P. R. (2000). A new fossil resin with biological inclusions in Lower Cretaceous deposits from álava (northern Spain, Basque-Cantabrian Basin). *Journal of Paleontology*, 74(1):158–178.

Amorim, D. S. (2008). Catalogue of neotropical Diptera. Scatopsidae. *Neotropical Diptera*, 4:1–17.

Amorim, D. S. and Grimaldi, D. A. (2006). Valeseguyidae, a new family of Diptera in the Scatopsoidea, with a new genus in Cretaceous amber from Myanmar. *Systematic Entomology*, 31(3):508–516.

Andersen, N. M. (1998). Water striders from the Paleogene of Denmark with a review of the fossil record and evolution of semiaquatic bugs (Hemiptera, Gerromorpha). *Biologiske Skrifter*, 50:1–157.

Andersen, N. M. and Grimaldi, D. A. (2001). A fossil water measurer from the mid-Cretaceous Burmese amber (Hemiptera: Gerromorpha: Hydrometridae). *Insect Systematics & Evolution*, 32(4):381–392.

Andersen, S. (2001). Silky lacewings (Neuroptera: Psychopsidae) from the Eocene-Paleocene transition of Denmark with a review of the fossil record and comments on phylogeny and zoogeography. *Insect Systematics & Evolution*, 32:419–438.

Ansorge, J. (1993). *Dobbertiniopteryx capniomimus* gen. et sp. nov. - die erste Steinfliege (Insecta: Plecoptera) aus dem Europäischen Jura. *Paläontologische Zeitschrift*, 67(3/4):287–292.

Ansorge, J. (1996a). Insekten aus dem oberen Lias von Grimmen (Vorpommern, Norddeutschland). *Neue Paläontologische Abhandlungen*, 2:1–132.

Ansorge, J. (1996b). Zur systematischen Position von *Schesslitziella haupti* Kuhn 1952 (Insecta: Phasmatodea) aus dem Oberen Lias von Nordfranken (Deutschland). *Paläontologische Zeitschrift*, 70(3/4):475–479.

Ansorge, J. (1999). *Heterophlebia buckmani* (Brodie 1845) (Odonata: "Anisozygoptera") - das erste Insekt aus dem untertoarcischen Posidonienschiefer von Holzmaden (Wüttemberg, SW Deutschland). *Stuttgarter Beiträge zur Naturkunde Serie B (Geologie und Paläontologie)*, 275:1–9.

Ansorge, J. (2001). *Dobbertinia reticulata* Handlirsch 1920 from the Lower Jurassic of Dobbertin (Mecklenburg/Germany) Ũ the oldest representative of Sialidae (Megaloptera). *Neues Jahrbuch für Geologie und Paläontologie, Monatshefte*, 2001(9):553–564.

Ansorge, J. (2002). Revision of the "Trichoptera" described by Geinitz and Handlirsch from the Lower Toarcian of Dobbertin (Germany) based on new material. *Nova Supplementa Entomologica*, 15:55–74.

Ansorge, J. (2003a). Insects from the lower Toarcian of middle Europe and England. *Acta zoologica cracoviensia*, 46(suppl.- Fossil Insects):291–310.

Ansorge, J. (2003b). Upper Liassic amphiesmenopterans (Trichoptera + Lepidoptera) from Germany - a review. *Acta zoologica cracoviensia*, 46(suppl.- Fossil Insects):285–290.

Ansorge, J. and Krzemiński, W. (1995). Revision of *Mesorhyphus* Handlirsch, *Eoplecia* Handlirsch and *Heterorhyphus* Bode (Diptera: Anisopodomorpha, Bibionomorpha) from the Upper Liassic of Germany. *Paläontologische Zeitschrift*, 69(1/2):167–172.

Ansorge, J. and Krzemiński, W. (2002). Lower Jurassic tanyderids (Diptera: Tanyderidae) from Germany. *Studia dipterologica*, 9(1):21–29.

Archibald, S. B. (2005). New Dinopanorpidae (Insecta: Mecoptera) from the Eocene Okanagan Highlands (British Columbia, Canada; Washington State, USA). *Canadian Journal of Earth Sciences*, 42:119–136.

Archibald, S. B. (2009). New Cimbrophlebiidae (Insecta: Mecoptera) from the Early Eocene at McAbee, British Columbia, Canada and Republic, Washington, USA. *Zootaxa*, 2249:51–62.

Archibald, S. B., Cover, S. P., and Moreau, C. S. (2006). Bulldog ants of the Eocene Okanagan Highlands and history of the subfamily (Hymenoptera: Formicidae: Myrmeciinae). *Annals of the Entomological Society of America*, 99(3):487–523.

Archibald, S. B., Rasnitsyn, A. P., and Akhmetiev, M. A. (2005). The ecology and distribution of Cenozoic Eomeropidae (Mecoptera), and a new species of *Eomerope* Cockerell from the early Eocene McAbee locality, British Columbia, Canada. *Annals of the Entomological Society of America*, 98(4):503–514.

Arillo, A. and Engel, M. S. (2006). Rock crawlers in Baltic amber (Notoptera: Mantophasmatodea). *American Museum Novitates*, 3539:1–10.

Arillo, A. and Ortuño, V. M. (2005). Catalogue of fossil insect species described from Dominican amber (Miocene). *Stuttgarter Beiträge zur Naturkunde Serie B (Geologie und Paläontologie)*, 352:1–68.

Arillo, A., Peñalver, E., and García-Gimeno, V. (2009). First fossil *Litoleptis* (Diptera: Spaniidae) from the Lower Cretaceous amber of San Just (Teruel Province, Spain). *Zootaxa*, 2026:33–39.

Aristov, D. S. (2000a). A new family of early Permian grylloblattids (Insecta: Grylloblattida) from Ural Mountains. *Far Eastern Entomologist*, 85:1–4.

Aristov, D. S. (2000b). New insects of the order Grylloblattida (Insecta) from the Lower Permian of the middle Urals. *Paleontological Journal*, 34(5):519–521.

Aristov, D. S. (2004a). The fauna of grylloblattid insects (Grylloblattida) from the end of the late Permian to the first half of the Triassic. *Paleontological Journal*, 38(5):514–521.

Aristov, D. S. (2004b). The fauna of grylloblattid insects (Grylloblattida) of the Lower Permian locality of Tshekarda. *Paleontological Journal*, 38((Suppl. 2)):S80–S145.

Aristov, D. S. (2004c). Grylloblattids of the family Chaulioditidae (=Tomiidae syn. nov.) (Insecta: Grylloblattida) from the Upper Permian of the Orenburg Region. *Paleontological Journal*, 38(Suppl. 2):S146–S149.

Aristov, D. S. (2005). New Grylloblattids (Insecta: Grylloblattida) from the Triassic of eastern Europe, eastern Kazakhstan and Mongolia. *Paleontological Journal*, 39(2):173–177.

Aristov, D. S. (2008a). New Grylloblattida (Insecta) from the Middle and Upper Permian of the Russia. *Far Eastern Entomologist*, 188:1–7.

Aristov, D. S. (2008b). New grylloblattids of the family Megakhosaridae (Insecta: Grylloblattida) from the Permian of Russia. *Paleontological Journal*, 42(3):269–272.

Aristov, D. S. (2009a). A new family of the order Grylloblattida (Insecta) from the Middle Permian of Russia. *Paleontological Journal*, 43(2):178–182.

Aristov, D. S. (2009b). New Grylloblattida (Insecta) from Kargala locality (Russia; Middle Permian). *Far Eastern Entomologist*, 192:1–8.

Aristov, D. S. (2009c). New grylloblattids of the family lemmatophoridae (insecta: Grylloblattida) from the permian of russia. *Paleontological Journal*, 43(3):272–276.

Aristov, D. S. (2009d). Review of the stratigraphic distribution of Permian Grylloblattida (Insecta), with descriptions of new taxa. *Paleontological Journal*, 43(6):643–651.

Aristov, D. S. and Bashkuev, A. S. (2008). New insects (Insecta: Mecoptera, Grylloblattida) from the Middle Permian Chepanikha Locality, Udmurtiya. *Paleontological Journal*, 42(2):159–165.

Aristov, D. S., Novokshonov, V. G., and Pan'kov, N. N. (2006). Taxonomy of the fossil grylloblattid nymphs (Insecta: Grylloblattida). *Paleontological Journal*, 40(1):79–89.

Aristov, D. S., Prevec, R., and Mostovski, M. B. (2009a). New and poorly known grylloblattids (Insecta: Grylloblattida) from the Lopingian of the Lebombo Basin, South Africa. *African Invertebrates*, 50(2):279–286.

Aristov, D. S. and Rasnitsyn, A. P. (2008). Position and taxonomy of the Permian fossil insect family Permembiidae (Insecta: Palaeomanteida = Miomoptera). *Russian Entomological Journal*, 17(4):327–334.

Aristov, D. S. and Rasnitsyn, A. P. (2009). The family Tillyardembiidae Zalessky, 1938 and the system of the plecopteroid insects. *Russian Entomological Journal*, 18(4):257–264.

Aristov, D. S., Wappler, T., and Rasnitsyn, A. P. (2009b). New and little-known grylloblattids of the family Geinitziidae (Insecta: Grylloblattida) from the Triassic and Jurassic of Europe, Asia, and South Africa. *Paleontological Journal*, 43(4):418–424.

Aspöck, U. and Aspöck, H. (2004). Two significant new snakeflies from Baltic amber, with discussion on autapomorphies of the order and its included taxa (Raphidioptera). *Systematic Entomology*, 29(1):11–19.

Azar, D. (2007). Preservation and accumulation of biological inclusions in Lebanese amber and their significance. *Comptes Rendus Palevol*, 6(1-2):151–156.

Azar, D., Hajar, L., Indary, C., and Nel, A. (2008). Paramesopsocidae, a new Mesozoic psocid family (Insecta: Psocodea "Psocoptera": Psocomorpha). *Annales de la Société entomologique de France (Nouvelle série)*, 44(4):459–470.

Azar, D. and Nel, A. (2004). Four new Psocoptera from Lebanese amber (Insecta: Psocomorpha: Trogiomorpha). *Annales de la Société entomologique de France (Nouvelle série)*, 40(2):185–192.

Azar, D. and Nel, A. (2008). First Baltic amber megapodagrionid damselfly (Odonata: Zygoptera). *Annales de la Société entomologique de France (Nouvelle série)*, 44(4):451–457.

Azar, D., Nel, A., and Néraudeau, D. (2009). A new Cretaceous psocodean family from the Charente-Maritime amber (France) (Insecta, Psocodea, Psocomorpha). *Geodiversitas*, 31(1):117–127.

Barraclough, D. A. and McAlpine, D. K. (2006). Natalimyzidae, a new African family of acalyptrate flies (Diptera: Schizophora: Sciomyzoidea). *African Invertebrates*, 47:117–134.

Basibuyuk, H. H., Rasnitsyn, A. P., Fitton, M. G., and Quicke, D. L. J. (2002). The limits of the family Evaniidae (Insecta: Hymenoptera) and a new genus from Lebanese amber. *Insect Systematics & Evolution*, 33(1):23–34.

Baz, A. and Ortuño, V. M. (2000). Archaeatropidae, a new family of Psocoptera from the Cretaceous amber of Alava, northern Spain. *Annals of the Entomological Society of America*, 93(3):367–373.

Baz, A. and Ortuño, V. M. (2001). New genera and species of empheriids (Psocoptera: Empheriidae) from the Cretaceous amber of Alava, northern Spain. *Cretaceous Research*, 22(5):575–584.

Beattie, R. (2007). The geological setting and palaeoenvironmental and palaeoecological reconstructions of the Upper Permian insect beds at Belmont, New South Wales, Australia. *African Invertebrates*, 48(1):41–57.

Bechly, G. (1996). Morphologische Untersuchungen am Flügelgeäder der rezenten Libellen und deren Stammgruppenvertreter (Insecta: Pterygota: Odonata), unter besonderer Berücksichtigung der Phylogenetischen Systematik und des Grundplanes der Odonata. *Petalura, Special Volume*, 2:1–402.

Bechly, G. (1997). New fossil odonates from the Upper Triassic of Italy, with a redescription of *Italophlebia gervasuttii* Whalley, and a reclassification of Triassic dragonflies (Insecta: Odonata). *Rivista del Museo Civico di Scienze Naturali "Enrico Caffi"*, 19:31–70.

Bechly, G. (1998a). *Juracordulia schiemenzi* gen. et. [sic] sp. nov., Eine neue Libelle aus den Solnhofener Plattenkalken (Insecta: Odonata: Anisoptera). *Archaeopteryx*, 16:29–36.

Bechly, G. (1998b). New fossil damselflies from Baltic amber, with description of a new species, a redescription of *Litheuphaea carpenteri* Fraser, and a discussion on the phylogeny of Epallagidae (Zygoptera: Caloptera). *International Journal of Odonatology*, 1(1):33–63.

Bechly, G. (1998c). New fossil dragonflies from the Lower Cretaceous Crato Formation of north-east Brazil (Insecta: Odonata. *Stuttgarter Beiträge zur Naturkunde Serie B (Geologie und Paläontologie)*, 264:1–66.

Bechly, G. (2000). Two new fossil dragonfly species (Insecta: Odonata: Anisoptera: Araripegomphidae and Lindeniidae) from the Crato Limestone (Lower Cretaceous, Brazil). *Stuttgarter Beiträge zur Naturkunde Serie B (Geologie und Paläontologie)*, 296:1–16.

Bechly, G. (2003). Description of a new species of *Nannogomphus* (Insecta: Odonata: Nannogomphidae) from the Upper Jurassic Solenhofen Limestone in Germany. *Stuttgarter Beiträge zur Naturkunde Serie B (Geologie und Paläontologie)*, 339:1–6.

Bechly, G. (2005a). A new fossil dragonfly (Anisoptera: Corduliidae) from the Paleocene Fur Formation (Mo clay) of Denmark. *Stuttgarter Beiträge zur Naturkunde Serie B (Geologie und Paläontologie)*, 358:1–7.

Bechly, G. (2005b). A re-description of "*Stenophlebia*" *casta* (Insecta: Odonata: Parastenophlebiidae n. fam.) from the Upper Jurassic Solenhofen Limestone in Germany. *Stuttgarter Beiträge zur Naturkunde Serie B (Geologie und Paläontologie)*, 359:1–12.

Bechly, G. (2007a). 11.21 Trichoptera and Lepidoptera: caddisflies and butterflies. In Martill, D. M., Bechly, G., and Loveridge, R. F., editors, *The Crato Fossil Beds of Brazil: Window into an Ancient World*, pages 387–393. Cambridge University Press.

Bechly, G. (2007b). 11.5 Odonata: damselflies and dragonflies. In Martill, D. M., Bechly, G., and Loveridge, R. F., editors, *The Crato Fossil Beds of Brazil: Window into an Ancient World*, pages 184–222. Cambridge University Press.

Bechly, G. (2007c). 11.8 'Blattaria': cockroaches and roachoids. In Martill, D. M., Bechly, G., and Loveridge, R. F., editors, *The Crato Fossil Beds of Brazil: Window into an Ancient World*, pages 239–249. Cambridge University Press.

Bechly, G. (2007d). 11.9 Isoptera: termites. In Martill, D. M., Bechly, G., and Loveridge, R. F., editors, *The Crato Fossil Beds of Brazil: Window into an Ancient World*, pages 249–262. Cambridge University Press.

Bechly, G., Nel, A., Martínez-Delclòs, X., and Fleck, G. (1998). Four new dragonflies from the Upper Jurassic of Germany and the Lower Cretaceous of Mongolia (Anisoptera: Hemeroscopidae, Sonidae, and Proterogomphidae fam. nov.). *Odonatologica*, 27(2):149–187.

Bechly, G., Nel, A., Martínez-Delclòs, X., Jarzembowski, E. A., Coram, R., Martill, D., Fleck, G., Escuillié, F., Wisshak, M. M., and Maisch, M. (2001). A revision and phylogenetic study of Mesozoic Aeshnoptera, with description of numerous new taxa (Insecta: Odonata: Anisoptera). *Neue Paläontologische Abhandlungen*, 4:1–219.

Bechly, G. and Szwedo, J. (2007). 11.14 Coleorrhyncha: moss bugs. In Martill, D. M., Bechly, G., and Loveridge, R. F., editors, *The Crato Fossil Beds of Brazil: Window into an Ancient World*, pages 313–317. Cambridge University Press.

Bechly, G. and Ueda, K. (2002). The first fossil record and first New World record for the dragonfly clade Chlorogomphida (Insecta: Odonata: Anisoptera: Araripechlorogomphidae n. fam.) from the Crato Limestone (Lower Cretaceous, Brazil). *Stuttgarter Beiträge zur Naturkunde Serie B (Geologie und Paläontologie)*, 328:1–11.

Bechly, G. and Wichard, W. (2008). Damselfly and dragonfly nymphs in Eocene Baltic amber (Insecta: Odonata), with aspects of their palaeobiology. *Palaeodiversity*, 1:37–73.

Bechly, G. and Wittmann, M. (2000). Two new tropical bugs (Insecta: Heteroptera: Thaumastocoridae - Xylastodorinae and Hypsipterygidae) from Baltic amber. *Stuttgarter Beiträge zur Naturkunde Serie B (Geologie und Paläontologie)*, 289:1–11.

Beckemeyer, R. J. (2000). The Permian insect fossils of Elmo, Kansas. *The Kansas School Naturalist*, 46(1):1–16.

Beckemeyer, R. J. (2004a). A new species of the extinct family Lophioneuridae from the Lower Permian Wellington Formation of Noble County, Oklahoma. *Journal of the Kansas Entomological Society*, 77(2):132–136.

Beckemeyer, R. J. (2004b). Raaschiidae (Grylloblattida: Protoperlina), a new insect family from the Lower Permian Wellington Formation of Noble County, Oklahoma. *Journal of the Kansas Entomological Society*, 77(3):215–221.

Beckemeyer, R. J. (2009a). *Artinska ovata* (Sellards) 1909 and *Paraprisca fragilis* (Sellards) 1909 (Insecta: Polyneoptera: Lemmatophoridae) newly reported from the Lower Permian of Noble County, Oklahoma, with notes on Wellington Formation Lemmatophoridae. *Transactions of the Kansas Academy of Science*, 112(1/2):45–56.

Beckemeyer, R. J. (2009b). *Ligogramma wichita*, a new species of Caloneurodea (Polyneoptera: Orthopterida) from the Lower Permian Wellington Formation of Noble County, Oklahoma. *Journal of the Kansas Entomological Society*, 82(4):300–304.

Beckemeyer, R. J. and Engel, M. S. (2009). An enigmatic new genus of biarmohymenid from the early Permian Wellington Formation of Noble County, Oklahoma (Palaeodictyopterida: Diaphanopterodea). *Transactions of the Kansas Academy of Science*, 112(1/2):103–108.

Beckemeyer, R. J. and Hall, J. D. (2007). *Permopanorpa inaequalis* Tillyard, 1926 (Insecta: Holometabola: Panorpida: Permopanorpidae): A fossil mecopteroid newly reported for the Lower Permian Wellington Formation of Noble County, Oklahoma. *Transactions of the Kansas Academy of Science*, 110(1/2):23–29.

Bellamy, C. L. (1995). Buprestidae (Coleoptera) from amber deposits: a brief review and family switch. *Coleopterists Bulletin*, 42(2):175–177.

Bennett, D. J. and Engel, M. S. (2005). A primitive sapygid wasp in Burmese amber (Hymenoptera: Sapygidae). *Acta zoologica cracoviensia*, 48B(1-2):1–9.

Bennett, D. J. and Engel, M. S. (2006). A new moustache wasp in Dominican amber, with an account of apoid wasp evolution emphasizing Crabroninae (Hymenoptera: Crabronidae). *American Museum Novitates*, 3529:1–10.

Berger, H., Heiss, E., and Kerzhner, I. M. (2001). Removal of homonymy between Urostylidae Dallas, 1851 (Insecta, Heteroptera) and Urostylidae Bütschli, 1889 (Ciliophora, Hypotrichia). *Annalen des Naturhistorischen Museums in Wien*, 103B:301–302.

Béthoux, O. (2003). *Protophasma dumasii* Brogniart, 1879, a link between Orthoptera and the 'dictyopterid' orders? *Journal of Orthoptera Research*, 12(1):57–62.

Béthoux, O. (2005). Cnemidolestodea (Insecta): an ancient order reinstated. *Journal of Systematic Palaeontology*, 3(4):403–408.

Béthoux, O. (2006). Revision of *Cacurgus* Handlirsch, 1911, a basal Pennsylvanian Archaeorthoptera (Insecta: Neoptera). *Bulletin of the Peabody Museum of Natural History*, 47(1-2):29–35.

Béthoux, O. (2007a). Cladotypic taxonomy applied: titanopterans are orthopterans. *Arthropod Systematics & Phylogeny*, 65(2):135–156.

Béthoux, O. (2007b). Emptying the Paleozoic wastebasket for insects: member of a Carboniferous 'protorthopterous family' assigned to natural groups. *Alavesia*, 1:41–48.

Béthoux, O. (2007c). Ordinal assignment of the genus *Tococladus* carpenter 1996 (Insecta: Archaeorthoptera). *Alavesia*, 1:3.

Béthoux, O. (2008a). The insect fauna from the Permian of Lodève (Hérault, France): state of the art and perspectives. *Journal of Iberian Geology*, 34(1):109–113.

Béthoux, O. (2008b). Revision and phylogenetic affinities of the lobeattid species *bronsoni* Dana, 1864 and *silvatica* Laurentiaux & Laurentiaux-Vieira, 1980 (Pennsylvanian; *Archaeorthoptera*). *Arthropod Systematics & Phylogeny*, 66(2):145–163.

Béthoux, O. (2009). The earliest beetle identified. *Journal of Paleontology*, 83(6):931–937.

Béthoux, O., Beattie, R. G., and Nel, A. (2007a). Wing venation and relationships of the order Glosselytrodea (Insecta). *Alcheringa*, 31(3):285–296.

Béthoux, O. and Beckemeyer, R. J. (2007). New and rare insect species from the Wellington Formation (Orthoptera, Grylloblattodea; Lower Permian, USA). *Alavesia*, 1:49–61.

Béthoux, O. and Briggs, D. E. G. (2008). How *Gerarus* lost its head: stem-group Orthoptera and Paraneoptera revisited. *Systematic Entomology*, 33(3):529–547.

Béthoux, O., Galtier, J., and Nel, A. (2004a). Earliest evidence of insect endophytic oviposition. *Palaios*, 19(4):408–413.

Béthoux, O., Klass, K.-D., and Schneider, J. W. (2009). Tackling the Şprotoblattoidea problemŤ: Revision of *Protoblattinopsis stubblefieldi* (Dictyoptera; late Carboniferous). *European Journal of Entomology*, 106:145–152.

Béthoux, O., McBride, J., and Maul, C. (2004b). Surface laser scanning of fossil insect wings. *Palaeontology*, 47(1):13–19.

Béthoux, O. and Nel, A. (2002a). New data on Tcholmanvissiidae (Orthoptera; Permian). *Journal of Orthoptera Research*, 11(2):223–235.

Béthoux, O. and Nel, A. (2002b). Venation pattern and revision of Orthoptera *sensu nov.* and sister groups. phylogeny of Palaeozoic and Mesozoic Orthoptera *sensu nov. Zootaxa*, 96:1–88.

Béthoux, O. and Nel, A. (2003a). Révision de *Protagrion audouini* Brongniart, 1893, du Carbonifère supérieur (Palaeoptera). *Bulletin de la Société entomologique de France*, 108(3):237–244.

Béthoux, O. and Nel, A. (2003b). Revision of *Diaphanoptera* species and new diagnosis of Diaphanopteridae (Palaeoptera: Diaphanopterodea). *Journal of Paleontology*, 77(5):1016–1020.

Béthoux, O. and Nel, A. (2005). Some Palaeozoic 'Protorthoptera' are 'ancestral' orthopteroids: major wing braces as clues to a new split among the 'Protorthoptera' (Insecta). *Journal of Systematic Palaeontology*, 2 [for 2004](4):285–309.

Béthoux, O., Nel, A., Galtier, J., Lapeyrie, J., and Gand, G. (2003a). A new species of Tococladidae Carpenter, 1966 from the Permian of France (Insecta: Archaeorthoptera). *Geobios*, 36(3):275–283.

Béthoux, O., Nel, A., Gand, G., and Lapeyrie, J. (2001). *Surijoka lutevensis* nov. sp.: the first Glosselytrodea (Insecta) from the Upper Permian of France (Lodève Basin). *Geobios*, 34(4):405–413.

Béthoux, O., Nel, A., Gand, G., Lapeyrie, J., and Galtier, J. (2002a). Discovery of the genus *Iasvia* Zalessky, 1934 in the Upper Permian of France (Lodève basin) (Orthoptera, Ensifera, Oedischiidae). *Geobios*, 35(3):293–302.

Béthoux, O., Nel, A., and Lapeyrie, J. (2004c). The extinct order Caloneurodea (Insecta: Pterygota: Panorthoptera): wing venation, systematics and phylogenetic relationships. *Annales zoologici*, 54(2):289–318.

Béthoux, O., Nel, A., Lapeyrie, J., and Gand, G. (2003b). The Permostridulidae fam. n. (Panorthoptera), a new enigmatic insect family from the Upper Permian of France. *European Journal of Entomology*, 100:581–585.

Béthoux, O., Nel, A., Lapeyrie, J., and Gand, G. (2005). New data on Paleozoic grylloblattid insects (Neoptera). *Journal of Paleontology*, 79(1):125–138.

Béthoux, O., Nel, A., Lapeyrie, J., Gand, G., and Galtier, J. (2002b). *Raphogla rubra* gen. n., sp. n., the oldest representative of the clade of modern Ensifera (Orthoptera: Tettigoniidea, Gryllidae). *European Journal of Entomology*, 99(1):111–116.

Béthoux, O., Nel, A., Lapeyrie, J., Gand, G., and Galtier, J. (2003c). New Martynoviidae from the Permian of Southern France (Lodève basin) (Insecta: Palaeoptera: Diaphanopterodea). *Geobios*, 36:131–139.

Béthoux, O., Nel, A., Schneider, J. W., and Gand, G. (2007b). *Lodetiella magnifica* nov. gen. and nov. sp. (Insecta: Palaeodictyoptera; Permian), an extreme situation in wing morphology of palaeopterous insects. *Geobios*, 40(2):181–189.

Béthoux, O. and Schneider, J. W. (2009). Description of a hind wing of a new basal *Archaeorthoptera* (Mazon Creek, IL; Pennsylvanian). *Alavesia*, 3:81–85.

Béthoux, O. and Wieland, F. (2009). Evidence for carboniferous origin of the order mantodea (insecta: Dictyoptera) gained from forewing morphology. *Zoological Journal of the Linnean Society*, 156:79–113.

Beutel, R. G. and Baum, E. (2008). A longstanding entomological problem finally solved? Head morphology of *Nannochorista* (Mecoptera, Insecta) and possibly phylogenetic implications. *Journal of Zoological Systematics and Evolutionary Research*, 46(4):346–367.

Bitsch, J. and Nel, A. (1999). Morphology and classification of the extinct Archaeognatha and related taxa (Hexapoda). *Annales de la Société entomologique de France*, 35(1):17–29.

Blagoderov, V. and Grimaldi, D. A. (2004). Fossil Sciaroidea (Diptera) in Cretaceous ambers, exclusive of Cecidomyiidae, Sciaridae, and Keroplatidae. *American Museum Novitates*, 3433:1–76.

Blagoderov, V., Grimaldi, D. A., and Fraser, N. C. (2007). How time flies for flies: diverse Diptera from the Triassic of Virginia and early radiation of the order. *American Museum Novitates*, 3572:1–39.

Blagoderov, V. A. (1998). Fungus gnats of the tribes Gnoristini and Leiini (Diptera, Mycetophilidae) from the early Cretaceous of Transbaikalia. *Paleontological Journal*, 32(1):54–59.

Blagoderov, V. A. (1999). New Bibionomorpha from the Triassic of Australia and Jurassic of Central Asia with notes on Paraxymyiidae Rohdendorf (Insecta, Diptera). In Scoggin, M., editor, *AMBA projects AM/PFICM98/1.99: Proceedings of the First International Palaeoentomological Conference, Moscow 1998*, pages 11–15.

Blagoderov, V. A., Lukashevitch, E. D., and Mostovski, M. B. (2002). 2.2.1.3.4.4. Order Diptera Linné, 1758. The true flies (=Muscida Laicharting, 1781). In Rasnitsyn, A. P. and Quicke, D. L. J., editors, *History of Insects*, pages 227–240. Kluwer Academic Publishers, The Netherlands.

Blagoderov, V. V. and Martínez-Delclòs, X. (2001). Two new fungus gnats (Insecta, Diptera, Mycetophilidae) from the Lower Cretaceous of Spain. *Geobios*, 34(1):63–67.

Blank, S. M., Taeger, A., Liston, A. D., Smith, D. R., Rasnitsyn, A. P., Shinohara, A., Heidemaa, M., and Viitasaari, M. (2009). Studies toward a world catalog of Symphyta (Hymenoptera). *Zootaxa*, 2254:1–96.

Böcher, J. (1995). Palaeoentomology of the Kap København Formation, a Plio-Pleistocene sequence in Peary Land, North Greenland. *Meddelelser om Grønland, Geoscience*, 33:1–82.

Bolton, H. (1912). Insect-remains from the Midland and South-Eastern Coal Measures. *Quarterly Journal of the Geological Society*, 68(3):310–323.

Bolton, H. (1921). *A Monograph of the Fossil Insects of the British Coal Measures. Part I.* Palaeontographical Society, London.

Bonde, N., Andersen, S., Hald, N., and Jakobsen, S. L. (2008). *Danekrae - Danmarks bedste fossiler*. Gyldendal, Copenhagen.

Bordy, E. M., Bumby, A. J., Catuneanu, O., and Eriksson, P. G. (2009). Possible trace fossils of putative termite origin in the Lower Jurassic (Karoo Supergroup) of South Africa and Lesotho. *South African Journal of Science*, 105:356–362.

Borkent, A. (1997). Upper and Lower Cretaceous biting midges (Ceratopogonidae: Diptera) from Hungarian and Austrian amber and the Koonwarra fossil bed of Australia. *Stuttgarter Beiträge zur Naturkunde Serie B (Geologie und Paläontologie)*, 249:1–10.

Borkent, A. (2008). The frog-biting midges of the world (Corethrellidae: Diptera). *Zootaxa*, 1804:1–456.

Börner, C. (1904). Zur Systematik der Hexapoden. *Zoologischer Anzeiger*, 27:511–533.

Bouchard, P., Bousquet, Y., Davies, A. E., Alonso-Zarazaga, M. A., Lawrence, J. F., Lyal, C. H. C., Newton, A. F., Reid, C. A. M., Schmitt, M., Ślipiński, S. A., and Smith, A. B. T. (2011). Family-group names in Coleoptera (Insecta). *ZooKeys*, 88:1–972.

Bourgoin, T. and Lefèbvre, F. (2002). A new fossil Kinnaridae from Dominican amber (Hemiptera: Fulgoromorpha). *Annales zoologici*, 52(4):583–585.

Bourgoin, T. and Szwedo, J. (2008). The 'cixiid-like' fossil planthopper families. *Bulletin of Insectology*, 61(1):107–108.

Braby, M. F., Trueman, J. W. H., and Eastwood, R. (2005). When and where did troidine butterflies (Lepidoptera: Papilionidae) evolve? Phylogenetic and biogeographic evidence suggests an origin in remnant Gondwana in the late Cretaceous. *Invertebrate Systematics*, 19(2):113–143.

Brasero, N., Nel, A., and Michez, D. (2009). Insects from the early Eocene amber of Oise (France): diversity and palaeontological significance. *Denisia*, 26:41–52.

Brauckmann, C. (1991). Ein neuer Inseckten-Rest (Megasecoptera) aus dem Ober-Karbon von Osnabrück. *Osnabrücker naturwissenschafliche Mitteilungen*, 17:25–32.

Brauckmann, C. (1993). Notiz über Insekten-Reste aus dem Ober-Karbon in Spanien. *Jahresberichte des Naturwissenschaften Vereins in Wuppertal*, 46:115–119.

Brauckmann, C. (2005). Ausgewählte Arthropoden: Insecta, Arachnida, Xiphosura, Eurypterida, "Myriapoda", Arthropleurida und Trilobita. *Courier Forschungsinstitut Senckenberg*, 254:87–102.

Brauckmann, C., Arillo, A., and Ortuño, V. M. (2001). A new Geraridae (Insecta, hemipteroid stem assemblage) from the Upper Carboniferous of La Magdalena (León, northern Spain). *Boletín Geológico y Minero*, 112(2):57–62.

Brauckmann, C. and Hahn, G. (1980). Ein neuer Insektenfund aus dem Westfalium von Ibbenbüren (Westdeutschland). *Paläontologische Zeitschrift*, 54(3-4):301–312.

Brauckmann, C. and Herd, K. J. (2003). Insekten-Funde aus dem Westfalium D (Ober-Karbon) des Piesberges bei Osnabrück (Deutschland). Teil 1: Palaeoptera. *Osnabrücker naturwissenschafliche Mitteilungen*, 28 (for 2002):27–69.

Brauckmann, C. and Herd, K. J. (2006). Insekten-Funde aus dem Westfalium D (Ober-Karbon) des Piesberges bei Osnabrück (Deutschland). Teil 2: Neoptera. *Osnabrücker naturwissenschafliche Mitteilungen*, 30/31 (for 2005):19–65.

Brauckmann, C., Koch, L., and Kemper, M. (1985). Spinnentiere (Arachnida) und Insekten aus den Vorhalle-Schichten (Namurium B; Ober-Karbon) von Hagen-Vorhalle (West Deutschland). *Geologie und Paläontologie in Westfalen*, 3:1–131.

Brauckmann, C. and Schneider, J. (1996). Ein unter-karbonisches Insekt aus dem Raum Bitterfeld/Delitzsch (Pterygota, Arnsbergium, Deutschland). *Neues Jahrbuch für Geologie und Paläontologie, Monatshefte*, 1996(1):17–30.

Brauckmann, C., Schöllmann, L., and Sippel, W. (2003). Die fossilen Insekten, Spinnentiere und Eurypteriden von Hagen-Vorhalle. *Geologie und Paläontologie in Westfalen*, 59:5–89.

Bridges, C. A. (1994). *Catalogue of the family-group, genus-group and species-group names of the Odonata of the world (3rd edition)*. Urbana, Illinois, USA.

Britt, B. B., Scheetz, R. D., and Dangerfield, A. (2008). A suite of dermestid beetle traces on dinosaur bone from the Upper Jurassic Morrison Formation, Wyoming, USA. *Ichnos*, 15(2):59–71.

Brodsky, A. K. (1994). *The evolution of insect flight.* Oxford University Press.

Brongniart, C. (1885). Les insectes fossiles des terrains primaires. coup d'œil rapide sur la faune entomologique des terrains paléozoïques. *Bulletin de la Société des amis des sciences naturelles de Rouen*, 1885:50–68.

Brongniart, C. (1893). Recherches pour servir à l'histoire des insectes fossiles des temps primaires, précédées d'une étude sur la nervation des ailes des insectes. *Thèse présentée à la Fauclté des Sciences de Paris*, 821:495.

Brooks, D. R. and Evenhuis, N. L. (1995). Serendipidae Evenhuis, 1994 (Insecta: Diptera) and Serendipidae Brooks and Barriga, 1995 (Platyhelminthes: Eucestoda): proposed removal of homonymy. *Journal of Parasitology*, 81(5):762.

Brothers, D. J. (2003). The first fossil Ephutini (Hymenoptera: Mutillidae), a new species of *Ephuta* Say from Dominican amber. *Acta zoologica cracoviensia*, 46(suppl. - Fossil Insects):101–107.

Brothers, D. J. and Rasnitsyn, A. P. (2003). Diversity of Hymenoptera and other insects in the late Cretaceous (Turonian) deposits at Orapa, Botswana: a preliminary review. *African Entomology*, 11(2):221–226.

Brothers, D. J. and Rasnitsyn, A. P. (2008). A new genus and species of Euparagiinae from the late Cretaceous of southern Africa (Hymenoptera: Vespidae). *Alavesia*, 2:73–76.

Brues, C. T. and Melander, A. L. (1915). *Key to the families of North American insects. An introduction to the classification of insects.* Boston and Pullman.

Brullé, G. A. (1832). IV. classe Insectes. p63-345. In *Expédition Scientifique de Morée. Section des sciences physiques. Tome III. I. Partie. Zoologie. Deuxiéme Section. Des animaux articulés*, page 395. Lavrault, Paris.

Brunner von Wattenwyl, K. (1882). *Prodromus der europäischen orthoptèren.* Engelmann, Leipzig.

Brunner von Wattenwyl, K. (1893). Prodromus der europäischen orthoptèren. révision du sytème des Orthoptères. *Annali del Museo civico di storia naturale di Genoa*, 2(13):1–230.

Burmeister, H. C. C. (1838-1839). *Handbuch der Entomologie, 2 volumes.* Reimer, Berlin.

Bütschli, O. (1889). Suctoria. In Bronn, H. G., editor, *Klassen und Ordnungen des Thier-Teichs, Band 1, Protozoa*, pages 1842–1945. Winter, Leipzig.

Bybee, S. M., Ogden, T. H., Branham, M. A., and Whiting, M. F. (2008). Molecules, morphology and fossils: a comprehensive approach to odonate phylogeny and the evolution of the odonate wing. *Cladistics*, 24:477–514.

Cairncross, B., Anderson, J. M., and Anderson, H. M. (1995). Palaeoecology of the Triassic Molteno Formation, Karoo Basin, South Africa Ű sedimentological and palaeontological evidence. *South African Journal of Geology*, 98(4):452–478.

Carle, F. L. (1995). Evolution, taxonomy, and biogeography of ancient gondwanian libelluloides, with comments on anisopteroid evolution and phylogenetic systematics (anisoptera: Libelluloidea). *Odonatologica*, 24(4):383–424.

Carpenter, F. M. (1950). The Lower Permian insects of Kansas. Part 10. the order Protorthoptera: The family Liomopteridae and its relatives. *Proceedings of the American Academy of Arts and Sciences*, 78(4):187–219.

Carpenter, F. M. (1951). Studies on Carboniferous insects from Commentry, France: Part II. the Megasecoptera. *Journal of Paleontology*, 25(3):336–355.

Carpenter, F. M. (1963a). A megasecopteran from Upper Carboniferous strata in Spain. *Psyche*, 70:44–49.

Carpenter, F. M. (1963b). Studies on Carboniferous insects from Commentry, France: Part V. the genus *Diaphanoptera* and the order Diaphanopterodea. *Psyche*, 70:240–256.

Carpenter, F. M. (1986). Substitute names for some extinct genera of fossil insects. *Psyche*, 92 [for 1985]:575–583.

Carpenter, F. M. (1991). A substitute name for the extinct genus *Proberotha* Riek (Neuroptera). *Psyche*, 98(1):87.

Carpenter, F. M. (1992a). Studies of North American Carboniferous insects. 8. new Palaeodictyoptera from Kansas, U.S.A. *Psyche*, 99(2/3):141–146.

Carpenter, F. M. (1992b). Superclass Hexapoda. In *Treatise on Invertebrate Paleontology, Part R, Arthropoda 4 (3&4)*, pages xxi + 655. Boulder, C. O. and Lawrence, K. A.: Geological Society of America and University of Kansas Press.

Carpenter, F. M. (1997). 14A Insecta. In Shabica, C. W. and Hay, A. A., editors, *Richardson's Guide to The Fossil Fauna of Mazon Creek*, pages 184–193. Northeastern Illinois University.

Carvalho, J. C. M. (1985). Mirídeos neotropicais, CCLIII: descriçĩes de bivis gêneros e espécies da tribo Orthotylni Van Duzee (Hemiptera). *Revista Brasileira de Biologia*, 45(3):249–298.

Casasola González, J. A. (2006). Phylogenetic relationships of the genera of Epipsocetae (Psocoptera: Psocomorpha). *Zootaxa*, 1194:1–32.

Chandler, P. (2002). *Heterotricha* Loew and allied genera (Diptera : Sciaroidea): offshoots of the stem group of Mycetophilidae and or Sciaridae? *Annales de la Société entomologique de France (Nouvelle série)*, 38(1-2):101–144.

Chang, H.-L., Kirejtshuk, A. G., Ren, D., and Shih, C.-K. (2009). First fossil click beetles from the Middle Jurassic of Inner Mongolia, China (Coleoptera: Elateridae). *Annales zoologici*, 59(1):7–14.

Chen, S.-X. and Tan, J.-J. (1973). A new family of Coleoptera from the Lower Cretaceous of Kansu. *Acta Entomologica Sinica*, 16(2):169–178.

Christiansen, K. and Nascimbene, P. (2006). Collembola (Arthropoda, Hexapoda) from the mid Cretaceous of Myanmar (Burma). *Cretaceous Research*, 27:318–363.

Christiansen, K. and Pike, E. M. (2002). Cretaceous Collembola (Arthropoda, Hexapoda) from the Upper Cretaceous of Canada. *Cretaceous Research*, 23(2):165–188.

Cifuentes-Ruiz, P., Vršanský, P., Vega, F. J., Cevallos-Ferriz, S. R. S., González-Soriano, E., and Delgado de Jesús, C. R. (2006). Campanian terrestrial arthropods from the Cerro del Pueblo Formation, Difunta Group in northeastern Mexico. *Geologica Carpathica*, 57(5):347–354.

Clifford, E., Coram, R. A., Jarzembowski, E. A., and Ross, A. J. (1994). A supplement to the insect fauna from the Purbeck Group of Dorset. *Proceedings of the Dorset Natural History and Archaeological Society*, 115:143–146.

Coram, R. A. and Jarzembowski, E. A. (1999). New fossil flies (Insecta: Diptera) from the Purbeck Limestone Group (Lower Cretaceous, Berriasian) of Dorset, UK. *Cretaceous Research*, 20:853–861.

Coram, R. A., Jarzembowski, E. A., and Mostovski, M. B. (2000). Two rare eremoneuran flies (Diptera: Empididae and Opetiidae) from the Purbeck Limestone Group. *Paleontological Journal*, 34(Suppl. 3):S370–S373.

Coram, R. A. and Nel, A. (2009). A new petalurid dragonfly from the Lower Cretaceous of southern England (Odonata: Petalurida: ?Cretapetaluridae). *Palaeodiversity*, 2:205–208.

Costa, C., Vanin, S. A., Lawrence, J. F., Ide, S., and Branham, M. A. (2006). Review of the family Brachypsectridae (Coleoptera: Elateroidea). *Annals of the Entomological Society of America*, 99(3):409–432.

Dalgleish, R. C., Palma, R. L., Price, R. D., and Smith, V. S. (2006). Fossil lice (insecta: Phthiraptera) reconsidered. *Systematic Entomology*, 31(4):648–651.

Damgaard, J. (2008a). Evolution of the semi-aquatic bugs (Hemiptera: Heteroptera: Gerromorpha) with a re-interpretation of the fossil record. *Acta Entomologica Musei Nationalis Pragae*, 48(2):251–268.

Damgaard, J. (2008b). Phylogeny of the semiaquatic bugs (Hemiptera-Heteroptera, Gerromorpha). *Insect Systematics & Evolution*, 39(4):431–460.

Damgaard, J., Klass, K.-D., Picker, M. D., and Buder, G. (2008). Phylogeny of the heelwalkers (Insecta: Mantophasmatodea) based on mtDNA sequences, with evidence for additional taxa in South Africa. *Molecular Phylogenetics and Evolution*, 47(2):433–462.

de Geer, C. (1773). *Mémoires pour servir à l'histoire des insectes, v. 3.* Stockholm.

de Jong, R. (2007). Estimating time and space in the evolution of the Lepidoptera. *Tijdschrift voor Entomologie*, 150:319–346.

Delclòs, X., Arillo, A., Peñalver, E., Barrón, E., Soriano, C., López-Del-Valle, R., Bernárdez, E., Corral, C., and Ortuño, V. M. (2007). Fossiliferous amber deposits from the Cretaceous (Albian) of Spain. *Comptes Rendus Palevol*, 6(1-2):135–149.

Delclòs, X., Nel, A., Bechly, G., Dunlop, J. A., Engel, M. S., and Heads, S. W. (2008). The enigmatic Mesozoic insect taxon Chresmodidae (Polyneoptera): New palaeobiological and phylogenetic data, with the description of a new species from the Lower Cretaceous of Brazil. *Neues Jahrbuch für Geologie und Paläontologie, Abhandlungen*, 247(3):353–381.

Dikow, T. (2009). Phylogeny of Asilidae inferred from morphological characters of imagines (Insecta: Diptera: Brachycera: Asiloidea). *Bulletin of the American Museum of Natural History*, 319:1–175.

Dmitriev, V. U. and Zherikhin, V. V. (1988). Izmeneniya raznoobraziya semeistv nasekomykh po dannym metoda nakoplennykh poyavlenii. In Ponomarenko, A. G., editor, *Melovoi biotsenoticheskii krizis i evolyutsiya nasekomykh [The Cretaceous biocenotic crisis and evolution of insects]*, pages 208–215. Nauka, Moscow.

Engel, M. S. (2000). A new interpretation of the oldest fossil bee (Hymenoptera: Apidae). *American Museum Novitates*, 3296:1–11.

Engel, M. S. (2001). A monograph of the Baltic amber bees and evolution of the Apoidea (Hymenoptera). *Bulletin of the American Museum of Natural History*, 259:1–192.

Engel, M. S. (2002). The smallest snakefly (Raphidioptera: Mesoraphidiidae): a new species in Cretaceous amber from Myanmar, with a catalog of fossil snakeflies. *American Museum Novitates*, 3363:1–22.

Engel, M. S. (2003a). An anteonine wasp in Cenomanian-Albian amber from Myanmar (Hymenoptera: Dryinidae). *Journal of the Kansas Entomological Society*, 76(4):616–621.

Engel, M. S. (2003b). The earwigs of Kansas, with a key to genera north of Mexico (Insecta: Dermaptera). *Transactions of the Kansas Academy of Science*, 106(3/4):115–123.

Engel, M. S. (2004a). 10. Arthropods in Mexican amber p.175-186. In Bousquets, J. L., Morrone, J. J., Ordóñez, O. Y., and Fernández, I. V., editors, *Biodiversidad, taxonomía y biogeografía de artrópodos de México: hacia una síntesis de su conocimiento*, pages xvi+660. Universidad Nacional Autónoma de México.

Engel, M. S. (2004b). The alderflies of Kansas (Megaloptera: Sialidae). *Transaction of the Kansas Academy of Science*, 107(3):119–125.

Engel, M. S. (2004c). The dustywings in Cretaceous Burmese amber (Insecta: Neuroptera: Coniopterygidae). *Journal of Systematic Palaeontology*, 2(2):133–136.

Engel, M. S. (2005a). An Eocene ectoparasite of bees: The oldest definitive record of phoretic meloid triungulins (Coleoptera: Meloidae; Hymenoptera: Megachilidae). *Acta zoologica cracoviensia*, 48B(1-2):43–48.

Engel, M. S. (2005b). A new sawfly from the Triassic of Queensland, Australia (Hymenoptera: Xyelidae). *Memoirs of the Queensland Museum*, 51(2):558.

Engel, M. S. (2006a). A note of the relic silverfish *Tricholepidion gertschi* (Zygentoma). *Transactions of the Kansas Academy of Science*, 109(3/4):236–238.

Engel, M. S. (2006b). Two ensign wasps in cretaceous amber from new jersey and myanmar (hymenoptera: Evaniidae). *Polskie Pismo Entomologiczne*, 75(3):443–454.

Engel, M. S. (2008a). A new apterous *Zorotypus* in Miocene amber from the Dominican Republic (Zoraptera: Zorotypidae). *Acta Entomolgica Slovenica*, 16(2):127–136.

Engel, M. S. (2008b). A stem-group cimicid in mid-Cretaceous amber from Myanmar (Hemiptera: Cimicoidea). *Alavesia*, 2:233–237.

Engel, M. S. (2008c). The wasp family Rhopalosomatidae in mid-Cretaceous amber from Myanmar (Hymenoptera: Vespoidea). *Journal of the Kansas Entomological Society*, 81(3):168–174.

Engel, M. S. (2009a). A new Lower Permian bristletail from the Wellington Formation in Kansas (Archaeognatha: Dasyleptidae). *Transactions of the Kansas Academy of Science*, 112(1/2):40–44.

Engel, M. S. (2009b). A new termite bug in Miocene amber from the Dominican Republic (Hemiptera: Termitaphididae). *ZooKeys*, 25:61–68.

Engel, M. S. and Archibald, S. B. (2003). An early Eocene bee (Hymenoptera: Halictidae) from Quilchena, British Columbia. *The Canadian Entomologist*, 135:63–69.

Engel, M. S. and Grimaldi, D. A. (2004a). The first Mesozoic stephanid wasp (Hymenoptera: Stephanidae). *Journal of Paleontology*, 78(6):1192–1197.

Engel, M. S. and Grimaldi, D. A. (2004b). A primitive earwig in Cretaceous amber from Myanmar (Dermaptera: Pygidicranidae). *Journal of Paleontology*, 78(5):1018–1023.

Engel, M. S. and Grimaldi, D. A. (2005). Primitive new ants in Cretaceous amber from Myanmar, New Jersey, and Canada (Hymenoptera: Formicidae). *American Museum Novitates*, 3485:1–23.

Engel, M. S. and Grimaldi, D. A. (2006a). The earliest webspinners (Insecta: Embiodea). *American Museum Novitates*, 3514:1–15.

Engel, M. S. and Grimaldi, D. A. (2006b). The first Cretaceous sclerogibbid wasp (Hymenoptera: Sclerogibbidae). *American Museum Novitates*, 3515:1–7.

Engel, M. S. and Grimaldi, D. A. (2006c). The first Cretaceous spider wasp (Hymenoptera: Pompilidae). *Journal of the Kansas Entomological Society*, 79(4):359–368.

Engel, M. S. and Grimaldi, D. A. (2007a). Cretaceous Scolebythidae and the phylogeny of the family (Hymenoptera: Chrysidoidea). *American Museum Novitates*, 3568:1–16.

Engel, M. S. and Grimaldi, D. A. (2007b). The neuropterid fauna of Dominican and Mexican amber (Neuropterida: Megaloptera, Neuroptera). *American Museum Novitates*, 3587:1–58.

Engel, M. S. and Grimaldi, D. A. (2007c). New false fairy wasps in Cretaceous amber from New Jersey and Myanmar (Hymenoptera: Mymarommatoidea). *Transactions of the Kansas Academy of Science*, 110(3/4):159–168.

Engel, M. S. and Grimaldi, D. A. (2008a). Diverse Neuropterida in Cretaceous amber, with particular reference to the paleofauna of Myanmar (Insecta). *Nova Supplementa Entomologica*, 20:1–86.

Engel, M. S. and Grimaldi, D. A. (2008b). A jugular-horned beetle in Cretaceous amber from Myanmar (Coleoptera: Prostomidae). *Alavesia*, 2:215–218.

Engel, M. S. and Grimaldi, D. A. (2009). Diversity and phylogeny of the mesozoic wasp family stigmaphronidae (hymenoptera: Ceraphronoidea). *Denisia*, 26:53–68.

Engel, M. S., Grimaldi, D. A., and Krishna, K. (2007a). Primitive termites from the early Cretaceous of Asia (Isoptera). *Stuttgarter Beiträge zur Naturkunde Serie B (Geologie und Paläontologie)*, 371:1–32.

Engel, M. S., Grimaldi, D. A., and Krishna, K. (2007b). A synopsis of Baltic amber termites (Isoptera). *Stuttgarter Beiträge zur Naturkunde Serie B (Geologie und Paläontologie)*, 372:1–20.

Engel, M. S., Grimaldi, D. A., and Krishna, K. (2009a). Termites (Isoptera): their phylogeny, classification, and rise to ecological dominance. *American Museum Novitates*, 3650:1–27.

Engel, M. S. and Haas, F. (2007). Family-group names for earwigs (Dermaptera). *American Museum Novitates*, 3567:1–20.

Engel, M. S., Ortega-Blanco, J., and Bennett, D. J. (2009b). A remarkable tiphiiform wasp in mid-Cretaceous amber from Myanmar (Hymenoptera: Tiphiidae). *Transactions of the Kansas Academy of Science*, 112(1/2):1–6.

Engel, M. S. and Perkovsky, E. E. (2006). Psocoptera (Insecta) in Eocene Rovno amber (Ukraine). *Vestnik zoologii*, 40(2):175–179.

Etter, W. and Kuhn, O. (2000). An articulated dragonfly (Insecta, Odonata) from the Upper Liassic Posidonia Shale of northern Switzerland. *Palaeontology*, 43(5):967–977.

Evans, J. W. (1956). Palaeozoic and Mesozoic Hemiptera (Insecta). *Australian Journal of Zoology*, 4(2):165–258.

Evenhuis, N. L. (1994). *Catalogue of the fossil flies of the World.* Backhuys, Leiden.

Evenhuis, N. L. (2002). Catalog of the Mythicomyiidae of the world (Insecta: Diptera. *Bishop Museum Bulletin in Entomology*, 10:1–85.

Fabricius, J. C. (1793). *Entomologicae Systematica, v. 2.* Hafniae.

Fernández-Rubio, F. (1999). Fossil butterflies. Causes of their rarity and how they influence our knowledge of phylogeny and distribution of Zygaenini (Lepidoptera: Zygaenidae). *Boletin de la S.E.A.*, 26:521–532.

Fernández-Rubio, F. and Nel, A. (2000). *Neurosymploca*? *oligocenica* a new fossil species of Lepidoptera Zygaenoidea of the Oligocene of Céreste (Lubéron, France). *Boletin de la S.E.A.*, 27:7–16.

Fleck, G., Bechly, G., Martínez-Delclòs, X., Jarzembowski, E., Coram, R., and Nel, A. (2003). Phylogeny and classification of the Stenophlebioptera (Odonata: Epiproctophora). *Annales de la Société entomologique de France (Nouvelle série)*, 39(1):55–93.

Fleck, G., Bechly, G., Martínez-Delclòs, X., Jarzembowski, E. A., and Nel, A. (2004). A revision of the Upper Jurassic-Lower Cretaceous dragonfly family Tarsophlebiidae, with a discussion on the phylogenetic positions of the Tarsophlebiidae and Sieblosiidae (Insecta, Odonatoptera, Panodonata). *Geodiversitas*, 26(1):33–60.

Fleck, G. and Nel, A. (2003). Revision of the Mesozoic family Aeschnidiidae (Odonata: Anisoptera). *Zoologica*, 153:1–172.

Fleck, G., Nel, A., Bechly, G., Delclòs, X., Jarzembowski, E. A., and Coram, R. A. (2008). New Lower Cretaceous 'libelluloid' dragonflies (Insecta: Odonata: Cavilabiata) with notes about estimated divergence dates for this group. *Palaeodiversity*, 1:19–36.

Fleck, G., Nel, A., Bechly, G., and Martínez-Delclòs, X. (2001). Revision and phylogenetic affinities of the Jurassic Steleopteridae Handlirsch, 1906 (Odonata: Zygoptera). *Insect Systematics & Evolution*, 32:285–305.

Fleck, G., Nel, A., and Martínez-Delclòs, X. (1999). The oldest record of libellulid dragonflies from the Upper Cretaceous of Kazakhstan (Insecta: Odonata, Anisoptera). *Cretaceous Research*, 20:655–658.

Fleck, G., Waller, A., Serafin, J., and Nel, A. (2009). The oldest Calopterygidae in the Eocene Baltic amber (Odonata: Zygoptera). *Zootaxa*, 1985:52–56.

Foldi, I. (2005). Ground pearls: a generic revision of the Margarodidae *sensu stricto* (Hemiptera: Sternorrhyncha: Coccoidea). *Annales de la Société entomologique de France (Nouvelle série)*, 41(1):18–125.

Fujiyama, I. (1994). Two parasitic wasps from Aptian (Lower Cretaceous) Choshi amber, Chiba, Japan. *Natural History Research*, 3(1):1–5.

Gaimari, S. D. and Mostovski, M. B. (2000). *Burmapsilocephala cockerelli*, a new genus and species of Asiloidea (Diptera) from Burmese amber. *Bulletin of The Natural History Museum, Geology Series*, 56(1):43–45.

Gall, J.-C. and Grauvogel-Stamm, L. (2005). The early Middle Triassic 'Grès à Voltzia' Formation of eastern France: a model of environmental refugium. *Comptes Rendus Palevol*, 4(6-7):637–652.

Gáll, L. F. and Tiffney, B. H. (1983). A fossil noctuid moth egg from the late Cretaceous of eastern North America. *Science*, 219(4584):507–509.

Gao, T.-P., Ren, D., and Shih, C.-K. (2009). The first Xyelotomidae (Hymenoptera) from the Middle Jurassic in China. *Annals of the Entomological Society of America*, 102(4):588–596.

Garrouste, R., Nel, A., and Gand, G. (2009). New fossil arthropods (Notostraca and Insecta: Syntonopterida) in the continental Middle Permian of Provence (Bas-Argens Basin, France). *Comptes Rendus Palevol*, 8(1):49–57.

Geertsema, H., van Dijk, D. E., and van den Heever, J. A. (2002). Palaeozoic insects of southern Africa: a review. *Palaeontologia africana*, 38:19–25.

Gentilini, G. (2002). Fossil damselflies and dragonflies from the Eocene of Monte Bolca, Italy (Insecta: Odonata). *Studi e Ricerche sui Giaimenti Terziari di Bolca*, 9:7–22.

Gentilini, G., Korneyev, V. A., and Kameneva, E. P. (2006). Fossil tephritoid flies (diptera: Pallopteridae, ulidiidae, tephritidae) from the upper miocene of monte castellaro, italy, and a review of fossil european tephtitoids. *Instrumenta Biodiversitatis*, 7:85–104.

Gibson, G. A. P. (2008). Description of *Leptoomus janzeni*, n. gen. and n. sp. (Hymenoptera: Chalcidoidea) from Baltic amber, and discussion of its relationships and classification relative to Eupelmidae, Tanaostigmatidae and Encyrtidae. *Zootaxa*, 1730:1–26.

Gibson, G. A. P., Read, J., and Huber, J. T. (2007). Diversity, classification and higher relationships of Mymarommatoidea (Hymenoptera). *Journal of Hymenoptera Research*, 16(1):51–146.

Godunko, R. J. and Kłonowska-Olejnik, M. (2006). The first fossil representative of the genus *Analetris* Edmunds, 1972 (Insecta: Ephemeroptera: Acanthametropodidae) from the Eocene Baltic amber. *Annales zoologici*, 56(4):785–790.

Godunko, R. J. and Krzemiński, W. (2009). New fossil findings of the mayfly genera *Balticobaetisca* Staniczek & Bechly, 2002 (Ephemeroptera: Baetiscidae) and *Borinquena* Traver, 1938 (Leptophlebiidae: Atalophlebiinae). *Aquatic Insects*, 31(Supplement 1):125–136.

Godunko, R. J. and Neumann, C. (2006). Fossil mayfly collections of the Museum für Naturkunde, Humboldt University Berlin. I. *Electroletus soldani* gen. and sp. nov. (Ephemeroptera: Ameletidae) from the Eocene Baltic amber. *Annales zoologici*, 56(1):175–180.

Godunko, R. J., Neumann, C., and Krzemiński, W. (2008). Fossil mayfly collections of the Museum für Naturkunde, Humboldt University, Berlin. II. redescription of *Baltameletus oligocaenicus* Demoulin, 1968 with notes on Ameletidae McCafferty, 1991 (Insecta: Ephemeroptera) from the Eocene Baltic amber. *Annales zoologici*, 58(1):105–114.

Goldenberg, C. F. (1877). *Fauna Saraepontana fossilis. Die fossilen Thiere aus der Steinkohlenformation von Saarbrücken, Part II.* Möllinger: Saarbrücken.

Golub, V. B. and Popov, Y. A. (2000). A remarkable fossil lace bug from Upper Cretaceous New Jersey amber (Heteroptera: Tingoidea, Vianaididae), with some phyogenetic commentary. In Grimaldi, D. A., editor, *Studies on Fossils in Amber, with Particular Reference to the Cretaceous of New Jersey*, pages 231–239. Backhuys Publishers, Leiden, The Netherlands.

Golub, V. B. and Popov, Y. A. (2003). The new fossil genus of Vianaididae (Heteroptera: Tingoidea) from the Cretaceous amber of New Jersey; evolution of the family in the Late Cretaceous. *Acta zoologica cracoviensia*, 46(suppl.- Fossil Insects):109–116.

Golub, V. B. and Popov, Y. A. (2008). A new species of Tingidae (Insecta: Hemiptera: Heteroptera) from the Lower Cretaceous of Transbaikalia. *Paleontological Journal*, 42(1):86–89.

Gorjunova, R. V. (1988). New Carboniferous bryozoans of the Gobi Altai. *Sovmestnaya Sovetsko-Mongolskaya Paleontologischeskaya Ekspeditsiya Trudy*, 33:10–23.

Gorokhov, A. V. (1985). Mesozoic crickets (Orthoptera, Grylloidea) of Asia. *Paleontological Journal*, 19(2):56–66.

Gorokhov, A. V. (1986). Triassic grasshoppers of the superfamily Hagloidea (Orthoptera). *Trudy Zoologicheskogo Instituta*, 143:65–100. In Russian.

Gorokhov, A. V. (1987a). New fossil Orthopterans of the families Adumbratomorphidae fam. n., Pruvostitidae and Proparagryllacrididae (Orthoptera, Ensifera) from Permian and Triassic deposits of the USSR [in Russian]. *Vestnik zoologii*, 1987(4):20–28.

Gorokhov, A. V. (1987b). New fossil Orthopterans of the families Bintoniellidae, Mesoedischiidae fam. n. and Pseudelcanidae fam. n. (Orthoptera, Ensifera) from Permian and Triassic deposits of the USSR [in Russian]. *Vestnik zoologii*, 1987(1):18–23.

Gorokhov, A. V. (1988a). Classification and phylogeny of grasshoppers (Gryllida = Orthoptera, Tettigonioidea) [in Russian]. In Ponomarenko, A. G., editor, *The Cretaceous Biocenotic Crisis and the Evolution of Insects*, pages 145–190. Nauka, Moscow.

Gorokhov, A. V. (1988b). Grasshoppers of the superfamily Hagloidea (Orthoptera) from the Lower and Middle Jurassic [in Russian]. *Paleontologicheskii Zhurnal*, 1988(2):54–66.

Gorokhov, A. V. (1988c). On the classification of fossil grasshoppers of the superfamily Phasmomimoidea (Orthoptera) with descriptions of new taxa [in Russian]. *Trudy Paleontologicheskogo instituta Akademiia nauk SSSR*, 178:32–44.

Gorokhov, A. V. (1994). New data on Triassic Orthoptera from Middle Asia. *Zoosystematica Rossica*, 3(1):53–54.

Gorokhov, A. V. (1995a). On the system and evolution of the order Orthoptera. *Zoologicheskii Zhurnal*, 74(10):39–45.

Gorokhov, A. V. (1995b). System and evolution of the suborder Ensifere (Orthoptera), Part 1. *Trudy Paleontologicheskogo instituta Akademiia nauk SSSR*, 260(1):1–224.

Gorokhov, A. V. (2000). Phasmomimidae: are they Orthoptera or Phasmatoptera? *Paleontological Journal*, 34(3):295–300.

Gorokhov, A. V. (2005a). Review of Triassic Orthoptera with descriptions of new and little known taxa: Part 1. *Paleontological Journal*, 39(2):68–76.

Gorokhov, A. V. (2005b). Review of Triassic Orthoptera with descriptions of new and little known taxa: Part 2. *Paleontological Journal*, 39(3):272–279.

Gorokhov, A. V. (2006). New and little known orthopteroid insects (Polyneoptera) from fossil resins: Communication 1. *Paleontological Journal*, 40(6):646–654.

Gorokhov, A. V. (2007). The first representative of the suborder Mesotitanina from the Paleozoic and notes on the system and evolution of the order Titanoptera (Insecta: Polyneoptera). *Paleontological Journal*, 41(6):621–625.

Gorokhov, A. V., Jarzembowski, E. A., and Coram, R. A. (2006). Grasshoppers and crickets (Insecta: Orthoptera) from the Lower Cretaceous of southern England. *Cretaceous Research*, 27(5):641–662.

Gorokhov, A. V. and Rasnitsyn, A. P. (2002). 2.2.2.3. Superorder Gryllidea Laicharting, 1781 (=Orthopteroidea Handlirsch, 1903). In Rasnitsyn, A. P. and Quicke, D. L. J., editors, *History of Insects*, pages 293–303. Kluwer Academic Publishers, The Netherlands.

Gratshev, V. G. and Zherikhin, V. V. (1994). New fossil mantids (Insecta, Mantida). *Paleontological Journal*, 27 (for 1993)(1A):148–165.

Gratshev, V. G. and Zherikhin, V. V. (2003). The fossil record of weevils and related beetle families (Coleoptera, Curculionoidea). *Acta zoologica cracoviensia*, 46(suppl. - Fossil Insects):129–138.

Grazia, J., Schuh, R. T., and Wheeler, W. C. (2008). Phylogenetic relationships of family groups in Pentatomoidea based on morphology and DNA sequences (Insecta: Heteroptera). *Cladistics*, 24(6):932–976.

Greenwood, D. R., Archibald, S. B., Mathewes, R. W., and Moss, P. T. (2005). Fossil biotas from the Okanagan Highlands, southern British Columbia and northern Washington State: climates and ecosystems across an Eocene landscape. *Canadian Journal of Earth Sciences*, 42(2):167–185.

Grimaldi, D., Zhang, J.-F., Fraser, N. C., and Rasnitsyn, A. P. (2005a). Revision of the bizarre Mesozoic scorpionflies in the Pseudopolycentropodidae (Mecopteroidea). *Insect Systematics & Evolution*, 36(4):443–458.

Grimaldi, D. A. (1999). The co-radiations of pollinating insects and angiosperms in the Cretaceous. *Annals of the Missouri Botanical Garden*, 86(2):373–406.

Grimaldi, D. A. (2003a). First amber fossils of the extinct family Protopsyllidiidae, and their phylogenetic significance among Hemiptera. *Insect Systematics & Evolution*, 34(3):329–344.

Grimaldi, D. A. (2003b). A revision of Cretaceous mantises and their relationships, including new taxa (Insecta: Dictyoptera: Mantodea). *American Museum Novitates*, 3412:1–47.

Grimaldi, D. A. (2007). 11.7 Mantodea: praying mantises. In Martill, D. M., Bechly, G., and Loveridge, R. F., editors, *The Crato Fossil Beds of Brazil: Window into an Ancient World*, pages 234–238. Cambridge University Press.

Grimaldi, D. A. (2008). A stalk-eyed ephydroid fly from the Eocene (Diptera: Ephydroidea: Camillidae). *Proceedings of the Entomological Society of Washington*, 110(3):543–550.

Grimaldi, D. A., Amorim, D. S., and Blagoderov, V. (2003). The Mesozoic family Archizelmiridae (Diptera: Insecta). *Journal of Paleontology*, 77(2):368–381.

Grimaldi, D. A. and Arillo, A. (2008). The Tethepomyiidae, a new family of enigmatic Cretaceous Diptera. *Alavesia*, 2:259–265.

Grimaldi, D. A. and Cumming, J. (1999). Brachyceran Diptera in Cretaceous ambers and Mesozoic diversification of the Eremoneura. *Bulletin of the American Museum of Natural History*, 239:1–124.

Grimaldi, D. A., Cumming, J. M., and Arillo, A. (2009). Chimeromyiidae, a new family of eremoneuran Diptera from the Cretaceous. *Zootaxa*, 2078:34–54.

Grimaldi, D. A. and Engel, M. S. (2005). *Evolution of the Insects.* Cambridge University Press.

Grimaldi, D. A. and Engel, M. S. (2006a). Extralimital fossils of the "Gondwanan" family Sphaeropsocidae (Insecta: Psocodea). *American Museum Novitates*, 3523:1–18.

Grimaldi, D. A. and Engel, M. S. (2006b). Fossil Liposcelididae and the lice ages (Insecta: Psocodea). *Proceedings of the Royal Society, B*, 273(1586):625–633.

Grimaldi, D. A. and Engel, M. S. (2008a). A termite bug in early Miocene amber of the Dominican Republic (Hemiptera: Termitaphididae). *American Museum Novitates*, 3619:1–10.

Grimaldi, D. A. and Engel, M. S. (2008b). An unusual, primitive Piesmatidae (Insecta: Heteroptera) in Cretaceous amber from Myanmar (Burma). *American Museum Novitates*, 3611:1–17.

Grimaldi, D. A., Engel, M. S., and Krishna, K. (2008). The species of isoptera (insecta) from the early cretaceous crato formation: a revision. *American Museum Novitates*, 3626:1–30.

Grimaldi, D. A., Engel, M. S., and Nascimbene, P. C. (2002). Fossiliferous Cretaceous amber from Myanmar (Burma): its rediscovery, biotic diversity, and paleontological significance. *American Museum Novitates*, 3361:1–71.

Grimaldi, D. A., Kathirithamby, J., and Schawaroch, V. (2005b). Strepsiptera and triungula in Cretaceous amber. *Insect Systematics & Evolution*, 36(1):1–20.

Grimaldi, D. A., Shmakov, A., and Fraser, N. C. (2004). Mesozoic thrips and early evolution of the order Thysanoptera (Insecta). *Journal of Paleontology*, 78(5):941–952.

Grimaldi, D. A. and Triplehorn, D. M. (2008). Insects from the Upper Miocene Grubstake Formation of Alaska. *American Museum Novitates*, 3612:1–19.

Gumovsky, A. V. (2001). The status of some genera allied to *Chrysonotomyia* and *Closterocerus* (Hymenoptera: Eulophidae, Entedoninae), with description of a new species from Dominican amber. *Phegea*, 29(4):125–141.

Gumovsky, A. V. and Perkovsky, E. E. (2005). Taxonomic notes on Tetracampidae (Hymenoptera: Chalcidoidea) with description of a new fossil species of *Dipricocampe* from Rovno amber. *Entomological Problems*, 35(2):123–130.

Gutiérrez, P. R., Muzón, J., and Limarino, C. O. (2000). The earliest late Carboniferous winged insect (Insecta, Protodonata) from Argentina: geographical and stratigraphical location. *Ameghiniana*, 37(3):375–378.

Haas, F. (2007). 11.6 Dermaptera: earwigs. In Martill, D. M., Bechly, G., and Loveridge, R. F., editors, *The Crato Fossil Beds of Brazil: Window into an Ancient World*, pages 222–234. Cambridge University Press.

Haeckel, E. H. P. A. (1896). *Systematische Phylogenie. Entwurf eines natürlichen Systema der Organismen auf Grund ihrer Stammesgeschichte. Zweiter Theil: Systematische Phylogenie der Wirbellosen Thiere (Invertebrata).* Reimer, Berlin.

Haenni, J.-P. (2003). Fossil Diptera in Baltic amber: the collection of the Muséum d'histoire naturelle Neuchâtel. *Acta zoologica cracoviensia*, 46(suppl.- Fossil Insects):407–410.

Haliday, A. H. (1836). An Epitome of the British genera, in the order Thysanoptera, with indications of a few species. *Entomological Magazine*, 3:439–451.

Hall, J. P., Robbins, R. K., and Harvey, D. J. (2004). Extinction and biogeography in the Caribbean: new evidence from a fossil riodinid butterfly in Dominican amber. *Proceedings of the Royal Society of London, B*, 271(1541):797–801.

Hamilton, K. G. A. (1990). Chapter 6. Homoptera. In Grimaldi, D. A., editor, *Insects from the Santana Formation, Lower Cretaceous of Brazil*, volume 195, pages 82–122. Bulletin of the American Museum of Natural History.

Hamilton, K. G. A. (1992). Lower Cretaceous Homoptera from the Koonwarra Fossil Bed in Australia, with a new superfamily and synopsis of Mesozoic Homoptera. *Annales of the Entomological Society of America*, 85:423–430.

Handlirsch, A. (1906). Revision of American Paleozoic insects. *Proceedings of the United States National Museum*, 29:661–820.

Handlirsch, A. (1906-1908). *Die fossilen Insekten und die Phylogenie der rezenten Formen. Ein Handbuch für Paläontologen und Zoologen.* Engelmann, Leipzig.

Handlirsch, A. (1911). New Paleozoic insects from the vicinity of Mazon Creek, Illinois. *American Journal of Science (series 4)*, 31:297–326.

Handlirsch, A. (1919). Revision der paläozoischen Insekten. *Denkschriften der Kaiserlichen Akademie der Wissenschaften, Mathematisch-Naturwissenschaftliche Klasse*, 96:511–592.

Handlirsch, A. (1937). Neue Untersuchungen über die fossilen Insekten mit Ergänzungen und Nachträgen sowie Ausblicken auf phylogenetische, palaeogeographische und allgemein biologische Probleme. I Teil. *Annalen des Naturhistorischen Museums in Wien*, 48:1–140.

Handlirsch, A. (1939). Neue Untersuchungen über die fossilen Insekten. II. Teil. *Annalen des Naturhistorischen Museums in Wien*, 49:1–240.

Harbach, R. E. (2007). The Culicidae (Diptera): a review of taxonomy, classification and phylogeny. *Zootaxa*, 1668:591–638.

Harris, A. C. and Raine, J. I. (2002). A sclerite from a late Cretaceous moth (Insecta: Lepidoptera) from Rakaia Gorge, Canterbury, New Zealand. *Journal of the Royal Society of New Zealand*, 32(3):457–462.

Hauser, M. and Winterton, S. L. (2007). A new fossil genus of small-headed flies (Diptera: Acroceridae: Philopotinae) from Baltic amber. *Annals of the Entomological Society of America*, 100(2):152–156.

Hava, J., Prokop, J., and Herrmann, A. (2006). New fossil dermestid beetles (Coleoptera: Dermestidae) from the Baltic amber. *Acta Societatis Zoologicae Bohemicae*, 69:281–287.

Heads, S. W. (2008a). The first fossil Proscopiidae (Insecta, Orthoptera, Eumastacoidea) with comments on the historical biogeography and evolution of the family. *Palaeontology*, 51(2):499–507.

Heads, S. W. (2008b). A new species of *Yuripopovia* (Coleorrhyncha: Progonocimicidae) from the early Cretaceous of the Isle of Wight. *British Journal of Entomology and Natural History*, 21:247–253.

Heads, S. W. (2009a). New pygmy grasshoppers in Miocene amber from the Dominican Republic (Orthoptera: Tetrigidae). *Denisia*, 26:69–74.

Heads, S. W. (2009b). A new pygmy mole cricket in Cretaceous amber from Burma (Orthoptera: Tridactylidae). *Denisia*, 26:75–82.

Heads, S. W. and Martins-Neto, R. G. (2007). 11.11 Orthopterida: grasshoppers, crickets, locusts and stick insects. In Martill, D. M., Bechly, G., and Loveridge, R. F., editors, *The Crato Fossil Beds of Brazil: Window into an Ancient World*, pages 265–283. Cambridge University Press.

Heie, O. E. (1985). Fossil aphids. A catalogue of fossil aphids, with comments on systematics and evolution. In *Evolution and biosystematics of aphids. Proceedings of the International Aphidological Symposium at Jablona, 5-11 April 1951*, pages 101–131. Polska Akademia Nauk, Instytut Zoologii, Warzawa.

Heie, O. E. (1987). Palaeontology and phylogeny. In Minks, A. K. and Harrewijn, P., editors, *Aphids: their biology, natural enemies and control, Volume A*, pages 367–391. Elsevier Academic Press, Amsterdam.

Heie, O. E. (1999). Aphids of the past (Hemiptera, Sternorrhyncha). In Scoggin, M., editor, *AMBA projects AM/PFICM98/1.99: Proceedings of the First International Palaeoentomological Conference, Moscow 1998*, pages 49–55.

Heie, O. E. and Azar, D. (2000). Two new species of aphids found in Lebanese amber and a revision of the family Tajmyraphididae Kononova, 1975 (Hemiptera: Sternorrhyncha). *Annals of the Entomological Society of America*, 93(6):1222–1225.

Heie, O. E. and Pike, E. M. (1992). New aphids in Cretaceous amber from Alberta (Insecta, Homoptera). *The Canadian Entomologist*, 124(6):1027–1053.

Heie, O. E. and Wegierek, P. (1998). A list of fossil aphids (Homoptera: Aphidinea). *Annals of the Upper Silesian Museum (Entomology)*, 8-9:159–192.

Heraty, J. M. and Darling, D. C. (2009). Fossil Eucharitidae and Perilampidae (Hymenoptera: Chalcidoidea) from Baltic amber. *Zootaxa*, 2306:1–16.

Herczek, A. and Popov, Y. A. (2001). Redescription of the oldest plant bugs from the Upper Jurassic of the southern Kazakhstan (Heteroptera: Cimicomorpha, Miridae). *Annals of the Upper Silesian Museum (Entomology)*, 10-11:121–128.

Hong, Y.-C. (1980a). The discovery of Late Palaeozoic insecta in Shanxi Province. *Geological Review*, 26(2):89–95.

Hong, Y.-C. (1980b). Granulidae, a new family of Homoptera from the Middle Triassic of Tongchuan, Shaanxi Province. *Acta Zootaxonomica Sinica*, 5(1):63–70. in Chinese with English abstract.

Hong, Y.-C. (1983). *Middle Jurassic Fossil Insects in North China*. Geological Publishing House, Beijing.

Hong, Y.-C. (1984). Curvicubitidae fam. nov. (Lepidoptera? Insecta) from Middle Triassic of Shaanxi. *Acta Palaeontologica Sinica*, 2(6):782–785. in Chinese with English summary.

Hong, Y.-C. (1985). New fossil genera and species of Shanxi Formation in Xishan of Taiyuan. *Entomotaxonomia*, 7(2):83–91.

Hong, Y.-C. (1998a). Establishment of fossil entomofaunas and their evolutionary succession in north China. *Entomologia Sinica*, 5(4):283–300.

Hong, Y.-C. (1998b). A new early Cretaceous beetle family - Magnocoleidae fam. nov. (Insecta: Coleoptera) in Hebei Province. *Geoscience*, 12(1):40–49.

Hong, Y.-C. (2002a). *Amber insects of China*. Scientific and Technological Publishing House, Beijing.

Hong, Y.-C. (2002b). *Atlas of Amber Insects of China*. Scientific and Technological Publishing House, Henan.

Hong, Y.-C. (2003). *Hebeigramma* nom. nov., a new name for *Mesogramma* Hong, 1984 (Caloneurodea) from the Lower Cretaceous of Hebei Province, China. *Geological Bulletin of China*, 22(9):686–687.

Hong, Y.-C. (2006). First discovery of fossil Protomecoptera in the Tongchuan region, Shaanxi, China. *Geological Bulletin of China*, 25(5):560–564.

Hong, Y.-C. (2007a). Discovery of the fossil glosselytrods (Insecta: Glosselytrodea) from Shaanxi, China. *Acta Entomolgica Sinica*, 50(3):271–280.

Hong, Y.-C. (2007b). Mid Triassic new genera and species of Mesopanorpodidae (Insecta, Mecoptera) from Shaanxi, China. *Acta Zootaxonomica Sinica*, 32(2):261–267.

Hong, Y.-C. (2009a). First discovery of Midtriassic order Miomoptera (Insecta) in China. *Geological Bulletin of China*, 28(1):11–15.

Hong, Y.-C. (2009b). Mid Triassic new genera and species of Orthophlebiidae and Neorthophlebiidae (Insecta, Mecoptera) from Shaanxi, China. *Acta Zootaxonomica Sinica*, 34(3):423–427.

Hong, Y.-C. and Guo, X.-R. (2003). Two new Middle Triassic genera and species of Mesopanorpodidae from the Shaanxi (Insecta, Mecoptera). *Acta Zootaxonomica Sinica*, 28(4):715–720.

Hong, Y.-C. and Li, Z. (2007). Discovery of the oldest fossil Meropeidae (Insecta, Mecoptera) from Shaanxi, China. *Acta Zootaxonomica Sinica*, 32(4):875–880.

Hong, Y.-C. and Li, Z.-Y. (2004). A new early Cretaceous family from Liupanshan, Ningxia, China (Insecta, Trichoptera). *Acta Zootaxonomica Sinica*, 29(2):224–233.

Hong, Y.-C. and Wang, W.-L. (1990). Fossil insects from the Laiyang Basin, Shandong Province. In *The Stratigraphy and Palaeontology of Laiyang Basin, Shandong Province*, pages 44–189. Shandong Bureau of Geology and Mineral Resources.

Hong, Y.-C. and Zhang, Z.-J. (2007). Reclassification of fossil Orthophlebiidae (Insecta: Mecoptera). *Entomotaxonomia*, 29(1):26–36.

Hong, Y.-C., Zhang, Z.-J., Guo, X.-R., and Heie, O. E. (2009). A new species representing the oldest aphid (Hemiptera, Aphidomorpha) from the Middle Triassic of China. *Journal of Paleontology*, 83(5):826–831.

Hörnschemeyer, T. (1999). Fossil insects from the Lower Permian of Nierdermoschel [sic] (Germany). In Scoggin, M., editor, *AMBA projects AM/PFICM98/1.99: Proceedings of the First International Palaeoentomological Conference, Moscow 1998*, pages 57–60.

Hörnschemeyer, T. (2005). *Archostemata Kolbe, 1908*, pages 29–42. Morphology and systematics (Archostemata, Adephaga, Myxophaga, Polyphaga partim). Handbuch der Zoologie, 4: Arthropoda, 2. Insecta. Coleoptera, beetles, Part 38, Volume 1.

Hörnschemeyer, T. and Stapf, H. (1999). Die Insektentaphozönose von Niedermoschel (Asselian, unt. Perm; Deutschland). *Terra Nostra. Schriften der Alfred-Wegener-Stiftung*, 99/8:98.

Hörnschemeyer, T. and Stapf, H. (2001). Review of Blattinopsidae (Protorthoptera) with description of new species from the Lower Permian of Niedermoschel (Germany). *Neues Jahrbuch für Geologie und Paläontologie, Abhandlungen*, 221(3):81–132.

Huang, D.-Y. and Nel, A. (2007a). A new Middle Jurassic "grylloblattodean" family from China (Insecta: Juraperlidae fam. n.). *European Journal of Entomology*, 104(4):837–840.

Huang, D.-Y. and Nel, A. (2007b). Oldest 'libelluloid' dragonfly from the Middle Jurassic of China (Odonata: Anisoptera: Cavilabiata). *Neues Jahrbuch für Geologie und Paläontologie, Abhandlungen*, 246(1):63–68.

Huang, D.-Y. and Nel, A. (2008). A new Middle Jurassic aphid family (Insecta: Hemiptera: Sternorrhyncha: Sinojuraphididae fam. nov.) from Inner Mongolia, China. *Palaeontology*, 51(3):715–719.

Huang, D.-Y. and Nel, A. (2009a). The first Chinese Tarsophlebiidae from the Lower Cretaceous Yixian Formation, with morphological and phylogenetic implications (Odonatoptera: Panodonata). *Cretaceous Research*, 30(2):429–433.

Huang, D.-Y. and Nel, A. (2009b). Oldest webspinners from the Middle Jurassic of Inner Mongolia, China (Insecta: Embiodea). *Zoological Journal of the Linnean Society*, 156(4):889–895.

Huang, D.-Y., Nel, A., Azar, D., and Nel, P. (2008a). Phylogenetic relationships of the Mesozoic paraneopteran family Archipsyllidae (Insecta: Psocodea). *Geobios*, 41(4):461–464.

Huang, D.-Y., Nel, A., and Lin, Q.-B. (2003). A new genus and species of aeshnopteran dragonfly from the Lower Cretaceous of China. *Cretaceous Research*, 24(2):141–147.

Huang, D.-Y., Nel, A., Lin, Q.-B., and Dong, F.-B. (2007a). The first Glosselytrodea (Insecta) from the latest Middle Permian of Anhui Province, China. *Bulletin de la Société entomologique de France*, 112(2):179–182.

Huang, D.-Y., Nel, A., and Petrulevičius, J. F. (2008b). New evolutionary evidence of Grylloblattida from the Middle Jurassic of Inner Mongolia, north-east China (Insecta, Polyneoptera). *Zoological Journal of the Linnean Society*, 152(1):17–24.

Huang, D.-Y., Nel, A., Zompro, O., and Waller, A. (2008c). Mantophasmatodea now in the Jurassic. *Naturwissenschaften*, 95:947–952.

Huang, J.-D., Ren, D., and Sun, J.-H. (2007b). Progress in the study of Ephemeroptera (mayfly) fossils. *Acta Zootaxonomica Sinica*, 32(2):391–404.

Hubbard, M. D. (1987). Ephemeroptera. *Fossilium Catalogus 1: Animalia*, 129:1–99.

Huber, J. T. (2005). The gender and derivation of genus-group names in Mymaridae and Mymarommatidae (Hymenoptera). *Acta Societatis Zoologicae Bohemicae*, 69:167–183.

Huguet, A., Nel, A., Martínez-Delclòs, X., Bechly, G., and Martins-Neto, R. (2002). Preliminary phylogenetic analysis of the Protanisoptera (Insecta: Odonatoptera). *Geobios*, 35(5):537–560.

Hyatt, A. and Arms, J. M. (1890). *Guides for Science-Teaching, no. 8: Insecta.* Boston.

Ivanov, V. D. (1992). A new family of caddis flies (Insecta, Trichoptera) from the Permian of the middle Urals. *Paleontological Journal*, 26(4):36–41.

Ivanov, V. D. (2006). Larvae of caddisflies (Insecta: Trichoptera) from the Mesozoic of Siberia. *Paleontological Journal*, 40(2):178–189.

Ivanov, V. D. and Melnitsky, S. I. (2006). The morphology of *Dajella tenera* (Trichoptera, Glossosomatidae): taxonomic status and evidence for the pheromone communication in the Mesozoic. *Entomological Review*, 86(5):568–575.

Ivanov, V. D. and Sukatsheva, I. D. (2002). 2.2.1.3.4.2. Order Trichoptera Kirby, 1813. The caddisflies (=Phryganeida Latreille, 1810). In Rasnitsyn, A. P. and Quicke, D. L. J., editors, *History of Insects*, pages 199–220. Kluwer Academic Publishers, The Netherlands.

Jacobus, L. M. and McCafferty, W. P. (2006). Reevaluation of the phylogeny of the Ephemeroptera infraorder Pannota (Furcatergalia), with adjustments to higher classification. *Transactions of the American Entomological Society*, 132(1/2):81–90.

Jacobus, L. M. and McCafferty, W. P. (2008). Revision of Ephemerellidae genera (Ephemeroptera). *Transactions of the American Entomological Society*, 134(1/2):185–274.

Jarzembowski, E. A. (1990). Early Cretaceous zygopteroids of southern England, with the description of *Cretacoenagrion alleni* gen. nov., spec. nov. (Zygoptera: Coenagrionidae; "Anisozygoptera": Tarsophlebiidae, Euthemistidae). *Odonatologica*, 19(1):27–37.

Jarzembowski, E. A. (1992). A provisional checklist of fossil insects from the Purbeck Beds of Dorset. *Proceedings of the Dorset Natural History and Archaeological Society*, 114:175–179.

Jarzembowski, E. A. (1995). Fossil caddisflies (Insecta: Trichoptera) from the early Cretaceous of southern England. *Cretaceous Research*, 16:695–703.

Jarzembowski, E. A. (1999). Chapter 10. Arthropods 2: Insects p.149-160. In Swift, A. and Martill, D. M., editors, *Fossils of the Rhaetian Penarth Group*, page 312. The Palaeontological Association, London.

Jarzembowski, E. A. and Coram, R. (1996). New fossil records from the Purbeck of Dorset and the Wealden of the Weald. *Proceedings of the Dorset Natural History and Archaeological Society*, 1996:119–124.

Jarzembowski, E. A., Martínez-Delclòs, X., Bechly, G., Nel, A., Coram, R., and Escuillié, F. (1998). The Mesozoic non-calopterygoid Zygoptera: description of new genera and species from the Lower Cretaceous of England and Brazil and their phylogenetic significance (Odonata, Zygoptera, Coenagrionoidea, Hemiphlebioidea, Lestoidea). *Cretaceous Research*, 19(3-4):403–444.

Jarzembowski, E. A. and Nel, A. (1996). New fossil dragonflies from the Lower Cretaceous of SE England and the phylogeny of the superfamily Libelluloidea (Insecta: Odonata). *Cretaceous Research*, 17:67–85.

Jarzembowski, E. A. and Nel, A. (2002). The earliest damselfly-like insect and the origin of modern dragonflies (Insecta: Odonatoptera: Protozygoptera). *Proceedings of the Geologists' Association*, 113:165–169.

Jarzembowski, E. A. and Schneider, J. W. (2007). The stratigraphical potential of blattodean insects from the late Carboniferous of southern Britain. *Geological Magazine*, 144(3):449–456.

Jaschhof, M. (2007). A neontologist's review of two recently published articles on inclusions of lestremiinae (diptera: Cecidomyiidae) in rovno amber. *Paleontological Journal*, 41(1):103–106.

Jaschhof, M. and Didham, R. K. (2002). Rangomaramidae fam. nov. from New Zealand and implications for the phylogeny of the Sciaroidea (Diptera: Bibionomorpha). *Studia Dipterologica Supplement*, 11:1–60.

Jell, P. A. (2004). The fossil insects of Australia. *Memoirs of the Queensland Museum*, 50(1):1–124.

Jennings, J. T. and Korgmann, L. (2009). A new species of *Pristaulacus* Kieffer (Hymenoptera: Aulacidae) from Baltic amber. *Insect Systematics & Evolution*, 40(2):201–207.

Jepson, J. E. and Jarzembowski, E. A. (2008). Two new species of snakefly (Insecta: Raphidioptera) from the Lower Cretaceous of England and Spain with a review of other fossil raphidiopterans from the Jurassic/Cretaceous transition. *Alavesia*, 2:193–201.

Jepson, J. E., Makarkin, V. N., and Jarzembowski, E. A. (2009). New lacewings (Insecta: Neuroptera) from the Lower Cretaceous Wealden supergroup of southern England. *Cretaceous Research*, 30(5):1325–1338.

Jepson, J. E. and Penney, D. (2007). Neuropteran (insecta) palaeodiversity with predictions for the cretaceous fauna of the wealden. *Palaeogeography, Palaeoclimatology, Palaeoecology*, 248(1-2):109–118.

Johnson, N. F., Musetti, L., and Janzen, J.-W. (2001). A new fossil species of the Australian endemic genus *Peradenia* Naumann & Masner (Hymenoptera: Proctotrupoidea, Peradeniidae) from Baltic amber. *Insect Systematics & Evolution*, 32:191–194.

Johnson, N. F., Musetti, L., and Manser, L. (2008). The Cretaceous scelionid genus *Proteroscelio* brues (Hymenoptera: Platygastroidea). *American Museum Novitates*, 3603:1–7.

Kaddumi, H. F. (2005). *Amber of Jordan, the Oldest Prehistoric Insects in Fossilized Resin.* Publications of the Eternal River Museum of Natural History, Amman.

Kalugina, N. S. and Kovalev, V. G. (1985). *Two winged insects from the Jurassic of Siberia [in Russian].* Nauka, Moscow.

Kania, I. and Wegierek, P. (2008). Palaeoaphididae (Hemiptera, Sternorrhyncha) from Lower Cretaceous Baissa deposits. Morphology and classification. *Monografie Faunistyczne*, 25:1–132.

Kevan, D. K. M. and Wighton, D. C. (1981). Paleocene orthopteroids from south-central Alberta, Canada. *Canadian Journal of Earth Sciences*, 18(12):1824–1837.

Kinzelbach, R. and Lutz, H. (1984). Eine neue Eintagsfliege *Misthodotes stapfi* n. sp. aus dem Rotliegenden des Nahe-Gebietes (Ephemeroptera: Permoplectoptera: Misthodotidae). *Paläontologische Zeitschrift*, 58(3/4):247–253.

Kirby, W. (1815a). *An introduction to Entomology, volume 1.* Longman, London.

Kirby, W. (1815b). Strepsiptera, a new order of Insects proposed and the characters of the order, with those of its genera, laid down. *Transactions of the Linnean Society of London*, 11:86–123.

Kirejtshuk, A. G. (1994). Parandrexidae fam. nov., Jurassic beetles of the Infraorder Cucujiformia (Coleoptera, Polyphaga). *Paleontological Journal*, 28(1):69–78.

Kirejtshuk, A. G. (2008). A current generic classification of sap beetles (Coleoptera, Nitidulidae). *Zoosystematica Rossica*, 17(1):107–122.

Kirejtshuk, A. G. (2009). A new genus and species of Sphaeriusidae (Coleoptera, Myxophaga) from Lower Cretaceous Burmese amber. *Denisia*, 26:99–102.

Kirejtshuk, A. G. and Azar, D. (2008). New taxa of beetles (insecta, coleoptera) from lebanese amber with evolutionary and systematic comments. *Alavesia*, 2:15–46.

Kirejtshuk, A. G., Azar, D., Beaver, R. A., Mandelshtam, M. Y., and Nel, A. (2009a). The most ancient bark beetle known: a new tribe, genus and species from Lebanese amber (Coleoptera, Curculionidae, Scolytinae). *Systematic Entomology*, 34(1):101–112.

Kirejtshuk, A. G., Azar, D., Tafforeau, P., Boistel, R., and Fernandez, V. (2009b). New beetles of Polyphaga (Coleoptera, Polyphaga) from Lower Cretaceous Lebanese amber. *Denisia*, 26:119–130.

Kirejtshuk, A. G. and Nel, A. (2008). New beetles of the suborder Polyphaga from the lowermost Eocene French amber (Isecta: Coleoptera). *Annales de la Société entomologique de France (Nouvelle série)*, 44(4):419–442.

Kirejtshuk, A. G. and Nel, A. (2009). New genera and species of Cucujiformia (Coleoptera, Polyphaga) from lowermost Eocene French amber. *Denisia*, 26:103–118.

Kirejtshuk, A. G. and Poinar, G. O. (2006). Haplochelidae, a new family of Cretaceous beetles (Coleoptera: Myxophaga) from Burmese amber. *Proceedings of the Entomological Society of Washington*, 108(1):155–164.

Kirk-Spriggs, A. H. (2007). A reappraisal of the type fossil of *Curtonotum* Ę*gigas* théobald, 1937 (diptera: Curtonotidae), a compression fossil of early oligocene age from provence, france. *Annals of the Eastern Cape Museums*, 6:13–20.

Klass, K.-D., Zompro, O., Kristensen, N. P., and Adis, J. (2002). Mantophasmatodea: A new insect order with extant members in the afrotropics. *Science*, 296(5572):1456–1459.

Klimaszewski, S. M. (1997). New psyllids from the Baltic amber (Insecta: Homoptera, Aphalaridae). *Mitteilungen aus dem Geologisch-Paläontologischen Institut der Universität Hamburg*, 80:151–171.

Kluge, N. J. (1994). New data on mayflies (Ephemeroptera) from Mesozoic and Cenozoic resins. *Paleontological Journal*, 27(1A):35–49.

Kluge, N. J. (1996). A new suborder of Thysanura for the Carboniferous insect originally described as larva of *Bojophlebia*, with comments on characters of the orders Thysanura and Ephemeroptera. *Zoosystematica Rossica*, 4(1):71–75.

Kluge, N. J. (2004). *The phylogenetic system of Ephemeroptera*. Kluwer Academic Publishers, The Netherlands.

Kluge, N. J., Godunko, R. J., and Krzemiński, W. (2006). A new mayfly family (Insecta: Ephemeroptera) from Eocene Baltic amber. *Annales zoologici*, 56(1):181–185.

Kluge, N. J. and Sinitshenkova, N. D. (2002). 2.2.1.1.1.3 Order Ephemerida Latreille, 1810. the true mayflies (=Ephemeroptera Hyatt et Arms, 1891 (s.l.); =Euephemeroptera Kluge, 2000). In Rasnitsyn, A. P. and Quicke, D. L. J., editors, *History of Insects*, pages 89–97. Kluwer Academic Publishers, The Netherlands.

Koçak, A. O. and Kemal, M. (2008). Replacement names among the genus and family group taxa in Orthoptera. *Centre for Entomological Studies Ankara, Miscellaneous Papers*, 141:1–5.

Kopylov, D. S. (2009). A new subfamily of ichneumonids from the Lower Cretaceous of Transbaikalia and Mongolia (Insecta: Hymenoptera: Ichneumonidae). *Paleontological Journal*, 43(1):83–93.

Koteja, J. (1989). *Inka minuta* gen. et sp. n. (Homoptera, Coccinea) from Upper Cretaceous Taymyrian amber. *Annales zoologici*, 43(5):77–101.

Koteja, J. (2000a). Advances in the study of fossil coccids (Hemiptera: Coccinea). *Polskie Pismo Entomologiczne*, 69(2):187–218.

Koteja, J. (2000b). Scale insects (Homoptera, Coccinea) from Upper Cretaceous New Jersey amber. In Grimaldi, D. A., editor, *Studies on fossils in amber, with particular reference to the Cretaceous of New Jersey*, pages 147–229. Backhuys Publishers, Leiden, The Netherlands.

Koteja, J. (2004). Scale insects (Hemiptera: Coccinea) from Cretaceous Myanmar (Burmese) amber. *Journal of Systematic Palaeontology*, 2(2):109–114.

Koteja, J. (2008). Xylococcidae and related groups (Hemiptera: Coccinea) from Baltic amber. *Prace Muzeum Ziemi*, 49:19–56.

Koteja, J. and Azar, D. (2008). Scale insects from Lower Cretaceous amber of Lebanon (Hemiptera: Sternorrhyncha: Coccinea). *Alavesia*, 2:133–167.

Koteja, J. and Poinar, G. O. (2001). A new family, genus, and species of scale insect (Hemiptera: Coccinea: Kukaspididae, new family) from Cretaceous Alaskan amber. *Proceedings of the Entomological Society of Washington*, 103(2):356–363.

Kotrba, M. (2009). *Prosphyracephala kerneggeri* spec. nov. - a new stalk-eyed fly from Baltic amber. *Spixiana*, 32(2):187–197.

Kozlov, M. V. (1988). Paleontology of lepidopterans and problems in the phylogeny of the order Papilionida. In Ponomarenko, A. G., editor, *The Cretaceous Biocoenotic Crisis in the Evolution of Insects*, pages 16–69. Nauka, Moscow.

Kozlov, M. V., Ivanov, V. D., and Rasnitsyn, A. P. (2002). 2.2.1.3.4.3. Order Lepidoptera Linné, 1758. The butterflies and moths (=Papilionida Laicharting, 1781). In Rasnitsyn, A. P. and Quicke, D. L. J., editors, *History of Insects*, pages 220–227. Kluwer Academic Publishers, The Netherlands.

Krassilov, V. A. (2007). Mines and galls on fossil leaves from the late cretaceous of southern negev, israel. *African Invertebrates*, 48(1):13–22.

Krell, F.-T. (2007). Catalogue of fossil Scarabaeoidea (Coleoptera: Polyphaga) of the Mesozoic and Tertiary. Technical report, Denver Museum of Nature and Science Technical Report 2007-8.

Krishna, K. and Grimaldi, D. A. (2003). The first Cretaceous Rhinotermitidae (Isooptera): a new species, genus, and subfamily in Burmese amber. *American Museum Novitates*, 3390:1–10.

Kristensen, N. P. and Skalski, A. W. (1999). Palaeontology and phylogeny. In Kristensen, N. P., editor, *Handbuch der Zoology: Eine Naturgeschichte der Stämme des Tierreiches: Band IV: Arthopoda: Insecta: Tielband 35: Lepidoptera, Moths and Butterflies: Volume 1: Evolution, Systematics, and Biogeography*, pages 7–25. Walter de Gruyter, Berlin.

Krumbiegel, G. (1997). Der Bitterfelder Bernstein (Succinit). Technical report, Lausitzer und Mitteldeutsche Bergbau-Verwaltungsgesellschaft mbH.

Krzemińska, E., Blagoderov, V., and Krzemiński, W. (1993). Elliidae, a new fossil family of the infraorder Axymyiomorpha (Diptera). *Acta zoologica cracoviensia*, 35:581–591.

Krzemińska, E., Krzemiński, W., and Dahl, C. (2009). *Monograph of fossil Trichoceridae (Diptera): over 180 million years of evolution*. Institute of Systematics and Evolution of Animals, Kraków.

Krzemiński, W. (1992). The oldest Polyneura (Diptera) and their importance to the phylogeny of the group. *Acta zoologica cracoviensia*, 35(1):45–52.

Krzemiński, W. (1992). Triassic and Lower Jurassic stage of Diptera evolution. *Mitteilungen der Schweizerischen entomologischen Gesellschaft*, 65:39–59.

Krzemiński, W. (2007). A revision of Eocene Bittacidae (Mecoptera) from Baltic amber, with the description of a new species. *African Invertebrates*, 48(1):153–162.

Krzemiński, W. and Ansorge, J. (2000). On *Protobrachyceron* Handlirsch, 1920 (Diptera: Brachycera) from the Lower Jurassic of Germany. *Polskie Pismo Entomologiczne*, 69(2):231–237.

Krzemiński, W. and Ansorge, J. (2005). A new rhagionid fly from the Lower Jurassic of Germany (Diptera: Brachycera: Rhagionidae). *Polskie Pismo Entomologiczne*, 74(3):369–372.

Krzemiński, W. and Evenhuis, N. L. (2000). Review of Diptera palaeontological records. In Papp, L. and Darvas, B., editors, *Contributions to a Manual of Palaearctic Diptera, Volume 1, General and applied Dipterology*, pages 535–564. Science Herald, Budapest.

Krzemiński, W. and Krzemińska, E. (2002). Rhaetaniidae, a new family of the Diptera from the Upper Triassic of Great Britain (Diptera: Nematocera). *Annales zoologici*, 52(2):211–213.

Krzemiński, W. and Krzemińska, E. (2003). Triassic Diptera: descriptions, revisions and phylogenetic relations. *Acta zoologica cracoviensia*, 46(suppl.- Fossil Insects):153–184.

Krzemiński, W., Krzemińska, E., and Papier, F. (1994). *Grauvogelia arzvilleriana* sp. n. Ű the oldest Diptera species (Lower/Middle Triassic of France). *Acta zoologica cracoviensia*, 37(2):95–99.

Krzemiński, W. and Lombardo, C. (2001). New fossil Ephemeroptera and Coleoptera from the Ladinian (Middle Triassic) of Canton Ticino (Switzerland). *Rivista Italiana di Paleontologia e Stratigrafia*, 107(1):69–78.

Krzemiński, W. and Lukashevitch, L. (1993). Ansorgiidae, a new family from the Upper Cretaceous of Kazakhstan (Diptera, Ptychopteromorpha). *Acta zoologica cracoviensia*, 35:593–596.

Kubisz, D. (2000). Fossil beetles (Coleoptera) from Baltic amber in the collection of the Museum of Natural History of ISEA in Kraków. *Polskie Pismo Entomologiczne*, 69(2):225–230.

Kukalová, J. (1964). Permian insects of Moravia Part II - Liomopteridea. *Sborník Geologických Věd, Paleontologie, P*, 3:39–118.

Kukalová, J. (1969a). Revisional study of the order Palaeodictyoptera in the Upper Carboniferous Shales of Commentry, France. part I. *Psyche*, 76:163–215.

Kukalová, J. (1969b). Revisional study of the order Palaeodictyoptera in the Upper Carboniferous Shales of Commentry, France. part II. *Psyche*, 76:349–486.

Kukalová-Peck, J. (1975). Megasecoptera from the Lower Permian of Moravia. *Psyche*, 82:1–19.

Kukalová-Peck, J. (1985). Ephemeroid wing venation based upon new gigantic Carboniferous mayflies and basic morphology, phylogeny and metamorphosis of pterygote insects (Insecta, Ephemerida). *Canadian Journal of Zoology*, 63:933–955.

Kukalová-Peck, J. (1987). New Carboniferous Diplura, Monura, and Thysanura, the hexapod ground plan, and the role of thoracic side lobes in the origin of wings (Insecta). *Canadian Journal of Geology*, 65:2327–2345.

Kukalová-Peck, J. and Brauckmann, C. (1990). Wing folding in pterygote insects, and the oldest Diaphanopterodea from the early Late Carboniferous of West Germany. *Canadian Journal of Zoology*, 68:1104–1111.

Kukalová-Peck, J. and Sinitshenkova, N. D. (1992). The wing venation and systematics of Lower Permian Diaphanopterodea from the Ural Mountains, Russia (Insecta: Paleoptera). *Canadian Journal of Zoology*, 70:229–235.

Kumar, P. (2004). Antiquity of Phthiraptera: fossil evidence. *Journal of the Palaeontological Society of India*, 49:159–168.

Kupryjanowicz, J. (2001). Arthropods in Baltic amber and their photographic record. In Kosmowska-Ceranowicz, B., editor, *The amber treasure trove*, pages 19–72. Oficyna Wydawnicza Sadyba, Warsaw.

Kusnezov, N. J. (1903). A new species of *Embia* Latr. from the Crimea (Neuroptera, Embiodea) (preliminary description). *Revue Russe d'Entomologie*, 3(3-4):208–210.

Kvaček, Z., Böhme, M., Dvořák, Z., Konzalová, M., Mach, K., Prokop, J., and Rajchl, M. (2004). Early Miocene freshwater and swamp ecosystems of the Most Basin (northern bohemia) with particular reference to the Bílina Mine section. *Journal of the Czech Geological Society*, 49(1-2):1–40.

Labandeira, C. C. (1994). A compendium of fossil insect families. *Milwaukee Public Museum Contributions in Biology and Geology*, 88:1–71.

Labandeira, C. C. (2001). 1.3.9 Rise and diversification of insects. In Briggs, D. E. G. and Crowther, P. R., editors, *Palaeobiology II*, pages 82–88. Blackwell Science, London.

Labandeira, C. C. (2002). Paleobiology of middle Eocene plant-insect associations from the Pacific Northwest: A preliminary report. *Rocky Mountain Geology*, 37:31–59.

Labandeira, C. C., Kvaček, J., and Mostovski, M. M. (2007). Pollination drops, pollen, and insect pollination of mesozoic gymnosperms. *Taxon*, 56(3):663–695.

Latreille, P. A. (1802). *Histoire Naturelle Générale et Particulièr des Crustacés et des Insectes, Tome 3.* Dufart, Paris, France.

Latreille, P. A. (1825). *Familles naturelles du règne animal, exposées succinctement et dans un ordre analytique, avec l'indication de leurs genres.* Paris.

Laurentiaux-Vieira, F. and Laurentiaux, D. (1986). Paleodictyoptere nouveau du Namurien belge. *Annales de la Société Géologique du Nord*, 105:187–193.

Lawrence, J. F., Archibald, S. B., and Ślipiński, A. (2008). A new species of Prionoceridae from the Eocene of British Columbia. *Annales zoologici*, 58(4):689–693.

Legalov, A. A. (2009a). Annotaed checklist of fossil and Recent species of the family Nemonychidae (Coleoptera from the world fauna). *Amurian zoological journal*, 1(3):200–213.

Legalov, A. A. (2009b). Annotated checklist of Recent and fossil species of the family Belidae (Coleoptera) from the world fauna. *Amurian zoological journal*, 1(4):296–324.

Legalov, A. A. (2009c). A review of fossil and recent species of the family Ithyceridae (Coleoptera) from the world fauna. *Amurian zoological journal*, 1(2):117–131.

Lewis, R. E. and Grimaldi, D. A. (1997). A pulicid flea in Miocene amber from the Dominican Republic (Insecta: Siphonaptera: Pulicidae). *American Museum Novitates*, 3205:1–9.

Lewis, S. E. (1977). Two new species of fossil mayflies (Ephemeroptera: Neoephemeridae and Siphlonuridae) from the Ruby River Basin (Oligocene) of southwestern Montana. *Proceedings of the Entomological Society of Washington*, 79(4):583–587.

Li, T.-T. and Ren, D. (2009). A new fossil genus of Mesosciophilidae (Diptera, Nematocera) with two new species from the Middle Jurassic of Inner Mongolia, China. *Progress in Natural Science*, 19(12):1837–1841.

Li, X.-H., Chen, S., Cao, K., Chen, Y.-H., Xu, B.-L., and Ji, Y. (2009). Paleosols of the Mid-Cretaceous: a report from Zhejiang and Fujian, SE China. *Earth Science Frontiers*, 16(5):63–70.

Li, Y.-L., Ren, D., and Shih, C.-K. (2008). Two Middle Jurassic hanging-flies (Insecta: Mecoptera: Bittacidae) from northeast China. *Zootaxa*, 1929:38–46.

Li, Z., Hong, Y.-C., and Yang, D. (2007). A new middle triassic genus and species of mylacridae (blattodea) from china. *Zootaxa*, 1660:53–59.

Liang, J.-H., Ren, D., Ye, Q.-P., Liu, M., and Meng, X.-M. (2006). The fossil blattaria of china: a review of present knowledge. *Acta Zootaxonomica Sinica*, 31(1):102–108.

Liang, J.-H., Vršanský, P., Ren, D., and Shih, C.-K. (2009). A new Jurassic carnivorous cockroach (Insecta, Blattaria, Raphidiomimidae) from the Inner Mongolia in China. *Zootaxa*, 1974:17–30.

Lienhard, C. and Smithers, C. N. (2002). Psocoptera (Insecta): World catalogue and bibliography. *Instrumenta Biodiversitatis*, 5:xli+745.

Lin, Q.-B. (1980). Mesozoic insects from Zhejiang and Anhui provinces, China. In of Geology, N. I. and Palaeontology, editors, *Division and correlation of the Mesozoic volcano-sedimentary formations in the provinces of Zhejiang and Anhui*, pages 211–234. Science Press, Beijing.

Lin, Q.-B. (1992). Late Triassic insect fauna from Toksun, Xinjiang. *Acta Palaeontologica Sinica*, 31(3):313–335. In Chinese, English summary.

Lin, Q.-B. (1994). Cretaceous insects of China. *Cretaceous Research*, 15:305–316.

Lin, Q.-B. and Huang, D.-Y. (2006). Revision of "*Parahagla lamina*" Lin, 1982 and two new species of *Aboilus* (Orthoptera: Prophalangopsidae) from the Early-Middle Jurassic of northwest China. *Progress in Natural Science*, 16(Special Issue):303–307.

Lin, Q.-B. and Huang, D.-Y. (2008). New Middle Jurassic mayflies (Insecta: Ephemeroptera: Siphlonuridae) from Inner Mongolia, China. *Annales zoologici*, 58(3):521–527.

Lin, Q.-B., Huang, D.-Y., and Nel, A. (2007). A new family of Cavilabiata from the Lower Cretaceous Yixian Formation, China (Odonata: Anisoptera). *Zootaxa*, 1469:59–64.

Lin, Q.-B., Zhang, S., and Huang, D.-Y. (2004). *Fuxiaeschna hsiufnia* gen. nov., spec. nov., a new Lower Cretaceous dragonfly from northwestern China (Aeshnoptera: Rudiaeschnidae). *Odonatologica*, 33(4):437–442.

Linnaeus, C. (1758). *Systema Naturae per Regna Tria Naturae, Secundum Classes, Ordines, Genera, Species, cum Characteribus, Differentiis, Synonymis, Locis [10th edition, revised]*. Salviae, Holmiae [Stockholm], Sweden.

Liu, M., Zhao, Y.-Y., and Ren, D. (2008a). Discovery of three new mordellids (coleoptera, tenebrionoidea) from the yixian formation of western liaoning, china. *Cretaceous Research*, 29(3):445–450.

Liu, Y.-S. and Ren, D. (2006). Progress in the study of Plecoptera fossils. *Acta Zootaxonomica Sinica*, 31(4):758–768.

Liu, Y.-S. and Ren, D. (2008). Two new Jurassic stoneflies (Insecta: Plecoptera) from Daohugou, Inner Mongolia, China. *Progress in Natural Science*, 18:1039–1042.

Liu, Y.-S., Ren, D., Sinitshenkova, N. D., and Shih, C.-K. (2006). A new Middle Jurassic stonefly from Daohugou, Inner Mongolia, China (Insecta: Plecoptera). *Annales zoologici*, 56(3):549–554.

Liu, Y.-S., Ren, D., Sinitshenkova, N. D., and Shih, C.-K. (2008b). Three new stoneflies (Insecta: Plecoptera) from the Yixian Formation of Liaoning, China. *Acta geologica sinica*, 82(2):249–256.

Liu, Y.-S., Sinitshenkova, N. D., and Ren, D. (2007a). A new genus and species of stonefly (Insecta: Plecoptera) from the Yixian Formation, Liaoning Province, China. *Cretaceous Research*, 28(2):322–326.

Liu, Y.-S., Sinitshenkova, N. D., and Ren, D. (2009). A revision of the Jurassic stonefly genera *Dobbertiniopteryx* Ansorge and *Karanemoura* Sinitshenkova (Insecta: Plecoptera), with the description of new species from the Daohugou locality, China. *Paleontological Journal*, 43(2):183–190.

Liu, Z.-W., Engel, M. S., and Grimaldi, D. A. (2007b). Phylogeny and geological history of the cynipoid wasps (Hymenoptera: Cynipoidea). *American Museum Novitates*, 3583:1–48.

López Ruf, M., Pérez Goodwyn, P., and Martins-Neto, R. G. (2005). New Heteroptera (Insecta) from the Santana Formation, Lower Cretaceous (Northeastern Brazil), with description of a new family and new taxa of Naucoridae and Gelastocoridae. *Gaea (Acta Geologica Leopoldensia)*, 1(2):68–74.

Lopez-Vaamonde, C., Wikström, N., Kjer, K. M., Weiblen, G. D., Rasplus, J. Y., Machado, C. A., and Cook, J. M. (2009). Molecular dating and biogeography of fig-pollinating wasps. *Molecular Phylogenetics and Evolution*, 52(3):715–726.

Lopez-Vaamonde, C., Wikstrom, N., Labandeira, C. C., Godfray, H. C. J., Goodman, S. J., and Cook, J. M. (2006). Fossil-calibrated molecular phylogenies reveal that leaf-mining moths radiated millions of years after their host plants. *Journal of Evolutionary Biology*, 19(4):1314–1326.

Lubbock, J. W. (1871). Notes on the Thysanura. part IV. *Transactions of the Linnean Society of London*, 27:277–297.

Lukashevitch, E. D. (1996). Mesozoic Dixidae (Insecta: Diptera) and systematic position of *Dixamima* Rohdendorf, 1964 and *Rhaetomyia* Rohdendorf, 1962. *Paleontological Journal*, 30(1):46–51.

Lukashevitch, E. D. (2000). On the systematic position of *Prodocidia* (Diptera) from the Lower Lias of England. *Paleontological Journal*, 34(Suppl. 3):S352–S354.

Lukashevitch, E. D. (2008). Ptychopteridae (Insecta: Diptera): History of its study and limits of the family. *Paleontological Journal*, 42(1):66–74.

Lukashevitch, E. D., Huang, D.-Y., and Lin, Q.-B. (2006). Rare families of lower Diptera (Hennigmatidae, Blephariceridae, Perissommatidae) from the Jurassic of China. *Studia dipterologica*, 13(1):127–143.

Lukashevitch, E. D. and Shcherbakov, D. E. (1999). A new Triassic family of Dipera from Australia. In Scoggin, M., editor, *AMBA projects AM/PFICM98/1.99: Proceedings of the First International Palaeoentomological Conference, Moscow 1998*, pages 81–89.

MacLeay, W. S. (1825). *Annulosa Javanica, or an attempt to illustrate the natural affinities and analogies of the insects collected in Java by Thomas Horsfield, M.D. F.L. & G.S. and deposited by him in the museum of the honourable East-India Company. Number 1.* Kingsbury, Parbury and Allen, London.

Makarkin, V. M. (2010). New psychopsoid Neuroptera from the Early Cretaceous of Baissa, Transbaikalia. *Annales de la Société entomologique de France (Nouvelle série)*, 46(1-2):254–261.

Makarkin, V. N. (1990). *Baissoleon cretaceus* gen. and sp. nov. Fossil Neuroptera from the Lower Cretaceous of Baisa, East Siberia. 2. Nymphitidae. *Annales de la Société Entomologique de France*, 26(1):125–126.

Makarkin, V. N. (1998). New Tertiary Neuroptera (Insecta) from the Russian Far East. *Tertiary Research*, 18(3-4):77–83.

Makarkin, V. N. (1999). Fossil Neuroptera of the Lower Cretaceous of Baisa, east Siberia. Part 6. Mesithonidae (Insecta). *Neues Jahrbuch für Geologie und Paläontologie, Monatshefte*, 1999(12):705–712.

Makarkin, V. N. and Archibald, S. B. (2003). Family affinity of the genus *Palaeopsychops* Andersen with description of a new species from the early Eocene of British Columbia, Canada (Neuroptera: Polystoechotidae). *Annals of the Entomological Society of America*, 96(3):171–180.

Makarkin, V. N. and Archibald, S. B. (2005). Substitute names for three genera of fossil Neuroptera, with taxonomic notes. *Zootaxa*, 1054:15–23.

Makarkin, V. N. and Menon, F. (2005). New species of the Mesochrysopidae (Insecta, Neuroptera) from the Crato Formation of Brazil (Lower Cretaceous), with taxonomic treatment of the family. *Cretaceous Research*, 26(5):801–812.

Makarkin, V. N. and Menon, F. (2007). First record of fossil Śrapismatid-likeŠ Ithonidae (Insecta, Neuroptera) from the Lower Cretaceous Crato Formation of Brazil. *Cretaceous Research*, 28(5):743–753.

Makarkin, V. N. and Perkovsky, E. E. (2009). *Rophalis relicta* Hagen (Neuroptera, Nevrorthidae) in the late Eocene Rovno amber, with a discussion of the taxonomic status of the genus. *Denisia*, 26:137–144.

Makarkin, V. N., Ren, D., and Yang, Q. (2009). Two new species of Kalligrammatidae (Neuroptera) from the Jurassic of China, with comments on venational homologies. *Annals of the Entomological Society of America*, 102(6):964–969.

Manley, D. G. and Poinar, G. O. (2003). A new specimen of fossil Mutillidae (Hymenoptera) from Dominican amber. *Proceedings of the Entomological Society of Washington*, 105(4):1069–1071.

Marchal-Papier, F., Nel, A., and Grauvogel-Stamm, L. (2000). Nouveaux Orthoptères (Ensifera, Insecta) du Trias des Vosges (France). *Acta Geologica Hispanica*, 35(1-2):5–18.

Marshall, S. A., Buck, M., Skevington, J. H., and Grimaldi, D. (2009). A revision of the family Syringogastridae (Diptera: Diopsoidea). *Zootaxa*, 1996:1–80.

Martill, D. M., Bechly, G., and Heads, S. W. (2007). Appendix: species list for the Crato Formation. In Martill, D. M., Bechly, G., and Loveridge, R. F., editors, *The Crato Fossil Beds of Brazil: Window into an Ancient World*, pages 582–607. Cambridge University Press.

Martin, S. K. (2008). A new protorhyphid fly (Insecta: Diptera: Protorhyphidae) from the Lower Jurassic of the Perth Basin, western Australia. *Alavesia*, 2:253–257.

Martins-Neto, R. G. (1992). Neurópteros (Insecta, Planipennia) da Formação Santana (Cretáceo Inferior), Bacia do Araripe, nordeste do Brasil. VII - Palaeoleontinae, nova subfamilia de Myrmeleontidae e descriçã de novos táxons. *Revista Brasileira de Entomologia*, 36(4):803–815.

Martins-Neto, R. G. (1995a). Araripelocustidae, fam. n. uma nova família de gafanhotos (Insecta, Caelifera) da formação Santana Cretáceo Inferior do nordeste do Brasil. *Revista Brasileira de Entomologia*, 39(2):311–319.

Martins-Neto, R. G. (1995b). Complementos ao estudo sobre os Ensifera (Insecta, Orthopteroida) da Formação Santana, Cretáceo Inferior do nordeste do Brasil. *Revista Brasileira de Entomologia*, 39(2):321–345.

Martins-Neto, R. G. (2001). Review of some Insecta from Mesozoic and Cenozoic Brazilian deposits, with descriptions of new taxa. *Acta Geologica Leopoldensia*, 24(52/53):115–124.

Martins-Neto, R. G. (2002). The Santana Formation paleoentomofauna reviewed. part I - Neuropteroida (Neuroptera and Raphidioptera): systematic and phylogeny, with description of new taxa. *Acta Geologica Leopoldensia (São Leopoldo)*, 25(55):35–66.

Martins-Neto, R. G. (2003). Systematics of the Caelifera (Insecta Orthopteroida) from the Santana Formation, Araripe Basin (Lower Cretaceous, northeast Brazil). *Acta zoologica cracoviensia*, 46(suppl. - Fossil Insects):205–228.

Martins-Neto, R. G. (2005). Estágio atual da paleoartropodologia brasileira: Hexápodes, Miriápodes, Crustáceos (Isopoda, Decapoda, Eucrustacea e Copepoda) e quelicerados. *Arquivos do Museu Nacional, Rio de Janeiro*, 63(3):471–494.

Martins-Neto, R. G. (2007). New Orthoptera Stenopelmatoidea and Hagloidea (Ensifera) from the Santana Formation (Lower Cretaceous, northeast Brazil) with description of new taxa. *Gaea*, 3(1):3–8.

Martins-Neto, R. G. and Gallego, O. F. (2006). Review of Dysmorphoptilidae Handlirsch (Hemiptera: Cicadomorpha) from the Argentinean Triassic, with description of a new subfamily, and a new species. *Polskie Pismo Entomologiczne*, 75(2):185–197.

Martins-Neto, R. G., Gallego, O. F., Brauckmann, C., and Cruz, J. L. (2007a). A review of the South American Palaeozoic entomofauna part I: the Ischnoneuroidea and Cacurgoidea, with description of new taxa. *African Invertebrates*, 48(1):87–101.

Martins-Neto, R. G., Gallego, O. F., and Mancuso, A. C. (2006). The triassic insect fauna from argentina. coleoptera from the los rastros formation (bermejo basin), la rioja province. *Ameghiniana*, 43(3):591–609.

Martins-Neto, R. G., Gallego, O. F., and Zavattieri, A. (2008). The Triassic insect fauna from Argentina: Coleoptera, Hemiptera and Orthoptera from the Potrerillos Formation, south of cerro Cacheuta, Cuyana basin. *Alavesia*, 2:47–58.

Martins-Neto, R. G., Gallego, O. F., and Zavattieri, A. M. (2007b). A new Triassic insect fauna from Cerro Bayo, Potrerillos (Mendoza Province, Argentina) with descriptions of new taxa (Insecta: Blattoptera and Coleoptera). *Alcheringa*, 31(2):199–213.

Martins-Neto, R. G., Heads, S. W., and Bechly, G. (2007c). 11.16 Neuropterida: snakeflies, dobsonflies and lacewings. In Martill, D. M., Bechly, G., and Loveridge, R. F., editors, *The Crato Fossil Beds of Brazil: Window into an Ancient World*, pages 328–340. Cambridge University Press.

Martins-Neto, R. G., Mancuso, A. C., and Gallego, O. F. (2005). The Triassic insect fauna from Argentina. Blattoptera from Los Rastros Formation (Bermejo Basin) La Rioja province. *Ameghiniana*, 42(4):705–723.

Martins-Neto, R. G. and Pesenti, M. (2006). The first fossil Termitidae (Isoptera) from the Oligocene of South America: the Entre-Córregos Formation of the Aiuruoca Basin, Minas Gerais, Brazil. *Journal of the Entomological Research Society*, 8(3):63–68.

Martins-Neto, R. G. and Rodrigues, V. Z. (2009). New Neuroptera (Insecta, Osmylidae and Mesochrysopidae) from the Santana Formation, Lower Cretaceous of northeast Brazil. *Gaea*, 5(1):15–20.

Martins-Neto, R. G. and Tassi, L. V. (2009). The Orthoptera (Ensifera) from the Santana Formation (early Cretaceous, northeast Brazil): A statistical and paleoecological approach, with description of new taxa. *Zootaxa*, 2080:21–37.

Martins-Neto, R. G. and Vulcano, M. A. (1989). Neurópteros (Insecta, Planipennia) da Formação Santana (Cretáceo Inferior), Bacia do Araripe, Nordeste do Brasil. II - Superfamília Myrmeleontoidea. *Revista Brasileira de Entomologia*, 33(2):367–402.

Martynov, A. V. (1927). über eine neue Ordnung der fossilen Insekten, Miomoptera nov. *Zoologischer Anzeiger*, 72:99–109.

Martynov, A. V. (1938). On a new Permian order of orthopteroid insects, Glosselytrodea [in russian]. *Izvestiya akademii nauk SSSR, otdelenie matematicheskikh i estestvennykh nauk*, 1938:187–206.

Mazzarolo, L. A. and Amorim, D. S. (2000). *Cratomyia macrorrhyncha*, a Lower Cretaceous brachyceran fossil from the Santana Formation, Brazil, representing a new species, genus and family of the Stratiomyomorpha (Diptera). *Insect Systematics & Evolution*, 31(1):91–102.

McCafferty, W. P. (1990). Chapter 2. Ephemeroptera. In Grimaldi, D. A., editor, *Insects from the Santana Formation, Lower Cretaceous, of Brazil*, volume 195, chapter 2, pages 25–50. Bulletin of the American Museum of Natural History.

McCafferty, W. P. (1991). Toward a phylogenetic classification of the Ephemeroptera (Insecta): a commentary on systematics. *Annals of the Entomological Society of America*, 84(4):343–360.

McCafferty, W. P. (1997). Discovery and analysis of the oldest mayflies (Insecta, Ephemeroptera) known from amber. *Bulletin de la Société d'Histoire Naturelle de Toulouse*, 133:77–82.

McCafferty, W. P. (2004). Higher classification of the burrowing mayflies (Ephemeroptera: Scapphodonta). *Entomological News*, 115:84–92.

McCafferty, W. P. and Santiago-Blay, J. A. (2009). A new Cretaceous mayfly from Burmese amber (Ephemeroptera: Australiphemeridae). *Entomological News*, 119(5):492–496.

McKellar, R. C., Wolfe, A. P., Tappert, R., and Muehlenbachs, K. (2008). Correlation of Grassy Lake and Cedar Lake ambers using infrared spectroscopy, stable isotopes, and palaeoentomology. *Canadian Journal of Earth Sciences*, 45(9):1061–1082.

Melnitsky, S. I. (2009). A new caddisfly of the extinct genus *Archaeotinodes* (Insecta: Trichoptera: Ecnomidae) from the Baltic amber. *Paleontological Journal*, 43(3):296–299.

Mendes, L. F. and Poinar, G. O. (2004). A new fossil Nicoletiidae (Zygentoma, "Apterygota") in Dominican amber. *Proceedings of the Entomological Society of Washington*, 106(1):102–109.

Menon, F., Heads, S. W., and Szwedo, J. (2007). 11.12 Cicadomorpha: cicadas and relatives. In Martill, D. M., Bechly, G., and Loveridge, R. F., editors, *The Crato Fossil Beds of Brazil: Window into an Ancient World*, pages 283–297. Cambridge University Press.

Menon, F. and Makarkin, V. N. (2008). New fossil lacewings and antlions (insecta, neuroptera) from the lower cretaceous crato formation of brazil. *Palaeontology*, 51(1):149–162.

Mey, E. (2005). *Psittacobrosus bechsteini*: ein neuer ausgestorbener Federling (Insecta, Phthiraptera, Amblycera) vom Dreifarbenara *Ara tricolor* (Psittaciiformes), nebst einer annotierten übersicht über fossile und rezent ausgestorbene Tierläuse. *Anzeiger des Vereins Thüringer Ornithologen*, 5:201–217.

Meyer, H. W. (2003). *The Fossils of Florissant*. Smithsonian Institution Press, Washington.

Michelsen, V. (2000). Oldest authentic record of a fossil calyptrate fly (Diptera): a species of Anthomyiidae from early Coenozoic Baltic amber. *Studia dipterologica*, 7(1):11–18.

Michelsen, V. (2009). Hoffeinsmyiidae, a new extinct family of Schizophora (Diptera) from Baltic amber. *Studia dipterologica*, 15 [for 2008](1/2):211–222.

Michez, D., de Meulemeester, T., Rasmont, P., Nel, A., and Patiny, S. (2009). New fossil evidence of the early diversification of bees: *Paleohabropoda oudardi* from the French Paleocene (Hymenoptera, Apidae, Anthophorini). *Zoologica Scripta*, 38(2):171–181.

Michez, D., Nel, A., Menier, J.-J., and Rasmont, P. (2007). The oldest fossil of a melittid bee (Hymenoptera: Apiformes) from the early Eocene of Oise (France). *Zoological Journal of the Linnean Society*, 150(4):701–709.

Mockford, E. L. (2007). Species of *Philotarsus* from north and middle America and a new philotarsine genus from Mexicao, Guatemala, and the Greater Antilles (Psocoptera: Philotarsidae: Philotarsinae). *Journal of the New York Entomological Society*, 114(3):108–139.

Mostovski, M. B. (1995). New taxa of Ironomyiidae (Diptera, Phoromorpha) from the Cretaceous of Siberia and Mongolia [in Russian]. *Paleontologicheskii Zhurnal*, 4:86–103.

Mostovski, M. B. (1997). On knowledge of fossil flies of the superfamily Archisargoidea (Diptera, Brachycera). *Paleontological Journal*, 31(1):72–78.

Mostovski, M. B. (1999). A brief review of brachycerous flies (Diptera, Brachycera) in the Mesozoic, with descriptions of some curious taxa. In Scoggin, M., editor, *AMBA projects AM/PFICM98/1.99: Proceedings of the First International Palaeoentomological Conference, Moscow 1998*, pages 103–110.

Mostovski, M. B. (2009). Brachyceran assemblages (Insecta: Diptera) as indicators of terrestrial palaeoenvironments in the late Mesozoic. *Palaeontologia africana*, 44:121–125.

Mostovski, M. B., Jarzembowski, E. A., and Coram, R. A. (2003a). Horseflies and anthericids (Diptera: Tabanidae, Athericidae) from the Lower Cretaceous of England and Transbaikalia. *Paleontological Journal*, 37(2):162–169.

Mostovski, M. B. and Martínez-Delclòs, X. (2000). New Nemestrinoidea (Diptera: Brachycera) from the Upper Jurassic-Lower Cretaceous of Eurasia, taxonomy and palaeobiology. *Entomological Problems*, 31(2):137–148.

Mostovski, M. B., Ross, A. J., Szadziewski, R., and Krzemiński, W. (2003b). Redescription of *Simulidium priscum* Westwood and *Pseudosimulium humidum* (Brodie) (Insecta: Diptera: Rhagionidae) from the Purbeck Limestone Group (Lower Cretaceous) of England. *Journal of Systematic Palaeontology*, 1(1):59–64.

Mound, L. A. and Morris, D. C. (2007). The insect order Thysanoptera: classification versus systematics. *Zootaxa*, 1668:395–411.

Nagatomi, A. and Liu, N. (1994). Apystomyiidae, a new family of Asiloidea (Diptera). *Acta Zoologica Academiae Scientiarum Hungaricae*, 40:203–218.

Nagatomi, A., Saigusa, T., Nagatomi, H., and Lyneborg, L. (1991). Apsilocephalidae, a new family of the orthorrhaphous Brachycera (Insecta, Diptera). *Zoological Science*, 8:579–591.

Nagatomi, A. and Yang, D. (1998). A review of extinct Mesozoic genera and families of Brachycera (Insecta, Diptera, Orthorrhapha. *Entomologist's Monthly Magazine*, 134:95–192.

Naumann, I. D. and Masner, L. (1985). Parasitic wasps of the proctotrupoid complex: a new family from Australia and a key to world families (Hymenoptera: Proctotrupoidea sensu lato). *Australian Journal of Zoology*, 33:761–783.

Navás, L. (1916). Notas sobre el orden de los Rafidiópteros (Ins.). *Memorias de la Real Academia de Ciencias y Artes de Barcelona*, 12:507–513.

Nazarenko, V. Y. and Perkovsky, E. E. (2009). A new genus and species of dryophthorid weevils (Coleoptera, Dryophthoridae: Stromboscerinae) from the Rovno amber. *Paleontological Journal*, 43(9):1097–1100.

Nel, A. (1989). *Piroutetia liasina* meunier, 1907, Insecte du Lias de France, espèce-type des Piroutetiidae nov. fam. (Odonatoptera, Meganeurina). *Bulletin du Muséum National d'Histoire Naturelle, Série 4, Section C*, 11(1):15–19.

Nel, A. (2004). New and poorly known Cenozoic sawflies of France (Hymenoptera, Tenthredinoidea, Pamphilioidea). *Deutsche entomologische Zeitschrift*, 51(2):253–269.

Nel, A. (2009). A new Odonata family from the Jurassic of Central Asia (Odonata: Epiproctophora). *Journal of Natural History*, 43(1-2):57–64.

Nel, A. and Arillo, A. (2006). The first Baltic amber dysagrionine damselfly (Odonata: Zygoptera: Thaumatoneuridae: Dysagrioninae). *Annales de la Société entomologique de France (Nouvelle série)*, 42(2):179–182.

Nel, A., Azar, D., Martínez-Delclòs, X., and Makhoul, E. (2004a). A new Upper Cretaceous species of *Chresmoda* from Lebanon - a latest representative of Chresmodidae (Insecta: Polyneoptera inc. sed.): first record of homeotic mutations in the fossil record of insects. *European Journal of Entomology*, 101(1):145–151.

Nel, A. and Bechly, G. (2009). The third petalurid dragonfly from the Lower Cretaceous of Brazil (Odonata: Cretapetaluridae). *Annales zoologici*, 59(3):281–285.

Nel, A., Bechly, G., Delclòs, X., and Huang, D.-Y. (2009a). New and poorly known Mesozoic damsel-dragonflies (Odonata: Isophlebioidea: Campterophlebiidae, Isophlebiidae). *Palaeodiversity*, 2:209–232.

Nel, A., Bechly, G., Jarzembowski, E. A., and Martínez-Delclòs, X. (1998). A revision of the fossil petalurid dragonflies (Insecta: Odonata: Anisoptera: Petalurida). *Paleontologica Lombarda Nuova serie*, 10:1–68.

Nel, A., Bechly, G., and Martínez-Delclòs, X. (2001a). A new fossil dragonfly from the Upper Jurassic in Germany [Odonata, Anisoptera, Protolindeniidae]. *Revue française d'Entomologie*, 23(4):257–261.

Nel, A., Bechly, G., Martínez-Delclòs, X., and Fleck, G. (2001b). A new family of Anisoptera from the Upper Jurassic of Karatau in Kazakhstan (Insecta: Odonata: Juragomphidae n. fam.). *Stuttgarter Beiträge zur Naturkunde Serie B (Geologie und Paläontologie)*, 314:1–9. zzz ICS says 2002.

Nel, A., Béthoux, O., Bechly, G., Martínez-Delclòs, X., and Papier, F. (2001c). The Permo-Triassic Odonatoptera of the "Protodonate" grade (Insecta: Odonatoptera). *Annales de la Société entomologique de France (Nouvelle série)*, 37(4):501–525.

Nel, A., Delclòs, X., and Hutin, A. (2005a). Mesozoic chrysopid-like Planipennia: a phylogenetic approach (Insecta: Neuroptera). *Annales de la Société entomologique de France (Nouvelle série)*, 41(1):29–68.

Nel, A., Fleck, G., Garrouste, R., Gand, G., Lapeyrie, J., Bybee, S. M., and Prokop, J. (2009b). Revision of Permo-Carboniferous griffenflies (Insecta: Odonatoptera: Meganisoptera) based upon new species and redescription of selected poorly known taxa from Eurasia. *Palaeontographica Abteilung A*, 289(4-6):89–121.

Nel, A., Gand, G., Fleck, G., Béthoux, O., Lapeyrie, J., and Garric, J. (1999a). *Saxonagrion minutus* nov. gen. et sp., the oldest damselfly from the Upper Permian of France (Odonatoptera, Panodonata, Saxonagrionidae nov. fam.). *Geobios*, 32(6):883–888.

Nel, A., Gand, G., and Garric, J. (1999b). A new family of Odonatoptera from the continental Upper Permian: The Lapeyriidae (Lodève Basin, France). *Geobios*, 32(1):63–72.

Nel, A., Gand, G., Garric, J., and Lapeyrie, J. (1999c). The first recorded protozygopteran insects from the Upper Permian of France. *Palaeontology*, 42(1):83–97.

Nel, A., Garrouste, R., Bechly, G., Pohl, B., and Escuillié, F. (2006). *Rafaeliana*, a replacement generic name for *Rafaelia* Nel *et al.*, 2005 (Neuropterida). *Bulletin de la Société entomologique de France*, 111(2):190.

Nel, A. and Huang, D.-Y. (2009). First Chinese Cymatophlebiidae from the Middle Jurassic of Inner Mongolia (Odonata: Anisoptera: Aeshnoptera). *Palaeodiversity*, 2:199–204.

Nel, A. and Huguet, A. (2002). Revision of the enigmatic Upper Carboniferous insect *Campyloptera eatoni* Brongniart, 1893 (Insecta: Odonatoptera). *Organisms Diversity & Evolution*, 2(4):313–318.

Nel, A. and Jarzembowski, E. A. (1998). New protomyrmeleontid dragonflies from the Lower Cretaceous of southern England (Insecta, Odonata, Archizygoptera). *Cretaceous Research*, 19(3-4):393–402.

Nel, A., Marchal-Papier, F., Béthoux, O., and Gall, J.-C. (2004b). A "stick insect-like" from the Triassic of the Vosges (France) ("pre-Tertiary Phasmatodea"). *Annales de la Société entomologique de France (Nouvelle série)*, 40(1):31–36.

Nel, A., Marie, V., and Schmeißner, S. (2002). Revision of the lower Mesozoic dragonfly family Triassolestidae Tillyard, 1918 (Odonata: Epiproctophora). *Annales de Paléontologie*, 88:189–214.

Nel, A., Martínez-Delclòs, X., Escuillié, F., and Brisac, P. (1994). Les Aeshnidae fossils: Etat actuel des connaissances (Odonata, Anisoptera). *Neues Jahrbuch für Geologie und Paläontologie, Abhandlungen*, 194(2/3):143–186.

Nel, A., Menier, J.-J., Waller, A., Hodebert, G., and de Ploëg, G. (2003a). New fossil spongilla-flies from the lowermost Eocene amber of France (Insecta, Neuroptera, Sisyridae). *Geodiversitas*, 25(1):109–117.

Nel, A., Néraudeau, D., Perrichot, V., Girard, V., and Gomez, B. (2008). A new dragonfly family from the Upper Cretaceous of France. *Acta Palaeontologica Polonica*, 53(1):165–168.

Nel, A. and Paicheler, J.-C. (1992). Les Odonata fossiles: état actuel des connaissances. Deuxième partie: Les Petaluridae et Cordulegastridae fossiles (Odonata, Anisoptera, Petaluroidea). *Nouvelle Revue dŠEntomologie*, 9(4):305–323.

Nel, A. and Paicheler, J.-C. (1993). Les Odonata fossiles: état actuel des connaissances. Huitième partie: Les Calopterygoidea fossiles (Odonata, Zygoptera). *Bulletin de la Société entomologique de France*, 97(4):381–396.

Nel, A. and Paicheler, J.-C. (1994a). Les Lestoidea (Odonata, Zygoptera) fossils: un inventaire critique. *Annales de Paléontologie*, 80(1):1–59.

Nel, A. and Paicheler, J.-C. (1994b). Les Libelluloidea autres ue Libellulidae fossils. un inventaire critique (Odonata, Corduliidae, Macromiidae, Synthemistidae, Chlorogomphidae et Mesophlebiidae). *Nouvelle Revue d'Entomologie*, 11(4):321–334.

Nel, A., Paicheler, J.-C., and Henrotay, M. (1993). Les "Anisozygoptera" fossiles. phylogénie et classification (Odonata). *Martinia*, 3:1–311.

Nel, A., Papier, F., Grauvogel-Stamm, L., and Gall, J.-C. (1996). *Voltzialestes triasicus* gen. nov., sp. nov., le premier Odonata Protozygoptera du Trias inférieur des Vosges (France). *Paleontologica Lombarda Nuova serie*, 5:25–36.

Nel, A., Perrichot, V., Azar, D., and Néraudeau, D. (2005b). New Rhachiberothidae (Insecta: Neuroptera) in early Cretaceous and early Eocene ambers from France and Lebanon. *Neues Jahrbuch für Geologie und Paläontologie, Abhandlungen*, 235(1):51–85.

Nel, A., Perrichot, V., and Néraudeau, D. (2003b). The oldest trigonalid wasp in the late Albian amber of Charente-Maritime (SW France) (Hymenoptera: Trigonalidae). *Eclogae geologicae Helvetiae*, 96(3):503–508.

Nel, A. and Petrulevičius, J. F. (2005). A new genus and species of damsel-dragonfy from the early Liassic of Germany (Odonata, Liassophlebiidae). *Bulletin de la Société entomologique de France*, 110(2):185–188.

Nel, A., Petrulevičius, J. F., Gentilini, G., and Martínez-Delclòs, X. (2005c). Phylogenetic analysis of the Cenozoic family Sieblosiidae (Insecta: Odonata), with description of new taxa from Russia, Italy and France. *Geobios*, 38(2):219–233.

Nel, A., Petrulevičius, J. F., and Henrotay, M. (2004c). New early Jurassic sawflies from Luxembourg: the oldest record of Tenthredinoidea (Hymenoptera: "Symphyta"). *Acta Palaeontologica Polonica*, 49(2):283–288.

Nel, A., Petrulevičius, J. F., and Jarzembowski, E. A. (2005d). New fossil Odonata from the European Cenozoic (Insecta: Odonata: Thaumatoneuridae, Aeshnidae, ?Idionychidae, Libellulidae). *Neues Jahrbuch für Geologie und Paläontologie, Abhandlungen*, 235(3):343–380.

Nel, A., Petrulevičius, J. F., and Martínez-Delclòs, X. (2005e). New Mesozoic Protomyrmeleontidae (Insecta: Odonatoptera: Archizygoptera) from Asia with a new phylogenetic analysis. *Journal of Systematic Palaeontology*, 3(2):187–201.

Nel, A., Prokop, J., de Ploëg, G., and Millet, J. (2005f). New Psocoptera (Insecta) from the lowermost Eocene amber of Oise, France. *Journal of Systematic Palaeontology*, 3(4):371–391.

Nel, A., Roques, P., Nel, P., Prokop, J., and Steyer, J. S. (2007a). The earliest holometabolous insect from the Carboniferous: a ŞcrucialŤ innovation with delayed success (Insecta Protomeropina Protomeropidae). *Annales de la Société entomologique de France (Nouvelle série)*, 43(3):349–355.

Nel, A. and Roy, R. (1996). Revision of the fossil ŞmantidŤ and ŞephemeridŤ species described by Piton from the Palaeocene of Menat (France) (Mantodea: Chaeteessidae, Mantidae; Ensifera: Tettigonioidea). *European Journal of Entomology*, 93:223–234.

Nel, A. and Waller, A. (2007). The first fossil Compsocidae from Cretaceous Burmese amber (Insecta, Psocoptera, Troctomorpha). *Cretaceous Research*, 28(6):1039–1041.

Nel, P., Azar, D., and Nel, A. (2007b). A new 'primitive' family of thrips from early Cretaceous Lebanese amber (Insecta, Thysanoptera). *Cretaceous Research*, 28(6):1033–1038.

Nelson, C. R. and Tidwell, W. D. (1987). *Brodioptera stricklani* n. sp. (Megasecoptera: Brodiopteridae), a new fossil insect from the Upper Manning Canyon Shale Formation, Utah (Lowermost Namurian B). *Psyche*, 94:309–316.

Novokshonov, V. G. (1992). Caddisflies of the genus *Kamopanorpa* (Trichoptera, Microptysmatidae) from the Kungurian of Chekarda (Perm District). *Paleontological Journal*, 26(3):136–141.

Novokshonov, V. G. (1994a). Caddis flies (Insecta, Trichoptera, Microptysmatidae). *Paleontological Journal*, 27(1A):90–102.

Novokshonov, V. G. (1994b). New insects (Insecta) from the Lower Permian of Chekarda (Central Urals). *Paleontological Journal*, 27 [for 1993](1A):172–178.

Novokshonov, V. G. (1994c). Permian scorpionflies (Insecta: Panorpida) of the families Kaltanidae, Permochoristidae and Robinjohniidae. *Paleontological Journal*, 28(1):79–95.

Novokshonov, V. G. (1997a). New taxa of fossil insects from the Lower Permian of the middle Urals. *Paleontological Journal*, 31(4):383–388.

Novokshonov, V. G. (1997b). Some Mesozoic scorpionflies (Insecta: Panorpida = Mecoptera) of the families Mesopsychidae, Pseudopolycentropodidae, Bittacidae, and Permochoristidae. *Paleontological Journal*, 31(1):65–71.

Novokshonov, V. G. (1998a). New fossil insects (Insecta: Grylloblattida, Caloneurodea, Hypoperlida?, *ordinis incertis*) from the Kungurian beds of the middle Urals. *Paleontological Journal*, 32(4):362–368.

Novokshonov, V. G. (1998b). New insects (Insecta: Hypoperlida, Mischopterida, Jurinida) from the Lower Permian of the Middle Urals. *Paleontological Journal*, 32(1):46–53.

Novokshonov, V. G. (1999). New fossil insects (Insecta: Hypoperlida, Panorpida, *ordinis incertis*) from the Chekarda locality. *Paleontological Journal*, 33(1):52–56.

Novokshonov, V. G. (2000). New fossil insects (Insecta: Grylloblattida, ordinis incertis) from the Lower Permian of the middle Urals. *Paleontological Journal*, 34(5):513–518.

Novokshonov, V. G. (2001). New and little-known representatives of the family Hypoperlidae (Insecta: Hypoperlida). *Paleontological Journal*, 35(1):40–44.

Novokshonov, V. G. (2002a). 2.2.1.3.4.1. Order Panorpida Latreille, 1802. The Scorpionflies (=Mecoptera Packard, 1886, =Mecoptera Comstock et Comstock, 1895, +Neomecoptera

Hinton, 1958, +Paratrichoptera Tillyard, 1919, +Paramecoptera Tillyard, 1919). In Rasnitsyn, A. P. and Quicke, D. L. J., editors, *History of Insects*, pages 194–199. Kluwer Academic Publishers, The Netherlands.

Novokshonov, V. G. (2002b). New enigmatic insects (Insecta: Hypoperlidea?: Sojanoperidae) from the Upper Permian of northern Russia. *Paleontological Journal*, 36(1):48–49.

Novokshonov, V. G. (2004). The first mecopteroids (Insecta: Papilionidea = Mecopteroidea) and the origin of scorpionflies (Panorpida = Mecoptera), with description of a legless eruciform larva. *Paleontological Journal*, 38(Suppl. 2):S204–S213.

Novokshonov, V. G. and Aristov, D. S. (2002). New and little-known Permian insects (Insecta: Grylloblattida; Orthoptera) from the Chekarda locality, Central Ural Mountains. *Paleontological Journal*, 36(6):644–649.

Novokshonov, V. G. and Aristov, D. S. (2004). New taxa of hypoperlids (Insecta: Hypoperlida) from the Upper Permian of the Arkhangelsk Region. *Paleontological Journal*, 38(1):60–66.

Novokshonov, V. G., Ivanov, V. V., and Aristov, D. S. (2002). New insects from the late Permian of the Ural Mountains. *Paleontological Journal*, 36(2):157–160.

Novokshonov, V. G. and Novokshonova, E. A. (1997). *Okolpania favorabilis* n. sp. (Planipennia; Neuroptera: Permithonidae) from the Lower Permian of Ural. *Paläontologische Zeitschrift*, 71(1/2):89–90.

Novokshonov, V. G. and Rasnitsyn, A. P. (2000). A new enigmatic group of insects (Psocidea, Tshekarcephalidae) from Tshekarda (Lower Permian of the middle Urals. *Paleontological Journal*, 34(Suppl. 3):S284–S287.

Novokshonov, V. G. and Zhuzhgova, L. V. (2004). Discussion of the system and phylogeny of the order Palaeomanteida (= Miomoptera) with description of new representatives of the genus *Permosialis* Mart. from the late Permian of Kirov Region and Triassic of Kyrgyzstan. *Paleontological Journal*, 38(Suppl. 2):S173–S184.

Nyman, T., Zinovjev, A. G., Vikberg, V., and Farrell, B. D. (2006). Molecular phylogeny of the sawfly subfamily Nematinae (Hymenoptera: Tenthredinidae). *Systematic Entomology*, 31(4):569–583.

Oberprieler, R. G., Marvaldi, A. E., and Anderson, R. S. (2007). Weevils, weevils, weevils everywhere. *Zootaxa*, 1668:419–520.

Ogden, T. H., Gattolliat, J. L., Sartori, M., Staniczek, A. H., Soldán, T., and Whiting, M. F. (2009). Towards a new paradigm in mayfly phylogeny (Ephemeroptera): combined analysis of morphological and molecular data. *Systematic Entomology*, 34(4):616–634.

Ohl, M. (2004). The first fossil representative of the wasp genus *Dolichurus*, with a review of fossil Ampulicidae (Hymenoptera: Apoidea). *Journal of the Kansas Entomological Society*, 77(4):322–342.

Olivier, G. A. (1789). *Encyclodpédie méthodique. Dictionnaire des insectes, v. 5*. Pankouke, Paris.

Ortega-Blanco, J., Rasnitsyn, A. P., and Delclòs, X. (2008). First record of anaxyelid woodwasps (Hymenoptera: Anaxyelidae) in Lower Cretaceous Spanish amber. *Zootaxa*, 1937:39–50.

Osten, T. (2007). 11.8 Hymenoptera: bees, wasps and ants. In Martill, D. M., Bechly, G., and Loveridge, R. F., editors, *The Crato Fossil Beds of Brazil: Window into an Ancient World*, pages 350–365. Cambridge University Press.

Özdikmen, H. (2008a). New subfamily and genus names for Ferganiinae Gorochov, 1987 and *Fergania* Sharov, 1968 (Orthoptera). *Munis Entomology and Zoology*, 3(2):731–732.

Özdikmen, H. (2008b). Some nomenclatural changes for Blattodea and Dictyoneurida (=Palaeodictyoptera). *Munis Entomology and Zoology*, 3(2):745–748.

Packard, A. S. (1886). A new arrangement of the orders of insects. *American Naturalist*, 20:808.

Papier, F. and Nel, A. (2001). Les Subioblattidae (Blattodea, Insecta) du Trias d'Asie Centrale. *Paläontologische Zeitschrift*, 74(4):533–542.

Papier, F., Nel, A., Grauvogel-Stamm, L., and Gall, J.-C. (1997). La plus ancienne sauterelle Tettigoniidae, Orthoptera (Trias, NE France): mimétisme ou exaptation? *Paläontologische Zeitschrift*, 71(1/2):71–77.

Peñalver, E. and Arillo, A. (2007). A new species of the family Hybotidae in the Lower Cretaceous amber of El Caleyu (Asturias, Spain); *Alavesia prietoi* n. sp. *Alavesia*, 1:63–68.

Peñalver, E. and Grimaldi, D. A. (2006). New data on Miocene butterflies in Dominican amber (Lepidoptera, Riodinidae and Nymphalidae) with the description of a new nymphalid. *American Museum Novitates*, 3519:1–17.

Peñalver, E., Martínez-Delclòs, X., and Arillo, A. (1999). Yacimientos con insectos fósiles en España. *Revista Española de Paleontología*, 14(2):231–245.

Peñalver, E., Nel, A., and Martínez-Delclòs, X. (1996). Insectos del Mioceno inferior de Ribesalbes (Castellón, Spain). Paleoptera y Neoptera poli- y paraneoptera. *Treballs del Museu de Geología de Barcelona*, 5:15–95.

Peng, D.-C., Hong, Y.-C., and Zhang, Z.-J. (2005). Namurian insects (Diaphanopterodea) from Qilianshan Mountains, China. *Geological Bulletin of China*, 24(3):219–234.

Pérez, D. E., Hierro, B., Dominici, G. O., and Otte, D. (1997). New eumastacid greasshopper taxa (Orthoptera: Eumastacidae: Episactinae) from Hispaniola, including a fossil new genus and species from Dominican amber. *Journal of Orthoptera Research*, 6:139–151.

Pérez-Gelabert, D. E. (2008). Arthropods of Hispaniola (Dominican Republic and Haiti): A checklist and bibliography. *Zootaxa*, 1831:1–530.

Pérez-Gelabert, D. E. and Rowell, C. H. F. (2006). Further investigations of Hispaniolan eumastacoid grasshoppers (Espagnolinae: Episactidae: Orthoptera). *Journal of Orthoptera Research*, 15(2):241–249.

Perkovsky, E. E. (2001). The systematic position of the Lower Cretaceous beetle *Mesecanus parvus* (Coleoptera, Staphylinoidea) from Turga. *Vestnik zoologii*, 35(4):79–81.

Perkovsky, E. E., Rasnitsyn, A. P., Vlaskin, A. P., and Taraschuk, M. V. (2007). A comparative analysis of the Baltic and Rovno amber arthropod faunas: representative samples. *African Invertebrates*, 48(1):229–245.

Perkovsky, E. E., Zosimovich, V. Y., and Vlaskin, A. P. (2003). Rovno amber insects: first results of analysis. *Russian Entomological Journal*, 12(2):119–126.

Perrichot, V. (2004). Early Cretaceous amber from south-western France: insight into the Mesozoic litter fauna. *Geologica Acta*, 2(1):9–22.

Perrichot, V. (2009). Long-tailed wasps (Hymenoptera: Megalyridae) from Cretaceous and Paleogene European amber. *Paleontological Contributions*, 1:1–35.

Perrichot, V., Azar, D., Néraudeau, D., and Nel, A. (2003). New Psocoptera in the early Cretaceous amber of SW France and Lebanon (Insecta: Psocoptera: Trogiomorpha). *Geological Magazine*, 140(6):669–683.

Perrichot, V. and Engel, M. S. (2007). Early Cretaceous snakefly larvae in amber from Lebanon, Myanmar, and France (Raphidioptera). *American Museum Novitates*, 3598:1–11.

Perrichot, V. and Nel, A. (2008a). Eocene bethylid wasps from French amber (Hymenoptera: Bethylidae). *Neues Jahrbuch für Geologie und Paläontologie, Abhandlungen*, 248(1):91–101.

Perrichot, V. and Nel, A. (2008b). A new belytine wasp in Cretaceous amber from France (Hymenoptera: Diapriidae). *Alavesia*, 2:203–209.

Perrichot, V., Nel, A., Guilbert, E., and Néraudeau, D. (2006). Fossil Tingoidea (Heteroptera: Cimicomorpha) from French Cretaceous amber, including Tingidae and a new family, Ebboidae. *Zootaxa*, 1203:57–68.

Perrichot, V., Nel, A., and Néraudeau, D. (2004). Two new wedge-shaped beetles in Albo-Cenomanian ambers of France (Coleoptera: Ripiphoridae: Ripiphorinae). *European Journal of Entomology*, 101(4):577–581.

Perrichot, V., Nel, A., and Quicke, D. L. J. (2009). New braconid wasps from French Cretaceous amber (Hymenoptera, Braconidae): synonymization with Eoichneumonidae and implications for the phylogeny of Ichneumonoidea. *Zoologica Scripta*, 38(1):79–88.

Perrichot, V., Néraudeau, D., Azar, D., Menier, J.-J., and Nel, A. (2002). A new genus and species of fossil mole cricket in the Lower Cretaceous amber of Charente-Maritime, SW France (Insecta: Orthoptera: Gryllotalpidae). *Cretaceous Research*, 23(3):307–314.

Perrichot, V., Néraudeau, D., Nel, A., and de Ploëg, G. (2007). A reassessment of the Cretaceous amber deposits from France and their palaeontological significance. *African Invertebrates*, 48(1):213–227.

Pescador, M. L., Richard, B. A., Hubbard, M. D., and Staniczek, A. H. (2009). Evolution of Baetiscidae (Ephemeroptera): current state of knowledge of the family. *Aquatic Insects*, 31(Supplement 1):137–147.

Petrulevičius, J. F. (2009). A panorpid (Insecta: Mecoptera) from the Lower Eocene of Patagonia, Argentina. *Journal of Paleontology*, 83(6):994–997.

Petrulevičius, J. F. and Nel, A. (2002). New palaeomacromiid dragonflies from the upper Palaeocene of Argentina. *Palaeontology*, 45(4):751–758.

Petrulevičius, J. F. and Nel, A. (2003a). Frenguelliidae, a new family of dragonflies from the earliest Eocene of Argentina (Insecta: Odonata): phylogenetic relationships within Odonata. *Journal of Natural History*, 37(24):2909–2917.

Petrulevičius, J. F. and Nel, A. (2003b). Oldest petalurid dragonfly (Insecta: Odonata): a Lower Cretaceous specimen from south Patagonia, argentina. *Cretaceous Research*, 24(1):31–34.

Petrulevičius, J. F. and Nel, A. (2004). A new damselfly family from the Upper Palaeocene of Argentina. *Palaeontology*, 47(1):109–116.

Petrulevičius, J. F. and Nel, A. (2005). Austroperilestidae, a new family of damselflies from early Eocene of Argentina (Insecta: Odonata). phylogenetic relationships within Odonata. *Journal of Paleontology*, 79(4):658–662.

Petrulevičius, J. F. and Nel, A. (2007). Enigmatic and little known Odonata (Insecta) from the Paleogene of Patagonia and northwest Argentina. *Annales de la Société entomologique de France (Nouvelle série)*, 43(3):341–347.

Petrulevičius, J. F. and Nel, A. (2009). First Cordulephyidae dragonfly in America: A new genus and species from the Paleogene of Argentina (Insecta: Odonata). *Comptes Rendus Palevol*, 8(4):385–388.

Petrulevičius, J. F., Nel, A., and Muzón, J. (1999). A new libelluloid family from the upper Palaeocene of Argentina. *Palaeontology*, 42(4):677–682.

Petrulevičius, J. F., Nel, A., Rust, J., Bechly, G., and Kohls, D. (2007). New Paleogene Epallagidae (Insecta: Odonata) recorded in North America and Europe. biogeographic implications. *Alavesia*, 1:15–25.

Pinto, I. D. (1986). Carboniferous insects from Argentina III Familia Xenopteridae Pinto, nov. Ordo Megasecoptera. *Pesquisas*, 18:23–29.

Pinto, I. D. (1994). *Sphecorydaloides lucchesei* a new Carboniferous megasecopteran Insecta from Argentina. *Pesquisas*, 21(2):85–89.

Pinto, I. D. (1996). *Rigattoptera ornellasae* n. g., n. sp., a new fossil insect from the Carboniferous of Argentina. *Neues Jahrbuch für Geologie und Paläontologie, Monatshefte*, 1996(1):43–47.

Pinto, I. D. and Adami-Rodrigues, K. (1999). A revision of South American Paleozoic insects. In Scoggin, M., editor, *AMBA projects AM/PFICM98/1.99: Proceedings of the First International Palaeoentomological Conference, Moscow 1998*, pages 117–124.

Pinto, I. D. and Pinto de Ornellas, L. P. (1991). Substitute names for the extinct Insecta families Narkemocacurgidae Pinto & Ornellas, 1978 and Cacurgonarkemidae Pinto, 1990. *Pesquisas*, 18(1):93.

Pohl, H. (2009). The oldest fossil strepsipteran larva (Insecta: Strepsiptera) from the Geisel Valley, Germany (Eocene). *Insect Systematics & Evolution*, 40(4):333–347.

Pohl, H., Beutel, R. G., and Kinzelbach, R. (2005). Protoxenidae fam. nov. (Insecta, Strepsiptera) from Baltic amber - a 'missing link' in strepsipteran phylogeny. *Zoologica Scripta*, 34(1):57–69.

Poinar, G. and Poinar, R. (1999). *The amber forest, a reconstruction of a vanished world.* Princeton University Press.

Poinar, G. O. (1992). *Life in amber.* Stanford University Press, Standford, California.

Poinar, G. O. (2009a). Description of an early Cretaceous termite (Isoptera: Kalotermitidae) and its associated intestinal protozoa, with comments on their co-evolution. *Parasites & Vectors*, 2(2):17pp.

Poinar, G. O. (2009b). *Melittosphex* (Hymenoptera: Melittosphecidae), a primitive bee and not a wasp. *Palaeontology*, 52(2):483–484.

Poinar, G. O. and Brown, A. E. (2005). New Aphidoidea (Hemiptera: Sternorrhyncha) in Burmese amber. *Proceedings of the Entomological Society of Washington*, 107(4):835–845.

Poinar, G. O. and Brown, A. E. (2006). Remarks on *Parvaverrucosa annulata* (= *Verrucosa annulata* Poinar and Brown 2005) (Hemiptera: Sternorrhyncha: Aphidoidea). *Proceedings of the Entomological Society of Washington*, 108(3):734–735.

Poinar, G. O. and Brown, A. E. (2009). *Pantostictus burmanicus*, a new genus and species of Cretaceous beetles (Coleoptera: Hydrophiloidea: Histeridae) in Burmese amber. *Proceedings of the Entomological Society of Washington*, 111(1):38–46.

Poinar, G. O. and Buckley, R. (2009). *Palaeoleptus burmanicus* n. gen., n. sp., an Early Cretaceous shore bug (Hemiptera: Palaeoleptidae n. fam.) in Burmese amber. *Cretaceous Research*, 30(4):1000–1004.

Poinar, G. O. and Danforth, B. N. (2006). A fossil bee from early Cretaceous Burmese amber. *Science*, 314:614.

Poinar, G. O., Gorokhov, A. V., and Buckley, R. (2007). *Longioculus burmensis*, n. gen., n. sp. (Orthoptera: Elcanidae) in Burmese amber. *Proceedings of the Entomological Society of Washington*, 109(3):649–655.

Poinar, G. O. and Milki, R. (2001). *Lebanese Amber: The Oldest Insect Ecosystem in Fossilized Resin.* Oregon State University Press, Corvallis.

Poinar, G. O. and Poinar, R. (2008). *What Bugged the Dinosaurs? Insects, Disease, and Death in the Cretaceous.* Princeton University Press.

Poinar, G. O., Zavortink, T. J., Pike, T., and Johnston, P. A. (2000). *Paleoculicis minutus* (Diptera: Culicidae) n. gen., n. sp., from Cretaceous Canadian amber, with a summary of described fossil mosquitoes. *Acta Geologica Hispanica*, 35(1-2):119–128.

Polhemus, J. T. (2000). North American Mesozoic aquatic Heteroptera (Insecta, Naucoroidea, Nepoidea) from the Todilto Formation, New Mexico p.29-40. In Lucas, S. G., editor, *New Mexico's Fossil Record 2*, volume Bulletin 16, page 284. New Mexico Museum of Natural History and Science, Albuquerque.

Ponomarenko, A. G. (1985). Fossil insects from the Tithonian "Solnhofener Plattenkalke" in the Museum of Natural History, Vienna. *Annalen des Naturhistorischen Museums in Wien*, 87A:135–144.

Ponomarenko, A. G. (1986). Beetles. Scarabaeida (=Coleoptera). *The Joint Soviet-Mongolian Palaeontological Expedition*, 28:84–105.

Ponomarenko, A. G. (1992). Neuroptera (Insecta) from the Lower Cretaceous of Transbaykalia. *Paleontological Journal*, 26(3):56–66.

Ponomarenko, A. G. (1994). Two new species of Mesozoic dytiscoid beetles from Asia. *Paleontological Journal*, 27 [for 1993](1A):182–191.

Ponomarenko, A. G. (1996). Upper Liassic neuropterans (Insecta) from Lower Saxony, Germany. *Russian Entomological Journal*, 4(for 1995):73–89.

Ponomarenko, A. G. (2000a). New alderflies (Megaloptera: Parasialidae) and glosselytrodeans (Glosselytrodea: Glosselytridae) from the Permian of Mongolia. *Paleontological Journal*, 34(Suppl. 3):S309–S311.

Ponomarenko, A. G. (2000b). New beetles from the Permian of European Russia. *Paleontological Journal*, 34(Suppl. 3):S312–316.

Ponomarenko, A. G. (2002a). 2.2.1.3.2. Superorder Scarabaeidea Laicharting, 1781. Order Coleoptera Linné, 1758. The Beetles. In Rasnitsyn, A. P. and Quicke, D. L. J., editors, *History of Insects*, pages 164–176. Kluwer Academic Publishers, The Netherlands.

Ponomarenko, A. G. (2002b). 2.2.1.3.3. Superorder Myrmeleontidea Latreille, 1802 (=Neuropteroidea Handlirsch, 1903). In Rasnitsyn, A. P. and Quicke, D. L. J., editors, *History of Insect*, pages 176–189. Kluwer Academic Publishers, The Netherlands.

Ponomarenko, A. G. (2003a). Ecological evolution of beetles. *Acta zoologica cracoviensia*, 46(suppl. - Fossil Insects):319–328.

Ponomarenko, A. G. (2003b). On some Neuroptera (Insecta) from Upper Jurassic Solnhofen Limestone. *Annals of the Upper Silesian Museum (Entomology)*, 12:87–100.

Ponomarenko, A. G. (2008). New Triassic beetles (Coleoptera) from northern European Russia. *Paleontological Journal*, 42(6):600–606.

Ponomarenko, A. G. and Mostovski, M. B. (2005). New beetles (Insecta: Coleoptera) from the late Permian of South Africa. *African Invertebrates*, 46:253–260.

Ponomarenko, A. G. and Shcherbakov, D. E. (2004). New lacewings (Neuroptera) from the terminal Permian and basal Triassic of Siberia. *Paleontological Journal*, 38(Suppl. 2):S197–S203.

Ponomarenko, A. G., Sukatsheva, I. D., and Vasilenko, D. V. (2009). Some characteristics of the Trichoptera distribution in the Mesozoic of Eurasia (Insecta: Trichoptera. *Paleontological Journal*, 43(3):282–295.

Popov, Y. A. (1986). New peloridiids and heteropteran bugs Peloridiina (=Coleorhyncha) et Cimicina (=Heteroptera) [in Russian]. *Transactions of the Joint Soviet-Mongolian Palaeontological Expedition*, 28:50–83.

Popov, Y. A. (1990). Klopy. Cimicina [Bugs. Cimicina]. *Trudy Paleontologicheskogo instituta Akademiia nauk SSSR*, 239:20–39.

Popov, Y. A. (1992). Jurassic bugs (Hemiptera: Heteroptera) from the Museum of Natural History in Vienna. *Annalen des Naturhistorischen Museums in Wien*, 94A:7–14.

Popov, Y. A. (2007). A new notion on the heteropterofauna (Insecta: Hemiptera: Heteroptera) from the Pliocene of Willershausen. *Paläontologische Zeitschrift*, 81(4):429–439.

Popov, Y. A. (2008). *Pavlostysia wunderlichi* gen. nov. and sp. nov., the first fossil spider-web bug (Hemiptera: Heteroptera: Cimicomorpha: Plokiophilidae) from the Baltic Eocene amber. *Acta Entomologica Musei Nationalis Pragae*, 48(2):497–502.

Popov, Y. A. and Bechly, G. (2007). 11.15 Heteroptera: bugs. In Martill, D. M., Bechly, G., and Loveridge, R. F., editors, *The Crato Fossil Beds of Brazil: Window into an Ancient World*, pages 317–328. Cambridge University Press.

Popov, Y. A., Dolling, W. R., and Whalley, P. E. S. (1994). British Upper Triassic and Lower Jurassic Heteroptera and Coleorrhyncha (Insecta: Hemiptera). *Genus*, 5(4):307–347.

Popov, Y. A. and Shcherbakov, D. E. (1991). Mesozoic Peloridioidea and their ancestors. *Geologica et Palaeontologica*, 25:215–235.

Pritykina, L. N. (1986). Two new dragonflies from the Lower Cretaceous deposits of west Mongolia (Anisoptera: Sonidae fam. nov., Corduliidae). *Odonatologica*, 15(2):169–184.

Pritykina, L. N. (2006). Isophlebiid dragonflies from the late Mesozoic of eastern Transbaikalia (Odonata: Isophlebiidae). *Paleontological Journal*, 40(6):636–645.

Prokop, J. and Fikaček, M. (2007). An annotated list of early Oligocene insect fauna from Seifhennersdorf (Saxony, Germany). *Acta Musei Nationalis Pragae, Series B Ũ Historia Naturalis*, 63(2-4):209–217.

Prokop, J. and Nel, A. (2004). A new genus and species of Homoiopteridae from the Upper Carboniferous of the Intra-Sudetic Basin, Czech Republic (Insecta: Palaeodictyoptera). *European Journal of Entomology*, 101(4):583–589.

Prokop, J. and Nel, A. (2007). An enigmatic Palaeozoic stem-group: Paoliida, designation of new taxa from the Upper Carboniferous of the Czech Republic (Insecta: Paoliidae, Katerinkidae fam. n.). *African Invertebrates*, 48(1):77–86.

Prokop, J. and Nel, A. (2009). Systematic position of *Triplosoba*, hitherto the oldest mayfly, from Upper Carboniferous of Commentry in Central France (Insecta: Palaeodictyopterida). *Systematic Entomology*, 34(4):610–615.

Prokop, J., Nel, A., Hájek, J., and Bubík, M. (2004). First record of a fossil beetle (Coleoptera, Haliplidae) from the basal Paleocene flysch sediments in the Magura Unit (Outer Western Carpathians, Moravia). *Geologica Carpathica*, 55(6):469–473.

Prokop, J., Nel, A., and Hoch, I. (2005). Discovery of the oldest known Pterygota in the Lower Carboniferous of the Upper Silesian Basin in the Czech Republic (Insecta: Archaeorthoptera). *Geobios*, 38(3):383–387.

Prokop, J. and Ren, D. (2007). New significant fossil insects from the Upper Carboniferous of Ningxia in northern China (Palaeodictyoptera, Archaeorthoptera). *European Journal of Entomology*, 104(2):267–275.

Prokop, J., Smith, R., Jarzembowski, E. A., and Nel, A. (2006). New homoiopterids from the Late Carboniferous of England (Insecta: Palaeodictyoptera). *Comptes Rendus Palevol*, 5(7):867–873.

Pulawski, W. J., Rasnitsyn, A. P., Brothers, D. J., and Archibald, S. B. (2000). New genera of Angarosphecinae: *Cretosphecium* from early Cretaceous of Mongolia and *Eosphecium* from early Eocene of Canada (Hymenoptera: Sphecidae). *Journal of Hymenoptera Research*, 9(1):34–40.

Pütz, A., Hernando, C., and Ribera, I. (2004). A new genus of Limnichidae (Coleoptera) from Baltic amber. *Insect Systematics & Evolution*, 35(3):323–334.

Rasnitsyn, A. P. (1975). Hymenoptera Apocrita of the Mesozoic. *Trudy Paleontologicheskogo instituta Akademiia nauk SSSR*, 174:1–191. [in Russian].

Rasnitsyn, A. P. (1993). *Strashila incredibilis*, a new enigmatic mecopteroid insect with possible siphonapteran affinities from the Upper Jurassic of Siberia. *Psyche*, 99(4):323–333.

Rasnitsyn, A. P. (1996). New early Cretaceous Embolemidae (Vespida = Hymenoptera: Chrysidoidea). *Memoirs of the Entomological Society of Washington*, 17:183–187.

Rasnitsyn, A. P. (2000a). Taxonomy and morphology of *Dasyleptus* Brongniart, 1885, with description of a new species (Insecta: Machilida: Dasyleptidae). *Russian Entomological Journal*, 8 [for 1999](3):145–154.

Rasnitsyn, A. P. (2000b). Testing cladograms by fossil record: the ghost range test. *Contributions to Zoology*, 69(4):251–258.

Rasnitsyn, A. P. (2002a). 2.2 Subclass Scarabaeona Laicharting, 1781. The winged insects (=Pterygota Lang, 1888). In Rasnitsyn, A. P. and Quicke, D. L. J., editors, *History of Insects*, pages 75–82. Kluwer Academic Publishers, The Netherlands.

Rasnitsyn, A. P. (2002b). 2.2.1.1.1.2. Order Syntonopterida Handlirsch, 1911. In Rasnitsyn, A. P. and Quicke, D. L. J., editors, *History of Insects*, pages 88–89. Kluwer Academic Publishers, The Netherlands.

Rasnitsyn, A. P. (2002c). 2.2.1.2.1.1. Order Blattinopseida Bolton, 1925. In Rasnitsyn, A. P. and Quicke, D. L. J., editors, *History of Insects*, page 106. Kluwer Academic Publishers, The Netherlands.

Rasnitsyn, A. P. (2002d). 2.2.1.2.1.2. Order Caloneurida Handlirsch, 1906 (=Caloneurodea Martynov, 1938). In Rasnitsyn, A. P. and Quicke, D. L. J., editors, *History of Insects*, pages 106–108. Kluwer Academic Publishers, The Netherlands.

Rasnitsyn, A. P. (2002e). 2.2.1.2.2. Superorder Hypoperlidea Martynov, 1928. Order Hypoperlida Martynov, 1928. In Rasnitsyn, A. P. and Quicke, D. L. J., editors, *History of Insects*, pages 111–115. Kluwer Academic Publishers, The Netherlands.

Rasnitsyn, A. P. (2002f). 2.2.1.2.4.1. Order Psocida Leach, 1815. The booklice (=Psocoptera Shipley, 1904 =Copeognatha Enderlein, 1903). In Rasnitsyn, A. P. and Quicke, D. L. J., editors, *History of Insects*, pages 128–131. Kluwer Academic Publishers, The Netherlands.

Rasnitsyn, A. P. (2002g). 2.2.1.3.1. Superorder Palaeomanteidea Handlirsch, 1906. Order Palaeomanteida Handlirsch, 1906 (=Miomoptera Martynov, 1927). In Rasnitsyn, A. P. and Quicke, D. L. J., editors, *History of Insects*, pages 161–164. Kluwer Academic Publishers, The Netherlands.

Rasnitsyn, A. P. (2002h). 2.2.1.3.3.4. Order Jurinida M. Zalessky, 1928 (=Glosselytrodea Martynov, 1938). In Rasnitsyn, A. P. and Quicke, D. L. J., editors, *History of Insects*, pages 189–192. Kluwer Academic Publishers, The Netherlands.

Rasnitsyn, A. P. (2002i). 2.2.1.3.5. Superorder Vespida Laicharting, 1781. Order Hymenoptera Linné, 1758 (=Vespida Laicharting, 1781). In Rasnitsyn, A. P. and Quicke, D. L. J., editors, *History of Insects*, pages 242–254. Kluwer Academic Publishers, The Netherlands.

Rasnitsyn, A. P. (2002j). 2.2.2. Infraclass Gryllones Laicharting, 1781. The Grylloneans (=Polyneoptera Martynov, 1938). In Rasnitsyn, A. P. and Quicke, D. L. J., editors, *History of Insects*, pages 254–262. Kluwer Academic Publishers, The Netherlands.

Rasnitsyn, A. P. (2002k). 2.2.2.0.1. Order Eoblattida Handlirsch, 1906 (=Cacurgida Handlirsch, 1906, =Protoblattodea Handlirsch, 1906). In Rasnitsyn, A. P. and Quicke, D. L. J., editors, *History of Insects*, pages 256–260. Kluwer Academic Publishers, The Netherlands.

Rasnitsyn, A. P. (2002l). Subclass Lepismatona Latreille, 1804. the wingless insects (=Thysanura Latreille 1796, *s.l.*). In Rasnitsyn, A. P. and Quicke, D. L. J., editors, *History of Insects*, pages 69–74. Kluwer Academic Publishers, The Netherlands.

Rasnitsyn, A. P. (2003). On skimming hypothesis of the insect flight origin. *Acta zoologica cracoviensia*, 46(suppl.- Fossil Insects):85–88.

Rasnitsyn, A. P. (2006). Ontology of evolution and methodology of taxonomy. *Paleontological Journal*, 40(Suppl. 6):S679–S737.

Rasnitsyn, A. P. (2008). Hymenopterous insects (Insecta: Vespida) in the Upper Jurassic deposits of Shar Teg, SW Mongolia. *Russian Entomological Journal*, 17(3):299–310.

Rasnitsyn, A. P., Ansorge, J., and Zessin, W. (2003). New hymenopterous insects (Insecta: Hymenoptera) from the Lower Toarcian (Lower Jurassic) of Germany. *Neues Jahrbuch für Geologie und Paläontologie, Abhandlungen*, 227(3):321–342.

Rasnitsyn, A. P., Ansorge, J., and Zhang, H.-C. (2006a). Ancestry of the orussoid wasps, with description of three new genera and species of Karatavitidae (Hymenoptera = Vespida: Karatavitoidea stat. nov.). *Insect Systematics & Evolution*, 37(2):179–190.

Rasnitsyn, A. P. and Aristov, D. S. (2004). Two new insects from the Upper Permian (Tatarian) of Belmont, New South Wales, Australia (Insecta: Hypoperlida: Anthracoptilidae = Permarrhaphidae; Grylloblattida: Sylvaphlebiidae). *Paleontological Journal*, 38(Suppl. 2):S158–S163.

Rasnitsyn, A. P., Aristov, D. S., Gorokhov, A. V., Rowland, J. M., and Sinitshenkova, N. D. (2004a). Important new insect fossils from Carrizo Arroyo and the Permo-Carboniferous faunal boundary. In Lucas, S. G. and Zeigler, K. E., editors, *Carboniferous-Permian Transition at Carrizo Arroyo, Central New Mexico*, pages 215–246. New Mexico Museum of Natural History and Science, Albuquerque, Bulletin 25.

Rasnitsyn, A. P., Basibuyuk, H. H., and Quicke, D. L. J. (2004b). A basal chalcidoid (Insecta: Hymenoptera) from the earliest Cretaceous or latest Jurassic of Mongolia. *Insect Systematics & Evolution*, 35(2):123–135.

Rasnitsyn, A. P. and Brothers, D. J. (2007). Two new hymenopteran fossils from the mid-Cretaceous of southern Africa (Hymenoptera: Jurapriidae, Evaniidae). *African Invertebrates*, 48(1):193–202.

Rasnitsyn, A. P. and Brothers, D. J. (2009). New genera and species of Maimetshidae (Hymenoptera: Stephanoidea s.l.) from the Turonian of Botswana, with comments on the status of the family. *African Invertebrates*, 50(1):191–204.

Rasnitsyn, A. P., Jarzembowski, E. A., and Ross, A. J. (1998). Wasps (Insecta: Vespida = Hymenoptera) from the Purbeck and Wealden (Lower Cretaceous) of southern England and their biostratigraphical and palaeoenvironmental significance. *Cretaceous Research*, 19:329–391.

Rasnitsyn, A. P. and Kovalev, O. V. (1988). Gall wasps from the early Cretaceous of Transbaikalia (Hymenoptera, Cynipoidea, Archaeocynipidae fam. n.) [in Russian]. *Vestnik zoologii*, 1988(1):18–21.

Rasnitsyn, A. P. and Kozlov, M. V. (1990). A new group of fossil insects: Scorpionflies with the adaptations of bugs and butterflies [in Russian]. *Doklady Akademii Nauk SSSR*, 310(4):973–976.

Rasnitsyn, A. P. and Krassilov, V. A. (2000). The first documented occurrence of phyllophagy in pre-Cretaceous insects: Leaf tissues in the gut of Upper Jurassic insects from southern Kazakhstan. *Paleontological Journal*, 34(3):301–309.

Rasnitsyn, A. P. and Martínez-Delclòs, X. (2000). Wasps (Insecta: Vespida = Hymenoptera) from the early Cretaceous of Spain. *Acta Geologica Hispanica*, 35(1-2):65–95.

Rasnitsyn, A. P. and Ross, A. J. (2000). A preliminary list of arthropod families present in the Burmese amber collection at The Natural History Museum, London. *Bulletin of The Natural History Museum, Geology Series*, 56(1):21–24.

Rasnitsyn, A. P., Sukatsheva, I. D., and Aristov, D. S. (2005). Permian insects of the Vorkuta Group in the Pechora Basin, and their stratigraphic implications. *Paleontological Journal*, 39(4):404–416.

Rasnitsyn, A. P., Zhang, H., and Wang, B. (2006b). Bizarre fossil insects: web-spinning sawflies of the genus *Ferganolyda* (Vespida, Pamphilioidea) from the Middle Jurassic of Daohugou, Inner Mongolia, China. *Palaeontology*, 49(4):907–916.

Rasnitsyn, A. P. and Zhang, H.-C. (2004a). Composition and age of the Daohugou hymenopteran (Insecta, Hymenoptera = Vespida) assemblage from Inner Mongolia, China. *Palaeontology*, 47(6):1507–1517.

Rasnitsyn, A. P. and Zhang, H.-C. (2004b). A new family, Daohugoidae fam. n., of syricomorph hymenopteran (Hymenoptera = Vespida) from the Middle Jurassic of Daohugou in Inner Mongolia (China). *Proceedings of the Russian Entomological Society*, 75(1):12–16.

Rasnitsyn, A. P. and Zherikhin, V. V. (2000). First fossil chewing louse from the Lower Cretaceous of Baissa, Transbaikalia (Insecta, Pediculida = Phthiriaptera, Saurodectidae fam.n.). *Russian Entomological Journal*, 8 [for 1999](4):253–255.

Rasnitsyn, A. P. and Zherikhin, V. V. (2002). 4.1 Impression fossils. In Rasnitsyn, A. P. and Quicke, D. L. J., editors, *History of Insects*, pages 437–444. Kluwer Academic Publishers, The Netherlands.

Rehn, A. C. (2003). Phylogenetic analysis of higher-level relationships of Odonata. *Systematic Entomology*, 28(2):181–239.

Ren, D. (1998). Late Jurassic Brachycera from northeastern China. *Acta Zootaxonomica Sinica*, 23(1):65–82.

Ren, D. (2002a). A new lacewing family (Neuroptera) from the Middle Jurassic of Inner Mongolia, China. *Entomologia Sinica*, 9(12):53–67.

Ren, D. (2002b). Progress in the study of Mesozoic fossil insects during the last decade in China. *Acta Entomologica Sinica*, 45(2):234–240.

Ren, D. and Engel, M. S. (2007). A split-footed lacewing and two epiosmylines from the Jurassic of China (Neuroptera). *Annales zoologici*, 57(2):211–219.

Ren, D. and Engel, M. S. (2008). Aetheogrammatidae, a new family of lacewings from the Mesozoic of China (Neuroptera: Myrmeleontiformia). *Journal of the Kansas Entomological Society*, 81(3):161–167.

Ren, D., Gao, K.-Q., Guo, Z.-G., Ji, S., Tan, J.-J., and Song, Z. (2002). Stratigraphic division of the Jurassic in the Daohugou area, Ningcheng, Inner Mongolia. *Geological Bulletin of China*, 21(8-9):584–591. In Chinese with English summary.

Ren, D. and Guo, Z.-G. (1995). A new genus and two new species of short-horned flies of Upper Jurassic from northeast China (Diptera: Eremochaetidae). *Entomologia Sinica*, 2(4):300–307.

Ren, D., Labandeira, C. C., Santiago-Blay, J. A., Rasnitsyn, A. P., Shih, C.-K., Bashkuev, A., Logan, M. A. V., Hotton, C. L., and Dilcher, D. (2009). A probable pollination mode before angiosperms: Eurasian, long-proboscid scorpionflies. *Science*, 326:840–847.

Ren, D., Liu, J.-Y., and Cheng, X.-D. (2003). A new hemeroscopid dragonfly from the Lower Cretaceous of Northeast China (Odonata: Hemeroscopidae). *Acta Entomologica Sinica*, 46(5):622–628.

Ren, D. and Makarkin, V. N. (2009). Ascalochrysidae Ű a new lacewing family from the Mesozoic of China (Insecta: Neuroptera: Chrysopoidea). *Cretaceous Research*, 30(5):1217–1222.

Ren, D., Nel, A., and Prokop, J. (2008). New early griffenfly, *Sinomeganeura huangheensis* from the late Carboniferous of northern China (Meganisoptera: Meganeuridae). *Insect Systematics & Evolution*, 39:223–229.

Ren, D. and Shih, C.-K. (2005). The first discovery of fossil eomeropids from China (Insecta, Mecoptera). *Acta Zootaxonomica Sinica*, 30(2):275–280.

Ren, D., Yin, J.-C., and Dou, W.-X. (1998). New planthoppers and froghoppers from the late Jurassic of northeast China (Homoptera: Auchenorrhyncha). *Acta Zootaxonomica Sinica*, 23(3):281–287.

Riek, E. F. (1976). An entomobryid collembolan (Hexapoda: Collembola) from the Lower Permian of Southern Africa. *Paleontologica Africana*, 19:141–143.

Rindal, D. S. A. . E. (2007). Phylogeny of the Mycetophiliformia, with proposal of the subfamilies Heterotrichinae, Ohakuneinae, and Chiletrichinae for the Rangomaramidae (Diptera, Bibionomorpha). *Zootaxa*, 1535:1–92.

Rognes, K. (1997). The Calliphoridae (blowflies) (Diptera: Oestroidea) are not a monophyletic group. *Cladistics*, 13:27–66.

Rohdendorf, B. B. (1991). *Fundamentals of Paleontology Volume 9: Arthropoda, Tracheata, Chelicerta*. Smithsonian Institution Libraries and The National Science Foundation, Washington D.C.

Ross, A. J. (2001). The cockroaches (Blattodea) of the Purbeck Limestone Group and Wealden Supergroup (Lower Cretaceous) of southern England p.59-60. In *2nd International Congress on Palaeoentomology, Krakow, abstract volume*.

Ross, A. J. and Jarzembowski, E. A. (1993). Arthropoda (Hexapoda; Insecta). In Benton, M. J., editor, *The Fossil Record 2*, pages 363–426. Chapman and Hall, London.

Ross, A. J., Nicholson, D. B., and Jarzembowski, E. A. (2013). Boltonocostidae nom. nov. (Insecta, Hypoperlida), a replacement name for Orthocostidae Bolton, 1912. *Bulletin of Zoological Nomenclature*, 70(4):291–292.

Ross, A. J. and York, P. V. (2000). A list of type and figured specimens of insects and other inclusions in Burmese amber. *Bulletin of The Natural History Museum, Geology Series*, 56(1):11–20.

Ross, A. J. and York, P. V. (2004). A catalogue of the type and figured specimens of Hexapoda from the Rhynie chert (early Devonian) at The Natural History Museum, London, UK. *Transactions of the Royal Society of Edinburgh: Earth Sciences*, 94(4):319–395.

Ross, E. S. (2003). EMBIA contributions to the biosystematics of the insect order Embiidina Part 5: A review of the family Anisembiidae with descriptions of new taxa. *Occasional Papers of the California Academy of Sciences*, 154:1–123.

Rowland, J. M. (1997). The late Paleozoic insect assemblage at Carrizo Arroyo, New Mexico p.1-7. In Lucas, S. G., Estep, J. W., Williamson, T. E., and Morgan, G. S., editors, *New Mexico's Fossil Record 1*, volume Bulletin 11, page 143. New Mexico Museum of Natural History and Science, Albuquerque.

Rust, J. (1998). Biostratinomie von Insekten aus der Fur-Formation von Dänemark (Moler, oberes Paleozän / unteres Eozän). *Paläontologische Zeitschrift*, 72(1/2):41–58.

Rust, J., Petrulevičius, J. F., and Nel, A. (2008). The first damselflies from the lowermost Eocene of Denmark, with a description of a new subfamily (Odonata, Zygoptera: Dysagrionidae). *Paleontology*, 51(3):709–713.

Sabrosky, C. W., Thompson, F. C., and Evenhuis, N. L. (1999). Family-group names in Diptera. *Myia*, 10:1–576.

Schliephake, G. (2001). Weitere neue Funde fossiler Fransenflügler aus dem Baltischen Bernstein (Insecta, Thysanoptera). *Mitteilungen aus dem Geologisch-Paläontologischen Institut der Universität Hamburg*, 85:197–201.

Schlüter, T. (2003). Fossil insects in Gondwana - localities and palaeodiversity trends. *Acta zoologica cracoviensia*, 46(suppl.- Fossil Insects):345–371.

Schmied, H., Wappler, T., and Kolibáč, J. (2009). A new bark-gnawing beetle (Coleoptera, Trogossitidae) from the middle Eocene of Europe, with a checklist of fossil Trogossitidae. *Zootaxa*, 1993:17–26.

Schneider, J. (1983). Die Blattodea (Insecta) des Paläzoikums, Teil 1: Systematik, Ökologie und Biostratigraphie. *Freiberger Forschungenshefte, Reihe C*, 382:106–145.

Schneider, J. (1984). Die Blattodea (Insecta) des Palaozoikums Teil II: Morphogenese der Flugelstrukturen und Phylogenie. *Freiberger Forschungenshefte, Reihe C*, 391:5–34.

Schneider, J. W., Lucas, S. G., and Rowland, J. M. (2004). The Blattida (Insecta) fauna of Carrizo Arroyo, New Mexico - biostratigraphic link between marine and nonmarine Pennsylvanian/Permian boundary profiles. In Lucas, S. G. and Zeigler, K. E., editors, *Carboniferous-Permian Transition at Carrizo Arroyo, Central New Mexico*, volume Bulletin 25, pages 247–261. New Mexico Museum of Natural History and Science, Albuquerque.

Schneider, J. W. and Werneburg, R. (2006). Insect biostratigraphy of the Euramerican continental late Pennsylvanian and early Permian. In Lucas, S. G., Cassinis, G., and Schneider, J. W., editors, *Non-Marine Permian Biostratigraphy and Biochronology*, pages 325–336. Geological Society of London Special Publications 265.

Selden, P. A. and Penney, D. (2009). A fossil spider (Araneae: Pisauridae) of Eocene age from Horsefly, British Columbia, Canada. *Contributions to Natural History*, 12:1269–1282.

Sharov, A. G. and Sinitshenkova, N. D. (1977). Novyye Paleodictyoptera s territorii SSSR. [new Palaeodictyoptera from the USSR.]. *Paleontologicheskii Zhurnal*, 1977:48–63.

Shcherbakov, D. E. (1988). New Mesozoic Homoptera. *Sovmestnaya Sovetsko-Mongolskaya Paleontologischeskaya Ekspeditsiya Trudy*, 33:60–63.

Shcherbakov, D. E. (1992). The earliest leafhoppers (Hemiptera: Karajassidae n. fam.) from the Jurassic of Karatau. *Neues Jahrbuch für Geologie und Paläontologie, Monatshefte*, 1992(1):39–51.

Shcherbakov, D. E. (2000a). The most primitive whiteflies (Hemiptera; Aleyrodidae; Bernaeinae subfam. nov.) from the Mesozoic of Asia and Burmese amber, with an overview of Burmese amber hemipterans. *Bulletin of The Natural History Museum, Geology Series*, 56(1):29–37.

Shcherbakov, D. E. (2000b). Permian faunas of Homoptera (Hemiptera) in relation to phytogeography and the Permo-Triassic Crisis. *Paleontological Journal*, 34 (suppl.-3):S251–S267.

Shcherbakov, D. E. (2002). 2.2.2.2.3 Order Forficulida Latreille, 1810. The earwigs and protelytropterans (=Dermaptera DeGeer, 1773 +Protelytroptera Tillyard, 1931). In Rasnitsyn, A. P. and Quicke, D. L. J., editors, *History of Insects*, pages 288–291. Kluwer Academic Publishers, The Netherlands.

Shcherbakov, D. E. (2006). The earliest find of Tropiduchidae (Homoptera: Auchenorrhyncha), representing a new tribe, from the Eocene of Green River, USA, with notes on the fossil record of higher Fulgoroidea. *Russian Entomological Journal*, 15(3):315–322.

Shcherbakov, D. E. (2007a). Extinct four-winged precoccids and the ancestry of scale insects and aphids (Hemiptera). *Russian Entomological Journal*, 16(1):47–62.

Shcherbakov, D. E. (2007b). An extraordinary new family of Cretaceous planthoppers (Homoptera: Fulgoroidea). *Russian Entomological Journal*, 16(2):139–154.

Shcherbakov, D. E. (2007c). Mesozoic spider mimics Ű Cretaceous Mimarachnidae fam. n. (Homoptera: Fulgoroidea). *Russian Entomological Journal*, 16(3):259–264.

Shcherbakov, D. E. (2008a). Insect recovery after the Permian/Triassic crisis. *Alavesia*, 2:125–131.

Shcherbakov, D. E. (2008b). Madygen, Triassic Lagerstätte number one, before and after Sharov. *Alavesia*, 2:113–124.

Shcherbakov, D. E. (2008c). Mesozoic Velocipedinae (Nabidae s.l.) and Ceresopseidae (Reduvioidea), with notes on the phylogeny of Cimicomorpha (Heteroptera). *Russian Entomological Journal*, 16 [for 2007](4):401–414.

Shcherbakov, D. E. (2008d). On Permian and Triassic insect faunas in relation to biogeography and the Permian-Triassic crisis. *Paleontological Journal*, 42(1):15–31.

Shcherbakov, D. E. (2009). Review of the fossil and extant genera of the cicada family Tettigarctidae (Hemiptera: Cicadoidea). *Russian Entomological Journal*, 17 [for 2008](4):343–348.

Shcherbakov, D. E., Lukashevitch, E. D., and Blagoderov, V. A. (1995). Triassic Diptera and initial radiation of the order. *An International Journal of Dipterological Research*, 6(2):75–115.

Shcherbakov, D. E., Makarkin, V. N., Aristov, D. S., and Vasilenko, D. V. (2009). Permian insects from the Russky Island, South Pimorye. *Russian Entomological Journal*, 18(1):7–16.

Shcherbakov, D. E. and Popov, Y. A. (2002). 2.2.1.2.5. Superorder Cimicidea Laicharting, 1781 Order Hemiptera Linné, 1758. The bugs, cicadas, plantlice, scale insects, etc. (=Cimicida Laicharting, 1781, =Homoptera Leach, 1815 + Heteroptera Latreille, 1810). In Rasnitsyn, A. P. and Quicke, D. L. J., editors, *History of Insects*, pages 143–157. Kluwer Academic Publishers, The Netherlands.

Shcherbakov, D. E. and Wegierek, P. (1991). Creaphididae, a new and the oldest aphid family from the Triassic of middle Asia. *Psyche*, 98(1):81–85.

Shih, C.-K., Liu, C.-X., and Ren, D. (2009). The earliest fossil record of pelecinid wasps (Insecta, Hymenoptera, Proctotrupoidea, Pelecinidae) from Inner Mongolia, China. *Annals of the Entomological Society of America*, 102(1):20–38.

Shmakov, A. S. (2008). The Jurassic thrips *Liassothrips crassipes* (Martynov, 1927) and its taxonomic position in the order Thysanoptera (Insecta). *Paleontological Journal*, 42(1):47–52.

Shmakov, A. S. (2009). The oldest members of the families Aeolothripidae and Thripidae (Insecta: Thysanoptera) from the Lower Cretaceous of Transbaikalia. *Paleontological Journal*, 43(4):428–432.

Silvestri, F. (1913). Descrizione di un nuovo ordine di Insetti. *Bollettino del Laboratio di zoologia generale e agraria della R. scuola superiore di agricultura in Portici*, 7:193–209.

Simon, E. (1879). Essai d'une classification des Opiliones Mecostethi. *Annales de la Société entomologique de Belgique*, 22:183–241.

Simon, E. (1886). Étude sur les crustacés du sous-ordre des phyllopodes, I, revision des espécies Françaises. *Annales de la Société Entomologique de France, Ser. 6*, 6:393–460.

Simutnik, S. A. and Perkovsky, E. E. (2006). A description of the encyrtid male (Hymenoptera, Chalcidoidea, Encyrtidae) with archaic structure of metasoma from Rovno amber. *Vestnik zoologii*, 40(3):283–286.

Sinitshenkova, N. D. (1981a). A new species of the Tchirkovaeidae (Insecta, Dictyoneurida) from the Upper Carboniferous of the Tunguska Basin. *Paleontological Journal*, 15(1):121–123.

Sinitshenkova, N. D. (1981b). The peculiar "dipterous" insects from the Permian of Arkhangelsk district and Urals (Hypoperlida, Eukulojidae). *Bulletin of Moscow Society of Naturalists, Biological Series*, 56(3):116–126. in Russian.

Sinitshenkova, N. D. (1987). Istoricheskoe razvitie vesnyanok [Historical development of stoneflies]. *Trudy Paleontologicheskogo instituta Akademiia nauk SSSR*, 221:1–143. In Russian.

Sinitshenkova, N. D. (1989). New Mesozoic mayflies (Ephemerida) from Mongolia. *Paleontological Journal*, 23(3):26–37.

Sinitshenkova, N. D. (1990). New Mesozoic stoneflies from Asia. *Paleontological Journal*, 24(3):62–70.

Sinitshenkova, N. D. (1992). New upper Mesozoic stone flies from Yakutia (Insecta: Perlida=Plecoptera). *Paleontological Journal*, 26(3):43–55.

Sinitshenkova, N. D. (1994). A new family Aykhalidae from the Upper Palaeozoic of Yakutia Sakha (Insecta: Mischopteridae = Megasecoptera). *Paleontological Journal*, 27 (1993)(1A):131–134.

Sinitshenkova, N. D. (1998). New upper Mesozoic stoneflies from central Transbaikalia (Insecta, Perlida = Plecoptera). *Paleontological Journal*, 32(2):167–173.

Sinitshenkova, N. D. (1999). A new mayfly species of the extant genus *Neoephemera* from the Eocene of North America (Insecta: Ephemerida = Ephemeroptera: Neoephemeridae. *Paleontological Journal*, 33(4):403–405.

Sinitshenkova, N. D. (2000a). The first fossil prosopistomatid mayfly from Burmese amber (Ephemeroptera; Prosopistomatidae). *Bulletin of The Natural History Museum, Geology Series*, 56(1):25–28.

Sinitshenkova, N. D. (2000b). New Jersey amber mayflies: the first North American Mesozoic members of the order (Insecta; Ephemeroptera. In Grimaldi, D. A., editor, *Studies on Fossils in Amber, with Particular Reference to the Cretaceous of New Jersey*, pages 111–125. Backhuys Publishers, Leiden, The Netherlands.

Sinitshenkova, N. D. (2000c). A review of Triassic mayflies, with a description of new species from western Siberia and Ukraine (Ephemerida = Ephemeroptera). *Paleontological Journal*, 34(Suppl. 3):S275–S283.

Sinitshenkova, N. D. (2002a). 2.2.1.2.3 Superorder Dictyoneuridea Handlirsch, 1906 (=Palaeodictyopteroidea). In Rasnitsyn, A. P. and Quicke, D. L. J., editors, *History of Insects*, pages 115–124. Kluwer Academic Publishers, The Netherlands.

Sinitshenkova, N. D. (2002b). 2.2.2.2.2. Order Perlida Latreille, 1810. The Stoneflies (=Plecoptera Burmeister, 1839). In Rasnitsyn, A. P. and Quicke, D. L. J., editors, *History of Insects*, pages 281–287. Kluwer Academic Publishers, The Netherlands.

Sinitshenkova, N. D. (2002c). 3.3 Ecological history of the aquatic insects. In Rasnitsyn, A. P. and Quicke, D. L. J., editors, *History of Insects*, pages 388–426. Kluwer Academic Publishers, The Netherlands.

Sinitshenkova, N. D. (2002d). New late Mesozoic mayflies from the Shar-Teeg locality, Mongolia (Insecta, Ephemerida=Ephemeroptera). *Paleontological Journal*, 36(3):270–276.

Sinitshenkova, N. D. (2003). Main ecological events in aquatic insects history. *Acta zoologica cracoviensia*, 46(suppl. - Fossil Insects):381–392.

Sinitshenkova, N. D. (2004). New stoneflies of the family Palaeonemouridae from the Upper Permian of Udmurtiya and the Orenburg Region (Insecta: Perlida = Plecoptera). *Paleontological Journal*, 38(Suppl. 2):S164–S172.

Sinitshenkova, N. D., Marchal-Papier, F., Grauvogel-Stamm, L., and Gall, J.-C. (2005). The Ephemeridea (Insecta) from the Grès à Voltzia (early Middle Triassic) of the Vosges (NE France). *Paläontologische Zeitschrift*, 79(3):377–397.

Skalski, A. W. (1992). The families Nepticulidae and Thyrididae in Baltic amber (Lepidoptera). *Nota lepidopterologica Supplement*, 4:144–145.

Smith, V. S., Dalgleish, R. C., and Cruickshank, R. H. (2007). Fossil lice reconsidered Ű erratum. *Systematic Entomology*, 32(1):196.

Sobczyk, T. and Kobbert, M. J. (2009). Die Psychidae des baltischen Bernsteins. *Nota lepidopterologica*, 32(1):13–22.

Solórzano Kraemer, M. M. (2007). Systematic, palaeoecology, and palaeobiogeography of the insect fauna from the mexican amber. *Palaeontographica Abteilung A*, 282(1-6):1–133.

Soriano, C. (2009). First record of the family Belidae (Insecta, Coleoptera) in amber. New genus and species from the uppermost Albian amber of France. *Geodiversitas*, 31(1):99–104.

Soriano, C., Delclòs, X., and Ponomarenko, A. G. (2007). Beetle associations (insecta: Coleoptera) from the barremian (lower cretaceous) of spain. *Alavesia*, 1:81–88.

Soriano, C., Gratshev, V. G., and Delclòs, X. (2006a). New early Cretaceous weevils (Insecta, Coleoptera, Curculionoidea) from El Montsec, Spain. *Cretaceous Research*, 27(4):555–564.

Soriano, C., Kirejtshuk, A. G., and Delclòs, X. (2006b). The Mesozoic Laurasian family Parandrexidae (Insecta: Coleoptera), new species from the Lower Cretaceous of Spain. *Comptes Rendus Palevol*, 5(6):779–784.

Spahr, U. (1990). Ergänzungen und Berichtigungen zu R. Keilbach's Bibliographie und Liste der Bernsteinfossilien - "Apterygota". *Stuttgarter Beiträge zur Naturkunde Serie B (Geologie und Paläontologie)*, 166:1–23.

Spahr, U. (1992). Ergänzungen und Berichtigungen zu R. Keilbachs Bibliographie und Liste der Bernsteinfossilien - Klasse Insecta. (Ausgenommen: "Apterygota", Hemipteroidea, Coleoptera, Hymenoptera, Mecopteroidea). *Stuttgarter Beiträge zur Naturkunde Serie B (Geologie und Paläontologie)*, 182:1–102.

Staniczek, A. H. (2007). 11.4 Ephemeroptera: mayflies. In *The Crato Fossil Beds of Brazil: Window into an Ancient World*, pages 163–184. Cambridge University Press.

Staniczek, A. H. and Bechly, G. (2007). 11.2 Apterygota: primarily wingless insects. In Martill, D. M., Bechly, G., and Loveridge, R. F., editors, *The Crato Fossil Beds of Brazil: Window into an Ancient World*, pages 149–154. Cambridge University Press.

Storozhenko, S. Y. (1988). New and little known Mesozoic Grylloblattids. *Paleontological Journal*, 22(4):45–52.

Storozhenko, S. Y. (1990). New Permian and Mesozoic insects (Insecta, Grylloblattida: Blattogryllidae, Geinitziidae) from Asia. *Paleontological Journal*, 24(4):53–61.

Storozhenko, S. Y. (1992a). A new family of Triassic grylloblattids from Central Asia. *Spixiana*, 15(1):67–73.

Storozhenko, S. Y. (1992b). New Mesozoic grylloblattid insects (Grylloblattida) from Central Asia. *Paleontological Journal*, 26(1):85–95.

Storozhenko, S. Y. (1992c). Permian fossil insects of north-east Europe: new and little-known Ideliidae (Insecta, Plecopteroidea, Grylloblattida). *Entomologica Fennica*, 3(1):21–39.

Storozhenko, S. Y. (1994). New Triassic grylloblattids from Kirghizia. *Spixiana*, 17(1):27–35.

Storozhenko, S. Y. (1996a). New Triassic Mesorthopteridae. *Spixiana*, 19(1):115–127.

Storozhenko, S. Y. (1996b). New Upper Carboniferous grylloblattids (Insecta, Grylloblattida) from Siberia. *Far Eastern Entomologist*, 26:18–20.

Storozhenko, S. Y. (1997). Classification of the order Grylloblattida (Insecta), with description of new taxa. *Far Eastern Entomologist*, 42:1–20.

Storozhenko, S. Y. (2002). 2.2.2.2.1. Order Grylloblattida Walker, 1914 (=Notoptera Crampton, 1915, =Grylloblattodea Brues et Melander, 1932, +Protorthoptera Handlirsch, 1906, =Paraplecoptera Martynov, 1925, +Protoperlaria Tillyard, 1928). In Rasnitsyn, A. P. and Quicke, D. L. J., editors, *History of Insects*, pages 278–281. Kluwer Academic Publishers, The Netherlands.

Storozhenko, S. Y. and Novokshonov, V. G. (1994). Revision of the Permian family Sojanoraphidiidae (Grylloblattida). *Russian Entomological Journal*, 3(3-4):37–39.

Storozhenko, S. Y. and Vršanský, P. (1995). New fossil family of the Order Grylloblattida (Insecta: Plecopteroidea) from Asia. *Far Eastern Entomologist*, 19:1–4.

Stuke, J.-H. (2003). Eine neue Blasenkopffliege der Gattung *Palaeomyopa* Meunier aus dem Baltischen Bernstein (Diptera: Conopidae). *Studia dipterologica*, 10(1):91–96.

Sturm, H. and Poinar, G. O. (1998). *Cretaceomachilis libanensis*, the oldest known bristletail of the family Meinertellidae (Machiloidea, Archaeognatha, Insecta) from the Lebanese amber. *Deutsche entomologische Zeitschrift*, 45(1):43–48.

Sukatsheva, I. D. (1985). Yurskie rucheiniki yuzhnoi Sibiri [Jurassic caddisflies of Southern Siberia]. *Trudy Paleontologicheskogo Instituta*, 211:115–119. in Russian.

Sukatsheva, I. D. (2000). New fossil caddis flies (Trichoptera) from the Shar-Teg locality in Mongolia. *Paleontological Journal*, 34(Suppl. 3):S347–S351.

Sukatsheva, I. D., Beattie, R., and Mostovski, M. B. (2007). *Permomerope natalensis* sp. n. from the Lopingian of South Africa, and a redescription of the type species of *Permomerope* (Insecta: Trichoptera). *African Invertebrates*, 48(2):245–251.

Sukatsheva, I. D. and Jarzembowski, E. A. (2001). Fossil caddisflies (Insecta: Trichoptera) from the early Cretaceous of southern England II. *Cretaceous Research*, 22(6):685–694.

Sukatsheva, I. D. and Novokshonov, V. G. (1998). A new family of scorpionflies from the Mesozoic of Yakutia (Insecta; Mecoptera, Sibirioithaumatidae fam. nov.). *Paleontological Journal*, 32(6):596–597.

Sukatsheva, I. D. and Rasnitsyn, A. P. (2004). Jurassic insects (Insecta) from the Sai-Sagul locality (Kyrgyzstan, Southern Fergana). *Paleontological Journal*, 38(2):182–186.

Sun, J.-H., Ren, D., and Huang, J.-D. (2007a). Current knowledge of research on Mecoptera fossils in china. *Acta Zootaxonomica Sinica*, 32(4):881–889.

Sun, J.-H., Ren, D., and Shih, C.-K. (2007b). Middle Jurassic Mesopanorpodidae from Daohugou, Inner Mongolia, China (Insecta, Mecoptera). *Acta Zootaxonomica Sinica*, 32(4):865–874.

Svenson, G. J. and Whiting, M. F. (2009). Reconstructing the origins of praying mantises (Dictyoptera, Mantodea): the roles of Gondwanan vicariance and morphological convergence. *Cladistics*, 25(5):468–514.

Szwedo, J. (2006). First fossil record of Cedusini in the Eocene Baltic amber with notes on the tribe (Hemiptera: Fulgoromorpha: Derbidae). *Russian Entomological Journal*, 15(3):327–333.

Szwedo, J. (2007a). 11.13 Fulgoromorpha: planthoppers. In Martill, D. M., Bechly, G., and Loveridge, R. F., editors, *The Crato Fossil Beds of Brazil: Window into an Ancient World*, pages 297–313. Cambridge University Press.

Szwedo, J. (2007b). Nymphs of a new family Neazoniidae fam. n. (Hemiptera: Fulgoromorpha: Fulgoroidea) from the Lower Cretaceous Lebanese amber. *African Invertebrates*, 48(1):127–143.

Szwedo, J. (2008a). Achilidae from the Eocene Baltic amber. *Bulletin of Insectology*, 61(1):109–110.

Szwedo, J. (2008b). Distributional and palaeoecological pattern of the Lower Cretaceous Mimarachnidae (Hemiptera: Fulgoromorpha). *Entomologia Generalis*, 31(3):231–242.

Szwedo, J. (2008c). A new tribe of Dictyopharidae planthoppers from Eocene Baltic amber (Hemiptera: Fulgoromorpha: Fulgoroidea), with a brief review of the fossil record of the family. *Palaeodiversity*, 1:75–85.

Szwedo, J. (2008d). Paradise lost? Ũ cretaceous and palaeogene diversification of planthoppers and leafhoppers. *Bulletin of Insectology*, 61(1):111–112.

Szwedo, J. (2009). First discovery of Neazoniidae (Hemiptera: Fulgoromorpha) in the early Cretaceous Archingeay amber of SW France. *Geodiversitas*, 31(1):105–116.

Szwedo, J., Bourgoin, T., and Lefèbvre, F. (2004). *Fossil Planthoppers (Hemiptera: Fulgoromorpha) of the World. An annotated catalogue with notes on Hemiptera classification.* Studio 1, Warsaw.

Szwedo, J. and Wappler, T. (2006). New planthoppers (hemiptera: Fulgoromorpha) from the middle eocene messel maar. *Annales zoologici*, 56(3):555–566.

Szwedo, J. and Żyła, D. (2009). New Fulgoridiidae genus from the Upper Jurassic Karatau deposits, Kazakhstan (Hemiptera: Fulgoromorpha: Fulgoroidea). *Zootaxa*, 2281:40–52.

Tan, J.-J. and Ren, D. (2007). Two exceptionally well-preserved catiniids (Coleoptera: Archostemata: Catiniidae) from the late Mesozoic of northeastern China. *Annals of the Entomological Society of America*, 100(5):666–672.

Tan, J.-J. and Ren, D. (2009). *Mesozoic archostematan fauna from China.* Science Press, Beijing.

Tan, J.-J., Ren, D., and Shih, C.-K. (2007). New beetles (insecta: Coleoptera: Archostemata) from the late mesozoic of north china. *Annales zoologici*, 57(2):231–247.

Tasch, P. and Zimmerman, J. R. (1962). The Asthenohymen-Delopterum bed - a new Leonardian insect horizon in the Wellington of Kansas and Oklahoma. *Journal of Paleontology*, 36:1319–1333.

Thomson, C. G. (1859). *Skandinaviens Coleoptera, synoptiskt bearbetade, Vol. 1.* Berlingska Boktryckeriet, Lund.

Tilgner, E. H. (2001). The fossil record of Phasmida (Insecta: Neoptera). *Insect Systematics & Evolution*, 31(4):473–480.

van Dijk, D. E. and Geertsema, H. (1999). Permian insects from the Beaufort Group of Natal, South Africa. *Annals of the Natal Museum*, 40:137–171.

van Dijk, D. E. and Geertsema, H. (2004). A new genus of Permian Plecoptera (*Afroperla*) from KwaZulu-Natal, South Africa. *African Entomology*, 12(2):268–270.

Vasilenko, D. V. (2005). New damselflies (Odonata: Synlestidae, Hemiphlebiidae) from the Mesozoic Transbaikalian locality of Chernovski Kopi. *Paleontological Journal*, 39(3):280–283.

Vasilenko, D. V. and Rasnitsyn, A. P. (2007). Fossil ovipositions of dragonflies: review and interpretation. *Paleontological Journal*, 41(11):1156–1161.

Vilhelmsen, L. (2004). The old wasp and the tree: fossils, phylogeny and biogeography in the Orussidae (Insecta, Hymenoptera). *Biological Journal of the Linnean Society*, 82(2):139–160.

von Tschirnhaus, M. and Hoffeins, C. (2009). Fossil flies in Baltic amber - insights in the diversity of Tertiary Acalyptratae (Diptera, Schizophora), with new morphological characters and a key based on 1,000 collected inclusions. *Denisia*, 26:171–212.

Vršanský, P. (2000). Decreasing variability - from the Carboniferous to the present! (Validated on independent lineages of Blattaria). *Paleontological Journal*, 34(Suppl. 3):S374–S379.

Vršanský, P. (2002a). *Jantarimantis* nom. nov. and Jantarimantidae nom. nov., new replacement names for the genus *Archimantis* Vršanský, 2002, and the family Archimantidae vršanský, 2002 (Insecta, Mantodea). *AMBA projekty*, 6(2):1.

Vršanský, P. (2002b). Origin and the early evolution of mantises. *AMBA projekty*, 6(1):1–16.

Vršanský, P. (2003a). *Phyloblatta grimaldii* sp. nov. - a new Triassic cockroach (Insecta: Blattaria) from Virginia. *Entomological Problems*, 32(1-2):51–53.

Vršanský, P. (2003b). Umenocoleoidea - an amazing lineage of aberrant insects (Insecta, Blattaria). *AMBA projekty*, 7(1):1–32.

Vršanský, P. (2005). Lower Cretaceous cockroaches and mantids (Insecta: Blattaria, Mantodea) from the Sharin-Gol in Mongolia. *Entomological Problems*, 35(2):163–167.

Vršanský, P. (2007). Jumping cockroaches (Blattaria, Skokidae fam. n.) from the late Jurassic of Karatau in Kazakhstan. *Biologia*, 62(5):588–592.

Vršanský, P. (2008a). Central ocellus of extinct cockroaches (Blattida: Caloblattinidae). *Zootaxa*, 1958:41–50.

Vršanský, P. (2008b). Mesozoic relative of the common synanthropic German cockroach (Blattodea). *Deutsche entomologische Zeitschrift*, 55(2):215–221.

Vršanský, P. (2008c). New blattarians and a review of dictyopteran assemblages from the Lower Cretaceous of Mongolia. *Acta Palaeontologica Polonica*, 53(1):129–136.

Vršanský, P. (2009). Albian cockroaches (Insecta, Blattida) from French amber of Archingeay. *Geodiversitas*, 31(1):73–98.

Vršanský, P. and Ansorge, J. (2001). New Lower Cretaceous polyphagid cockroaches from Spain (Blattaria, Polyphagidae, Vitisminae subfam. nov.). *Cretaceous Research*, 22(2):157–162.

Vršanský, P. and Ansorge, J. (2007). Lower Jurassic cockroaches (Insecta: Blattaria) from Germany and England. *African Invertebrates*, 48(1):103–126.

Vršanský, P., Liang, J.-H., and Ren, D. (2009). Advanced morphology and behaviour of extinct earwig-like cockroaches (Blattida: Fuziidae fam. nov.). *Geologica Carpathica*, 60(6):449–462.

Vršanský, P., Vishniakova, V. N., and Rasnitsyn, A. P. (2002). 2.2.2.1.1. Order Blattida Latreille, 1810. The cockroaches (=Blattodea Brunner von Wattenvill, 1882). In Rasnitsyn, A. P. and Quicke, D. L. J., editors, *History of Insects*, pages 263–270. Kluwer Academic Publishers, The Netherlands.

Wagner, R., Barták, M., Borkent, A., Courtney, G., Goddeeris, B., Haenni, J.-P., Knutson, L., Pont, A., Rotheray, G. E., Rozkošný, R., Sinclair, B., Woodley, N., Zatwarnicki, T., and Zwick, P. (2008). Global diversity of dipteran families (Insecta Diptera) in freshwater (excluding Simulidae, Culicidae, Chironomidae, Tipulidae and Tabanidae). *Hydrobiologia*, 595(1):489–519.

Wagner, R., Hoffeins, C., and Hoffeins, H. W. (2000). A fossil nymphomyiid (Diptera) from the Baltic and Bitterfelder amber. *Systematic Entomology*, 25(1):115–120.

Wang, B., Ponomarenko, A. G., and Zhang, H.-C. (2009a). A new coptoclavid larva (Coleoptera: Adephaga: Dytiscoidea) from the Middle Jurassic of China, and its phylogenetic implication. *Paleontological Journal*, 43(6):652–659.

Wang, B., Szwedo, J., and Zhang, H.-C. (2009b). Jurassic Progonocimicidae (Hemiptera) from China and phylogenetic evolution of Coleorrhyncha. *Science in China Series D: Earth Sciences*, 52(12):1953–1961.

Wang, B., Zhang, H.-C., and Fang, Y. (2006a). Some Jurassic Palaeontinidae (Insecta, Hemiptera) from Daohugou, Inner Mongolia, China. *Palaeoworld*, 15(1):115–125.

Wang, B., Zhang, H.-C., and Szwedo, J. (2009c). Jurassic Palaeontinidae from China and the higher systematics of Palaeontinoidea (Insecta: Hemiptera: Cicadomorpha). *Palaeontology*, 52(1):53–64.

Wang, M.-X., Zhao, Y.-Y., and Ren, D. (2009d). New fossil caddisfly from Middle Jurassic of Daohugou, Inner Mongolia, China (Trichoptera: Philopotamidae). *Progress in Natural Science*, 19(10):1427–1431.

Wang, Y., Ren, D., Liang, J.-H., Liu, Y.-S., and Wang, Z.-H. (2006b). The fossil Homoptera of China: a review of present knowledge. *Acta Zootaxonomica Sinica*, 31(2):294–303.

Wappler, T. (2003). Die Insekten aus dem Mittel-Eozän des Eckfelder Maares, Vulkaneifel. *Mainzer Naturwissenschafliches Archiv, Beiheft*, 27:1–234.

Wappler, T. and Ben-Dov, Y. (2008). Preservation of armoured scale insects on angiosperm leaves from the Eocene of Germany. *Acta Palaeontologica Polonica*, 53(4):627–634.

Wappler, T., Engel, M. S., and Haas, F. (2005). The earwigs (Dermaptera: Forficulidae) from the middle Eocene Eckfeld maar, Germany. *Polskie Pismo Entomologiczne*, 74(3):227–250.

Wappler, T. and Petrulevičius, J. F. (2007). Priscalestidae, a new damselfly family (Odonata: Lestinoidea) from the Middle Eocene Eckfeld maar of Germany. *Alavesia*, 1:69–73.

Wappler, T., Smith, V. S., and Dalgleish, R. C. (2004). Scratching an ancient itch: an Eocene bird louse fossil. *Proceedings of the Royal Society of London, B*, 271(Supplement 5):S255–S258.

Ware, J. L., Litman, J., Klass, K.-D., and Spearman, L. A. (2008). Relationships among the major lineages of Dictyoptera: the effect of outgroup selection on dictyopteran tree topology. *Systematic Entomology*, 33(3):429–450.

Wedmann, S., Bradler, S., and Rust, J. (2007). The first fossil leaf insect: 47 million years of specialized cryptic morphology and behavior. *Proceedings of the National Academy of Sciences of the United States of America*, 104(2):565–569.

Wedmann, S. and Makarkin, V. N. (2007). A new genus of Mantispidae (Insecta: Neuroptera) from the Eocene of Germany, with a review of the fossil record and palaeobiogeography of the family. *Zoological Journal of the Linnean Society*, 149(4):701–716.

Wegierek, P. (1989). New species of Mesozoic aphids (Shaposhnikoviidae, Homoptera). *Paleontological Journal*, 23(4):40–49.

Wegierek, P. (1991). Cretaceous aphids of the Family Canadaphididae (Hemiptera, Aphidomorpha). *Paleontologicheskii Zhurnal*, 1991(2):114–115.

Wegierek, P. and Peñalver, E. (2002). Fossil representatives of the family Greenideidae (Hemiptera, Aphidoidea) from the Miocene of Europe. *Geobios*, 35(6):745–757.

Wegrzynowicz, P. (2007). Systematic position of the genus *Tarrodacne* Zhang, 1989 (Coleoptera: Helotidae non Erotylidae). *Annales Zoologici*, 57(4):757–758.

Weitschat, W. and Wichard, W. (2002). *Atlas of plants and animals in Baltic amber*. Verlag Dr. Friedrich Pfeil, München.

Whalley, P. E. S. (1985). The systematics and palaeogeography of the Lower Jurassic insects of Dorset, England. *Bulletin of the British Museum (Natural History), Geology*, 39(3):107–189.

Whalley, P. E. S. (1988). Mesozoic Neuroptera and Raphidioptera (Insecta) in Britain. *Bulletin of the British Museum (Natural History), Geology*, 44(1):45–63.

Whalley, P. E. S. and Jarzembowski, E. A. (1985). Fossil insects from the Lithographic Limestone of Montsech (late Jurassic-early Cretaceous), Lerida Province, Spain. *Bulletin of the British Museum (Natural History), Geology*, 38(5):381–412.

White, R. D. (1995). A type catalog of fossil invertebrates (arthropoda: Hexapoda) in the yale peabody museum. *Postilla*, 209:1–55.

Whiting, M. F., Whiting, A. S., Hastriter, M. W., and Dittmar, K. (2008). A molecular phylogeny of fleas (Insecta: Siphonaptera): origins and host associations. *Cladistics*, 24:1–31.

Wichard, W., Chatterton, C., and Ross, A. (2005). Corydasialidae fam. n. (Megaloptera) from Baltic amber. *Insect Systematics & Evolution*, 36:279–283.

Wichard, W., Gröhn, C., and Seredszus, F. (2009). *Aquatic insects in Baltic amber, Wasserinsketen in Baltischen Bernstein*. Verlag Kessel.

Wichard, W. and Lüer, C. (2003). *Phylocentropus swolenskyi* n. sp., eine Köcherfliege aus dem New Jersey Bernstein (Trichoptera, Dipseudopsidae). *Mitteilungen aus dem Geologisch-Paläontologischen Institut der Universität Hamburg*, 87:162–169.

Wichard, W., Solórzano Kraemer, M. M., and Luer, C. (2006). First caddisfly species from Mexican amber (Insecta: Trichoptera. *Zootaxa*, 1378:37–48.

Wichard, W. and Weitschat, W. (1996). Wasserinsketen im Bernstein - eine paläobiologische Studie. *Entomologische Mitteilungen aus dem Löbbecke-Museum und Aquazoo*, 4:1–122.

Willkommen, J. and Grimaldi, D. A. (2007). 11.20 Diptera: true flies, gnats and crane flies. In *The Crato Fossil Beds of Brazil*, pages 369–387. Cambridge University Press.

Willmann, R. (1978). Mecoptera (Insecta, Holometabola). *Fossilum Catalogus, Animalia*, 124:1–139.

Willmann, R. (1989). Evolution und Phylogenetisches System der Mecoptera (Insecta: Holometabola). *Abhandlungen der Senckenbergischen Naturforschenden Gesellschaft*, 544:1–153.

Willmann, R. and Novokshonov, V. (1998). Neue Mecopteren aus dem oberen Jura von Karatau (Kasachstan) (Insecta, Mecoptera: 'Orthophlebiidae'). *Paläontologische Zeitschrift*, 72(3/4):281–298.

Wilson, H. M. and Martill, D. M. (2001). A new japygid dipluran from the Lower Cretaceous of Brazil. *Palaeontology*, 44(5):1025–1031.

Winkler, J. R. (1987). Berendtimiridae fam. n., a new family of fossil beetles from Baltic amber (Coleoptera, Cantharoidea). *Mitteilungen der Münchner Entomologischen Gesellschaft*, 77:51–59.

Wolf-Schwenninger, K. and Schawaller, W. (2007). 11.17 Coleoptera: beetles. In Martill, D. M., Bechly, G., and Loveridge, R. F., editors, *The Crato Fossil Beds of Brazil: Window into an Ancient World*, pages 340–350. Cambridge University Press.

Wootton, R. J. and Kukalová-Peck, J. (2000). Flight adaptations in Palaeozoic Palaeoptera (Insecta. *Biological Review*, 75:129–167.

Yao, Y.-Z., Cai, W.-Z., and Ren, D. (2004). The fossil Heteroptera of China: a review of present knowledge. *Acta Zootaxonomica Sinica*, 29:33–37.

Yao, Y.-Z., Cai, W.-Z., and Ren, D. (2006a). Fossil flower bugs (Heteroptera: Cimicomorpha: Cimicoidea) from the late Jurassic of northeast China, including a new family, Vetanthocoridae. *Zootaxa*, 1360:1–40.

Yao, Y.-Z., Cai, W.-Z., and Ren, D. (2007). *Pristinochterus* gen. n. (Hemiptera: Ochteridae) from the Upper Mesozoic of northeastern China. *European Journal of Entomology*, 104(4):827–835.

Yao, Y.-Z., Cai, W.-Z., and Ren, D. (2008). New Jurassic fossil true bugs of Pachymerididae (Hemiptera: Pentatomomorpha) from northeast China. *Acta geologica sinica (English Edition)*, 82(1):35–47.

Yao, Y.-Z., Cai, W.-Z., Ren, D., and Shih, C.-K. (2006b). New fossil rhopalids (Heteroptera: Coreoidea) from the Middle Jurassic of Inner Mongolia, China. *Zootaxa*, 1384:41–58.

Yoshimoto, C. M. (1975). Cretaceous chalcidoid fossils from Canadian amber. *The Canadian Entomologist*, 107:499–528.

Zajíc, J. and Štamberg, S. (2004). Selected important fossiliferous horizons of the Boskovice Basin in the light of the new zoopaleontological data. *Acta Musei Reginaehradecensis. Series A, Scientiae naturales*, 30:5–14.

Zalessky, G. (1955). Two new Permian dragonfly-like insects of the order Permodonata [in russian]. *Doklady Akademii Nauk SSSR*, 104(4):630–633.

Zamboni, J. C. (2001). Contribution to the knowledge of the aquatic paleoentomofauna from Santana Formation (Araripe Basin, Lower Cretaceous, northeast Brazil) with description of new taxa. *Acta Geologica Leopoldensia*, 24(52/53):129–135.

Zessin, W. (1997). *Thueringoedischia trostheiedi* nov. gen. et nov. sp. (Insecta, Orthoptera) aus dem unteren Rotliegenden von Thüringen. *Veröffentlichungen Naturkundemuseum Erfurt*, 16:172–183.

Zessin, W. (2005). Eine unwahrscheinliche Erfolgsbilanz: die Evolution der Libellen. *Virgo, Mitteilungsblatt des Entomologischen Vereins Mecklenburg*, 8(1):54–66.

Zessin, W. (2006). Zwei neue Insektenreste (Megasecoptera, Odonatoptera) aus dem Westfalium D (Oberkarbon) des Piesberges bei Osnabrück, Deutschland. *Virgo, Mitteilungsblatt des Entomologischen Vereins Mecklenburg*, 9(1):37–45.

Zessin, W. (2008). Überblick über die paläozoischen Libellen (Insecta, Odonatoptera). *Virgo, Mitteilungsblatt des Entomologischen Vereins Mecklenburg*, 11(1):5–32.

Zessin, W. (2009). *Ploetzgerarus krempieni* n. gen. et sp.Ũ eine neue Geraride (Insecta: Panorthoptera: Geraridae) aus dem Oberkarbon (Stephanium C) von Plötz bei Halle (Deutschland). *Virgo, Mitteilungsblatt des Entomologischen Vereins Mecklenburg*, 12:22–29.

Zhang, G.-X. and Hong, Y.-C. (1999). A new family Drepanochaitophoridae (Homoptera: Aphidoidea) from Eocene Fushun amber of Liaoning Province, China. *Entomologia Sinica*, 6(2):127–134.

Zhang, H.-C. and Rasnitsyn, A. P. (2008). Middle Jurassic Praeaulacidae (Insecta: Hymenoptera: Evanioidea) of Inner Mongolia and Kazakhstan. *Journal of Systematic Palaeontology*, 6(4):463–487.

Zhang, H.-C., Wang, B., and Fang, Y. (2010). Evolution of insect diversity in the Jehol Biota. *Science China Earth Sciences*, 53(12):1908–1917.

Zhang, H.-C., Wang, Q.-F., and Zhang, J.-F. (2004). Some Jurassic homopteran insects from the Junggar basin, Xinjiang, China. *Acta Palaeontologica Sinica*, 42 [for 2003](4):548–551.

Zhang, J.-F. (1986). Luanpingitidae - a new fossil insect family. *Acta Palaeontologica Sinica*, 25(1):49–54. In Chinese, English summary.

Zhang, J.-F. (1993). New Miocene species of Bibionidae (Insecta: Diptera) with discussion on taxonomic position of *Clothonopsis miocenica*. *Acta Palaeontologica Sinica*, 32(2):141–150.

Zhang, J.-F. (2002a). The most primitive fossil earwigs (Archidermaptera, Dermaptera, Insecta) from the Upper Jurassic of Nei Mongol Autonomous Region, northeastern China. *Acta Micropalaeontologica Sinica*, 19(4):348–362.

Zhang, J.-F. (2004). First description of axymyiid fossils (Insecta: Diptera: Axymyiidae). *Geobios*, 37(5):687–694.

Zhang, J.-F. (2005). The first find of chrysomelids (Insecta: Coleoptera: Chrysomeloidea) from Callovian-Oxfordian Daohugou biota of China. *Geobios*, 38(6):865–871.

Zhang, J.-F. (2006a). Jurassic limoniid dipterans from china (diptera: Limoniidae). *Oriental Insects*, 40:115–126.

Zhang, J.-F. (2006b). New mayfly nymphs from the Jurassic of northern and northeastern China (Insecta: Ephemeroptera). *Paleontological Journal*, 40(5):553–559.

Zhang, J.-F. (2007a). New Mesozoic Protopleciidae (Insecta: Diptera: Nematocera) from China. *Cretaceous Research*, 28(2):289–296.

Zhang, J.-F. (2007b). Some anisopodoids (Insecta: Diptera: Anisopodoidea) from the late Mesozoic deposits of northeast China. *Cretaceous Research*, 28(2):281–288.

Zhang, J.-F., Golub, V. B., Popov, Y. A., and Shcherbakov, D. E. (2005). Ignotingidae fam. nov. (Insecta: Heteroptera: Tingoidea), the earliest lace bugs from the upper Mesozoic of eastern China. *Cretaceous Research*, 26(5):783–792.

Zhang, J.-F. and Kluge, N. J. (2007). Jurassic larvae of mayflies (Ephemeroptera) from the Daohugou Formation in Inner Mongolia, China. *Oriental Insects*, 41:351–366.

Zhang, J.-F. and Lukashevitch, E. D. (2007). The oldest known net-winged midges (Insecta: Diptera: Blephariceridae) from the late Mesozoic of northeast China. *Cretaceous Research*, 28(2):302–309.

Zhang, J.-F. and Rasnitsyn, A. P. (2004). Minute members of Baissinae (Insecta: Hymenoptera: Gasteruptiidae) from the upper Mesozoic of China and limits of the genus *Manlaya* Rasnitsyn, 1980. *Cretaceous Research*, 25(6):797–805.

Zhang, J.-F., Sun, B., and Zhang, X. (1994). *Miocene insects and spiders from Shanwang, Shandong.* Science Press, Beijing.

Zhang, K.-Y., Li, J.-H., Yang, D., and Ren, D. (2009a). A new species of *Archirhagio* Rohdendorf, 1938 from the Middle Jurassic of Inner Mongolia of China (Diptera: Archisargidae). *Zootaxa*, 1984:61–65.

Zhang, K.-Y., Yang, D., and Ren, D. (2007). Notes on the extinct family Protapioceridae, with description of a new species from China (Insecta: Diptera: Asiloidea). *Zootaxa*, 1530:27–32.

Zhang, K.-Y., Yang, D., and Ren, D. (2008). The first Middle Jurassic *Protobrachyceron* Handlirsch fly (Diptera: Brachycera: Protobrachyceridae) from Inner Mongolia (China). *Zootaxa*, 1879:61–64.

Zhang, X.-W., Ren, D., Pang, H., and Shih, C.-K. (2009b). Late Mesozoic chresmodids with forewing from Inner Mongolia, China (Polyneoptera: Archaeorthoptera). *Acta geologica sinica (English Edition)*, 84(1):38–46.

Zhang, Z.-J., Lu, L.-W., Jin, Y.-G., Fang, X.-S., and Hong, Y.-C. (2003). Discovery of fossil insects in the Tuodian Formation, central Yunnan. *Geological Bulletin of China*, 22(6):452–455.

Zhang, Z.-L., Sun, K.-Q., and Yin, J.-R. (1997). Sedimentology and sequence stratigraphy of the Shanxi Formation (Lower Permian) in the northwestern Ordos Basin, China: an alternative sequence model for fluvial strata. *Sedimentary Geology*, 112:123–136.

Zhang, Z.-Q. (2002b). Diptera of China (Insecta): an annotated and indexed bibliography. *Fauna of China*, 4:77–224.

Zherikhin, V. V. (1993). Podotryad Polyphaga [Suborder Polyphaga]. *Trudy Paleontologicheskogo instituta Akademiia nauk SSSR*, 252:20–37.

Zherikhin, V. V. (2000). Tertiary brachycerid weevils (Coleoptera: Brachyceridae) from the collections of Muséum Nationale d'Histoire Naturelle, Paris, with a review of other fossil Brachyceridae. *Paleontological Journal*, 34(Suppl. 3):S333–S343.

Zherikhin, V. V. (2002a). 2.2.1.2.4.3. Order Thripida Fallen, 1914. (=Thysanoptera Haliday, 1836) The thrips. In Rasnitsyn, A. P. and Quicke, D. L. J., editors, *History of Insects*, pages 133–143. Kluwer Academic Publishers, The Netherlands.

Zherikhin, V. V. (2002b). 2.2.2.1.3. Order Mantida Latreille, 1802. The Mantises (=Mantodea Burmeister, 1838). In Rasnitsyn, A. P. and Quicke, D. L. J., editors, *History of Insects*, pages 273–276. Kluwer Academic Publishers, The Netherlands.

Zherikhin, V. V. (2002c). 3.2 Ecological history of the terrestrial insects. In Rasnitsyn, A. P. and Quicke, D. L. J., editors, *History of Insects*, pages 331–388. Kluwer Academic Publishers, The Netherlands.

Zherikhin, V. V. and Gratshev, V. G. (1994). Obrieniidae, fam. nov., the oldest Mesozoic weevils (Coleoptera, Curculionoidea). *Paleontological Journal*, 27(1A):50–69.

Zherikhin, V. V. and Gratshev, V. G. (2004). Fossil curculionoid beetles (Coleoptera, Curculionoidea) from the Lower Cretaceous of northeastern Brazil. *Paleontological Journal*, 38(5):528–537.

Zhou, C.-F. and Peters, J. G. (2003). The nymph of *Siphluriscus chinensis* and additional imaginal description: a living mayfly with Jurassic origins (Siphluriscidae new family: Ephemeroptera). *Florida Entomologist*, 86(3):345–352.

Zlobin, V. V. (2007). The fossil limestone cyclorrhaphous Diptera Limestone of the Isle of Wight. *International Journal of Dipterological Research*, 18(2):129–136.

Zompro, O. (2001). The Phasmatodea and *Raptophasma* n. gen., Orthoptera incertae sedis, in Baltic amber (Insecta: Orthoptera). *Mitteilungen aus dem Geologisch-Paläontologischen Institut der Universität Hamburg*, 85:229–261.

Zompro, O. (2005). Inter- and intra-ordinal relationships of the Mantophasmatodea, with comments on the phylogeny of polyneopteran orders (Insecta: Polyneoptera). *Mitteilungen aus dem Geologisch-Paläontologischen Institut der Universität Hamburg*, 89:85–116.
